# High-Efficiency Light Emitters: 10-(Diphenylphosphoryl)-anthracenes from One-Pot Synthesis Including C–O–P to C–P(=O) Rearrangement

**DOI:** 10.1021/acs.joc.4c03139

**Published:** 2025-03-25

**Authors:** Vivek Vivek, Marek Koprowski, Ewa Różycka-Sokołowska, Marika Turek, Bogdan Dudziński, Krzysztof Owsianik, Łucja Knopik, Piotr Bałczewski

**Affiliations:** † Division of Organic Chemistry, Centre of Molecular and Macromolecular Studies, Polish Academy of Sciences, Sienkiewicza 112, Łódź 90-363, Poland; ‡ The Bio-Med-Chem Doctoral School of the University of Łódź and Łódź Institutes of the Polish Academy of Sciences, University of Łódź, Matejki 21/23, Łódź 90-237, Poland; § Institute of Chemistry, Faculty of Science and Technology, Jan Długosz University in Częstochowa, Armii Krajowej 13/15, Częstochowa 42-201, Poland

## Abstract

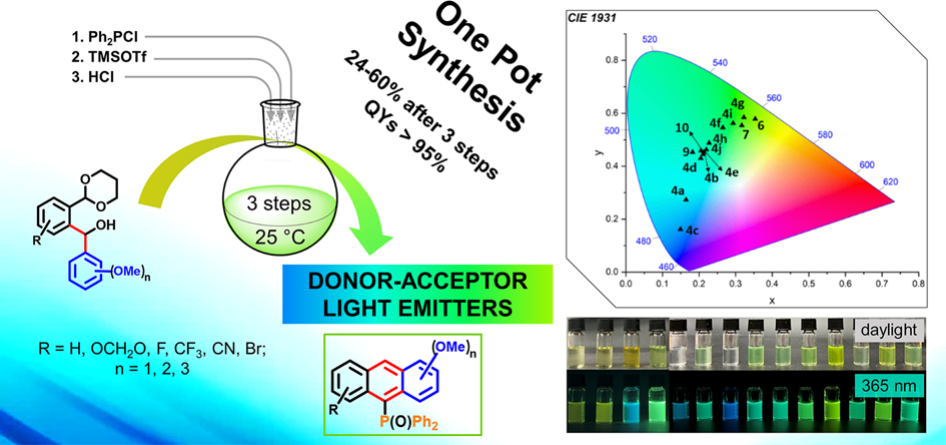

We report a one-pot synthesis of 10-(diphenylphosphoryl)-anthracenes, featuring a rare multisubstitution on flanking rings with donor–acceptor groups (F, Br, CN, CF_3_, MeO, OCH_2_O) in 24–60% yields. Catalyzed by TMSOTf, the process involves a phosphinite-to-phosphine oxide rearrangement and cyclization. These emitters exhibit excellent photoluminescence quantum yields of up to 95% in both solution and solid states. Postsynthetic anthracene functionalization as well as the optoelectronic effect of substituents, particularly the Ph_2_P=O group, and the aggregation effect in solid on the photophysical properties, were also explored.

## Introduction

Organic luminescent compounds have been the subject of current research due to their potential applications in organic light-emitting diodes (OLEDs),^1^ organic field-effect transistors (OFETs),^2^ sensors,^3^ and lasers.^4^ Many studies focus on organic-emitting materials based on fused aromatic hydrocarbons, such as anthracenes,^5−7^ pyrenes,^8^ benzimidazoles,^9^ fluorenes,^10^ fluoranthenes,^11^ and carbazoles,^12^ which are more stable than pentacenes or extended analogs. However, if appropriately substituted, they can exhibit reasonable stability and high fluorescence quantum yields and are therefore promising materials for organic electronic devices. Several reports have shown that anthracenes are useful for constructing efficient light-emitting materials.^13−15^ However, unsubstituted or poorly substituted anthracenes are unsuitable for direct use because they tend toward π–π stacking and crystallization, which results in low quantum yields. An effective approach to terminate the π–π stacking involves chemical modification by substitution of the anthracene core.^16−19^ Most of the known anthracenes are substituted at positions 9 and 10 only,^5b^ since electron density mapping of the anthracene aromatic system shows that the highest electron density occurs just at these positions. There is a lack of synthetic methods for introducing substituents into other positions, such as the flanking rings of anthracene, to fully exploit the potential of this aromatic system.

Traditionally, 10-substituted or 9,10-disubstituted anthracenes by dipenylphosphoryl group (Ph_2_P=O) are obtained from existing aromatic backbones by the reaction of chlorodiphenylphosphine with the corresponding lithio-anthracenes obtained via the Br/Li exchange followed by oxidation of the resulting P^III^ derivative with hydrogen peroxide.^20^

In this work, we present a one-pot, three-step synthesis of highly substituted anthracenes possessing the Ph_2_P=O group at position 10 and other substituents of differentiated electron character on the flanking rings. The presence of the electron-withdrawing Ph_2_P=O group lowers the energy levels of the neighboring π-electron system.^21,22^ In addition, the Ph_2_P=O group behaves as a Lewis basic site for complexing metals, cations, and protons through coordination, electrostatic, and hydrogen-bonding interactions.^23−25^ In the field of materials, the Ph_2_P=O group is used for constructing emitters, host matrices, and electron-transporting materials (ETMs) in OLEDs with excellent performance in all applications.^26−29^

### Background

The Michaelis–Arbuzov rearrangement is one of the key reactions in organophosphorus chemistry for the formation of phosphonates, phosphinates, and phosphine oxides. Wiemer et al. have reported a modification of this reaction for the synthesis of phosphonates from benzylic and allylic alcohols using Lewis acids,^30,31^ which has recently been modified by our group to synthesize diarylmethylphosphonates in excellent yields from diarylmethyl alcohols **1a**–**j** in the presence of ZnI_2_.^30−32^ However, analogous attempts to directly synthesize diarylmethyl phosphine oxides **3a**–**j** using diarylmethanols **1a**–**j** and ethyl-diphenylphosphinite (EtO-PPh_2_) instead of trialkyl phosphites, failed due to the rearrangement of the starting ethyl diphenylphosphinite (EtO-PPh_2_) to ethyl diphenyl phosphine oxide (Et-P(O)Ph_2_) under the influence of heat and Lewis acid (ZnI_2_). Renard et al. have also shown a Lewis-acid-catalyzed rearrangement of phosphinites to phosphine oxides using trimethylsilyl triflate (TMSOTf) and BF_3_·OEt_2_.^33^

**Scheme 1:**
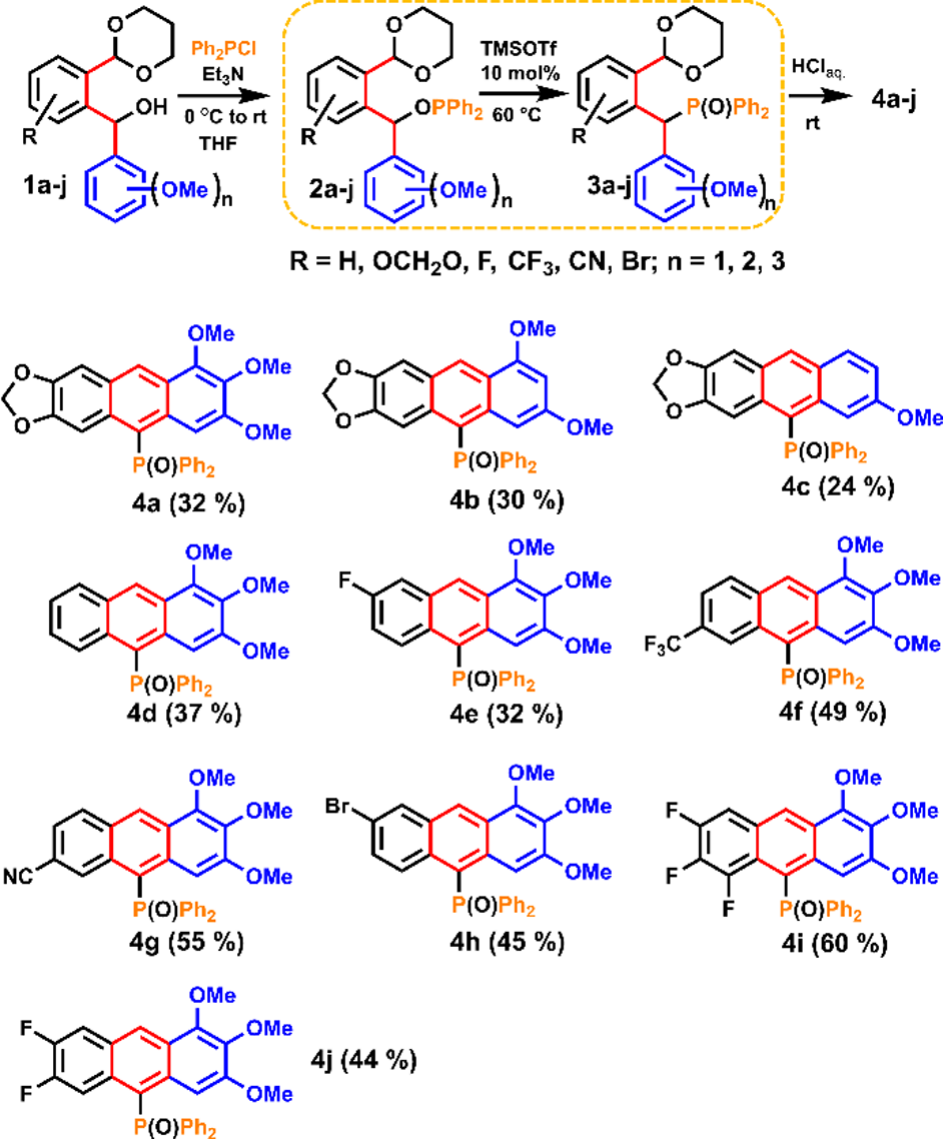
Synthesis of Donor–Acceptor 10-(Diphenylphosphoryl)-anthracenes **4a**–**j**

This inspired us to convert diarylmethyl alcohols **1a**–**j** to diarylmethyl phosphinites **2a**–**j** and then to rearrange the latter to diarylmethyl phosphine oxides **3a**–**j** with a catalytic amount of TMSOTf followed by cyclization to anthracenes **4a**–**j** (Scheme 1). This idea has been successfully realized as a one-pot process without separating intermediate products **2a**–**j** and **3a**–**j**. All obtained anthracenes **4a**–**j** were fully characterized, and their potential in organic electronics as light emitters has been shown.

## Results and Discussion

### Synthesis

The three-step, one-pot synthesis of multiply substituted anthracenes **4a**–**j** started from diarylmethyl alcohols **1a**–**j**, which were synthesized from 1-(1,3-dioxanyl)-2-bromo benzaldehydes according to our previous protocol.^19^

**Scheme 2:**
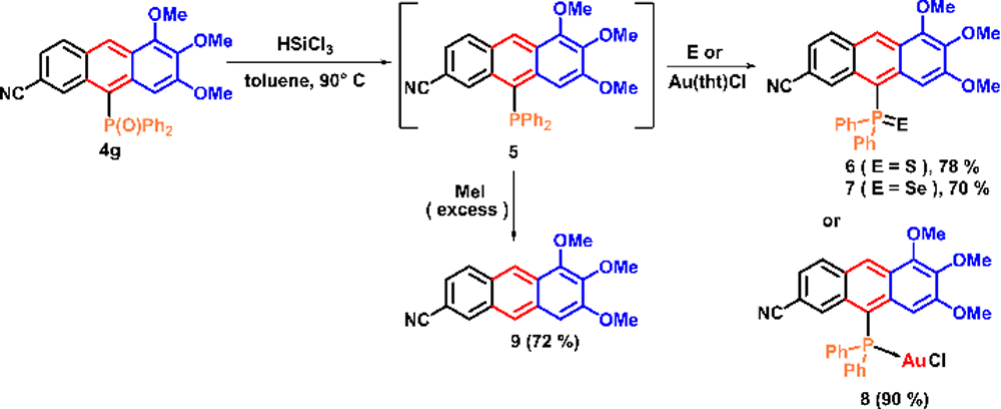
Syntheses of the Phosphine **5**, the Phosphine Sulfide **6**, the Phosphine Selenide **7**, the Au^I^ Complex **8**, and the Dephosphorylated Derivative **9**

The alcohols **1a**–**j** were converted into phosphinites **2a**–**j** with triethylamine (NEt_3_) and chlorodiphenylphosphine (PPh_2_Cl) at room temperature, followed by the addition of TMSOTf (10 mol %) to rearrange the resulting phosphinites **2a**–**j** to phosphine oxides **3a**–**j** at 60 °C [C–O–P to C–P(=O)] according to Scheme 1 (reaction mechanism, Scheme 1S, SI). Treatment of the latter with aqueous HCl (12 N) under mild conditions resulted in the formation of easily separable anthracenes **4a**–**j** in 24–60% yields after three steps, which gave an average of 70–85% per step (reaction mechanism, Scheme 2S, SI).

A further modification of the anthracene moiety was carried out starting with P^III^ derivative **5** (Scheme 2). Thus, the in situ reductive deoxygenation of the phosphine oxide **4g** (δ_31P_ = 29.8 ppm, CD_2_Cl_2_) with trichlorosilane in toluene resulted in the formation of the phosphine **5** (δ_31P_ = −24.2 ppm, CD_2_Cl_2_), which was subsequently converted into the phosphine sulfide **6** in 78% yield (δ_31P_ = 33.9 ppm, CD_2_Cl_2_) and the phosphine selenide **7** in 70% yield (δ_31P_ = 25.2 ppm, CD_2_Cl_2_) by treatment of the former with elemental sulfur and selenium, respectively, in refluxing toluene. Treatment of the phosphine derivative **5** with chloro(tetrahydrothiophene)gold, [Au^I^(tht)Cl] at room temperature for 2h, resulted in the formation of the gold(I) complex **8** in 90% yield (δ_31P_ = 23.3 ppm, CD_2_Cl_2_) (Scheme 2).^34,35^

Compounds **5** and **8** were unstable in air and could be easily oxidized back to phosphine oxide **4g**. Furthermore, when **4g** was reduced to **5** with an excess of HSiCl_3_ followed by treatment with an excess of iodomethane, unexpectedly, a dephosphorylated compound **9** was formed in 72% yield, instead of the expected phosphonium salt, as a result of the *ipso* attack of the Si–H bond, facilitated by the presence of the 6-CN group (reaction mechanism, Scheme S3, SI).

**Scheme 3:**
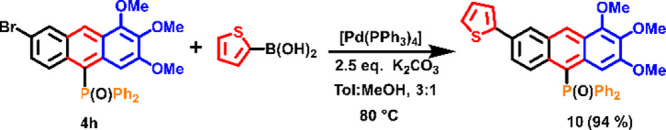
Synthesis of the 7-Thienyl Derivative **10**

Postsynthesis functionalization of the anthracene backbone to further enhance π-conjugation was demonstrated by the synthesis of the 7-thienyl derivative **10** in 94% yield, using the Suzuki - Miyaura cross-coupling reaction of 7-Br-**4h** with 2-thienylboronic acid (Scheme 3).^36^

### Crystal Structures

X-ray diffraction measurements for crystals of anthracenes **4b**, **4j**, and **9**, obtained from CH_2_Cl_2_ (DCM) solutions revealed that the anthracene **9** crystallized as solvent-free form (Figure S81 in SI) while **4b** and **4j** as DCM solvates, **4b**·**DCM** and **4j**·**DCM** (Figures S82–S83 in SI)). Crystal structure data are given in the Supporting Information, SI.

### Photophysical Properties in Solution and Solid

**Figure 1:**
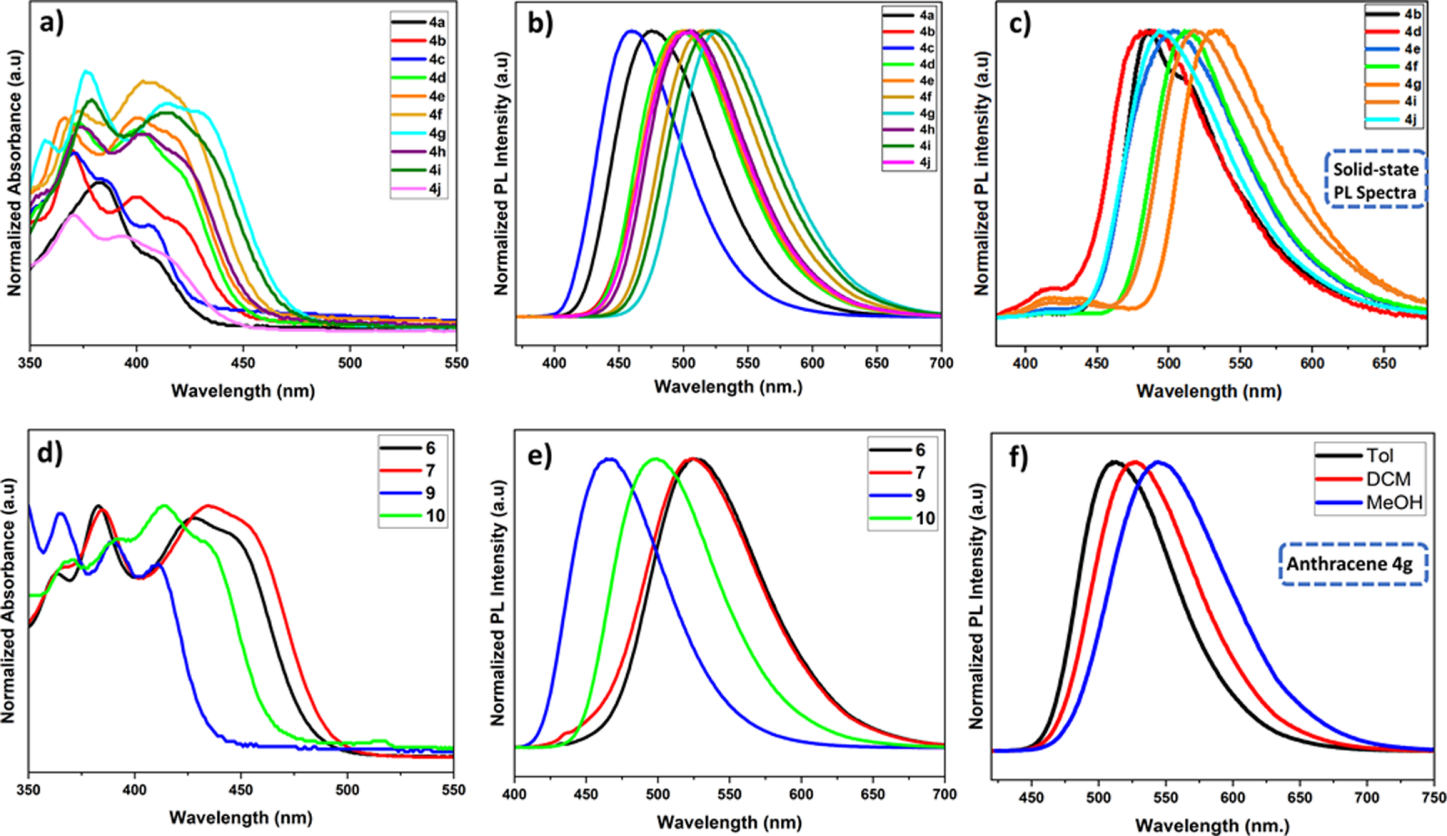
(a,b) Normalized absorption and emission spectra of anthracenes **4a**–**j** (10^–5^ mol/L, DCM); (c) normalized emission spectra of selected anthracenes in solid-state; (d,e) normalized absorption and emission spectra of anthracenes **6**, **7**, **9**, and **10** (10^–5^ mol/L, DCM); (f) normalized emission spectra of the anthracene **4g** in three different solvents (10^–5^ mol/L).

The ultraviolet–visible (UV–vis) absorption, photoluminescent emission (PL), and quantum yield data of anthracenes **4a**–**j**, **6**, **7**, **9,** and **10** in three different solvents (toluene, DCM, and methanol) are given in Table 1. As shown in Figure 1a, the absorption spectra of the substituted anthracenes **4a**–**j** exhibited intense bands in the 360–430 nm range. The lower energy absorption was assigned to the S_0_ → S_1_ transition and had the typical π → π* character, known for many anthracene derivatives.^37−40^ The anthracenes **4a**–**j** emitted blue to yellow-green light in a 450–550 nm range, in DCM solutions (Figure 1b).

**Table 1:** Photophysical Data for Anthracenes **4a**–**j**, **6**, **7**, **9**, and **10** in Different Solvents

	toluene				DCM				MeOH			
Nr	Abs.^a^ λ_max._ (nm)	PL^b^ λ_max._ (nm)	υ^c^ (cm^–1^)	QY^d^ (%)	Abs.^a^ λ_max._ (nm)	PL^b^ λ_max._ (nm)	υ^c^ (cm^–1^)	QY^d^ (%)	Abs.^a^ λ_max._ (nm)	PL^b^ λ_max._ (nm)	υ^c^ (cm^–1^)	QY^d^ (%)
4a	409	468	3082	60.5	409	476	3442	71.7	412	490	3863	51.4
**4b**	418	484	3262	69.9	421	499	3712	81.4	426	514	4018	63.9
**4c**	408	452	2385	44.7	408	460	2770	40.2	408	470	3233	31.8
**4d**	423	489	3190	66.6	422	498	3616	86.1	422	511	4127	60.7
**4e**	419	490	3458	69.9	420	500	3809	83.3	420	516	4429	58.4
**4f**	420	502	3889	71.4	421	514	4297	87.2	421	529	4849	56.7
**4g**	427	513	3926	82.5	432	527	4172	94.8	434	546	4726	34.4
**4h**	423	495	3438	72.1	424	505	3346	80.0	424	519	4317	64.8
**4i**	429	511	3740	64.4	430	521	4062	72.2	430	537	4633	37.7
**4j**	414	495	3952	72.7	413	503	4332	86.5	415	519	4828	57.6
**6**	447	513	4030	14.6	448	527	4389	15.8	446	538	4832	14.6
**7**	453	511	3420	1.9	456	524	3957	2.0	452	539	4543	2.1
**9**	411	451	5149	48.9	411	466	5938	71.7	406	471	6317	37.2
**10**	436	488	3604	46.0	439	499	4115	62.4	437	509	4392	64.5

a Abs. (λ_max._)—absorption maximum.

b PL (λ_max._)—emission maximum.

c Stokes shift, (υ) = 1/λ_abs_ – 1/λ_em_.

d The absolute photoluminescence quantum yield (QY).

The values of absorption maxima for anthracenes **4a** (409 nm), **4b** (418 nm), and **4c** (408 nm) with electron-donating 1,2,3-tri-MeO, 1,3-di-MeO, and 3-MeO groups, respectively, did not arrange themselves in a logical order depending on the number of MeO groups (Figure 1a). Similarly, no trend was observed in emission maxima of **4a**–**c** (λ_max_ = 476, 499, and 460 nm, respectively) (Figure 1b). However, the absorption maxima of **4f** (420 nm) and **4g** (427 nm), containing electron-withdrawing 6-CF_3_ and 6-CN groups, respectively, absorbed, as expected, lower than all three anthracenes **4a**–**c** with electron-donating MeO groups (Figure 1a). The same trend was observed in emission wavelengths of **4f** and **4g** (λ_max_ = 514 and 527 nm, respectively) versus that of **4d** (498 nm) (Figure 1b).

The anthracenes **4e** (419 nm) and **4h** (423 nm) with halogen 7-F and 7-Br substituents showed redshifts compared to that of the unsubstituted anthracene **4d** (422 nm) on the left flanking ring (Figure 1a). This was also the case with the wavelengths of light emission (Figure 1b). The values of absorption maxima for the multiply substituted fluoro anthracenes **4e** (419 nm), **4j** (414 nm), and **4i** (429 nm) with one 7-F, two 6,7-di-F and three 5,6,7-tri-F electron-withdrawing groups on the left flanking ring, respectively, again did not form a clear order in relation to the number of fluorine groups, as in the case of emission maxima (Figure 1a,b). On the other hand, all three fluoro anthracenes **4e**, **4i**, and **4j** showed redshifts compared to the unsubstituted anthracene **4d** in both absorption and emission spectra due to enhancing the donor–acceptor character of the whole conjugated system.

The absorption maxima of nitrile-substituted anthracenes 6-CN-**4g**, 6-CN-**6**, and 6-CN-**7** with 10-Ph_2_P=O (432 nm), 10-Ph_2_P=S (448 nm), and 10-Ph_2_P=Se (456 nm) groups on the anthracene backbone (Table 1), respectively, revealed all redshifts compared to the 10-unsubstituted anthracene **9** (411 nm) and a clear trend due to the increasing polarizability of P=O > P=S > P=Se bonds which made electron excitation easier (Figure 1d). Interestingly, the emission wavelengths of **4g**, **6,** and **7** showed practically no difference in toluene and DCM (Table 1, Figure 1e).

The anthracene **10** with 7-thienyl substituent, showed a higher absorption maximum (439 nm) than the starting 7-bromo anthracene **4h** (424 nm), as expected, due to the extension of the conjugate system (Figure 1d). In contrast, the latter (λ_max_ = 505 nm) emitted at a higher wavelength than anthracene **10** (λ_max_ = 498 nm) containing the 7-thienyl substituent (Figure 1e).

The emission properties of the anthracene derivatives were solvent-dependent.^5b^ Most anthracenes showed redshifts in more polar solvents, such as methanol, compared to less polar toluene and DCM (Figure 1f, Table 1). A comparison of emission maxima of 10-unsubstituted anthracene **9** (466 nm) and the anthracene 6-CN-**4g** (527 nm) showed the largest red-shift of 61 nm, indicating a significant effect of the 10-Ph_2_P=O group on the optical properties (Table 1).

The emission properties were also studied in the solid state (Figure 1c). The anthracenes **4e** (λ_max_ = 503 nm), **4f** (λ_max_ = 513 nm), and **4g** (λ_max_ = 533 nm) with electron-withdrawing 7-F, 6-CF_3_, and 6-CN groups showed the largest redshifts compared to the unsubstituted **4d** (λ_max_ = 483 nm) on the left flanking ring (Table S1, see SI). Like in solution, the synthesized anthracenes showed no trends in the solid-state emission maxima.

### Effect of Substituents on Quantum Yields (QYs) in Solution

**Figure 2:**
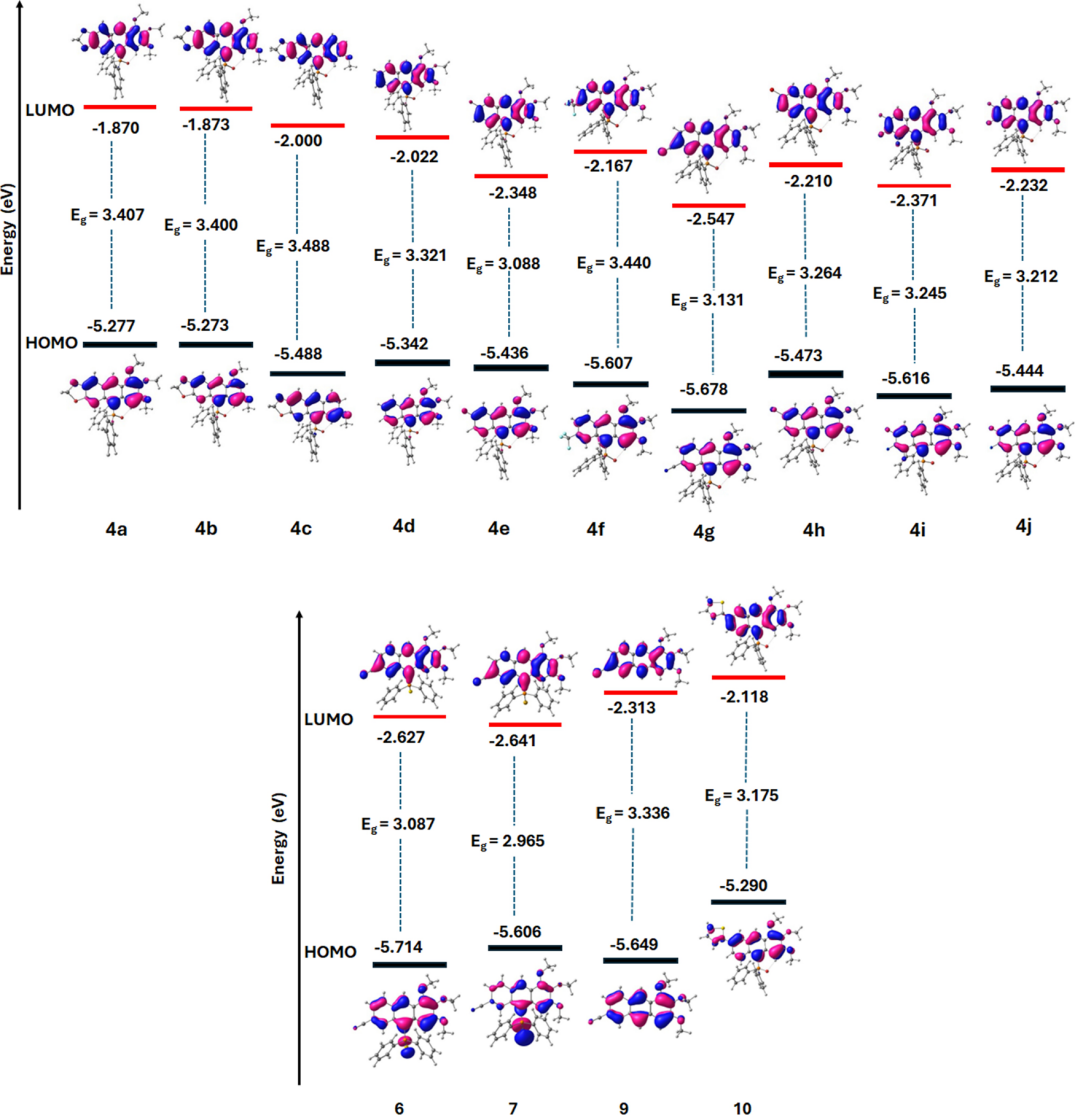
Molecular orbitals calculated for anthracenes **4a**–**j**, **6**, **7**, **9**, and **10** at DFTB3LYP/6-311++G(d,p).

The anthracenes **4a**–**4j** generally showed very good QYs with an average value of 78.3% for ten compounds, and the highest QY of 94.8% for the anthracene 7-CN-**4g** (all in DCM) (Table 1). The presence of many electron-donating groups (MeO, OCH_2_O) with electron (+M) effect, as in **4a**, weakened the donor–acceptor character of the investigated anthracenes in comparison to anthracene **4d** and anthracenes **4e**–**4j** with electron-withdrawing substituents. This resulted in a reduction in QYs (**4a**: 71.7 versus **4d**: 86.1 and **4e**–**4j**: 72.2–94.8%, all in DCM; Table 1). Moreover, in general, *E*_LUMO_, *E*_HOMO_, and *E*_g_ for **4d** and **4e**–**4j** were lower than those for **4a** (Figure 2, Table S17 in SI). High QYs observed in DCM, were strongly perturbed in MeOH due to the fluorescence quenching caused by the intermolecular P=O···H–O and, in the case of 7-CN-**4g**, additionally by C≡N···H–O hydrogen bonds.

Anthracenes **6** and especially **7** with Ph_2_P=S and Ph_2_P=Se groups, respectively, emitted much less efficiently than anthracenes **4a**–**4j** with the Ph_2_P=O group despite favorable *E*_g_ and *E*_LUMO_ values for the former (**6**/3.087, −2.627 eV; **7**/2.965, −2.641 eV, respectively, versus, e.g., **4g**/3.131, −2.547 eV; Figure 2, Tables S17 and S18 in SI).

**Figure 3:**
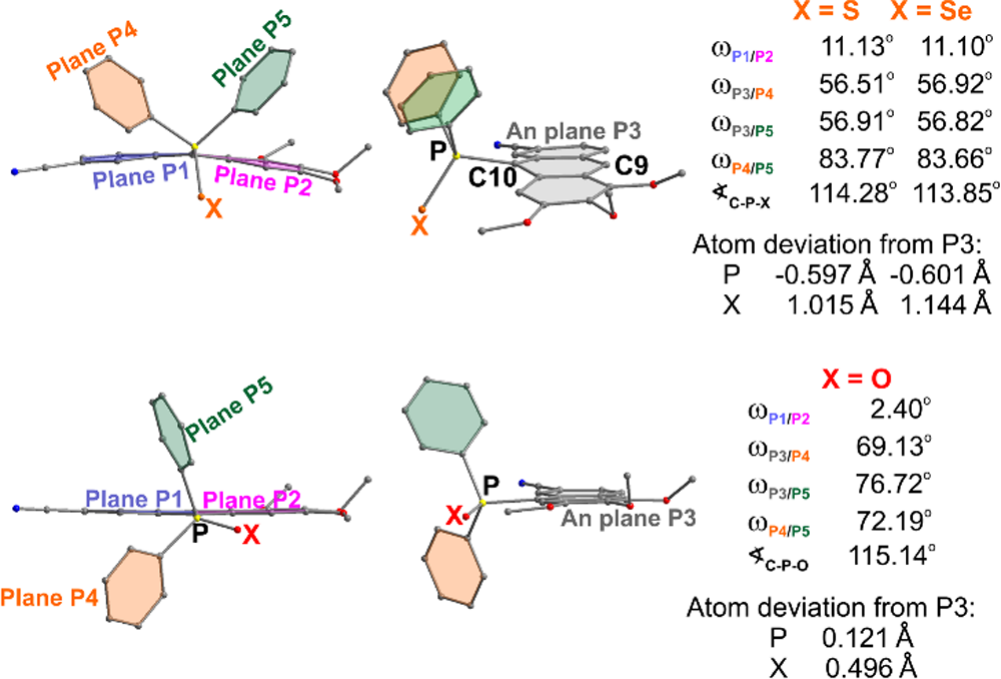
Molecular structures of anthracenes **6**, **7**, and **4g**, illustrating key geometric differences influencing the fluorescence QY.

Analysis of the molecular geometries of **6**, **7**, and their oxygen analog **4g** revealed that, unlike the P=O group, the presence of P=S and P=Se groups caused butterfly bending of the anthracene plane (also observed in other anthracenes)^35^ with the pronounced butterfly angle at the C9–C10 axis (ω_P1/P2_ is approximately 11° in **6** and **7** compared to 2° in **4g**) (Figure 3), thereby disturbing π-conjugation within the anthracene core. In compounds **6** and **7**, the dihedral angles between planes containing phosphine phenyls (P4 and P5 planes in Figure 3) and anthracene plane (i.e., ω_P3/P5_ and ω_P4/P5_), were notably larger than in **4g** (Figure 3). Additionally, the relative orientation between P4 and P5 planes in the Ph_2_P=S and Ph_2_P=Se groups exhibited increased twisting compared to that in the Ph_2_P=O group (Figure 3). In **6** and **7**, these planes are on the same side of the anthracene plane, while in **4g** they are on opposite sides of this plane.

Moreover, the P=O substituent in **4g** remained nearly coplanar with the anthracene plane, in contrast to the P=S and P=Se substituents in **6** and **7**, which were out of the plane. Both the differences in the situation of the P=X substituents relative to the anthracene plane and the butterfly bending causing a partial disturbance of π-conjugation, and additionally the heavy atom effect in the case of S and Se,^41^ were the reason for the significant decrease in quantum yields to the level of 15 and 2%, respectively.

The fluorescence lifetimes of **4b**, **4d**–**4g**, **4i**, and **4j** were within the typical range of 15.3 ns for 1,3-di-MeO-**4b** to 23.3 ns for 7-CN-**4g** in the same solvent (Table S1 in SI). In the solid, the highest QY, exceeding 95% was shown by the anthracene 7-CF_3_-**4f**, which again was the highest value in the group of anthracenes known in the literature (Table S1 in SI).

The remarkable effect of the Ph_2_P=O group on the emission properties of the obtained anthracenes is related to its high electron-withdrawing power, which promotes the formation of strong donor–acceptor systems. The measure of this effect is the Hammett σ_p_ constant, calculated with ACD/Percepta^42^ for Ph_2_P=O (σ_p_ = 0.6) which corresponds to the high σ_p_ values of other powerful electron-withdrawing CN and CF_3_ groups (σ_p_ = 0.66/0.51, respectively). Analysis of the σ_ind_ and σ_res_ components, which are positive, shows that inductive (-I) and resonance (-M) effects are both responsible for the electron-withdrawing character of the Ph_2_P=X groups (X=O: σ_ind_/σ_res_ = 0.26/0.34; X=S: σ_ind_/σ_res_ = 0.28/0.22).

For the Ph_2_P=O group, the resonance (-M) effect predominates over the inductive (-I) effect while the opposite is true for the Ph_2_P=S group. Electron-donating MeO groups in the donor–acceptor systems show σ_p_ = −0.27 and σ_ind_/σ_res_ = 0.30/–0.58 which means that the (+M) effect almost doubles the (-I) effect. It is interesting that fluorine, as the most electronegative element, shows the lowest sigma σ_p_ = 0.06 value among electron-withdrawing groups, which is due to the small difference between the σ_ind_ = 0.54 and σ_res_ = −0.48 values. This indicates only a slight predominance of the -I effect over the + M effect and a relatively significant contribution of the resonance structure with the fluorine group to the resonance hybrid.

### Color Space Studies

**Figure 4:**
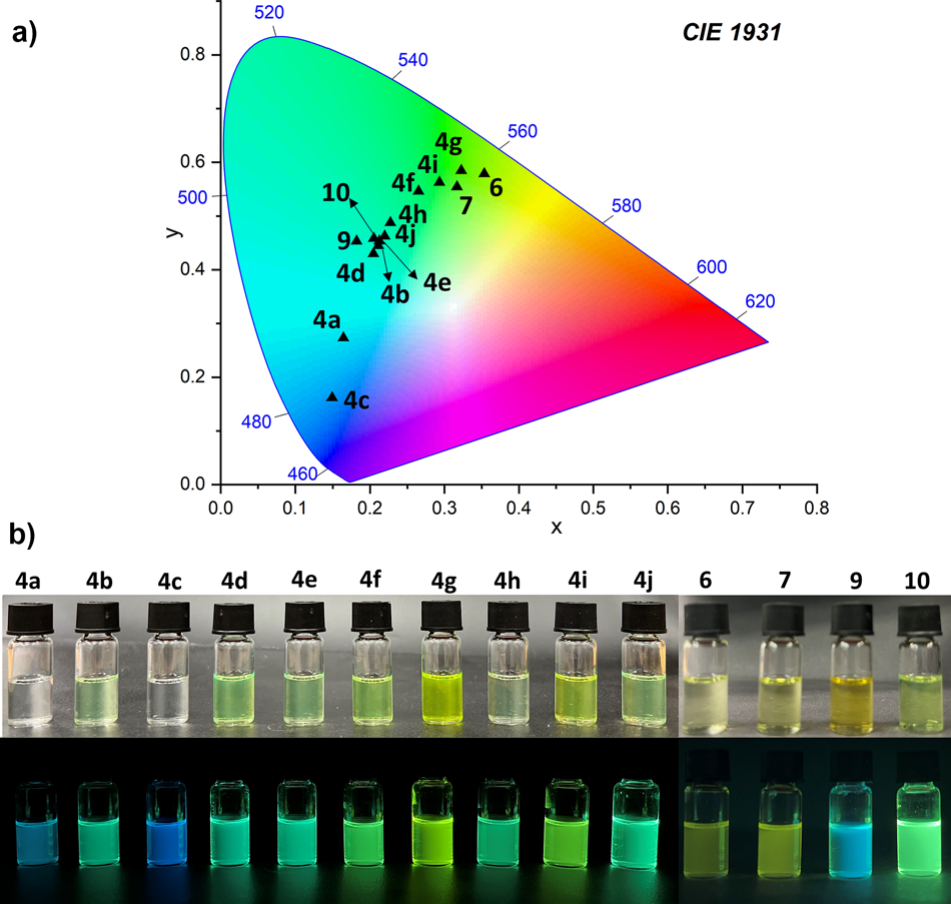
(a) Commission Internationale de L’Eclairage (CIE) 1931 chromaticity coordinates for anthracenes **4a**–**j** and **6–10** in DCM solutions; (b) photos of the anthracene **4a**–**j** and **6–10** samples in DCM solutions under daylight and UV (365 nm) light (bottom).

All anthracene phosphine oxides **4a–j** emit blue to yellow-green light in DCM solutions (Figure 4: **4a**, **4b**) Particularly striking is the rapid color change from blue-green (3xMeO), through green (2xMeO) toward again deep blue (1xMeO) for anthracenes **4a**–**c** differing only in the number of methoxy groups (Figure 4: **4b**). The CIE 1931 color space chromaticity coordinates of anthracenes **4a**–**j**, **6**, **7**, **9,** and **10** in DCM, toluene, and MeOH are provided in SI (Table S19).

### Aggregation Effects on the Photophysical Properties of **4j** and **4b** in Solids

For most of the compounds (**4b**, **4d**, **4e**, and **4j**), we observed that the QY in the DCM solution was much higher (about 81–87%) than in the solid state (about 37–47%). This indicated that various intermolecular interactions operated in solid, which led to molecular aggregation causing emission quenching (ACQ: Aggregation-Caused Quenching).^43,44^ The compound **4i** showed a dramatically low QY in the solid state (13.3%) with a much higher QY in the DCM (72.2%), indicating the strongest ACQ effect. The exceptions were **4f** and **4g**, which retained very high emissions in the solid state (>95 and 79.2%, respectively), suggesting an aggregation-induced emission (AIE) phenomenon^44,45^ in which aggregation reduced nonemission relaxation pathways, increasing QY. To determine the effect of aggregation in the solid state on emission properties, and in particular the significantly lower QY, we analyzed intermolecular interactions, molecular packing, and the possibility of excimer formation in crystals **4j** and **4b** (both DCM solvated).

**Figure 5:**
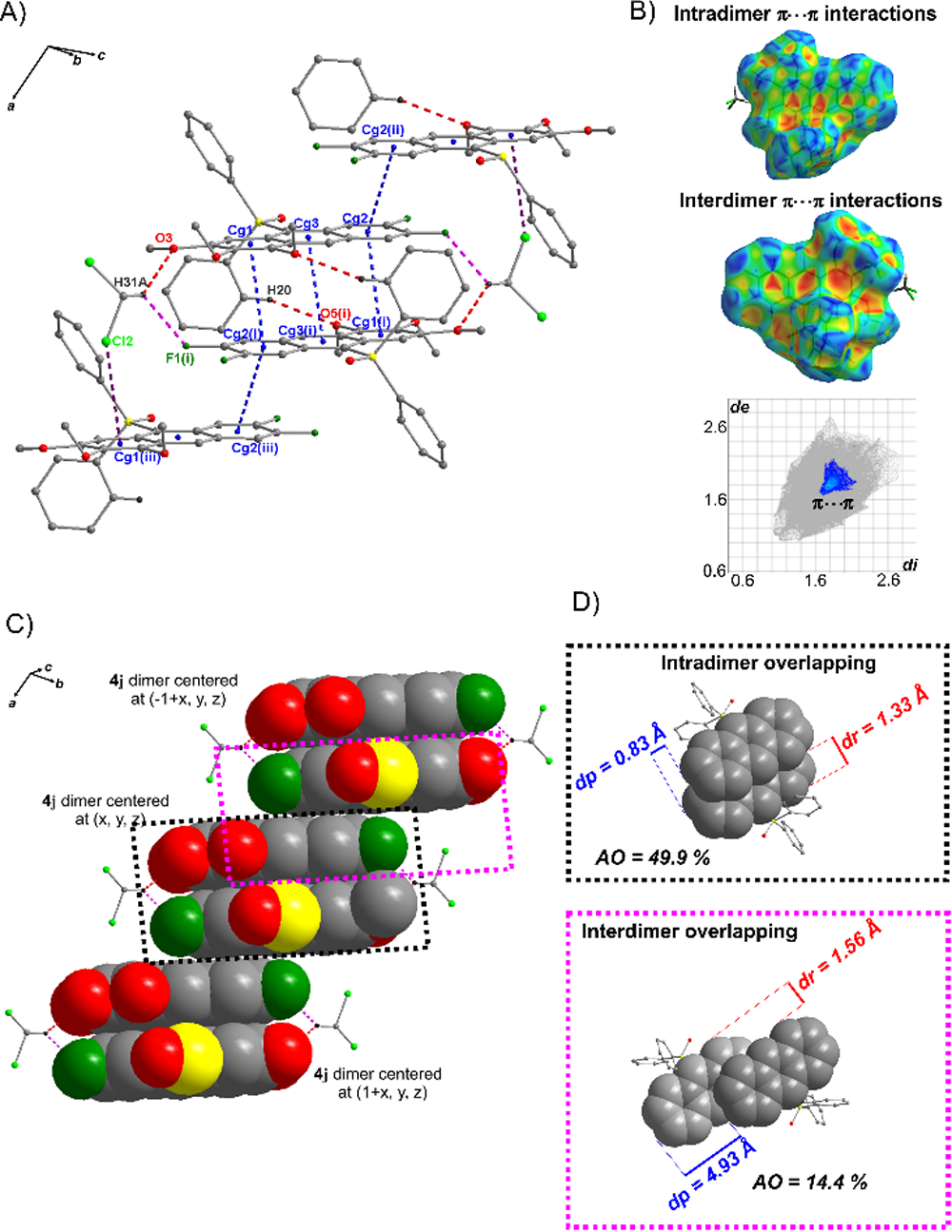
(A) Part of the crystal structure of **4j·DCM** showing key intermolecular interactions, including π···π interactions (blue dashed lines), hydrogen bonds (red and pink dashed lines), and C–Cl···π interaction (gray dashed line); the centroids of rings are denoted by small blue circles, and all H atoms not involved in the hydrogen bonds have been omitted for clarity. Symmetry codes (i)–(iii) are given in Table S2 in SI. (B) Molecular Hirshfeld surface mapped with shape index, and the corresponding fingerprint plot,^47^ showing π···π interactions. (C) Arrangement of **4j** dimers in stack parallel to the *a* axis. (D) Quantification of intra- and interdimer π–π overlapping, with associated AO and displacement pitch and roll parameters (dp and dr).^18^

**Figure 6:**
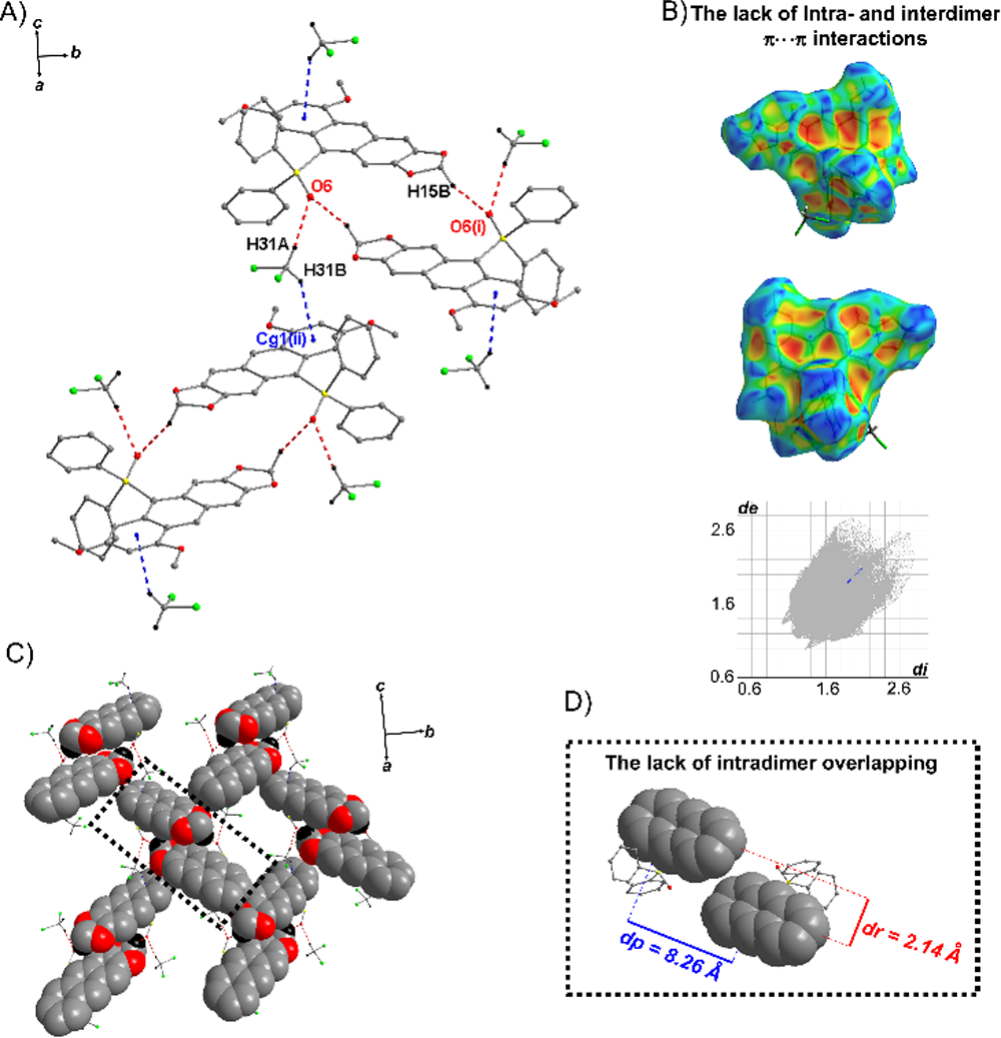
(A) Part of the crystal structure of **4b·DCM** showing key intermolecular interactions, including hydrogen bonds (red and pink dashed lines) and C–H···π interactions (blue dashed lines); the centroids of benzene rings are denoted by small blue spheres, and all H atoms not involved in the hydrogen bonds have been omitted for clarity. Symmetry codes (i)–(ii) are given in Table S2 in SI. (B) Molecular Hirshfeld surface mapped with the shape index and the corresponding fingerprint plot,^47^ showing the lack of π···π interactions. (C) Arrangement of **4b** dimers in layer parallel to the (101) plane. (D) Dimer of **4b** formed by C–H···O hydrogen bond, with dp and dr parameters.^18^

In the crystal structure of **4j·DCM**, three π···π interactions between anthracene units link molecules into the centrosymmetric dimer (Figure 5, Figure S85 and Table S2 in SI), showing a 49.9% area overlapping (AO) (Figure 5D), calculated using the phenomenological approach proposed by Curtis et al.^18^ in combination with a simple model introduced by Janzen et al.^46^ These π···π interactions are revealed on the three-dimensional, molecular Hirshfeld surface (HS) as three pairs of blue and red triangles and on the two-dimensional fingerprint plot (FP) as light blue points lying on the diagonal at around di = de = ∼1.8 Å (Figure 5B), both obtained using CrystalExplorer.^47^ The dimer is additionally stabilized by one weak C–H···O hydrogen bond and by two DCM molecules through two weak hydrogen bonds, i.e., (C–H)_DCM_···O_4j_ and (C–H)_DCM_···F_4j_ (Figures 5A and S85 in SI). Furthermore, the molecules of neighboring dimers are connected by a single π···π interaction (also revealed on HS and FP) and a (C–Cl)_DCM_···π interaction linking them in a stack running parallel to the *a*-axis (Figures 5A,C and S85). A slight blue shift of 9 nm relative to the solution indicates that despite stacking arrangement in solid, the π···π interactions between molecules are too weak to fully develop the excimer state with a characteristic, strong red shift. A decrease in fluorescence QY from 86.5% in solution to 36.6% in the solid state (Table S1 in SI) indicates that aggregation leads to ACQ. In contrast to that of **4j·DCM**, the crystal structure of **4b·DCM** reveals a markedly different mode of aggregation. Here, two molecules are connected solely by a single C–H···O=P hydrogen bond to form a dimer (Figures 6A and S84), with no significant π···π interactions (Figure 6A,B). Each dimer is further linked to four neighboring dimers via DCM molecules through two weak (C–H)_DCM_···O_4b_ and (C–H)_DCM_ ···π_4b_ hydrogen bonds, leading to the formation of a layer parallel to the (101) plane (Figure 6A,C). The lack of π–π stacking interactions in the crystal structure (Figure 6B), combined with the blue shift of the emission maximum (from 499 nm in DCM solution to 486 nm in the solid), indicated that the observed fluorescence is not due to an excimer excited state. The notable reduction in fluorescence QY in the solid state indicates that, similarly to **4j**, the molecular arrangement in **4b** leads to ACQ, where nonradiative relaxation pathways dominate, suppressing efficient emission.

Additionally, analysis of the molecular geometry of **4j** and **4b** in the dimers, identified in solid, showed that the anthracene plane in **4b** is more bent into a butterfly than in **4j** (ω_P1/P2_ angles are equal to ca. 7° and 2°, respectively), and the P=O substituent in **4j** is coplanar with this plane, in contrast to **4b** (deviations of P and O atoms from the plane are 0.096 and 0.025 Å, respectively, in **4j** versus 1.042 and 0.499 Å in **4b**). Moreover, we observed similar differences between molecules **4b** in monomer (from DFT calculations: ω_P1/P2_ is of ca. 3°, deviations of P and O atoms from the plane are 0.200 and 0.583 Å) and dimer (in crystal). π···π Interactions linking **4j** molecules into dimers with AO = 49.9% result in planarity of anthracene, while the absence of these interactions in **4b** dimers results in a greater anthracene flexibility and susceptibility to interactions of a different type, resulting in anthracene plane bending.

## Conclusions

In summary, a series of novel, multiply substituted 10-(diphenylphosphoryl)-anthracenes with electron-withdrawing 6-CN, 6-CF_3_, 7-F, 6,7-di-F, and 5,6,7-tri-F substituents on one of the flanking anthracene ring and electron-donating substituents on the other one, in addition to the strong electron-withdrawing Ph_2_P=O group on the middle anthracene ring, makes the obtained anthracenes very effective donor–acceptor emitters. To the best of our knowledge, these are the first multiply substituted compounds of this type in the literature. The QYs of the anthracene 6-CN-**4g** in the DCM solution and the anthracene 7-CF_3_-**4f** in the solid state exceeding 95%, are the highest values in this group of compounds and 6 times higher than unsubstituted anthracene (15%, DCM). The analysis of crystal structures of **4j·DCM** and **4b·DCM** showed the crucial role of noncovalent interactions and molecular packing in modulating emission properties in solid, where fluorescence quenching by aggregation took place, without excimer formation. The 10-Ph_2_P = X (X = O, S, Se) group attached to anthracene plays a key role in lowering the LUMO levels of the investigated donor–acceptor aromatic systems and tuning their electron and optical properties. Comparative analysis of the molecular geometries of **6** and **7** with **4g** revealed that heavy atoms (S or Se) in this group caused structural changes (butterfly bending of the anthracene plane and the situation of the P=S and P=Se groups out of this plane) that disturbed π-conjugation, leading to a lower fluorescence QY. The presented results underscore the potential of multiply substituted anthracenes as tunable emitters for use in organic electronics. Further studies are underway to develop other phosphorus-based anthracene-emitting materials.

## Experimental Section

### General Information

Tetrahydrofuran and toluene were dried using a Solvent Purification System (MBraun SPS-800). Dry glassware was obtained by oven-drying and assembly under dry argon. For flash chromatography, a Chromatography System–Büchi Pure C-850 FlashPrep was used. The melting points were obtained with an Electrothermal Model IA9100 apparatus and are uncorrected. Mass spectra were obtained using a SYNAPT G2-Si HDMS (Waters) instrument. NMR spectra were recorded with a Bruker AV 200 MHz, Bruker AVANCE Neo 400 MHz, or Bruker AVANCE III 500 MHz using CDCl_3_, C_6_D_6_, CD_2_Cl_2_, and CD_3_CN, as internal standards. The UV–vis absorption spectra were recorded in 1 cm cuvettes on a Shimadzu UV-2700 spectrophotometer UV-2700. Emission spectra were obtained with a Horiba Jobin Yvon, Fluoromax 4 Plus spectrofluorometer. The fluorescence quantum yields Φ of the obtained compounds were determined in three different solvents (EtOH, cyclohexane, CH_2_Cl_2_) on excitation at their absorption maximum using an integrating sphere (Horiba, Jobin Yvon, Quanta-φ F-3029 Integrating sphere).

### General Procedure for the Synthesis of **4a**–**j**

In a 50 mL Schlenk tube, a solution of diaryl alcohols **1a**–**j** (200 mg, 1 equiv) in anhydrous THF (10 mL) at 0 °C, triethyl amine (1.1 equiv) was added, and the reaction mixture was stirred at room temperature for 1 h. After cooling, the mixture, again to 0 °C, chlorodiphenylphosphine (1.2 equiv) was added and stirred at room temperature for another 3 h. Then, a catalytic amount of TMSOTf (10 mol %) was added, and the crude mixture was stirred overnight in an oil bath at 60 °C. Once the intermediate was consumed (checked with ^31^P NMR), the aqueous solution of HCl (2 mL, 12 N) was added, and the crude mixture was stirred for 1 h. After evaporation of the solvent, the organic layer was dissolved in ethyl acetate and washed with water (5 × 2 mL), then with NaHCO_3_ (5 mL) and extracted with ethyl acetate (10 mL). After drying over MgSO_4_ and evaporation of the solvent, the product was purified by flash chromatography (hexane/EtOAc) to give pure anthracene compounds **4a**–**j**.

#### (7,8,9-Trimethoxyanthra[2,3-*d*][1,3]dioxol-5-yl)diphenyl phosphine oxide **(4a)**

*R*_f_ = 0.43 (EtOAc), *n*-hexane:EtOAc (1:2), green solid, mp 202–204 °C, 81 mg, 32% yield: ^**1**^**H NMR** (400 MHz, CD_2_Cl_2_) δ 8.72 (s, 1H), 8.32 (s, 1H), 7.71–7.65 (m, 4H), 7.55–7.50 (m, 2H), 7.46–7.41 (m, 4H), 7.27(s, 1H), 7.22 (s, 1H), 5.99 (s, 2H), 4.10 (s, 3H), 3.89 (s, 3H), 3.25 (s, 3H); ^**13**^**C{**^**1**^**H} NMR** (101 MHz, CD_2_Cl_2_) δ 152.9 (s), 148.9 (s), 146.7 (d, *J*_PC_ = 2.2 Hz), 146.6 (s), 139.1 (s), 136.2 (d, *J*_PC_ = 102.4 Hz), 134.4 (d, *J*_PC_ = 8.4 Hz), 131.6 (d, *J*_PC_ = 9.5 Hz), 131.4 (d, *J*_PC_ = 2.7 Hz), 131.3 (d, *J*_PC_ = 9.9 Hz), 128.8 (d, *J*_PC_ = 12.1 Hz), 128.3 (d, *J*_PC_ = 11.0 Hz), 126.4 (d, *J*_PC_ = 3.1 Hz), 123.1 (d, *J*_PC_ = 10.9 Hz), 117.2 (d, *J*_PC_ = 100.4 Hz), 103.5 (s), 102.5 (d, *J*_PC_ = 7.0 Hz), 102.0 (d, *J*_PC_ = 7.9 Hz), 101.5 (s), 61.5 (s), 60.8 (s), 55.3 (s); ^**31**^**P NMR** (162 MHz, CD_2_Cl_2_) δ 30.54; **HRMS** (TOF MS ES+): calcd. for C_30_H_26_O_6_P [M + H^+^] 513.1467, found 513.1462.

#### (7,9-Dimethoxyanthra[2,3-*d*][1,3]dioxol-5-yl)diphenyl phosphine oxide (**4b**)

*R*_f_ = 0.50 (EtOAc), *n*-hexane:EtOAc (1:2), yellow crystals, mp 232–234 °C, 78 mg, 30% yield: ^**1**^**H NMR** (400 MHz, CD_2_Cl_2_) δ 8.84 (s, 1H), 8.46 (s, 1H), 7.72–7.66 (m, 4H), 7.53–7.49 (m, 2H), 7.45–7.40 (m, 4H), 7.25 (d, *J* = 1.7 Hz, 1H), 6.80 (s, 1H), 6.31 6.31 (d, *J* = 2.0 Hz, 1H), 5.99 (s, 2H), 3.99 (s, 3H), 3.18 (s, 3H); ^**13**^**C{**^**1**^**H} NMR** (101 MHz, CD_2_Cl_2_) δ 158.1 (s), 156.8 (d, *J*_PC_ = 2.2 Hz), 149.7 (s), 146.8 (s),136.7 (d, *J*_PC_ = 102.5 Hz), 135.9 (d, *J*_PC_ = 8.4 Hz), 135.4 (d, *J*_PC_ = 9.5 Hz), 131.7 (d, *J*_PC_ = 2.3 Hz), 131.6 (d, *J*_PC_ = 10.3 Hz), 129.1 (d, *J*_PC_ = 11.9 Hz), 128.1 (d, *J*_PC_ = 11.1 Hz), 127.4 (d, *J*_PC_ = 3.0 Hz), 120.8 (d, *J*_PC_ = 11.3 Hz), 117.2 (d, *J*_PC_ = 101.2 Hz), 104.3 (s), 102.9 (d, *J*_PC_ = 6.7 Hz), 101.9 (s), 98.1 (d, *J*_PC_ = 8.3 Hz), 96.6 (s), 56.1 (s), 55.3 (s). ^**31**^**P NMR** (162 MHz, CD_2_Cl_2_) δ 30.56; **HRMS** (TOF MS ES+): calcd. for C_29_H_24_O_5_P [M + H^+^] 483.1361, found 483.1358.

#### (7-Methoxyanthra[2,3-*d*][1,3]dioxol-5-yl)diphenyl phosphine oxide (**4c**)

*R*_f_ = 0.53 (EtOAc), *n*-hexane:EtOAc (1:2), green solid, mp 227–229 °C, 60 mg, 24% yield: ^**1**^**H NMR** (400 MHz, CD_2_Cl_2_) δ 8.42 (d, *J* = 12.0 Hz, 2H), 7.83 (dd, *J* = 9.1, 1.9 Hz, 1H), 7.72–7.67 (m, 4H), 7.55–7.50 (m, 2H), 7.46–7.41 (m, 4H), 7.31 (d, *J* = 2.4 Hz, 1H), 7.23 (d, *J* = 1.7 Hz, 1H), 7.00 (dd, *J* = 9.1, 2.4 Hz, 1H), 6.00 (s, 2H), 3.22 (s, 3H); ^**13**^**C{**^**1**^**H} NMR** (101 MHz, CD_2_Cl_2_) δ 157.5 (s), 149.6 (s), 147.0 (s), 136.6 (d, *J*_PC_ = 102.5 Hz), 135.6 (d, *J*_PC_ = 8.5 Hz), 135 (d, *J*_PC_ = 8.8 Hz), 133 (d, *J*_PC_ = 3.1 Hz), 131.8 (d, *J*_PC_ = 2.8 Hz), 131.7 (d, *J*_PC_ = 9.9 Hz), 130.6 (s), 129.2 (d, *J*_PC_ = 12.2 Hz), 128.6 (d, *J*_PC_ = 11.3 Hz), 127.1 (d, *J*_PC_ = 10.8 Hz), 119.39 (s), 117.7 (d, *J*_PC_ = 100.1 Hz), 105 (d, *J*_PC_ = 7.8 Hz), 103.5 (s), 103 (d, *J*_PC_ = 6.8 Hz), 102 (s), 55.24 (s); ^**31**^**P NMR** (162 MHz, CD_2_Cl_2_) δ 30.28; **HRMS** (TOF MS ES+): calcd. for C_28_H_21_O_4_P [M + H^+^] 453.1256, found 453.1256.

#### (2,3,4-Trimethoxyanthr-9-yl)diphenyl Phosphine Oxide (**4d**)

*R*_f_ = 0.41 (EtOAc), *n*-hexane:EtOAc (1:2), yellow solid, mp 150–151 °C, 96 mg, 37% yield: ^**1**^**H NMR** (400 MHz, CD_2_Cl_2_) δ 8.98 (d, *J* = 1.7 Hz, 1H), 8.62 (dt, *J* = 9.1, 1.0 Hz, 1H), 8.07 (dt, *J* = 8.4, 1.8 Hz, 1H), 7.73–7.67 (m, 4H), 7.61(s, 1H), 7.54–7.49(m, 4H), 7.46–7.40 (m, 2H), 7.39–7.36 (m, 1H), 7.27–7.23 (m, 1H), 4.15 (s, 3H), 3.94 (s, 3H), 3.39 (s, 3H); ^**13**^**C{**^**1**^**H} NMR** (101 MHz, CD_2_Cl_2_) δ 154 (s), 146.9 (s), 139.7 (s), 136.6 (d, *J*_PC_ = 102.5 Hz), 135.2 (d, *J*_PC_ = 8.5 Hz), 133.4 (d, *J*_PC_ = 9.0 Hz), 131.5 (d, *J*_PC_ = 2.9 Hz), 131.4 (d, *J*_PC_ = 9.8 Hz), 130.2 (d, *J*_PC_ = 10.9 Hz), 129.7 (s), 128.9 (d, *J*_PC_ = 11.9 Hz), 128.3 (d, *J*_PC_ = 3.2 Hz), 126.9 (d, *J*_PC_ = 7.0 Hz), 126.3 (s), 124.6 (d, *J*_PC_ = 11.1 Hz), 124.3 (s), 118.7 (d, *J*_PC_ = 100.1 Hz), 101.9 (d, *J*_PC_ = 7.5 Hz), 61.7 (s), 61.1 (s), 55.5 (s); ^**31**^**P NMR** (162 MHz, CD_2_Cl_2_) δ 29.57; **HRMS** (TOF MS ES+): calcd. for C_29_H_25_O_4_P [M + H^+^] 469.1568, found 469.1569.

#### (6-Fluoro-2,3,4-trimethoxyanthr-9-yl)diphenyl Phosphine Oxide (**4e**)

*R*_f_ = 0.46 (EtOAc), *n*-hexane:EtOAc (1:2), yellow solid, mp 154–156 °C, 78 mg, 32% yield: ^**1**^**H NMR** (400 MHz, CD_2_Cl_2_) δ 8.97–8.91 (m, 2H), 7.71–7.66 (m, 5H), 7.56–7.51 (m, 2H), 7.47–7.42 (m, 4H), 7.36 (s, 1H), 7.15–7.10 (m, 1H), 4.14 (s, 3H), 3.93 (s, 3H), 3.31 (s, 3H); ^**13**^**C{**^**1**^**H} NMR** (101 MHz, CD_2_Cl_2_) δ 159.4 (d, *J*_CF_ = 247.4 Hz), 154.1 (s), 146.8 (s), 140.4 (s), 136.4 (d, *J*_PC_ = 102.8 Hz), 133.1 (d, *J*_PC_ = 8.3 Hz), 132.7 (d, *J*_PC_ = 8.7 Hz), 132 (d, *J*_PC_ = 2.8 Hz), 131.6 (d, *J*_PC_ = 10 Hz), 131.2 (dd, *J*_PC_ = 10.1, *J*_CF_ = 19.7 Hz), 130.2 (dd, *J*_PC_ = 7.5, *J*_CF_ = 15.2 Hz), 129.2 (d, *J*_PC_ = 12.1 Hz), 127.5 (dd, *J* = 6.7, 3.2 Hz), 125.6 (d, *J*_PC_ = 11 Hz), 119.9 (d, *J*_PC_ = 99.7 Hz), 117.7 (d, *J*_CF_ = 26.1 Hz), 111.4 (d, *J*_CF_ = 20.0 Hz), 102.3 (d, *J*_PC_ = 7.8 Hz), 61.9 (s), 61.3 (s), 55.8 (s); ^**31**^**P NMR** (162 MHz, CD_2_Cl_2_) δ 29.87; ^**19**^**F NMR** (376 MHz, CD_2_Cl_2_) δ −117.04; **HRMS** (TOF MS ES+): calcd. for C_29_H_25_O_4_PF [M + H^+^] 487.1472, found 487.1474.

#### (2,3,4-Trimethoxy-7-(trifluoromethyl)anthr-9-yl)diphenyl Phosphine Oxide (**4f**)

*R*_f_ = 0.56 (EtOAc), *n*-hexane:EtOAc (1:2), green solid, mp 134–137 °C, 123 mg, 49% yield: ^**1**^**H NMR** (400 MHz, CD_2_Cl_2_) δ 9.22 (s, 1H), 9.06 (s, 1H), 8.20 (d, *J* = 8.8 Hz, 1H), 7.80–7.68 (m, 4H), 7.63(s, 1H), 7.80–7.68 (m, 4H), 4.17 (s, 3H), 3.97 (s, 3H), 3.44 (s, 3H); ^**13**^**C{**^**1**^**H} NMR** (101 MHz, CD_2_Cl_2_) δ: 154.8 (s), 147.1 (s), 140.7 (s), 136.2 (d, *J*_PC_ = 103.1 Hz), 134.1 (d, *J*_PC_ = 8.5 Hz), 133.8 (d, *J*_PC_ = 8.1 Hz), 132.1 (d, *J*_PC_ = 3.1 Hz), 131.6 (d, *J*_PC_ = 9.9 Hz), 131.3 (s), 130.9 (d, *J*_PC_ = 10.5 Hz), 129.3 (d, *J*_PC_ = 12.2 Hz), 128.5 (d, *J*_PC_ = 3.1 Hz), 127.5 (q, *J*_CF_ = 31.6 Hz), 126.3 (d, *J*_PC_ = 10.5 Hz), 125.3 (dq, *J*_PC_ = 5.3 Hz, *J*_PC_ = 5.3 Hz), 124.7 (q, *J*_CF_ = 271.8 Hz), 121.35(d, *J*_PC_ = 98.3 Hz), 119.5 (q, *J*_CF_ = 3.0 Hz), 102.4 (d, *J*_PC_ = 7.9 Hz), 62 (s), 61.3 (s), 55.9 (s); ^**31**^**P NMR** (162 MHz, CD_2_Cl_2_) δ 29.28; ^**19**^**F NMR** (376 MHz, CD_2_Cl_2_) δ −63.00; **HRMS** (TOF MS ES+): calcd. for C_30_H_25_O_4_PF_3_ [M + H^+^] 537.1442, found 537.1443.

#### 9-(Diphenylphosphoryl)-5,6,7-trimethoxyanthra-2-carbonitrile (**4g**)

*R*_f_ = 0.54 (EtOAc); *n*-hexane:EtOAc (1:2), yellow solid, mp 176–178 °C; 141 mg, 55% yield: ^**1**^**H NMR** (400 MHz, CD_2_Cl_2_) δ 9.61 (s, 1H), 9.03 (s, 1H), 8.14 (dd, *J* = 8.8, 1.8 Hz, 1H), 7.75–7.70 (m, 4H), 7.59–7.54 (m, 2H), 7.50–7.45 (m, 5H), 7.30 (s, 1H), 4.15 (s, 3H), 3.95 (s, 3H), 3.33 (s, 3H); ^**13**^**C{**^**1**^**H} NMR** (101 MHz, CD_2_Cl_2_) δ 155 (s), 147.1 (s), 140.9 (s), 135.8 (d, *J*_PC_ = 103.4 Hz), 134.3(s), 134.2 (d, *J*_PC_ = 5.6 Hz), 133.7 (d, *J*_PC_ = 8.7 Hz), 132.3 (d, *J*_PC_ = 3.2 Hz), 131.6 (d, *J*_PC_ = 10.0 Hz), 131.2 (s), 130.8 (d, *J*_PC_ = 10.2 Hz), 129.3 (d, *J*_PC_ = 12.2 Hz), 128.6 (d, *J*_PC_ = 3.1 Hz), 126.6 (d, *J*_PC_ = 10.6 Hz), 124.1 (s), 121.1 (d, *J*_PC_ = 97.2 Hz), 119.6 (s), 109.9 (s), 102.4 (d, *J*_PC_ = 7.9 Hz), 62 (s), 61.3 (s), 55.9 (s); ^**31**^**P NMR** (162 MHz, CD_2_Cl_2_) δ 29.88; **HRMS** (TOF MS ES+): calcd. for C_30_H_25_O_4_PN [M+H^+^] 494.1531, found 494.1521.

#### (6-Bromo-2,3,4-trimethoxyanthr-9-yl)diphenyl Phosphine Oxide (**4h**)

*R*_f_ = 0.45 (EtOAc), *n*-hexane:EtOAc (1:2), orange oil, 112 mg, 45% yield: ^**1**^**H NMR** (400 MHz, CD_2_Cl_2_) δ 8.87–8.86 (m, 1H), 8.67 (d, *J* = 9.6 Hz, 1H), 8.22 (dd, *J* = 2.1, *J* = 2.1 Hz, 1H), 7.69–7.64 (m, 4H), 7.55–7.51 (m, 2H), 7.46–7.41 (m, 5H), 7.30 (dd, *J* = 9.6, 2.2 Hz, 1H), 4.12 (s, 3H), 3.92 (s, 3H), 3.33 (s, 3H); ^**13**^**C{**^**1**^**H} NMR** (101 MHz, CD_2_Cl_2_) δ 154.2 (s), 146.8 (s), 140.1 (s), 36.1 (d, *J*_PC_ = 102.8 Hz),133.6 (d, *J*_PC_ = 8.6 Hz), 133.2 (d, *J*_PC_ = 8.7 Hz), 131.7 (d, *J*_PC_ = 2.7 Hz), 131.4 (d, *J*_PC_ = 10.1 Hz), 131.2 (s), 131.1 (s), 129.4 (s), 128.9 (d, *J*_PC_ = 12.1 Hz), 128.8 (d, *J*_PC_ = 6.5 Hz),127.2 (d, *J*_PC_ = 3.1 Hz), 125.3 (d, *J*_PC_ = 10.6 Hz), 119.5 (d, *J*_PC_ = 99.4 Hz), 118.2 (s), 102 (d, *J*_PC_ = 7.5 Hz), 61.7 (s), 61.1 (s), 55.5 (s); ^**31**^**P NMR** (162 MHz, CD_2_Cl_2_) δ 29.60; **HRMS** (TOF MS ES+): calcd. for C_29_H_24_O_4_PBr [M + H^+^] 547.0672, found 547.0670.

#### (1,2,3-Trifluoro-5,6,7-trimethoxyanthr-9-yl)diphenyl Phosphine Oxide (**4i**)

*R*_f_ = 0.40 (EtOAc), *n*-hexane:EtOAc (1:2), yellow solid, mp 186–188 °C; 151 mg, 60% yield: ^**1**^**H NMR** (400 MHz, C_6_D_6_) δ 8.70 (s, 1H), 8.11 (s, 1H), 7.71–7.66 (m, 4H), 6.96–6.89 (m, 7H), 3.92 (s, 3H), 3.71 (s, 3H), 3.25 (s, 3H); ^**13**^**C{**^**1**^**H} NMR** (101 MHz, C_6_D_6_) δ 155.1 (s), 149.1 (dd, *J*_CF_ = 251.2, 14.1 Hz), 146.9 (s), 146 (dd, *J*_CF_ = 251.03, 14.3 Hz), 140.4 (dd, *J*_CF_ = 251.8, 13.9 Hz), 141.1 (s), 138.2 (dd, *J*_PC_ = 105.9, 4.3 Hz), 135.6 (d, *J*_PC_ = 6.8 Hz), 130.9 (dd, *J*_PC_ = 9.5, 2.4 Hz), 130.7 (d, *J*_PC_ = 2.8 Hz), 129.3 (s), 128.4 (d, *J*_PC_ = 12.3 Hz), 127.1 (d, *J*_PC_ = 4.2 Hz), 126.1 (d, *J*_PC_ = 10.2 Hz), 124.1 (dd, *J*_PC_ = 12.1, 6.1 Hz), 119.2 (d, *J*_PC_ = 99.4 Hz), 109.4 (dd, *J*_CF_ = 16.6, 4.3 Hz), 103.5(d, *J*_PC_ = 8.0 Hz), 61.3 (s), 60.9 (s), 55.6 (s); ^**31**^**P NMR** (162 MHz, C_6_D_6_) δ 26.95 (d, *J*_PF_ = 12.2 Hz); ^**19**^**F NMR** (376 MHz, C_6_D_6_) δ −157.88 (ddd, *J*_FF_ = 19.6 Hz, *J*_FF_ = 17.5 Hz, *J*_HF_ = 7.5 Hz), −136.48 (ddd, *J*_FF_ = 19.6 Hz, *J*_FF_ = 5.5 Hz, *J*_HF_ = 10.0 Hz), −116.51 (ddd, *J*_FF_ = 17.5, *J*_PF_ = 12.2 Hz, *J*_FF_ = 5.5 Hz); ^**19**^**F{**^**1**^**H} NMR** (376 MHz, C_6_D_6_) δ −157.88 (dd, *J*_FF_ = 19.6 Hz, *J*_FF_ = 17.5 Hz), −136.48 (dd, *J*_FF_ = 19.6 Hz, *J*_FF_ = 5.5 Hz), −116.51 (ddd, *J*_FF_ = 17.5 Hz, *J*_PF_ = 12.2 Hz, *J*_FF_ = 5.5 Hz); **HRMS** (TOF MS ES+): calcd. for C_29_H_22_O_4_PF_3_ [M + H^+^] 523.1288, found 523.1286.

#### (6,7-Difluoro-2,3,4-trimethoxyanthr-9-yl)diphenyl Phosphine Oxide (**4j**)

*R*_f_ = 0.56 (EtOAc), *n*-hexane:EtOAc (1:2), green crystals, mp 176–178 °C; 112 mg, 44% yield: ^**1**^**H NMR** (400 MHz, C_6_D_6_) δ 9.47 (dd, *J* = 15.2, 8.3 Hz, 1H), 8.80 (s, 1H), 7.93 (s, 1H), 7.78 (dd, *J* = 12.1, 7.6 Hz, 4H), 6.99–6.90 (m, 7H), 3.87 (s, 3H), 3.71 (s, 3H), 3.12 (s, 3H); ^**13**^**C{**^**1**^**H} NMR** (101 MHz, C_6_D_6_) δ 154.6 (s), 151.3 (dd, *J*_CF_ = 251.7, 17.1 Hz), 148.8 (dd, *J*_CF_ = 250.8, 16.9 Hz), 147.3 (s), 140.5 (s), 136.8 (d, *J*_PC_ = 102.5 Hz), 133.5 (d, *J*_PC_ = 8.5 Hz), 133.4 (s), 132.1 (s), 131.8 (d, *J*_PC_ = 9.8 Hz), 131.5 (d, *J*_PC_ = 2.8 Hz), 128.9 (d, *J*_PC_ = 12.0 Hz), 125.1 (d, *J*_PC_ = 9.8 Hz), 120.4 (d, *J*_PC_ = 2.8 Hz), 119.9 (dd, *J*_PC_ = 102.6, 6.8 Hz), 114.2 (d, *J*_CF_ = 16.1 Hz), 113.8 (dd, *J*_CF_ = 21.6, 6.1 Hz), 102.6 (d, *J*_PC_ = 7.5 Hz), 61.2 (s), 60.8 (s), 55.4 (s); ^**31**^**P NMR** (162 MHz, C_6_D_6_) δ 28.83; ^**19**^**F NMR** (376 MHz, C_6_D_6_) δ −137.45 (ddd, *J*_FF_ = 20.0, *J*_HF_ = 15.7, 9.6 Hz), −131.50 (ddd, *J*_FF_ = 21.0, *J*_HF_ = 15.1, 8.7 Hz); ^**19**^**F{**^**1**^**H} NMR** (376 MHz, C_6_D_6_) δ −137.45 (d, *J*_FF_ = 21.1 Hz), −131.50 (d, *J*_FF_ = 20.9 Hz); **HRMS** (TOF MS ES+): calcd. for C_29_H_23_O_4_PF_2_ [M+H^+^] 505.1377, found 505.1380.

### Procedure for the Synthesis of **5**

In a Schlenk tube dried and filled with argon, **4g** (50 mg, 0.101 mmol) was dissolved in dry toluene (10 mL). Then, trichlorosilane (138 mg, 1.01 mmol) was added dropwise at room temperature and the mixture was stirred in an oil bath at 90 °C for 2 h. The volatiles were removed under reduced pressure and the crude product was dissolved in DCM (10 mL) and filtered through the aluminum oxide layer. After evaporation of the solvent, the crude compound containing 27% silane was obtained as a yellow solid and was further used as such in the syntheses of **6**–**9**.

#### 9-(Diphenylphosphanyl)-5,6,7-trimethoxyanthracene-2- carbonitrile (**5**)

Yellow solid, mp 193–195 °C; ^**1**^**H NMR** (400 MHz, C_6_D_6_) δ 9.83 (d, *J* = 7.1 Hz, 1H), 8.89 (s, 1H), 7.70 (d, *J* = 3.5 Hz, 1H), 7.47 (d, *J* = 8.6 Hz, 1H), 7.42–7.38 (m, 4H), 6.95–6.93 (m, 7H), 3.83 (s, 3H), 3.67 (s, 3H), 3.10 (s, 3H); ^**13**^**C{**^**1**^**H} NMR** (101 MHz, C_6_D_6_) δ 154.9 (s), 147.7 (s), 141.6 (s), 136.4 (d, *J*_PC_ = 22.1 Hz), 136.2 (d, *J*_PC_ = 14.1 Hz), 135.2 (d, *J*_PC_ = 5.1 Hz), 134.6 (d, *J*_PC_ = 36.09 Hz), 131.9 (d, *J*_PC_ = 18.4 Hz), 131.7 (s), 131.1 (s), 131.1 (d, *J* = 5.2 Hz) 128.9 (d, *J*_PC_ = 5.6 Hz), 127 (s),126.9 (s), 126.2 (s), 124.1 (s), 119.5 (s), 110.6 (s), 103.6 (d, *J*_PC_ = 15.3 Hz), 61.2 (s), 60.7 (s), 55.4 (s); ^**31**^**P NMR** (162 MHz, CD_2_Cl_2_) δ −24.21.

### Procedure for the Synthesis of **6** and **7**

Compound **5** (50 mg, 0.105 mmol) was treated with sublimed sulfur (15 mg, 0.467 mmol) in toluene (7 mL) at reflux under an argon atmosphere. Once compound **5** was consumed (checked with TLC), the solvent was evaporated, and the crude mixture was separated using silica-gel flash chromatography with hexane/EtOAc as eluents (2:1 v/v) to afford **6** (40 mg) as an orange solid (78% from **4g**). When elemental selenium (33 mg, 0.418 mmol) was used instead of sulfur, **7** (39 mg) was obtained as an orange solid in 70% yield from **4g**.

#### 9-(Diphenylphosphorothioyl)-5,6,7-trimethoxyanthracene-2-carbonitrile (**6**)

*R*_f_ = 0.86 (EtOAc), *n*-hexane:EtOAc (1:1), orange solid, mp 156–158 °C: 40 mg, 78% yield; ^**1**^**H NMR** (400 MHz, CD_2_Cl_2_) δ 8.94 (s, 1H), 8.20 (s, 1H), 8.11 (dd, *J* = 8.7, 2.0 Hz, 1H), 7.85–7.80 (m, 4H), 7.48–7.44 (m, 1H), 7.41–7.36 (m, 6H), 7.08 (s, 1H), 4.14 (s, 3H), 3.92 (s, 3H), 3.23 (s, 3H); ^**13**^**C{**^**1**^**H} NMR** (101 MHz, CD_2_Cl_2_) δ 154.7 (s), 147.2 (s), 141.2 (s), 136.9 (d, *J*_*PC*_ = 82.3 Hz), 133.5 (d, *J*_PC_ = 9.7 Hz), 132.5 (d, *J*_PC_ = 7.9 Hz), 131.5 (d, *J*_PC_ = 3.0 Hz), 131.3 (d, *J*_PC_ = 10.1 Hz), 130.9 (d, *J*_PC_ = 10.2 Hz), 129.2 (d, *J*_PC_ = 12.4 Hz), 127.9 (d, *J*_PC_ = 2.8 Hz), 127.4 (d, *J*_PC_ = 81.2 Hz), 127.1 (s), 126.9 (s), 123.9 (s), 122 (d, *J*_PC_ = 87.4 Hz), 119.1 (s), 109.1 (s), 102.9 (d, *J*_PC_ = 10.8 Hz), 62.1 (s), 61.4 (s), 55.9 (s); ^**31**^**P NMR** (162 MHz, CD_2_Cl_2_) δ 33.92; **HRMS** (TOF MS ES+): calcd. for C_30_H_25_O_3_PNS [M+H^+^] 510.1295, found 510.1296.

#### 9-(Diphenylphosphoroselenoyl)-5,6,7-trimethoxyanthracene-2-carbonitrile (**7**)

*R*_f_ = 0.83 (EtOAc); *n*-hexane:EtOAc (1:1), orange solid, mp 164–166 °C; 39 mg, 70% yield: ^**1**^**H NMR** (400 MHz, C_6_D_6_) δ 8.79 (s, 1H), 8.45 (s, 1H), 7.95- 7.89 (m, 4H), 7.51 (s, 1H), 7.36 (dd, *J* = 8.6, 2.0 Hz, 1H), 6.85- 6.80 (m, 6H), 6.78 (d, *J* = 1.4 Hz, 1H), 3.87 (s, 3H), 3.68 (s, 3H), 3.01 (s, 3H); ^**13**^**C{**^**1**^**H} NMR** (101 MHz, C_6_D_6_) δ 154 (s), 146.8 (s), 140.9 (s), 135.2 (d, *J*_PC_ = 73.3 Hz), 133.1 (d, *J*_PC_ = 9.5 Hz), 132.1 (d, *J*_PC_ = 7.6 Hz), 131.6 (d, *J*_PC_ = 10.8 Hz), 130.7 (d, *J*_PC_ = 3.1 Hz), 130.5 (d, *J*_PC_ = 10.3 Hz), 130.3 (s), 128.6 (d, *J*_PC_ = 77.1 Hz), 128.5 (d, *J*_PC_ = 12.6 Hz), 126.8 (d, *J*_PC_ = 3.5 Hz), 126.6 (d, *J*_PC_ = 10.5 Hz), 123.4 (s), 121.2 (d, *J*_PC_ = 78.8 Hz), 118.4 (s), 109.1 (s), 103.4 (d, *J*_PC_ = 11.4 Hz), 61 (s), 60.5 (s), 55.1 (s); ^**31**^**P NMR** (162 MHz, CD_2_Cl_2_) δ 25.25; (d,^1^*J*_PSe_ = 754 Hz); ^**77**^**Se NMR** (76 MHz, CD_2_Cl_2_) δ −289.76 (d,^1^*J*_PSe_ = 738.9 Hz); ^**77**^**Se NMR** (76 MHz, C_6_D_6_) δ −291.02 (d,^1^*J*_PSe_ = 754.5 Hz); **HRMS** (TOF MS ES+): calcd. for C_30_H_25_O_3_PNSe [M+H^+^] 557.0660, found 557.0686.

### Procedure for the Synthesis of the Gold(I) Complex **8**

Compound **5** (20 mg) was dissolved in CH_2_Cl_2_ (5 mL) and treated with [Au(tht)Cl] (12 mg, 0.042 mmol) at room temperature under an argon atmosphere. The resulting mixture was stirred for 2 h. After 2 h, the volatiles were removed under vacuum. The crude mixture was redissolved in CH_2_Cl_2_ and filtered through the aluminum oxide layer, and the product was obtained as a yellow solid (25 mg, 90% from **4g**).

**(8):** yellow solid, 25 mg, 90% yield: ^**1**^**H NMR** (400 MHz, CD_2_Cl_2_) δ 9.02 (s, 1H), 8.15 (dd, *J* = 8.7, 1.7 Hz, 1H), 8.06 (s, 1H), 7.71–7.65 (m, 4H), 7.60 (dd, *J* = 7.4, 2.0 Hz, 1H), 7.58–7.55 (m, 2H), 7.53–7.48 (m, 4H), 7.44 (dd, *J* = 8.6, 1.4 Hz, 1H), 4.17 (s, 3H), 3.97 (s, 3H), 3.62 (s, 3H); ^**13**^**C{**^**1**^**H} NMR** (101 MHz, CD_2_Cl_2_) δ 155.8 (s), 147.5 (s), 141.3 (s), 134.3 (d, *J*_PC_ = 10.6 Hz), 134.1 (d, *J*_PC_ = 14.2 Hz), 132.5 (d, *J*_PC_ = 2.7 Hz), 132.3 (d, *J*_PC_ = 10.5 Hz), 131.8 (s), 130.9 (d, *J*_PC_ = 8.3 Hz), 130.4 (d, *J*_PC_ = 60.3 Hz), 130.2 (d, *J*_PC_ = 12.1 Hz), 129.4 (d, *J*_PC_ = 12.2 Hz), 128.8 (d, *J*_PC_ = 2.9 Hz), 126.9 (d, *J*_PC_ = 8.9 Hz), 124.1 (s), 118.9 (s), 116.2 (d, *J*_PC_ = 58.4 Hz), 109.9 (s), 101.1 (d, *J*_PC_ = 18.6 Hz), 62.1 (s), 61.5 (s), 56.4 (s); ^**31**^**P NMR** (162 MHz, CD_2_Cl_2_) δ 23.39; **HRMS** (TOF MS ES+): calcd. for C_30_H_24_O_3_PNAuCl [M^+^] 709.0875, found 709.0888.

### Procedure for the Synthesis of **9**

Compound **5** (50 mg, 0.105 mmol) was treated with iodomethane (60 mg, 0.467 mmol) in toluene (7 mL) at reflux. Once compound **5** was consumed (checked with TLC), the solvent was evaporated, and the crude mixture was separated using silica-gel flash chromatography with hexane/EtOAc as eluents (2:1 v/v) to afford **9** (22 mg) as an orange solid (72% from **4g**).

#### 5,6,7-Trimethoxyanthracene-2-carbonitrile (**9**)

*R*_f_ = 0.87 (EtOAc); *n*-hexane:EtOAc (1:1), orange solid; mp 127–129 °C: 22 mg, 72% yield: ^**1**^**H NMR** (400 MHz, C_6_D_6_) δ 8.60 (s, 1H), 7.87 (s, 1H), 7.73 (s, 1H), 7.45 (d, *J* = 8.7 Hz, 1H), 6.99 (dd, *J* = 8.6, 1.6 Hz, 1H), 6.66 (s, 1H), 3.86 (s, 3H), 3.79 (s, 3H), 3.43 (s, 3H); ^**13**^**C{**^**1**^**H} NMR** (101 MHz, C_6_D_6_) δ 154.6, 147.6, 142.3, 134.7, 130.6, 130.4, 130.2, 129.9, 126.7, 125.6, 124.1, 121.2, 119.6, 109.4, 101.2, 61.1, 60.9, 55.3; **HRMS** (TOF MS ES+): calcd. for C_18_H_16_O_3_N [M+H^+^] 294.1116, found 294.1130.

### Procedure for the Synthesis of **10**

In a Schlenk tube, dried and filled with argon, **4h** (40 mg, 0.074 mmol), 2-thienylboronic acid (11 mg, 0.080 mmol), Pd(PPh_3_)_4_ (6 mg, 0.08 equiv) and K_2_CO_3_ (28 mg, 2.5 equiv) were dissolved in a toluene:MeOH (3:1 v/v) solution (4 mL). The reaction mixture was stirred in an oil bath at 80 °C for 36 h. Then, the solvent was removed in a vacuum, and the resulting solid was dissolved in EtOAc (10 mL) and washed with water (7 × 2 mL). After drying with MgSO_4_, the solvent was removed, and the crude product was purified using flash silica chromatography with hexane: EtOAc (1:1 v/v) used as an eluent. Finally, product **10** was obtained as a green solid (37 mg) in a 94% yield.

#### (2,3,4-Trimethoxy-6-(thien-2-yl)anthr-9-yl)diphenyl Phosphine Oxide (**10**)

*R*_f_ = 0.42 (EtOAc); *n*-hexane:EtOAc (1:2), yellow oil; 37 mg 94% yield: ^**1**^**H NMR** (400 MHz, CD_2_Cl_2_) δ 8.96 (s, 1H), 8.64 (d, *J* = 9.3 Hz, 1H), 8.26 (s, 1H), 7.73–7.67 (m, 4H), 7.57 (s, 1H), 7.55–7.50 (m, 4H), 7.46–7.43 (m, 4H), 7.42 (dd, *J* = 3.0, 1.2 Hz, 1H), 7.35 (dd, *J* = 5.1, 1.1 Hz, 1H), 7.13 (dd, *J* = 5.1, 3.6 Hz, 1H), 4.15 (s, 3H), 3.93 (s, 3H), 3.38 (s, 3H); ^**13**^**C{**^**1**^**H} NMR** (101 MHz, C_6_D_6_) δ 154.7 (s), 147.5 (s), 144.1 (s), 140.7 (s), 137.6 (d, *J*_PC_ = 102.0 Hz), 134.7 (dd, *J*_PC_ = 8.7, 6.0 Hz), 134. Three (d, *J*_PC_ = 102.56 Hz), 132.4 (d, *J*_PC_ = 9.6 Hz), 131.9 (d, *J*_PC_ = 9.8 Hz), 131.5 (d, *J*_PC_ = 2.8 Hz), 131.2 (d, *J*_PC_ = 2.8 Hz), 130.8 (d, *J*_PC_ = 10.7 Hz), 130.4 (s),128.8 (d, *J* = 12.0 Hz), 128.6 (d, *J* = 4.9 Hz), 128.5 (s), 125.9 (d, *J*_PC_ = 10.9 Hz), 125.4 (s), 125.3 (d, *J* = 2.8 Hz), 124.1 (s), 120 (d, *J*_PC_ = 98.5 Hz), 103 (d, *J*_PC_ = 6.8 Hz), 61.3 (s), 60.8 (s), 55.5 (s); ^**31**^**P NMR** (162 MHz, CD_2_Cl_2_) δ 29.57; **HRMS** (TOF MS ES+): calcd. for C_33_H_28_O_4_PS [M+H^+^] 551.1444, found 551.1446.

## Data Availability

The data underlying this study are available in the published article and its Supporting Information (SI).

## Supporting Information Available

General information, detailed experimental procedures, characterization data for compounds, NMR spectra, photophysical properties, crystal structure data, computational studies, and CIE 1931 color space coordinates data; and additional references 48–52 (PDF)
