# Supplementary material for: High-Efficiency Light Emitters: 10-(Diphenylphosphoryl)-anthracenes from One-Pot Synthesis Including C–O–P to C–P(=O) Rearrangement
Source: J Org Chem. 2025 Mar 25;90(13):4580–90. doi: 10.1021/acs.joc.4c03139 (PMC11976837; doi:10.1021/acs.joc.4c03139)
Supplement: Supplementary file 1 — jo4c03139_si_001.pdf [file jo4c03139_si_001.pdf]

High-Efficiency Light Emitters: 10-(Diphenylphosphoryl)-anthracenes from One-Pot Synthesis Including C-O-P to C-P(=O) Rearrangement

Vivek Vivek,[a,b] Marek Koprowski,[a] Ewa Różycka-Sokołowska,*[c] Marika Turek,[c] Bogdan Dudziński,[a] Krzysztof Owsianik,[a] Łucja Knopik[a,b] and Piotr Bałczewski*[a,c]

[a] Division of Organic Chemistry, Centre of Molecular and Macromolecular Studies, Polish Academy of Sciences, Sienkiewicza 112, Łódź, 90-363, Poland;
E-mail: piotr.balczewski@cbmm.lodz.pl

[b] The Bio-Med-Chem Doctoral School of the University of Łódź and Łódź Institutes of the Polish Academy of Sciences, University of Łódź, Matejki 21/23, Łódź, 90-237, Poland.

[c] Institute of Chemistry, Faculty of Science and Technology, Jan Długosz University in Częstochowa, Armii Krajowej 13/15, Częstochowa, 42-201, Poland;
E-mail: e.sokolowska@ujd.edu.pl

Table of contents

1. **General information** **S1**
2. **Synthetic procedures** **S2**
3. **NMR spectra** **S13**
4. **Photophysical properties** **S51**
5. **Crystal structure data** **S54**
6. **Computational studies** **S60**
7. **CIE 1931 color space coordinates data** **S80**
8. **Reaction mechanisms** **S86**
9. **References** **S88**

1. General information:

Tetrahydrofuran and toluene were dried using Solvent Purification System (MBraun SPS-800). Dry glassware was obtained by oven-drying and assembly under dry argon. For flash chromatography, Chromatography System – Büchi Pure C-850 FlashPrep was used. The melting points were obtained with an Electrothermal Model IA9100 apparatus and are uncorrected. Mass spectra were obtained by using a SYNAPT G2-Si HDMS (Waters) instrument. NMR spectra were recorded with a Bruker AV 200 MHz, Bruker AVANCE Neo 400 MHz or Bruker AVANCE III 500 MHz using $CDCl_3$, C_6D_6, CD_2Cl_2, CD_3CN, as internal standards. The UV-Vis absorption spectra were recorded in 1 cm cuvettes on a Shimadzu spectrophotometer UV-2700. Emission spectra were obtained with the Horiba Jobin Yvon, Fluoromax 4 Plus spectrofluorometer. The fluorescence quantum yields Φ of the obtained compounds were determined in three different solvents (EtOH, cyclohexane, CH_2Cl_2) on excitation at their absorption maximum using an integrating sphere (Horiba, Jobin Yvon, Quanta-φ F-3029 Integrating sphere).

2. Synthetic procedures:

General procedure for the synthesis of diarylmethanols 1a-j:

The corresponding *ortho*-bromo-1,3-dioxolanylbenzene (1 mmol) was dissolved in dry THF (50 mL), cooled to -78 °C and then *n*-BuLi in *n*-hexane (2.6 M, 1.2 mmol) was added. The resulting mixture was stirred for 40 min under argon atmosphere. Next, mono, di- or tri-methoxy substituted benzaldehyde (1.1 mmol) in dry THF (3 mL), was added at -78 °C and stirring was continued for 1.5 h from -78 °C to room temperature. Then, saturated aqueous solution of NH_4Cl was added and the organic layer was evaporated. The residue was diluted with ethyl acetate (50 mL) and washed with water (3×20 mL). The organic layer was dried ($MgSO_4$) and then filtrated. The solvent was removed in vacuum and the residue was purified by column chromatography (*n*-hexane/acetone in gradient as eluent) to give the corresponding diarymethanols **1b**, **1c**, **1e-j**. Compounds **1a** and **1d** were synthesized according to the literature.[19b]

General procedure for the synthesis of 4a-j:

In a 50 mL Schlenk tube, a solution of diarylmethanols **1a-j** (200 mg, 1 equiv.) in dry THF (10 mL) at 0 °C, triethyl amine (1.1 equiv.) was added and the reaction mixture was stirred at room temperature for 1 hour. After cooling, the mixture, again to 0 °C, chlorodiphenylphosphine (1.2 equiv.) was added and stirred at room temperature for another 3 hours. Then, a catalytic amount of TMSOTf (10 mol%) was added and the crude mixture was stirred overnight in an oil bath at 60 °C. Once the intermediate was consumed (checked with ^{31}P NMR), the aqueous solution of HCl (2 mL, 12 N) was added and the crude mixture was stirred for 1 hour. After evaporation of the solvent, the organic layer was dissolved in ethyl acetate and washed with water (5 × 2 mL), then with $NaHCO_3$ (5 mL) and extracted with ethyl acetate (10 mL). After drying over $MgSO_4$ and evaporation of the solvent, the product was purified by flash chromatography (*n*-hexane/EtOAc) to give pure anthracene compounds **4a-j**.

(6-(1,3-dioxan-2-yl)benzo[d][1,3]dioxol-5-yl)(3,5-dimethoxyphenyl)methanol:

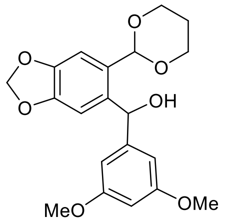

1b: R_f = 0.42 (EtOAc: *n*-hexane, 1:1 v/v); white crystals; m. p. = 126 – 128 °C; Yield = 62%. **^{1}H NMR** (400 MHz, C_6D_6) δ 7.39 (s, 1H), 7.03 – 7.01 (m, 1H), 6.98 (s, 1H), 6.55 (t, *J* = 2.3 Hz, 1H), 6.36 (s, 1H), 5.52 (s, 1H), 5.22 (d, *J* = 1.5 Hz, 1H), 5.18 (d, *J* = 1.4 Hz, 1H), 3.76 - 3.70 (m, 2H), 3.55 - 3.54 (m, 1H), 3.40 - 3.32 (m, 3H), 3.34 (s, 6H), 1.81 - 1.69 (m, 1H), 0.57 (ddt, *J* = 13.7, 2.9, 1.5 Hz, 1H); **^{13}C{^{1}H} NMR** (101 MHz, C_6D_6) δ 161.5, 148.5, 147.4, 146.5, 137.8, 131.1, 109.3, 107.5, 104.9, 101.2, 100.5, 99.8, 71.6, 67.2, 67.1, 54.8, 25.5; **HRMS** (TOF MS AP+): *m/z* Calc. for $C_{20}H_{22}O_7$-Na: 397.1263; Found: 397.1270.

(6-[1,3]Dioxan-2-yl-benzo[1,3]dioxol-5-yl)-(3-methoxy-phenyl)-methanol:

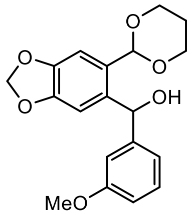

1c: Colorless liquid; R_f = 0.43 (EtOAc: *n-h*exane, 1:1 v/v); Yield = 68%. **^{1}H NMR** (CD_2Cl_2) (400 MHz): 1.43 (dtt, $^2J_{HH}$ = 13.6 Hz, $^3J_{HH}$ = 2.6 Hz, $^3J_{HH}$ = 1.4 Hz, 1H, $OCH_2C\underline{H}_2CH_2O$), 2.18 (tq, $^3J_{HH}$ = 13.6 Hz, $^3J_{HH}$ = 5.0 Hz, 1H, $OCH_2C\underline{H}_2CH_2O$), 3.44 (d, $^3J_{HH}$ = 3.5 Hz, 1H, OH), 3.78 (s, 3H, OCH_3), 3.84-4.02 (m, 2H, $OC\underline{H}_2CH_2CH_2O$), 4.14-4.30 (m, 2H, $OCH_2CH_2C\underline{H}_2O$), 5.61 (s, 1H, OCHO), 5.89 (d, $^2J_{HH}$ = 1.3 Hz, 1H, OCH_2O), 5.91 (d, $^2J_{HH}$ = 1.3 Hz, 1H, OCH_2O), 6.18 (d, $^3J_{HH}$ = 3.5 Hz, 1H, $C\underline{H}OH$), 6.60 (s, 1H, =CH), 6.80 (ddd, $^3J_{HH}$ = 8.2 Hz, $^4J_{HH}$ = 2.6 Hz, $^4J_{HH}$ = 0.7 Hz, 1H, =CH), 6.94 (ddd, $^3J_{HH}$ = 7.6 Hz, $^4J_{HH}$ = 1.7 Hz, $^4J_{HH}$ = 0.7 Hz, 1H, =CH), 6.97-7.01 (m, 1H, =CH), 7.06 (s, 1H, =CH), 7.25 (dd, $^3J_{HH}$ = 7.6 Hz, $^3J_{HH}$ = 8.2 Hz, 1H, =CH); **^{13}C{^{1}H} NMR** (CD_2Cl_2) (400 MHz): (DEPT 135) 24.3 ($OCH_2\underline{C}H_2CH_2O$, CH_2), 53.8 (OCH_3, CH_3), 66.1 ($O\underline{C}H_2CH_2\underline{C}H_2O$, CH_2), 69.4 (CHOH, CH), 98.7 (OCHO, CH), 100.2 (OCH_2O, CH_2), 105.5 (>CH, CH), 107.1 (>CH, CH), 110.7 (>CH, CH), 111.1 (>CH, CH), 117.4 (>CH, CH), 127.9 (>CH, CH), 128.9 (>$\underline{C}$-CHO, >C<), 135.5 (>$\underline{C}$-CHOH, >C<), 143.9 (>$\underline{C}$-CHOH, >C<), 145.5 (>$\underline{C}$-OCH_2, >C<), 146.6 (>$\underline{C}$-OCH_2, >C<), 158.4 (>$\underline{C}$-OCH_3, >C<); **HRMS** (TOF MS ES+): *m/z* Calc. for $C_{19}H_{20}O_6Na$: 367.1158; Found: 367.1159.

(2-[1,3]Dioxan-2-yl-4-fluoro-phenyl)-(3,4,5-trimethoxy-phenyl)-methanol:

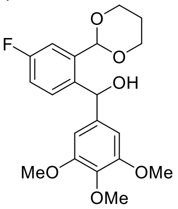

1e: White crystals; m.p. = 101 – 102 °C; R_f = 0.52 (EtOAc:*n*-hexane, 1:1 v/v); Yield = 84%; **^{19}F{^{1}H} NMR** (376 MHz, CD_2Cl_2): - 114.31 {ddd, $^3J_{HF}$ = 10.0 Hz, $^3J_{HF}$ = 8.6 Hz, $^4J_{HF}$ = 5.8 Hz in ^{19}F}; **^{1}H NMR** (400 MHz, CD_2Cl_2): 1.15 (dtt, $^3J_{HH}$ = 13.5 Hz, $^3J_{HH}$ = 2.6 Hz, $^2J_{HH}$ = 1.4 Hz, 1H, CH_2); 2.20 (dtt, $^2J_{HH}$ = 13.5 Hz, $^3J_{HH}$ = 5.1 Hz, $^3J_{HH}$ = 5.2 Hz, 1H, CH_2), 3.48 (d, $^3J_{HH}$ = 3.7 Hz, 1H, OH), 3.75 (s, 3H, OCH_3), 3.78 (s, 6H, 2x OCH_3), 3.86 – 4.04 (m, 2H, OCH_2), 4.18 – 4.33 (m, 2H, OCH_2), 5.61 (s 1H, OCHO), 6.13 (d, $^3J_{HH}$ = 3.7 Hz, 1H, CHOH), 6.62 (s, 2H, 2x =CH), 6.97 ($dd_{AB}d$, $^3J_{HF}$ = 8.6 Hz, $^3J_{HHAB}$ = 8.6 Hz, $^4J_{HH}$ = 2.8 Hz, 1H, =CH) {$d_{AB}d$, $^3J_{HHAB}$ = 8.6 Hz, $^4J_{HH}$ = 2.8 Hz in ^{1}H{^{19}F}}, 7.15 ($d_{AB}d$, $^3J_{HHAB}$ = 8.6 Hz, $^4J_{HF}$ = 5.8 Hz, 1H, =CH) {d_{AB}, $^3J_{HHAB}$ = 8.6 Hz in ^{1}H{^{19}F}}, 7.31 (dd, $^3J_{HF}$ = 10.0 Hz, $^4J_{HH}$ = 2.8 Hz, 1H, =CH) {d, $^4J_{HH}$ = 2.8 Hz in ^{1}H{^{19}F}}; **^{13}C{^{1}H} NMR** (101 MHz, CD_2Cl_2): 24.2 (s, CH_2, CH_2), 54.5 (s, 2x OCH_3, CH_3), 59.1 (s, OCH_3, CH_3), 66.2 (s, 2x OCH_2, CH_2), 69.7 (s, CHOH, CH), 98.1 (d, $^5J_{CF}$ = 1.3 Hz, OCHO, CH), 102.1 (s, 2x =CH, CH), 112.3 (d, $^2J_{CF}$ = 23.4 Hz, =CH, CH), 114.3 (d, $^2J_{CF}$ = 21.0 Hz, =CH, CH), 129.2 (d, $^3J_{CF}$ = 8.0 Hz, =CH, CH), 135.5 (s, =C-Ar, >C<), 136.9 (d, $^4J_{CF}$ = 3.1 Hz, =C-TMB, >C<), 137.1 (d, $^3J_{CF}$ = 7.4 Hz, =C-CO, >C<), 137.3 (s, =C-CH_3, >C<), 151.8 (s, 2x =C-OCH_3, >C<), 160.6 (d, $^1J_{CF}$ = 245.4 Hz, =C-F, >C<); **HRMS** (TOF MS ES+): *m/z* Calc. for $C_{20}H_{23}O_6F$+Na: 401.1376; Found: 401.1390.

(2-[1,3]Dioxan-2-yl-5-trifluoromethyl-phenyl)-(3,4,5-trimethoxy-phenyl)-methanol:

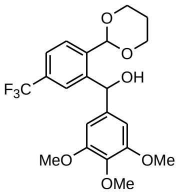

1f: White crystals; m. p. = 84 – 86 °C; R_f = 0.35 (EtOAc: *n-h*exane, 1:1 v/v); R_f = 0.72 (EtOAc); Yield = 78%. **^{19}F{^{1}H} NMR** (376 MHz, C_6D_6): - 61.59; **^{19}F{^{1}H} NMR** (376 MHz, CD_2Cl_2): -62.33; **^{1}H NMR** (400 MHz, C_6D_6): 0.62 – 0.77 (m, 1H, CH_2), 1.85 (dtt, $^{2}J_{HH}$ = 13.3 Hz, $^{3}J_{HH}$ = 5.1 Hz, $^{3}J_{HH}$ = 5.2 Hz, 1H, CH_2), 3.32 – 3.57 (m, 2H, OCH_2), 3.43 (s, 6H, 2x OCH_3), 3.70 (brs, 1H, OH), 3.73 – 3.99 (m, 2H, OCH_2), 3.86 (s, 3H, OCH_3), 5.56 (s, 1H, OCHO), 6.41 (s, 1H, CHOH), 6.87 (s, 2H, 2x =CH), 7.42 (dd, $^{3}J_{HH}$ = 8.2 Hz, $^{4}J_{HH}$ = 1.6 Hz, 1H, =CH), 7.82 (d, $^{3}J_{HH}$ = 8.2 Hz, 1H, =CH), 8.07 (d, $^{4}J_{HH}$ = 1.6 Hz, 1H, =CH); ^{1}H (CD_2Cl_2): 1.47 (dddd, $^{3}J_{HH}$ = 12.5 Hz, $^{3}J_{HH}$ = 2.5 Hz, $^{3}J_{HH}$ = 5.2 Hz, $^{3}J_{HH}$ -= 5.0 Hz, 1H, CH_2); 2.23 (dtt, $^{2}J_{HH}$ = 17.5 Hz, $^{3}J_{HH}$ = 12.5 Hz, $^{3}J_{HH}$ = 5.0 Hz, 1H, CH_2), 3.55 (d, $^{2}J_{HH}$ = 4.0 Hz, 1H, OH), 3.77 (s, 3H, OCH_3), 3.78 (s, 6H, 2x OCH_3), 3.83 – 4.04 (m, 2H, OCH_2), 4.18 – 4.34 (m, 2H, OCH_2), 5.65 (s, 1H, OCHO), 6.26 (d, $^{2}J_{HH}$ = 4.0 Hz, 1H, CHOH), 6.63 (s, 2H, 2x =CH), 7.53 – 7.63 (m, 2H, =CH, =CH), 7.73 (d, $^{3}J_{HH}$ = 8.6 Hz, 1H, =CH); **^{13}C{^{1}H} NMR** (101 MHz, CD_2Cl_2): 24.2 (s, CH_2), 54.5 (s, 2x OCH_3), 59.1 (s, OCH_3), 66.2 (s, 2x OCH_2), 69.8 (s, CHOH), 98.4 (s, OCHO), 102.2 (s, 2x =CH), 122.8 (q, $^{1}J_{CF}$ = 272.4 Hz, CF_3), 122.9 (q, $^{3}J_{CF}$ = 3.7 Hz, =CH), 123.8 (q, $^{3}J_{CF}$ = 3.8 Hz, =CH), 126.2 (s, =CH), 129.5 (q, $^{2}J_{CF}$ = 32.2 Hz, =C-CF_3), 135.7 (s, =C-O), 136.7 (s, =C-CO), 138.1 (q, $^{4}J_{CF}$ = 0.7 Hz, =C-CHOH), 142.1 (s, =C-CHOH), 151.9 (s, 2x =C-O); **HRMS** (TOF MS ES+): *m/z* Calc. for $C_{21}H_{23}O_6F_3$+Na: 451.1344; Found: 451.1345.

(4-[1,3]Dioxan-2-yl-3-[hydroxy-(3,4,5-trimethoxy-phenyl)-methyl]-benzonitrile:

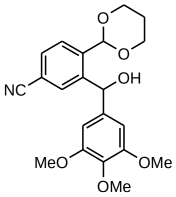

1g: Yellow crystals; m. p. = 52 – 58 °C; R_f = 0.23 (EtOAc: *n-h*exane, 1:1 v/v); Yield = 68%. **^{1}H NMR** (400 MHz, CD_2Cl_2): 1.40 – 1.54 (m, 1H, CH_2); 2.09 – 2.35 (m, 1H, CH_2), 3.74 (s, 1H, OH), 3.77 (s, 3H, OCH_3), 3.79 (s, 6H, 2x OCH_3), 3.89 – 4.05 (m, 2H, OCH_2), 4.18 – 4.36 (m, 2H, OCH_2), 5.67 (s, 1H, OCHO), 6.23 (s, 1H, CHOH), 6.60 (d, $^{4}J_{HH}$ = 0.5 Hz, 2H, 2x =CH), 7.51 – 7.62 (m, 2H, =CH, =CH), 7.70 (d, $^{3}J_{HH}$ = 7.9 Hz, 1H, =CH); **^{13}C{^{1}H} NMR** (101 MHz, CD_2Cl_2): 24.2 (s, CH_2), 54.6 (s, 2x OCH_3), 59.1 (s, OCH_3), 66.2 (s, 2x OCH_2), 69.2 (s, CHOH), 97.9 (s, OCHO), 102.3 (s, 2x =CH), 111.5 (s, =C-CN), 117.3 (s, CN), 126.3 (s, =CH), 129.7 (s, =CH), 130.6 (s, =CH), 135.8 (s, =C-COH), 136.6 (s, =C-C-OCH_2), 138.9 (s, =C-O), 142.5 (s, =C-CHOH), 151.9 (s, 2x =C-O); HRMS (TOF MS ES+): *m/z* Calc. for $C_{21}H_{23}NO_6$+H: 386.1604; Found: 386.1601; **HRMS** (TOF MS ES+): *m/z* Calc. for $C_{21}H_{23}NO_6$+Na: 408.1423; Found: 408.1424.

(4-Bromo-2-[1,3]dioxan-2-yl-phenyl)-(3,4,5-trimethoxy-phenyl)-methanol:

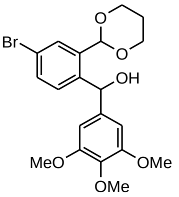

1h: Colorless liquid; R_f = 0.28 (*n*-*h*exane:EtOAc 1:1 v/v); Yield = 67%. **^{1}H NMR** (400 MHz, CD_2Cl_2) (400 MHz): 1.47 – 1.54 (m, 1H, CH_2); 2.25 (dtt, $^{2}J_{HH}$ = 17.5 Hz, $^{3}J_{HH}$ = 12.5 Hz, $^{3}J_{HH}$ = 5.0 Hz, 1H, CH_2), 3.47 (d, $^{3}J_{HH}$ = 3.9 Hz, 1H, OH), 3.80 (s, 3H, OCH_3), 3.82 (s, 6H, 2x OCH_3), 3.93 – 4.05 (m, 2H, OCH_2), 4.25 – 4.35 (m, 2H, OCH_2), 5.64 (s, 1H, OCHO), 6.18 (d, $^{3}J_{HH}$ = 3.9 Hz, 1H, CHOH), 6.65 (s, 2H, 2x =CH), 7.11 (d, $^{3}J_{HH}$ = 8.3 Hz, 1H, =CH), 7.46 (dd, $^{3}J_{HH}$ = 8.3 Hz, $^{4}J_{HH}$ = 1.7 Hz, 1H, =CH), 7.78 (d, $^{4}J_{HH}$ = 1.7 Hz, 1H, =CH); **^{13}C{^{1}H} NMR** (400 MHz, CD_2Cl_2) (400 MHz): 25.6 (s, CH_2), 55.9 (s, 2x OCH_3), 60.4 (s, OCH_3), 67.5 (s, 2x OCH_2), 71 (s, CHOH), 99.5 (s, OCHO), 103.5 (s, 2x =CH), 121.4 (s, =CBr), 136.9 (s, =C-CHOH), 138.4 (s, =C-O), 138.5 (s, =C-CO), 141.4 (s, =C-CHOH), 153.2 (s, 2x =C-O); **HRMS** (TOF MS APCI+): *m/z* Calc. for $C_{20}H_{23}O_6Br$-H: 437.0600/439.0579; Found: 437.0593/439.0580.

(6-[1,3]Dioxan-2-yl-2,3,4-trifluoro-phenyl)-(3,4,5-trimethoxy-phenyl)-methanol:

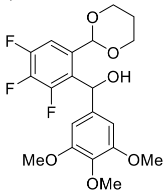

1i: White crystals; m.p. = 114 -115 °C; R_f = 0.25 (*n*-hexane:EtOAc 1:1 v/v);; Yield = 86% (96% by ^{19}F NMR); **^{19}F{^{1}H} NMR** (376 MHz, $CDCl_3$): -133.88 (dd, $^{3}J_{FF}$ = 21.1 Hz, $^{4}J_{FF}$ = 7.6 Hz, 1F) {ddd, $^{3}J_{FF}$ = 21.1 Hz, $^{4}J_{FF}$ = 7.6 Hz, $^{3}J_{HF}$ = 11.0 Hz in ^{19}F}, -136.12 (dd, $^{3}J_{FF}$ = 20.5 Hz, $^{4}J_{FF}$ = 7.6 Hz, 1F) {dd, $^{3}J_{FF}$ = 20.5 Hz, $^{4}J_{FF}$ = 7.6 Hz, in ^{19}F}, -158.20 (dd, $^{3}J_{FF}$ = 21.1 Hz, $^{3}J_{FF}$ =20.5 Hz, 1F) {ddd, $^{3}J_{FF}$ = 21.1 Hz, $^{3}J_{FF}$ =20.5 Hz, $^{4}J_{HF}$ = 7.3 Hz in ^{19}F}; **^{1}H NMR** (400 MHz, $CDCl_3$): 1.41 (dtt, $^{3}J_{HH}$ = 13.5 Hz, $^{3}J_{HH}$ = 2.6 Hz, $^{2}J_{HH}$ = 1.4 Hz, 1H, CH_2); 2.16 (dtt, $^{2}J_{HH}$ = 13.5 Hz, $^{3}J_{HH}$ = 5.1 Hz, $^{3}J_{HH}$ = 5.2 Hz, 1H, CH_2), 3.66 – 3.80 (m, 2H, OCH_2), 3.83 (s, 6H, 2x OCH_3), 3.87 (s, 3H, OCH_3), 4.01 (d, $^{3}J_{HH}$ = 9.2 Hz, 1H, OH), 4.16 – 4.22 (m, 2H, OCH_2), 5.15 (s 1H, OCHO), 6.28 (d, $^{3}J_{HH}$ = 9.2 Hz, 1H, CHOH), 6.59 (s, 2H, 2x =CH), 7.41 (ddd, $^{3}J_{HF}$ = 11.0 Hz, $^{4}J_{HF}$ = 7.3 Hz, $^{5}J_{HF}$ = 1.9 Hz, 1H, =CH) {s in ^{1}H{^{19}F}}; ^{13}C{^{1}H} ($CDCl_3$): 25.17 (s, CH_2, CH_2), 56.00 (s, 2x OCH_3, CH_3), 60.78 (s, OCH_3, CH_3), 66.82 (d, $^{3}J_{CF}$ = 5.1 Hz, CHOH, CH), 67.24 (s, OCH_2, CH_2), 67.29 (s, OCH_2, CH_2), 97.72 (s, OCHO, CH), 102.39 (s, 2x=CH, CH), 111.32 (dd, $^{2}J_{CF}$ = 19.2 Hz, $^{3}J_{CF}$ = 3.1 Hz, =CH, CH), 126.66 (dd, $^{2}J_{CF}$ = 10.0 Hz, $^{3}J_{CF}$ = 3.1 Hz, =C-CHOH, >C<), 132.74 (ddd, $^{3}J_{CF}$ = 7.2 Hz, $^{3}J_{CF}$ = 4.1 Hz, $^{4}J_{CF}$ = 3.0 Hz, =C-CHO, >C<), 136.81 (s, =C-OCH_3, >C<), 138.00 (s, =C-CHOH, >C<), 139.74 (ddd, $^{1}J_{CF}$ = 253.6 Hz, $^{2}J_{CF}$ =15.8 Hz, $^{2}J_{CF}$ =15.8 Hz, =CF, >C<), 149.84 (ddd, $^{1}J_{CF}$ = 250.0 Hz, $^{2}J_{CF}$ = 9.8 Hz, $^{3}J_{CF}$ = 9.8 Hz, =CF, >C<), 149.87 (ddd, $^{1}J_{CF}$ = 249.7 Hz, $^{2}J_{CF}$ =9.8 Hz 10.1 Hz, $^{3}J_{CF}$ = 9.8 Hz, =CF, >C<), 153.09 (s, 2x=C-OCH_3, >C<); **^{13}C{^{1}H} NMR** (101 MHz, CD_2Cl_2): 25.3 (s, CH_2, CH_2), 55.8 (s, 2x OCH_3, CH_3), 60.4 (s, OCH_3, CH_3), 66.9 (dd, $^{3}J_{CF}$ = 3.7 Hz, $^{4}J_{CF}$ = 1.5 Hz, CHOH, CH), 67.3 (s, OCH_2, CH_2), 67.4 (s, OCH_2, CH_2), 97.9 (s, OCHO, CH), 102.5 (s, 2x=CH, CH), 111.1 (dd, $^{2}J_{CF}$ = 19.1 Hz, $^{3}J_{CF}$ = 2.9 Hz, =CH, CH), 127.1 (dd, $^{2}J_{CF}$ = 9.9 Hz, $^{3}J_{CF}$ = 4.4 Hz, $^{4}J_{CF}$ = 1.6 Hz, =C-CHOH, >C<), 133.1 (ddd, $^{3}J_{CF}$ = 7.2 Hz, $^{3}J_{CF}$ = 4.1 Hz, $^{4}J_{CF}$ = 3.0 Hz, =C-CHO, >C<), 136.8 (s, =C-OCH_3, >C<), 138.1 (d, $^{4}J_{CF}$ = 0.5 Hz, =C-CHOH, >C<), 139.6 (ddd, $^{1}J_{CF}$ = 252.0 Hz, $^{2}J_{CF}$ =16.5 Hz, $^{2}J_{CF}$ =15.2 Hz, =CF, >C<), 149.8 (ddd, $^{1}J_{CF}$ = 249.3 Hz, $^{2}J_{CF}$ = 3.8 Hz, $^{3}J_{CF}$ = 3.3 Hz, =CF, >C<), 149.9 (ddd, $^{1}J_{CF}$ = 249.6 Hz, $^{2}J_{CF}$ =4.1 Hz 10.1 Hz, $^{3}J_{CF}$ =

4.1 Hz, =CF, >C<), 153.1 (s, 2x=C-OCH_3, >C<); **HRMS** (MS ES+): *m/z* Calc. for $C_{20}H_{21}O_6F_3$+Na: 437.1188; Found: 437.1183.

(2-[1,3]Dioxan-2-yl-4,5-difluoro-phenyl)-(3,4,5-trimethoxy-phenyl)-methanol:

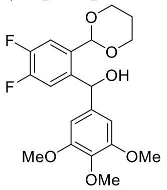

1j: White crystals; m.p. = 112 – 114 °C; R_f = 0.22 (EtOAc:*n*-hexane, 1:1 v/v); **$^{19}F\{^1H\}$ NMR** (376 MHz, CD_2Cl_2): - 137.32 (d, $^3J_{FF}$ = 21.8 Hz, 1F), - 139.37 (d, $^3J_{FF}$ = 21.8 Hz, 1F); **^1H** (400 MHz, CD_2Cl_2): 1.46 (dtt, $^3J_{HH}$ = 13.5 Hz, $^3J_{HH}$ = 2.6 Hz, $^2J_{HH}$ = 1.4 Hz, 1H, CH_2); 2.20 (dtt, $^2J_{HH}$ = 13.5 Hz, $^3J_{HH}$ = 5.1 Hz, $^3J_{HH}$ = 5.2 Hz, 1H, CH_2), 3.40 (d, $^3J_{HH}$ = 3.8 Hz, 1H, OH), 3.77 (s, 3H, OCH_3), 3.79 (s, 6H, 2x OCH_3), 3.85 – 4.05 (m, 2H, OCH_2), 4.18 – 4.32 (m, 2H, OCH_2), 5.60 (s 1H, OCHO), 6.12 (d, $^3J_{HH}$ = 3.8 Hz, 1H, CHOH), 6.60 (s, 2H, 2x =CH), 7.01 (dd, $^4J_{HF}$ = 8.1 Hz, $^3J_{HF}$ = 11.7 Hz, 1H, =CH) {s in $^1H\{^{19}F\}$}, 7.44 (dd, $^4J_{HF}$ = 8.2 Hz, $^3J_{HF}$ = 11.6 Hz, 1H, =CH) {s in $^1H\{^{19}F\}$}; **$^{13}C\{^1H\}$ NMR** (101 MHz, CD_2Cl_2): 24.1 (s, CH_2), 54.6 (s, 2x OCH_3), 59.1 (s, OCH_3), 66.2 (s, 2x OCH_2), 69.2 (s, CHOH), 97.6 (s, OCHO), 102.1 (s, 2x =CH), 114.6 (d, $^2J_{CF}$ = 19.0 Hz, =CH), 116 (d, $^2J_{CF}$ = 18.2 Hz, =CH), 131.8 (dd, $^3J_{CF}$ = 5.4 Hz, $^4J_{CF}$ = 3.7 Hz, =C-CO), 135.7 (s, =C-OCH_3), 136.5 (s, =C-C), 138.4 (dd, $^3J_{CF}$ = 4.3 Hz, $^4J_{CF}$ = 4.3 Hz, =C-C), 147.8 (dd, $^1J_{CF}$ = 247.1 Hz, $^2J_{CF}$ = 12.5 Hz, =CF), 148.8 (dd, $^1J_{CF}$ = 248.4 Hz, $^2J_{CF}$ = 12.4 Hz, =CF), 151.9 (s, 2x =C-OCH_3, >C<); **HRMS** (TOF MS ES+): *m/z* Calc. for $C_{20}H_{22}O_6F_2$+Na: 419.1282; Found: 419.1285.

(7,8,9-Trimethoxyanthra[2,3-d][1,3]dioxol-5-yl) diphenyl phosphine oxide:

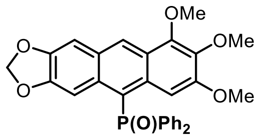

4a: R_f = 0.43 (EtOAc), *n*-hexane:EtOAc (1:2), green solid, m.p. 202-204 °C, 81 mg, 32% yield: **^1H NMR** (400 MHz, CD_2Cl_2) δ 8.72 (s, 1H), 8.32 (s, 1H), 7.71-7.65 (m, 4H), 7.55-7.50 (m, 2H), 7.46-7.41 (m, 4H), 7.27(s, 1H), 7.22 (s, 1H), 5.99 (s, 2H), 4.10 (s, 3H), 3.89 (s, 3H), 3.25 (s, 3H); **$^{13}C\{^1H\}$ NMR** (101 MHz, CD_2Cl_2) δ 152.9 (s), 148.9 (s), 146.7 (d, J_{PC} = 2.2 Hz), 146.6 (s), 139.1 (s), 136.2 (d, J_{PC} = 102.4 Hz), 134.4 (d, J_{PC} = 8.4 Hz), 131.6 (d, J_{PC} = 9.5 Hz), 131.4 (d, J_{PC} = 2.7 Hz), 131.3 (d, J_{PC} = 9.9 Hz), 128.8 (d, J_{PC} = 12.1 Hz), 128.3 (d, J_{PC} = 11.0 Hz), 126.4 (d, J_{PC} = 3.1 Hz), 123.1 (d, J_{PC} = 10.9 Hz), 117.2 (d, J_{PC} = 100.4 Hz), 103.5 (s), 102.5 (d, J_{PC} = 7.0 Hz), 102.1 (d, J_{PC} = 7.9 Hz), 101.5 (s), 61.51 (s), 60.8 (s), 55.3 (s); **^{31}P NMR** (162 MHz, CD_2Cl_2) δ 30.54; **HRMS** (TOF MS ES+): calcd. for $C_{30}H_{26}O_6P$ [M+H$^+$] 513.1467, found 513.1462.

(7,9-Dimethoxyanthra[2,3-d][1,3]dioxol-5-yl) diphenyl phosphine oxide:

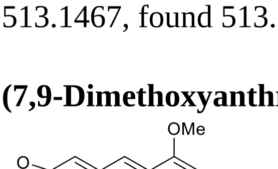

4b: R_f = 0.50 (EtOAc), *n*-hexane:EtOAc (1:2), yellow crystals, m.p. 232-234 °C, 78 mg, 30% yield: **^1H NMR** (400 MHz, CD_2Cl_2) δ 8.84 (s, 1H), 8.46 (s, 1H), 7.72-7.66 (m, 4H), 7.53-7.49 (m, 2H), 7.45-7.40 (m, 4H), 7.25 (d, *J* = 1.7 Hz, 1H), 6.80 (s, 1H), 6.31 6.31 (d, *J* = 2.0 Hz, 1H), 5.99 (s, 2H), 3.99 (s, 3H), 3.18 (s, 3H); **$^{13}C\{^1H\}$ NMR** (101 MHz, CD_2Cl_2) δ 158.1 (s), 156.8 (d, J_{PC}= 2.2 Hz), 149.7 (s), 146.8 (s),136.7 (d, J_{PC} = 102.5 Hz), 135.9 (d, J_{PC} = 8.4 Hz),

135.4 (d, J_{PC} = 9.5 Hz), 131.7 (d, J_{PC} = 2.3 Hz), 131.6 (d, J_{PC} = 10.3 Hz), 129.1 (d, J_{PC} = 11.9 Hz), 128.1 (d, J_{PC} = 11.1 Hz), 127.4 (d, J_{PC} = 3.0 Hz), 120.8 (d, J_{PC} = 11.3 Hz), 117.2 (d, J_{PC} = 101.2 Hz), 104.3 (s), 102.9 (d, J_{PC} = 6.7 Hz), 101.9 (s), 98.1 (d, J_{PC} = 8.3 Hz), 96.6 (s), 56.1 (s), 55.3 (s).**^{31}P NMR** (162 MHz, CD_2Cl_2) δ 30.56; **HRMS** (TOF MS ES+): calcd. for $C_{29}H_{24}O_5P$ [M+H$^+$] 483.1361, found 483.1358.

(7-Methoxyanthra[2,3-d][1,3]dioxol-5-yl) diphenyl phosphine oxide:

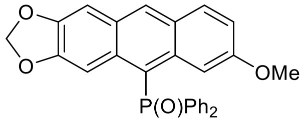

4c: R_f = 0.53 (EtOAc), *n*-hexane:EtOAc (1:2), green solid, m.p. 227-229 °C, 60 mg, 24% yield: **^{1}H NMR** (400 MHz, CD_2Cl_2) δ 8.42 (d, *J* = 12.0 Hz, 2H), 7.83 (dd, *J* = 9.1, 1.9 Hz, 1H), 7.72-7.67 (m, 4H), 7.55-7.50 (m, 2H), 7.46-7.41 (m, 4H), 7.31 (d, *J* = 2.4 Hz, 1H), 7.23 (d, *J* = 1.7 Hz, 1H), 7.00 (dd, *J* = 9.1, 2.4 Hz, 1H), 6.00 (s, 2H), 3.22 (s, 3H); **^{13}C{^{1}H} NMR** (101 MHz, CD_2Cl_2) δ 157.5 (s), 149.6 (s), 147 (s), 136.6 (d, J_{PC} = 102.5 Hz), 135.6 (d, J_{PC} = 8.5 Hz), 135.1 (d, J_{PC} = 8.8 Hz), 133 (d, J_{PC} = 3.1 Hz), 131.8 (d, J_{PC} = 2.8 Hz), 131.7 (d, J_{PC} = 9.9 Hz), 130.6 (s), 129.2 (d, J_{PC} = 12.2 Hz), 128.6 (d, J_{PC} = 11.3 Hz), 127.1 (d, J_{PC} = 10.8 Hz), 119.3 (s), 117.7 (d, J_{PC} = 100.1 Hz), 105.1 (d, J_{PC} = 7.8 Hz), 103.5 (s), 103 (d, J_{PC} = 6.8 Hz), 102 (s), 55.2 (s); **^{31}P NMR** (162 MHz, CD_2Cl_2) δ 30.28; **HRMS** (TOF MS ES+): calcd. for $C_{28}H_{21}O_4P$ [M+H$^+$] 453.1256, found 453.1256.

(2,3,4-Trimethoxyanthr-9-yl) diphenyl phosphine oxide:

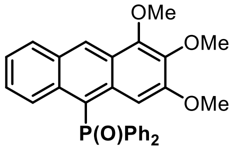

4d: R_f = 0.41 (EtOAc), *n*-hexane:EtOAc (1:2), yellow solid, m.p. 150-151 °C, 96 mg, 37% yield: **^{1}H NMR** (400 MHz, CD_2Cl_2) δ 8.98 (d, *J* = 1.7 Hz, 1H), 8.62 (dt, *J* = 9.1, 1.0 Hz, 1H), 8.07 (dt, *J* = 8.4, 1.8 Hz, 1H), 7.73-7.67 (m, 4H), 7.61(s, 1H), 7.54-7.49(m, 4H), 7.46-7.40 (m, 2H), 7.39-7.36 (m, 1H), 7.27-7.23 (m, 1H), 4.15 (s, 3H), 3.94 (s, 3H), 3.39 (s, 3H); **^{13}C{^{1}H} NMR** (101 MHz, CD_2Cl_2) δ 154 (s), 146.9 (s), 139.7 (s), 136.6 (d, J_{PC} = 102.5 Hz), 135.2 (d, J_{PC} = 8.5 Hz), 133.4 (d, J_{PC} = 9.0 Hz), 131.5 (d, J_{PC} = 2.9 Hz), 131.4 (d, J_{PC} = 9.8 Hz), 130.2 (d, J_{PC} = 10.9 Hz), 129.7 (s), 128.9 (d, J_{PC} = 11.9 Hz), 128.3 (d, J_{PC} = 3.2 Hz), 126.9 (d, J_{PC} = 7.0 Hz), 126.3 (s), 124.6 (d, J_{PC} = 11.1 Hz), 124.3 (s), 118.7 (d, J_{PC} = 100.1 Hz), 101.9 (d, J_{PC} = 7.5 Hz), 61.7 (s), 61.1 (s), 55.5 (s); **^{31}P NMR** (162 MHz, CD_2Cl_2) δ 29.57; **HRMS** (TOF MS ES+): calcd. for $C_{29}H_{25}O_4P$ [M+H$^+$] 469.1568, found 469.1569.

(6-Fluoro-2,3,4-trimethoxyanthr-9-yl) diphenyl phosphine oxide:

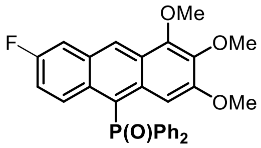

4e: R_f = 0.46 (EtOAc), *n*-hexane:EtOAc (1:2), yellow solid, m.p. 154-156 °C, 78 mg, 32% yield: **^{1}H NMR** (400 MHz, CD_2Cl_2) δ 8.97-8.91 (m, 2H), 7.71-7.66 (m, 5H), 7.56-7.51 (m, 2H), 7.47-7.42 (m, 4H), 7.36 (s, 1H), 7.15-7.10 (m, 1H), 4.14 (s, 3H), 3.93 (s, 3H), 3.31 (s, 3H); **^{13}C{^{1}H} NMR** (101 MHz, CD_2Cl_2) δ 159.41(d, J_{CF} = 247.4 Hz), 154.1 (s), 146.8 (s), 140.4 (s), 136.4 (d, J_{PC} =102.8 Hz), 133.1 (d, J_{PC} = 8.3Hz), 132.7 (d, J_{PC} =8.7 Hz), 132 (d, J_{PC} = 2.8 Hz), 131.6 (d, J_{PC} = 10 Hz), 131.2 (dd, J_{PC} = 10.1, J_{CF} = 19.7 Hz), 130.2 (dd, J_{PC} = 7.5, J_{CF} = 15.2 Hz), 129.2 (d, J_{PC} = 12.1 Hz), 127.5 (dd, *J* = 6.7, 3.2 Hz), 125.6 (d, J_{PC} = 11 Hz), 119.9 (d,

J_{PC} = 99.7 Hz), 117.7 (d, J_{CF} = 26.1 Hz), 111.4 (d, J_{CF} = 20.0 Hz), 102.3 (d, J_{PC} = 7.8 Hz), 61.9 (s), 61.3 (s), 55.8 (s); **^{31}P NMR** (162 MHz, CD_2Cl_2) δ 29.87; **^{19}F{^{1}H} NMR** (376 MHz, CD_2Cl_2) δ -117.04; **HRMS** (TOF MS ES+): calcd. for $C_{29}H_{25}O_4PF$ [M+H$^+$] 487.1472, found 487.1474.

(2,3,4-Trimethoxy-7-(trifluoromethyl)anthr-9-yl) diphenyl phosphine oxide:

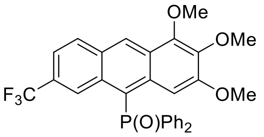

4f: R_f = 0.56 (EtOAc), *n*-hexane:EtOAc (1:2), green solid, m.p. 134-137 °C, 123 mg, 49% yield: **^{1}H NMR** (400 MHz, CD_2Cl_2) δ 9.22 (s, 1H), 9.06 (s, 1H), 8.20 (d, *J* = 8.8 Hz, 1H), 7.80-7.68 (m, 4H), 7.63(s, 1H), 7.80-7.68 (m, 4H), 4.17 (s, 3H), 3.97 (s, 3H), 3.44 (s, 3H); **^{13}C{^{1}H} NMR** (101 MHz, CD_2Cl_2) δ: 154.8 (s), 147.1 (s), 140.7 (s), 136.2 (d, J_{PC} = 103.1 Hz), 134.1 (d, J_{PC} = 8.5 Hz), 133.8 (d, J_{PC} = 8.1 Hz), 132.1 (d, J_{PC} = 3.1 Hz), 131.6 (d, J_{PC} = 9.9 Hz), 131.3 (s), 130.9 (d, J_{PC} = 10.5 Hz), 129.3 (d, J_{PC} = 12.2 Hz), 128.5 (d, J_{PC} = 3.1 Hz), 127.5 (q, J_{CF} = 31.6 Hz), 126.3 (d, J_{PC} = 10.5 Hz), 125.3 (dq, J_{PC} = 5.3 Hz, J_{PC} = 5.3 Hz), 124.7 (q, J_{CF} = 271.8 Hz), 121.3 (d, J_{PC} = 98.3 Hz), 119.5 (q, J_{CF} = 3.0 Hz), 102.4 (d, J_{PC} = 7.9 Hz), 62 (s), 61.3 (s), 55.9 (s); **^{31}P NMR** (162 MHz, CD_2Cl_2) δ 29.28; **^{19}F NMR** (376 MHz, CD_2Cl_2) δ -63.00; **HRMS** (TOF MS ES+): calcd. for $C_{30}H_{25}O_4PF_3$ [M+H$^+$] 537.1442, found 537.1443.

9-(Diphenylphosphoryl)-5,6,7-trimethoxyanthracene-2-carbonitrile:

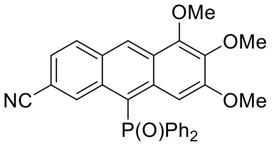

4g: R_f = 0.54 (EtOAc), *n*-hexane:EtOAc (1:2), yellow solid, m.p. 176-178 °C; 141mg, 55% yield: **^{1}H NMR** (400 MHz, CD_2Cl_2) δ 9.61 (s, 1H), 9.03 (s, 1H), 8.14 (dd, *J* = 8.8, 1.8 Hz, 1H), 7.75-7.70 (m, 4H), 7.59-7.54 (m, 2H), 7.50-7.45 (m, 5H), 7.30 (s, 1H), 4.15 (s, 3H), 3.95 (s, 3H), 3.33 (s, 3H); **^{13}C{^{1}H} NMR** (101 MHz, CD_2Cl_2) δ 155 (s), 147.1 (s), 140.9 (s), 135.8 (d, J_{PC} = 103.4 Hz), 134.3 (s), 134.2 (d, J_{PC} = 5.6 Hz), 133.7 (d, J_{PC} = 8.7 Hz), 132.3 (d, J_{PC} = 3.2 Hz), 131.6 (d, J_{PC} = 10.0 Hz), 131.2 (s), 130.8 (d, J_{PC} = 10.2 Hz), 129.3 (d, J_{PC} = 12.2 Hz), 128.6 (d, J_{PC} = 3.1 Hz), 126.6 (d, J_{PC} = 10.6 Hz), 124.1 (s), 121.1 (d, J_{PC} = 97.2 Hz), 119.6 (s), 109.9 (s), 102.4 (d, J_{PC} = 7.9 Hz), 62 (s), 61.3 (s), 55.9 (s); **^{31}P NMR** (162 MHz, CD_2Cl_2) δ 29.88; **HRMS** (TOF MS ES+): calcd. for $C_{30}H_{25}O_4PN$ [M+H$^+$] 494.1531, found 494.1521.

(6-Bromo-2,3,4-trimethoxyanthr-9-yl) diphenyl phosphine oxide:

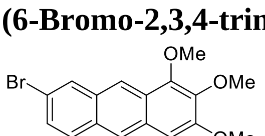

4h: R_f = 0.45 (EtOAc), *n*-hexane:EtOAc (1:2), orange oil, 112 mg, 45% yield: **^{1}H NMR** (400 MHz, CD_2Cl_2) δ 8.87 – 8.86 (m, 1H), 8.67 (d, *J* = 9.6 Hz, 1H), 8.22 (dd, *J* = 2.1, *J* = 2.1 Hz, 1H), 7.69 –7.64 (m, 4H), 7.55 – 7.51 (m, 2H), 7.46 –7.41 (m, 5H), 7.30 (dd, *J* = 9.6, 2.2 Hz, 1H), 4.12 (s, 3H), 3.92 (s, 3H), 3.33 (s, 3H); **^{13}C{^{1}H} NMR** (101 MHz, CD_2Cl_2) δ 154.2 (s), 146.8 (s), 140.1 (s), 36.1 (d, J_{PC} = 102.8 Hz),133.6 (d, J_{PC} = 8.6 Hz), 133.2 (d, J_{PC} = 8.7 Hz), 131.7 (d, J_{PC} = 2.7 Hz), 131.4 (d, J_{PC} = 10.1 Hz), 131.2 (s), 131.1 (s), 129.4 (s), 128.9 (d, J_{PC} = 12.1 Hz), 128.8 (d, J_{PC} = 6.5 Hz),127.2 (d, J_{PC} = 3.1 Hz), 125.3 (d, J_{PC} = 10.6 Hz), 119.5 (d, J_{PC} = 99.4 Hz), 118.2 (s), 102 (d, J_{PC} = 7.5 Hz), 61.7 (s), 61.1 (s), 55.5 (s); **^{31}P NMR** (162

MHz, CD_2Cl_2) δ 29.60; **HRMS** (TOF MS ES+): calcd. for $C_{29}H_{24}O_4PBr$ [M+H$^+$] 547.0672, found 547.067.

(1,2,3-Trifluoro-5,6,7-trimethoxyanthr-9-yl) diphenyl phosphine oxide:

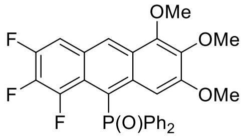

4i: R_f = 0.40 (EtOAc), *n*-hexane:EtOAc (1:2), yellow solid, m.p. 186-188 °C; 151 mg, 60% yield: **^{1}H NMR** (400 MHz, C_6D_6) δ 8.70 (s, 1H), 8.11 (s, 1H), 7.71 – 7.66 (m, 4H), 6.96 – 6.89 (m, 7H), 3.92 (s, 3H), 3.71 (s, 3H), 3.25 (s, 3H); **^{13}C{^{1}H} NMR** (101 MHz, C_6D_6) δ 155.1 (s), 149.1 (dd, J_{CF}= 251.2, 14.1 Hz), 146.9 (s), 146 (dd, J_{CF}= 251.03, 14.3 Hz), 140.4 (dd, J_{CF}= 251.8, 13.9 Hz), 141.1 (s), 138.2 (dd, J_{PC}=105.9, 4.3 Hz), 135.6 (d, J_{PC}= 6.8 Hz), 130.9 (dd, J_{PC}=9.5, 2.4 Hz), 130.7 (d, J_{PC} = 2.8 Hz), 129.3 (s), 128.4 (d, J_{PC} = 12.3 Hz), 127.1 (d, J_{PC}= 4.2 Hz), 126.1 (d, J_{PC}= 10.2 Hz), 124.1 (dd, J_{PC}=12.1, 6.1 Hz), 119.2 (d, J_{PC}= 99.4 Hz), 109.4 (dd, J_{CF}= 16.6, 4.3 Hz), 103.5(d, J_{PC}= 8.0 Hz), 61.3 (s), 60.9 (s), 55.6 (s); **^{31}P NMR** (162 MHz, C_6D_6) δ 26.95 (d, J_{PF} = 12.2 Hz); **^{19}F NMR** (376 MHz, C_6D_6) δ -157.88 (ddd, J_{FF} = 19.6 Hz, J_{FF} = 17.5 Hz, J_{HF} = 7.5 Hz), -136.48 (ddd, J_{FF} = 19.6 Hz, J_{FF} = 5.5 Hz, J_{HF} = 10.0 Hz), -116.51 (ddd, J_{FF} = 17.5, J_{PF} = 12.2 Hz, J_{FF} = 5.5 Hz); **^{19}F{^{1}H} NMR** (376 MHz, C_6D_6) δ -157.88 (dd, J_{FF} = 19.6 Hz, J_{FF} = 17.5 Hz), -136.48 (dd, J_{FF} = 19.6 Hz, J_{FF} =5.5 Hz), -116.51 (ddd, J_{FF} = 17.5 Hz, J_{PF} =12.2 Hz, J_{FF} = 5.5 Hz); **HRMS** (TOF MS ES+): calcd. for $C_{29}H_{22}O_4PF_3$ [M+H$^+$] 523.1288, found 523.1286.

(6,7-Difluoro-2,3,4-trimethoxyanthr-9-yl) diphenyl phosphine oxide:

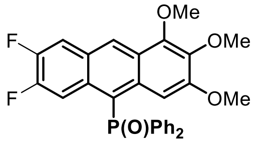

4j: R_f = 0.56 (EtOAc), *n*-hexane:EtOAc (1:2), green crystals, m.p. 176-178 °C; 112 mg, 44% yield: **^{1}H NMR** (400 MHz, C_6D_6) δ 9.47 (dd, *J* = 15.2, 8.3 Hz, 1H), 8.80 (s, 1H), 7.93 (s, 1H), 7.78 (dd, *J* = 12.1, 7.6 Hz, 4H), 6.99 – 6.90 (m, 7H), 3.87 (s, 3H), 3.71 (s, 3H), 3.12 (s, 3H); **^{13}C{^{1}H} NMR** (101 MHz, C_6D_6) δ 154.6 (s), 151.3 (dd, J_{CF} = 251.7, 17.1 Hz), 148.8 (dd, J_{CF} = 250.8, 16.9 Hz), 147.3 (s), 140.5 (s), 136.8 (d, J_{PC} = 102.5 Hz), 133.5 (d, J_{PC} = 8.5 Hz), 133.4 (s), 132.1 (s), 131.8 (d, J_{PC} = 9.8 Hz), 131.5 (d, J_{PC} = 2.8 Hz), 128.9 (d, J_{PC} = 12.0 Hz), 125.1 (d, J_{PC} = 9.8 Hz), 120.4 (d, J_{PC} = 2.8 Hz), 119.9 (dd, J_{PC} = 102.6, 6.8 Hz), 114.2 (d, J_{CF} = 16.1 Hz), 113.8 (dd, J_{CF} = 21.6, 6.1 Hz), 102.6 (d, J_{PC} = 7.5 Hz), 61.2 (s), 60.8 (s), 55.4 (s); **^{31}P NMR** (162 MHz, C_6D_6) δ 28.83; **^{19}F NMR** (376 MHz, C_6D_6) δ -137.45 (ddd, J_{FF} = 20.0, J_{HF} = 15.7, 9.6 Hz), -131.50 (ddd, J_{FF} = 21.0, J_{HF} = 15.1, 8.7 Hz); **^{19}F{^{1}H} NMR** (376 MHz, C_6D_6) δ -137.45 (d, J_{FF} = 21.1 Hz), -131.50 (d, J_{FF} = 20.9 Hz); **HRMS** (TOF MS ES+): calcd. for $C_{29}H_{23}O_4PF_2$ [M+H$^+$] 505.1377, found 505.1380.

Procedure for synthesis of 5:

In a Schlenk tube, dried and filled with argon, **4g** (50 mg, 0.101 mmol) was dissolved in dry toluene (10 mL). Then, trichlorosilane (138 mg, 1.01 mmol) was added dropwise at room temperature and the mixture was stirred in an oil bath at 90 °C for two hours. The volatiles were removed under reduced pressure and the crude product was dissolved in DCM (10 mL) and filtered through the aluminum oxide layer. After evaporation of the solvent, the crude compound containing 27% of silane was obtained as a yellow solid and was further used as such in syntheses of **6-9**.

9-(Diphenylphosphanyl)-5,6,7-trimethoxyanthracene-2-carbonitrile:

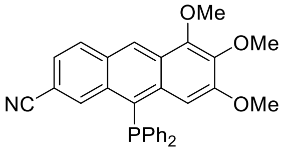

5: Yellow solid, m.p. 193-195 °C; **^{1}H NMR** (400 MHz, C_6D_6) δ 9.83 (d, J = 7.1 Hz, 1H), 8.89 (s, 1H), 7.70 (d, J = 3.5 Hz, 1H), 7.47 (d, J = 8.6 Hz, 1H), 7.42-7.38 (m, 4H), 6.95-6.93 (m, 7H), 3.83 (s, 3H), 3.67 (s, 3H), 3.10 (s, 3H); **^{13}C{^{1}H} NMR** (101 MHz, C_6D_6) δ 154.9 (s), 147.7 (s), 141.6 (s), 136.4 (d, J_{PC} = 22.1 Hz), 136.2 (d, J_{PC} = 14.1 Hz), 135.2 (d, J_{PC} = 5.1 Hz), 134.6 (d, J_{PC} = 36.09 Hz), 131.9 (d, J_{PC} = 18.4 Hz), 131.7 (s), 131.1 (s), 131 (d, J = 5.2 Hz) 128.9 (d, J_{PC} = 5.6 Hz), 127 (s),126.9 (s), 126.2 (s), 124.1 (s), 119.5 (s), 110.6 (s), 103.6 (d, J_{PC}= 15.3 Hz), 61.2 (s), 60.7 (s), 55.4 (s); **^{31}P NMR** (162 MHz, CD_2Cl_2) δ -24.21.

Procedures for synthesis of 6 and 7:

The compound **5** (50 mg, 0.105 mmol) was treated with sublimed sulfur (15 mg, 0.467 mmol) in toluene (7 mL) at reflux under an argon atmosphere. Once the compound **5** was consumed (checked with TLC), the solvent was evaporated, and the crude mixture was separated using silica-gel flash chromatography with hexane/EtOAc as eluents (2:1 v/v) to afford **6** (40 mg) as an orange solid (78% from **4g**). When elemental selenium (33 mg, 0.418 mmol) was used, instead of sulfur, **7** (39 mg) was obtained as an orange solid in 70% yield from **4g**.

9-(Diphenylphosphorothioyl)-5,6,7-trimethoxyanthracene-2-carbonitrile:

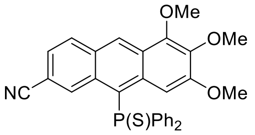

6: R_f = 0.86 (EtOAc), *n*-hexane:EtOAc (1:1), orange solid, m.p. 156-158 °C: 40 mg, 78% yield; **^{1}H NMR** (400 MHz, CD_2Cl_2) δ 8.94 (s, 1H), 8.20 (s, 1H), 8.11 (dd, J = 8.7, 2.0 Hz, 1H), 7.85-7.80 (m, 4H), 7.48-7.44 (m, 1H), 7.41-7.36 (m, 6H), 7.08 (s, 1H), 4.14 (s, 3H), 3.92 (s, 3H), 3.23 (s, 3H); **^{13}C{^{1}H} NMR** (101 MHz, CD_2Cl_2) δ 154.7 (s), 147.2 (s), 141.2 (s), 136.9 (d, J_{PC} = 82.3 Hz), 133.5 (d, J_{PC} = 9.7 Hz), 132.5 (d, J_{PC} = 7.9 Hz), 131.5 (d, J_{PC} = 3.0 Hz), 131.3 (d, J_{PC} = 10.1 Hz), 130.9 (d, J_{PC} = 10.2 Hz), 129.2 (d, J_{PC} = 12.4 Hz), 127.9 (d, J_{PC} = 2.8 Hz),127.4 (d, J_{PC} = 81.2 Hz), 127.1 (s), 126.9 (s), 123.9 (s), 122 (d, J_{PC} = 87.4 Hz), 119.1 (s), 109.1 (s), 102.9 (d, J_{PC} = 10.8 Hz), 62.1 (s), 61.4 (s), 55.9 (s); **^{31}P NMR** (162 MHz, CD_2Cl_2) δ 33.92; **HRMS** (TOF MS ES+): calcd. for $C_{30}H_{25}O_3PNS$ [M+H$^+$] 510.1295, found 510.1296.

9-(Diphenylphosphoroselenoyl)-5,6,7-trimethoxyanthracene-2-carbonitrile:

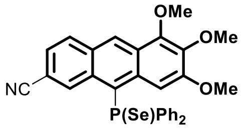

7: R_f = 0.83 (EtOAc), *n*-hexane:EtOAc (1:1), orange solid, m.p. 164-166 °C; 39 mg, 70% yield: **^{1}H NMR** (400 MHz, C_6D_6) δ 8.79 (s, 1H), 8.45 (s, 1H), 7.95- 7.89 (m, 4H), 7.51 (s, 1H), 7.36 (dd, J = 8.6, 2.0 Hz, 1H), 6.85- 6.80 (m, 6H), 6.78 (d, J = 1.4 Hz, 1H), 3.87 (s, 3H), 3.68 (s, 3H), 3.01 (s, 3H); **^{13}C{^{1}H} NMR** (101 MHz, C_6D_6) δ 154 (s), 146.8 (s), 140.9 (s), 135.2 (d, J_{PC} = 73.3 Hz), 133.1 (d, J_{PC} = 9.5 Hz), 132.1 (d, J_{PC} = 7.6 Hz), 131.6 (d, J_{PC} = 10.8 Hz), 130.7 (d, J_{PC} = 3.1 Hz), 130.5 (d, J_{PC} = 10.3 Hz), 130.3 (s), 128.6 (d, J_{PC} = 77.1 Hz), 128.5 (d, J_{PC} = 12.6 Hz), 126.8 (d, J_{PC} = 3.5 Hz), 126.6 (d, J_{PC} = 10.5 Hz), 123.4 (s), 121.2 (d, J_{PC} = 78.8 Hz), 118.4 (s), 109.1 (s), 103.4 (d, J_{PC} = 11.4 Hz), 61 (s), 60.5 (s), 55.1 (s); **^{31}P NMR** (162 MHz, CD_2Cl_2) δ 25.25 (d, $^1J_{PSe}$ = 754 Hz); ^{77}Se NMR (76 MHz, CD_2Cl_2) δ -289.76 (d, $^1J_{SeP}$ = 738.9 Hz); ^{77}Se

NMR (76 MHz, C_6D_6) δ -291.02 (d, $^1J_{SeP}$ = 754.5 Hz); **HRMS** (TOF MS ES+): calcd. for $C_{30}H_{25}O_3PNSe$ [M+H$^+$] 557.0660, found 557.0686.

Procedure for synthesis of the gold(I) complex 8:

The compound **5** (20 mg) was dissolved in CH_2Cl_2 (5 mL) and treated with [Au(tht)Cl] (12 mg, 0.042 mmol) at room temperature under argon atmosphere. The resulting mixture was stirred for 2h. After 2h, the volatiles were removed under vacuum. The crude mixture was re-dissolved in CH_2Cl_2 and filtered through the aluminum oxide layer, and the product was obtained as a yellow solid (25 mg, 90% from **4g**).

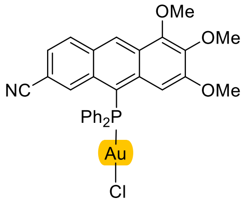

8: yellow solid, 25 mg, 90% yield: **^{1}H NMR** (400 MHz, CD_2Cl_2) δ 9.02 (s, 1H), 8.15 (dd, J = 8.7, 1.7 Hz, 1H), 8.06 (s, 1H), 7.71-7.65 (m, 4H), 7.60 (dd, J = 7.4, 2.0 Hz, 1H), 7.58-7.55 (m, 2H), 7.53-7.48 (m, 4H), 7.44 (dd, J = 8.6, 1.4 Hz, 1H), 4.17 (s, 3H), 3.97 (s, 3H), 3.62 (s, 3H); **^{13}C{^{1}H} NMR** (101 MHz, CD_2Cl_2) δ 155.8 (s), 147.5 (s), 141.3 (s), 134.3 (d, J_{PC} = 10.6 Hz), 134.1 (d, J_{PC} = 14.2 Hz), 132.5 (d, J_{PC} = 2.7 Hz), 132.3 (d, J_{PC} = 10.5 Hz), 131.8 (s), 130.9 (d, J_{PC} = 8.3 Hz), 130.4 (d, J_{PC} = 60.3 Hz), 130.2 (d, J_{PC} = 12.1 Hz), 129.4 (d, J_{PC} = 12.2 Hz), 128.8 (d, J_{PC} = 2.9 Hz), 126.9 (d, J_{PC} = 8.9 Hz), 124.1 (s), 118.9 (s), 116.2 (d, J_{PC} = 58.4 Hz), 109.9 (s), 101.1 (d, J_{PC} = 18.6 Hz), 62.1 (s), 61.5 (s), 56.4 (s); **^{31}P NMR** (162 MHz, CD_2Cl_2) δ 23.39; **HRMS** (TOF MS ES+): calcd. for $C_{30}H_{24}O_3PNAuCl$ [M$^+$] 709.0875, found 709.0888.

Procedure for synthesis of 9:

The compound **5** (50 mg, 0.105 mmol) was treated with iodomethane (60 mg, 0.467 mmol) in toluene (7 mL) at reflux. Once the compound **5** was consumed (checked with TLC), the solvent was evaporated, and the crude mixture was separated using silica-gel flash chromatography with hexane/EtOAc as eluents (2:1 v/v) to afford **9** (22 mg) as an orange solid (72% from **4g**).

5,6,7-Trimethoxyanthracene-2-carbonitrile:

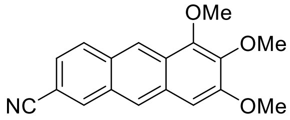

9: R_f = 0.87 (EtOAc), *n*-hexane:EtOAc (1:1), orange solid; m.p. 127-129 °C: 22 mg, 72% yield: **^{1}H NMR** (400 MHz, C_6D_6) δ 8.60 (s, 1H), 7.87 (s, 1H), 7.73 (s, 1H), 7.45 (d, J = 8.7 Hz, 1H), 6.99 (dd, J = 8.6, 1.6 Hz, 1H), 6.66 (s, 1H), 3.86 (s, 3H), 3.79 (s, 3H), 3.43 (s, 3H); **^{13}C{1H} NMR** (101 MHz, C_6D_6) δ 154.6, 147.6, 142.3, 134.7, 130.6, 130.4, 130.2, 129.9, 126.7, 125.6, 124.1, 121.2, 119.6, 109.4, 101.2, 61.1, 60.9, 55.3; **HRMS** (TOF MS ES+): calcd. for $C_{18}H_{16}O_3N$ [M+H$^+$] 294.1116, found 294.1130.

Procedure for synthesis of 10:

In a Schlenk tube, dried and filled with argon, **4h** (40 mg, 0.074 mmol), 2-thienylboronic acid (11 mg, 0.080 mmol), $Pd(PPh_3)_4$ (6 mg, 0.08 equiv.) and K_2CO_3 (28 mg, 2.5 equiv.) were dissolved in a toluene:MeOH (3:1 v/v) solution (4 mL). The reaction mixture was stirred in an oil bath at 80 °C for 36 hours. Then, the solvent was removed in vacuum, the resulting solid was dissolved in EtOAc (10 mL) and washed with water (7×2 mL). After drying with $MgSO_4$

the solvent was removed, and the crude product was purified using flash silica chromatography hexane: EtOAc (1:1 v/v) as eluent. Finally, the product **10** was obtained as a green solid (37 mg) in 94% yield.

(2,3,4-Trimethoxy-6-(thien-2-yl)anthr-9-yl) diphenyl phosphine oxide:

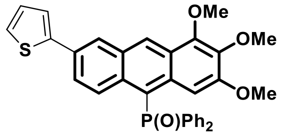

10: R_f = 0.42 (EtOAc), *n*-hexane:EtOAc (1:2), yellow oil; 37 mg 94% yield: **^{1}H NMR** (400 MHz, CD_2Cl_2) δ 8.96 (s, 1H), 8.64 (d, *J* = 9.3 Hz, 1H), 8.26 (s, 1H), 7.73-7.67 (m, 4H), 7.57 (s, 1H), 7.55-7.50 (m, 4H), 7.46- 7.43 (m, 4H), 7.42 (dd, *J* = 3.0, 1.2 Hz, 1H), 7.35 (dd, *J* = 5.1, 1.1 Hz, 1H), 7.13 (dd, *J* = 5.1, 3.6 Hz, 1H), 4.15 (s, 3H), 3.93 (s, 3H), 3.38 (s, 3H); **^{13}C{^{1}H} NMR** (101 MHz, C_6D_6) δ 154.7 (s), 147.5 (s), 144.1 (s), 140.7 (s), 137.6 (d, J_{PC} = 102.0 Hz), 134.7 (dd, J_{PC} = 8.7, 6.0 Hz), 134.3 (d, J_{PC} = 102.56 Hz), 132.4 (d, J_{PC} = 9.6 Hz), 131.9 (d, J_{PC} = 9.8 Hz), 131.5 (d, J_{PC} = 2.8 Hz), 131.2 (d, J_{PC} = 2.8 Hz), 130.8 (d, J_{PC} = 10.7 Hz), 130.4 (s),128.8 (d, *J* = 12.0 Hz), 128.6 (d, *J* = 4.9 Hz), 128.5 (s), 125.9 (d, J_{PC} = 10.9 Hz), 125.4 (s), 125.3 (d, *J* = 2.8 Hz), 124.1 (s), 120 (d, J_{PC} = 98.5 Hz), 103 (d, J_{PC} = 6.8 Hz), 61.3 (s), 60.8 (s), 55.5 (s); **^{31}P NMR** (162 MHz, CD_2Cl_2) δ 29.57; **HRMS** (TOF MS ES+): calcd. for $C_{30}H_{28}O_4PS$ [M+H$^+$] 551.1444, found 551.1446.

3. NMR spectra of diarymethanols, 4a-j and 5-10:

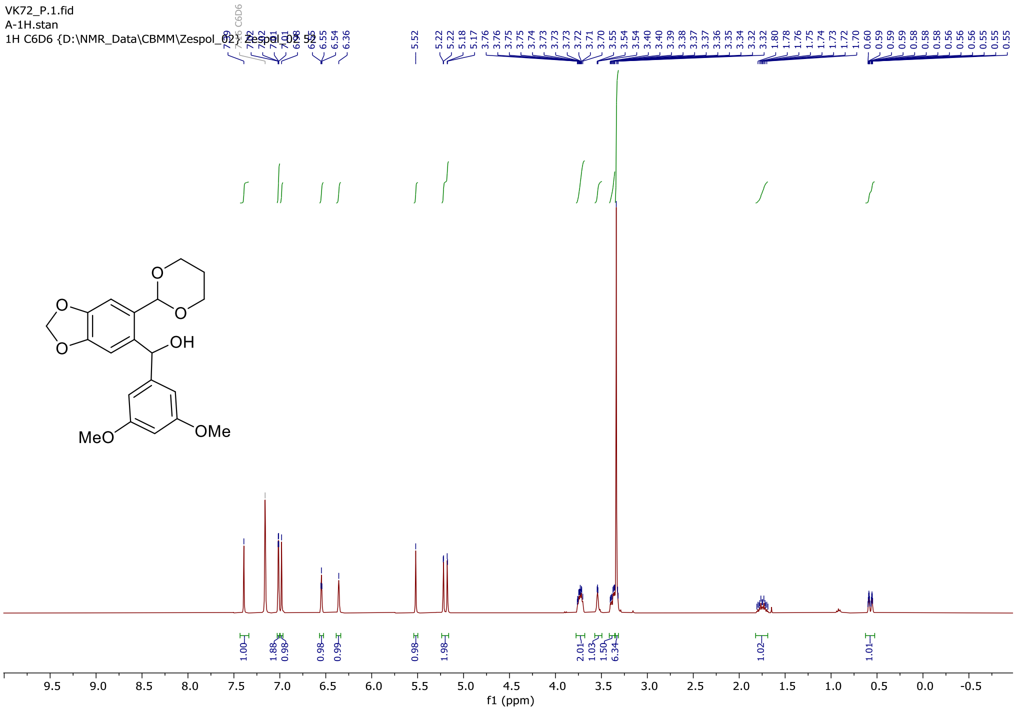

Figure S1.[1]*H NMR of* ***1b****.*

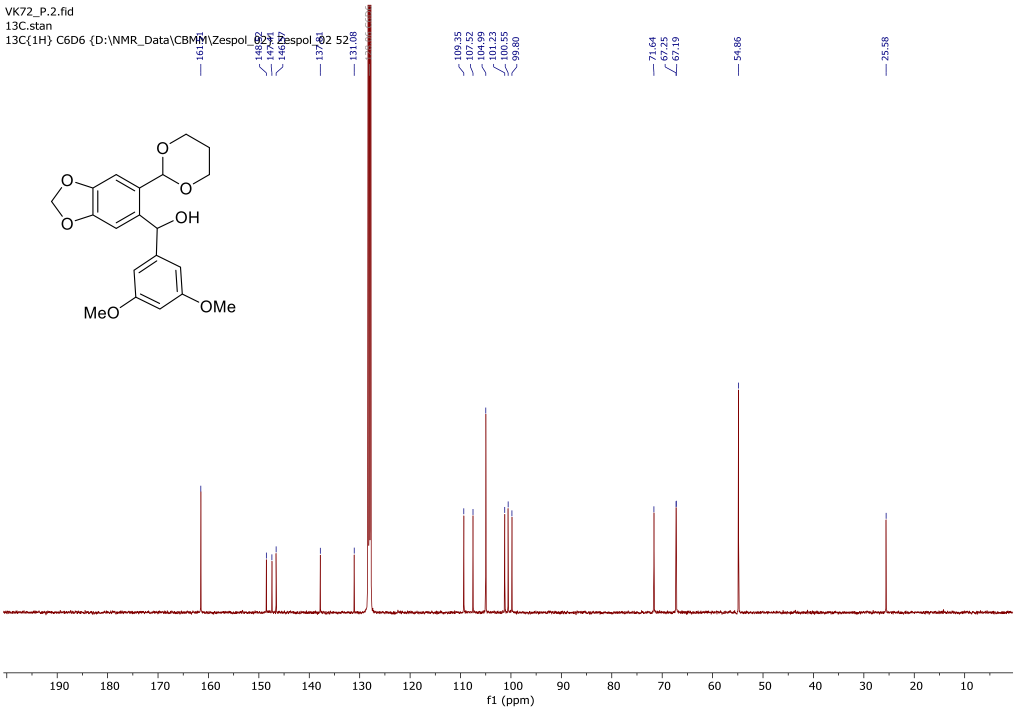

Figure S2.[13]*C{*[1]*H} NMR of* ***1b****.*

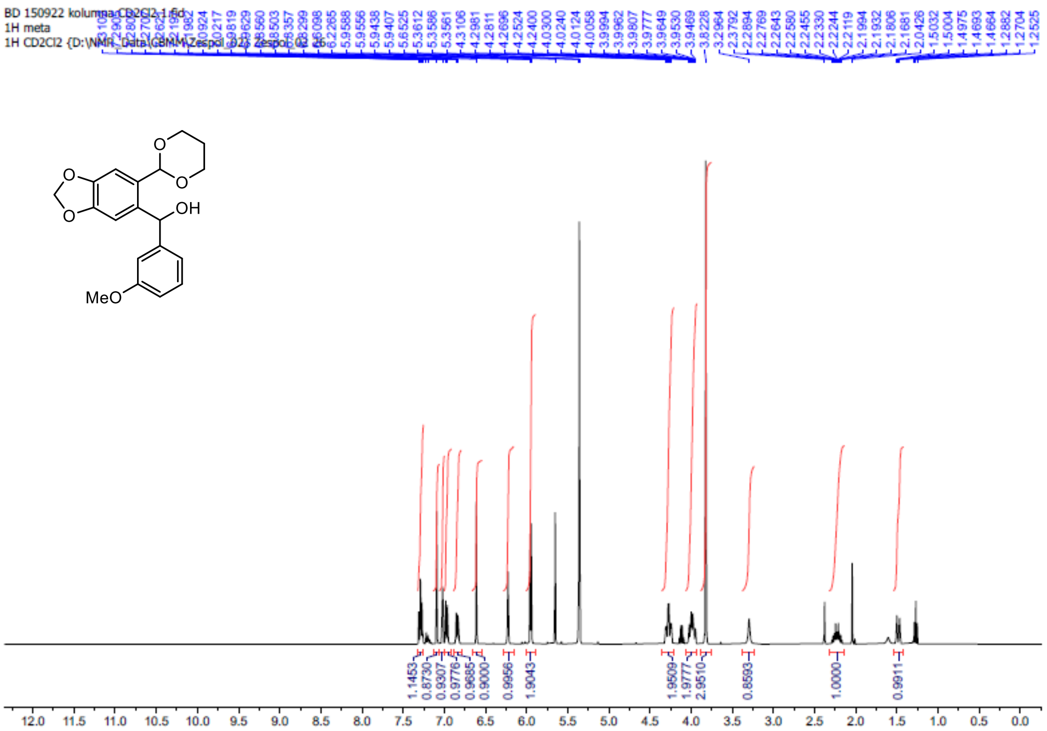

Figure S3.[1]*H NMR of* ***1c****.*

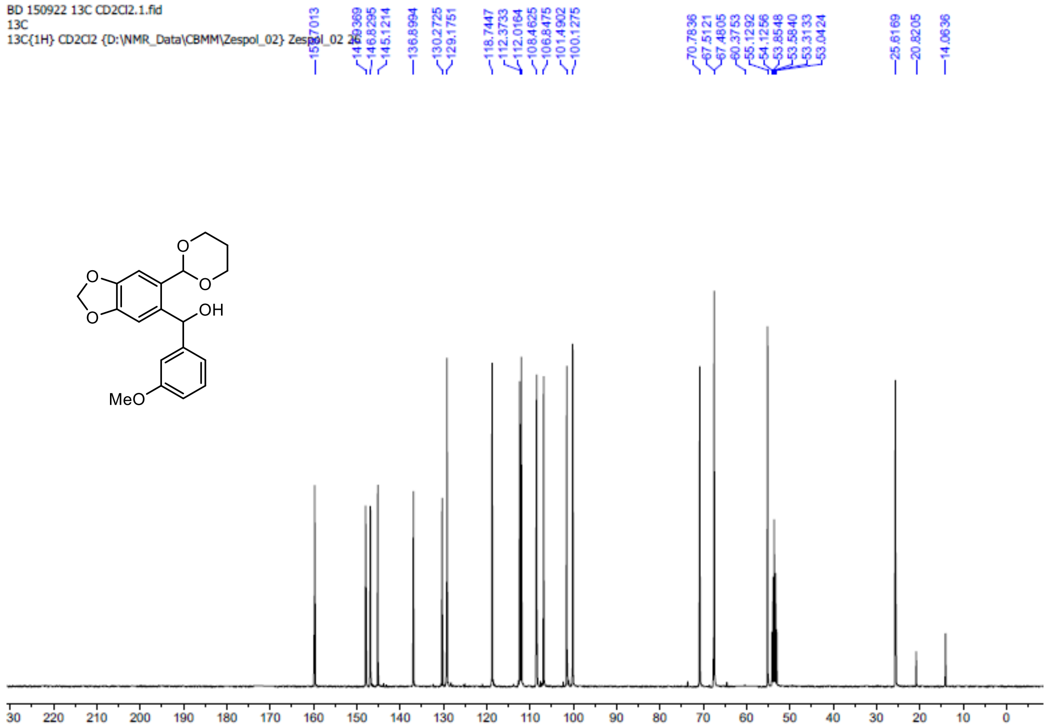

Figure S4.^{13}*C{*^{1}*H} NMR of* ***1c****.*

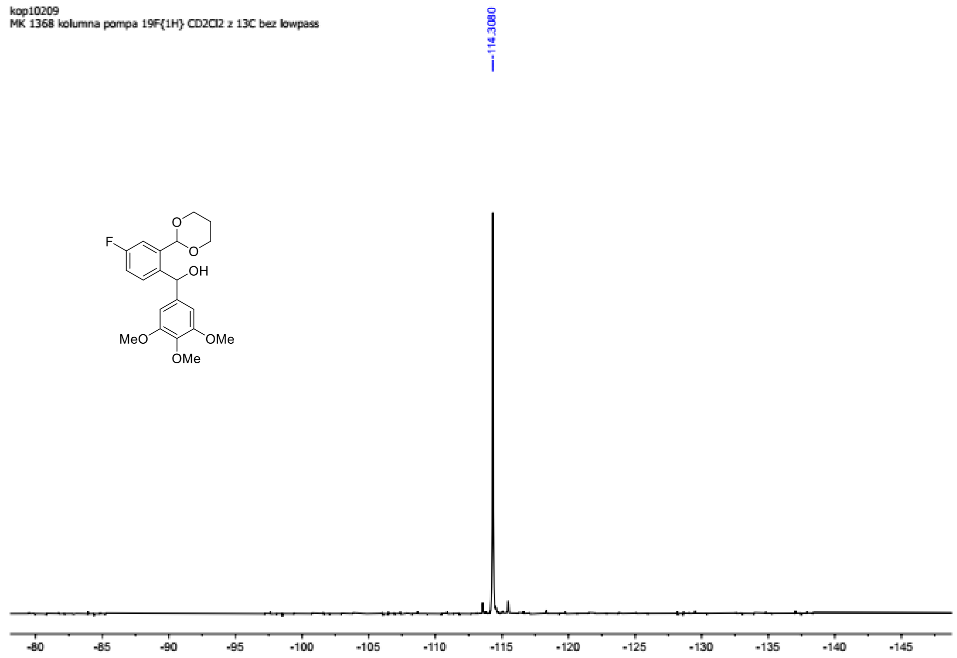

Figure S5. $^{19}F\{^{1}H\}$ *NMR of* ***1e****.*

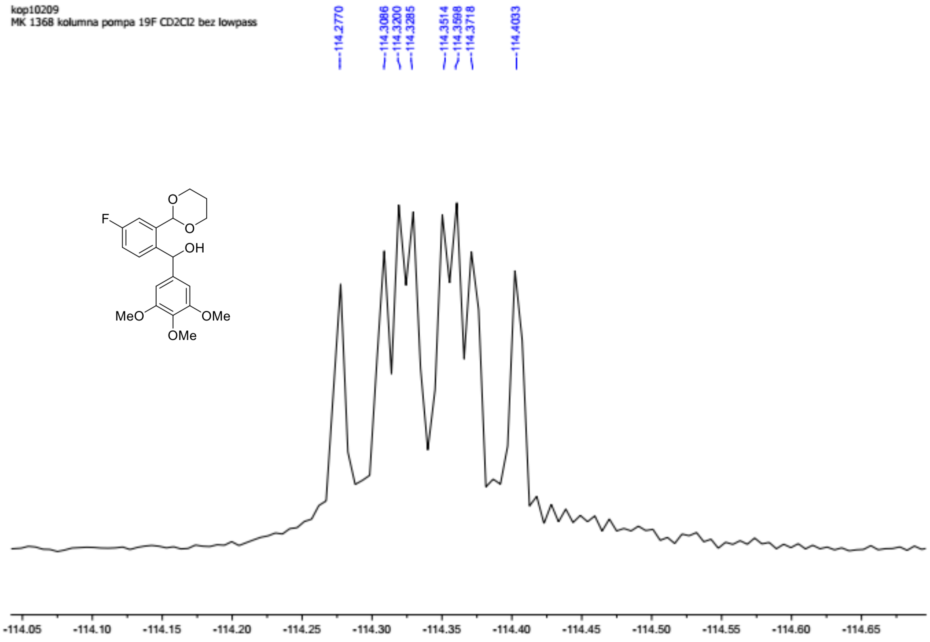

Figure S6. ^{19}F *NMR of* ***1e****.*

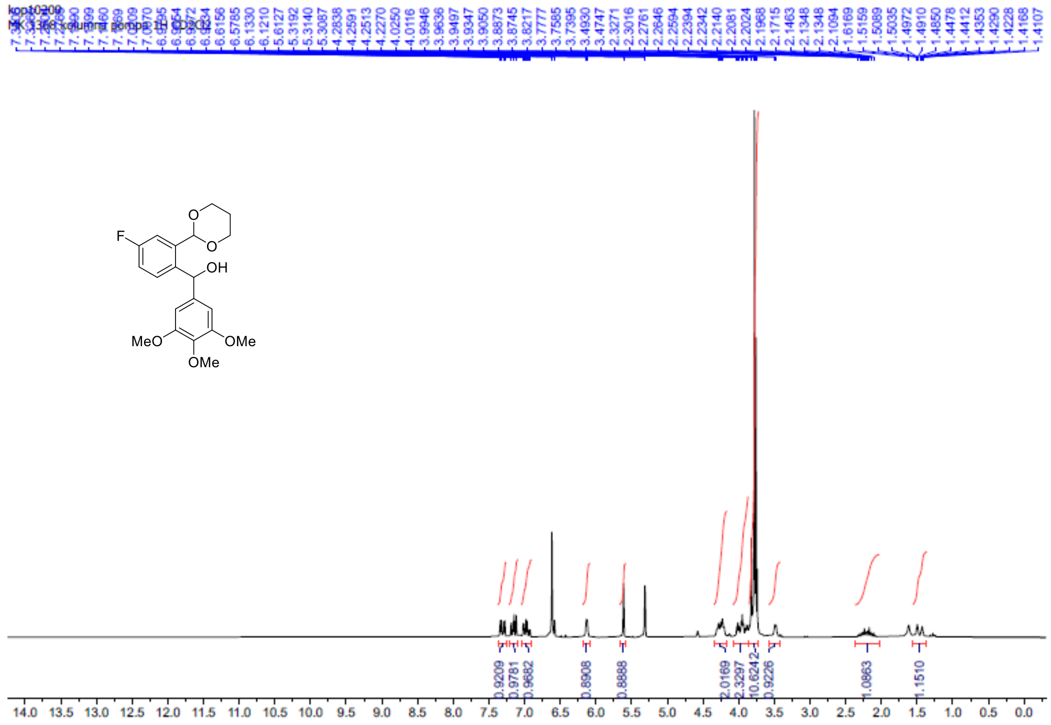

Figure S7. ¹H NMR of **1e**.

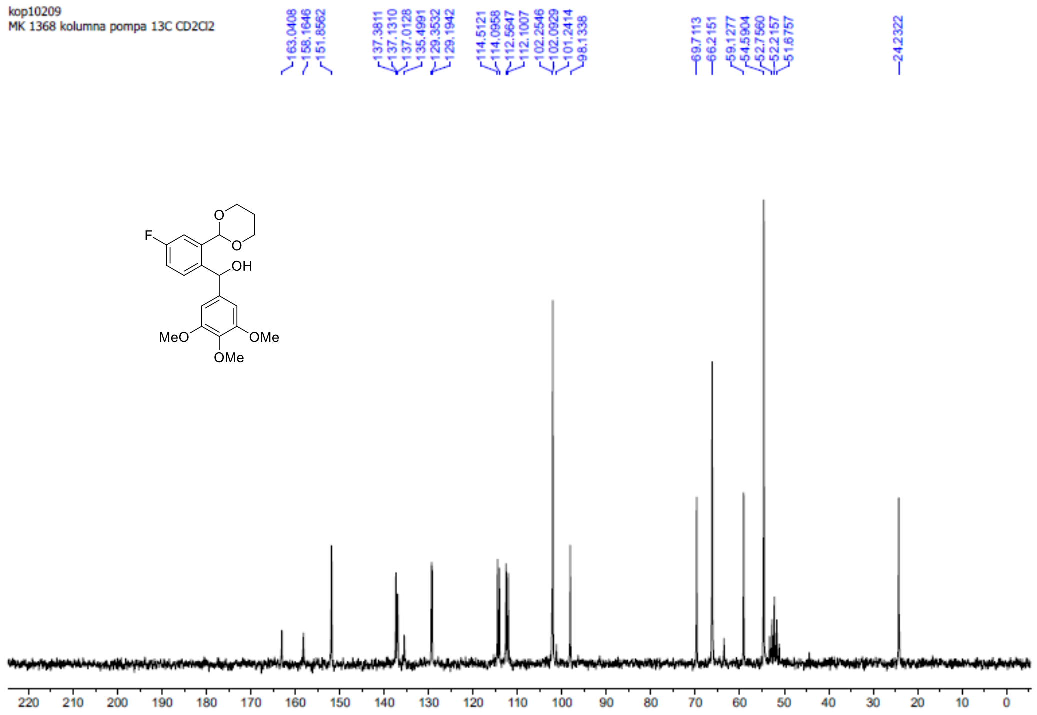

Figure S8. $^{13}C\{^1H\}$ NMR of **1e**.

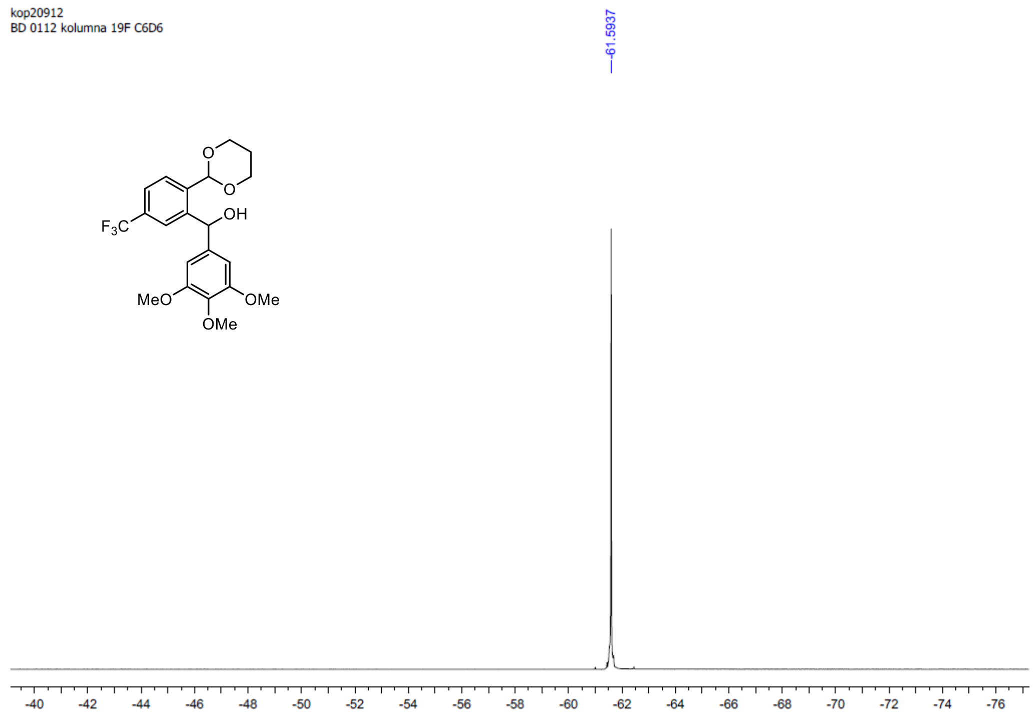

Figure S9. *^{19}F{^{1}H} NMR of* ***1f****.*

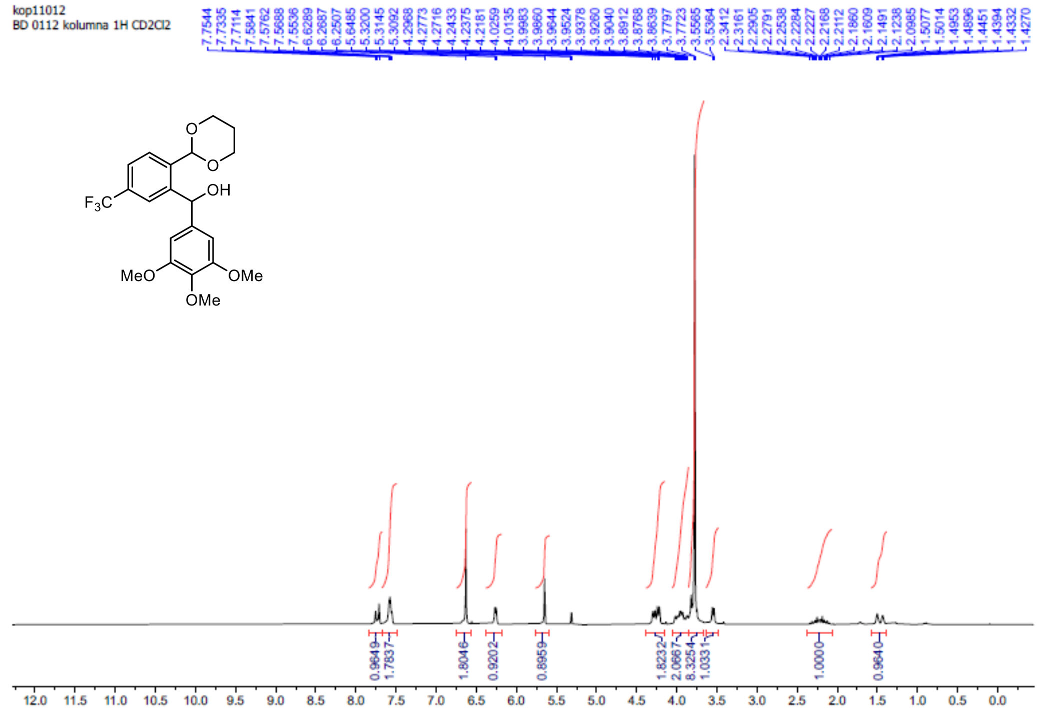

Figure S10. *^{1}H NMR of* ***1f****.*

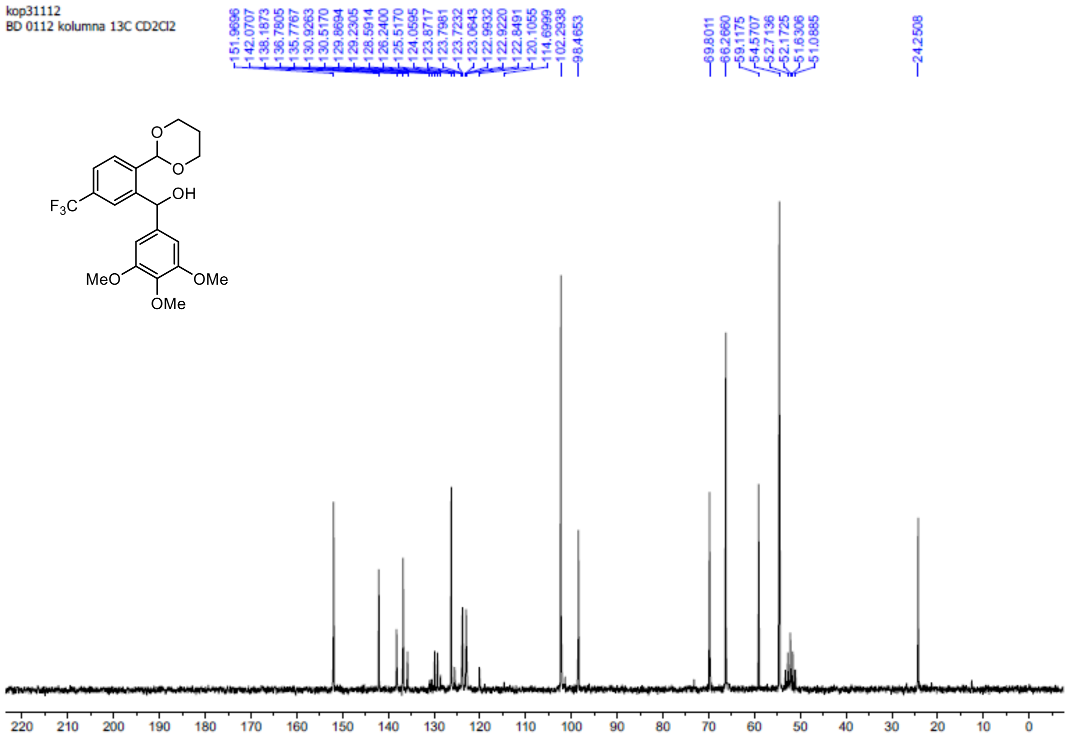

Figure S11.[13]*C{*[1]*H} NMR of **1f**.*

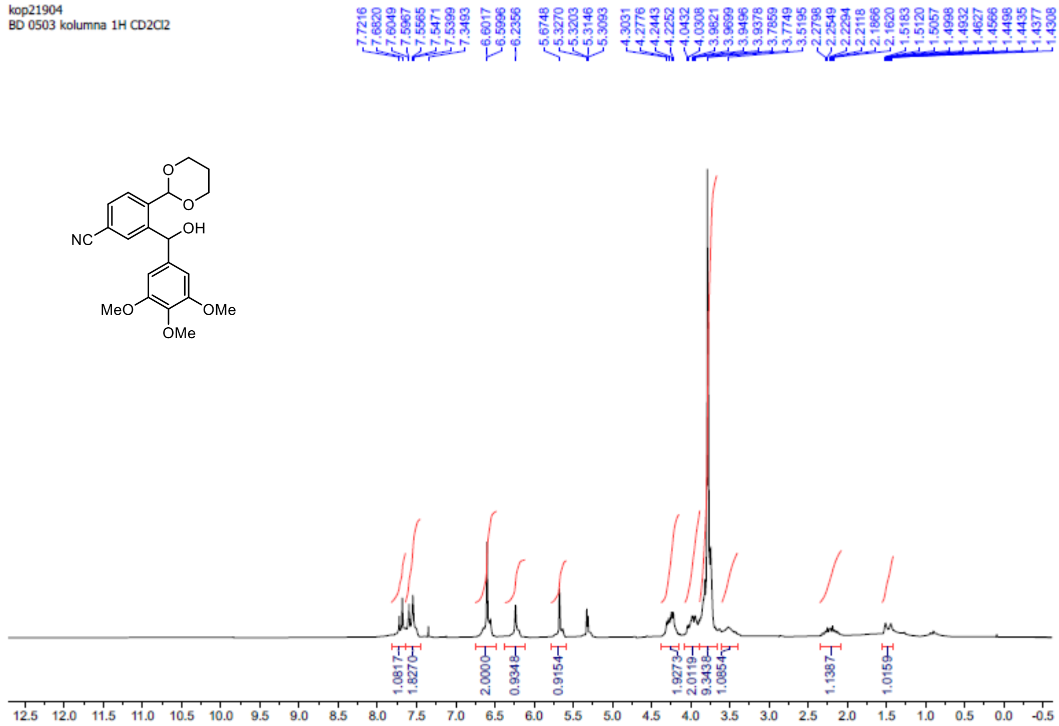

Figure S12.[1]*H NMR of **1g**.*

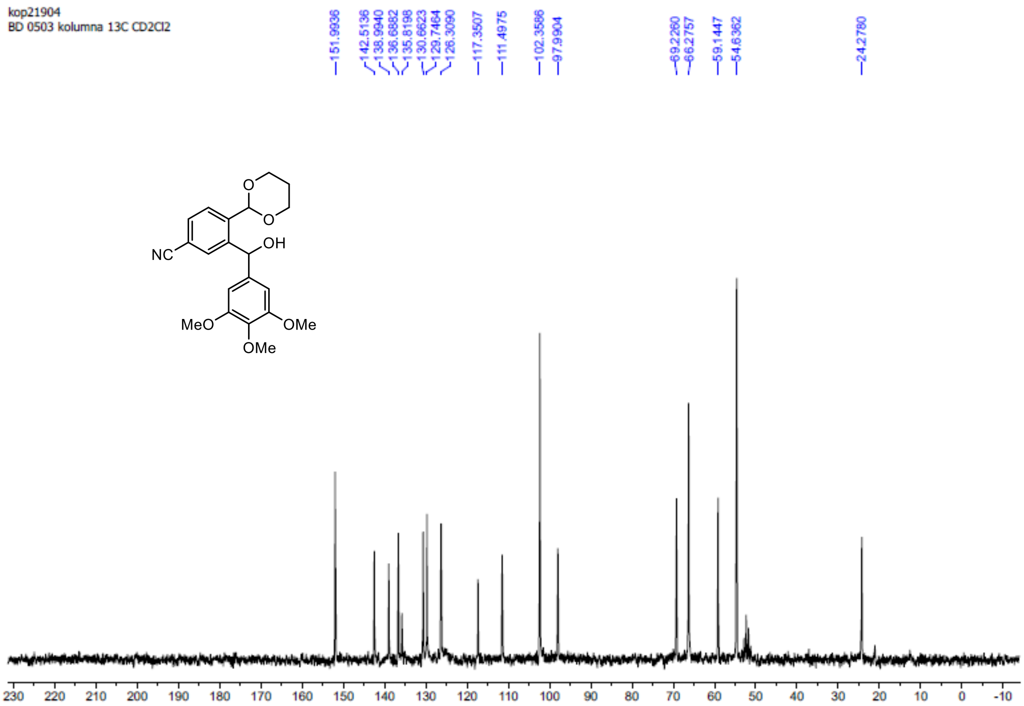

Figure S13. $^{13}C\{^{1}H\}$ *NMR of* ***1g****.*

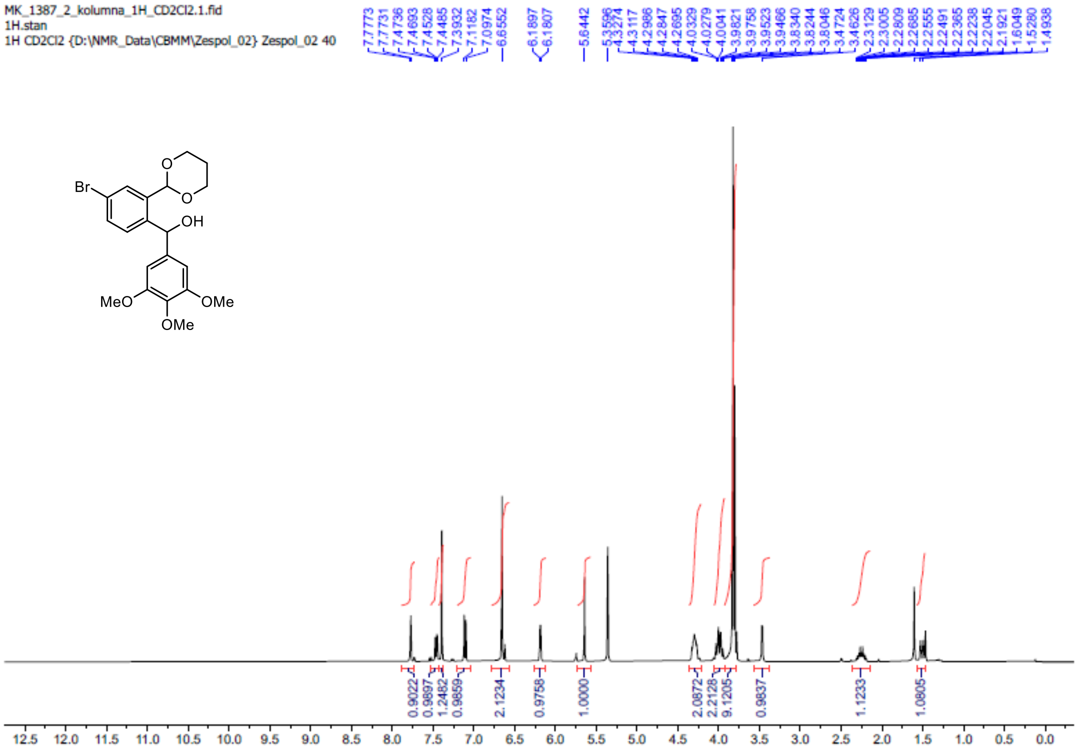

Figure S14. ^{1}H *NMR of* ***1h****.*

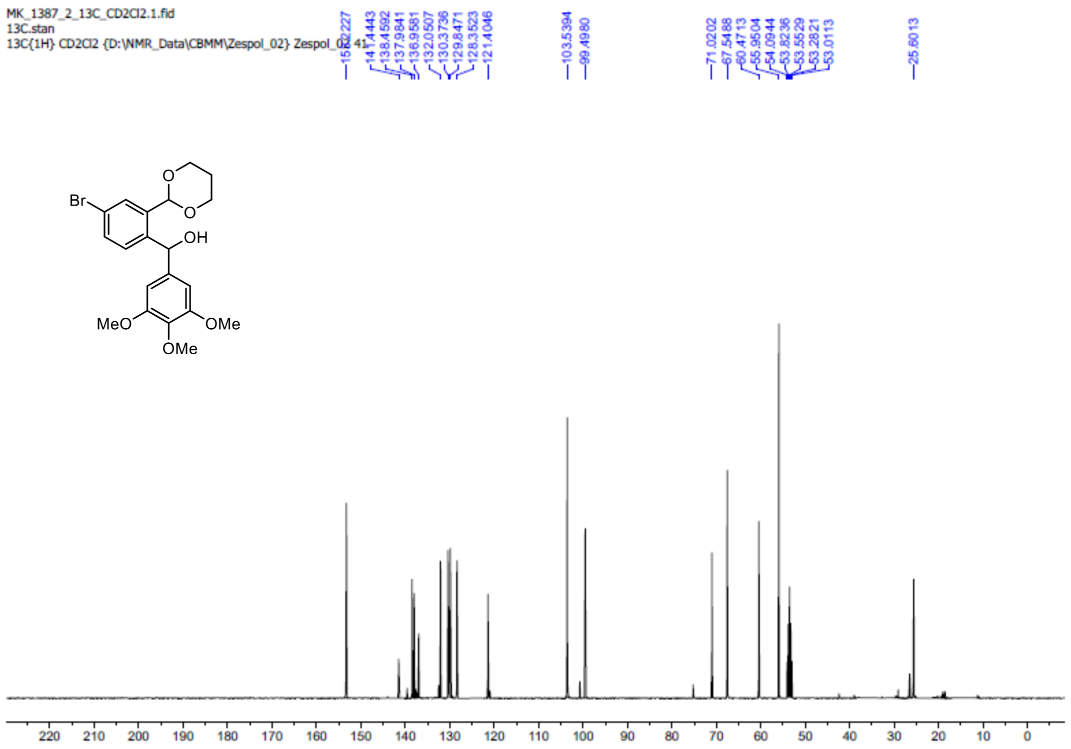

Figure S15. $^{13}C\{^{1}H\}$ *NMR of* ***1h****.*

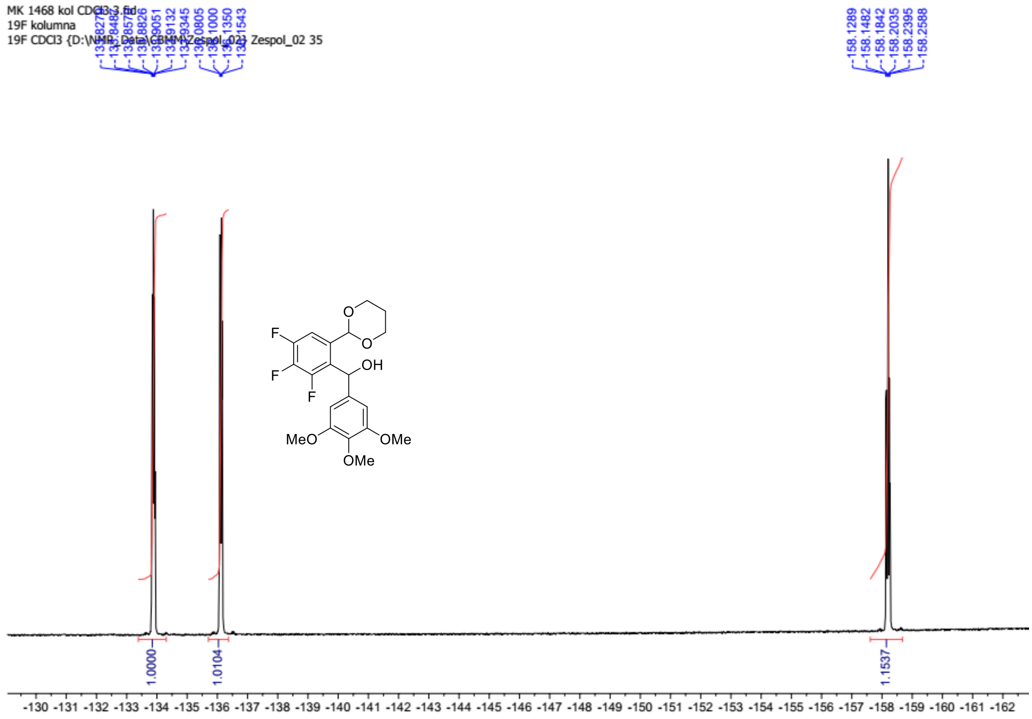

Figure S16. ^{19}F *NMR of* ***1i****.*

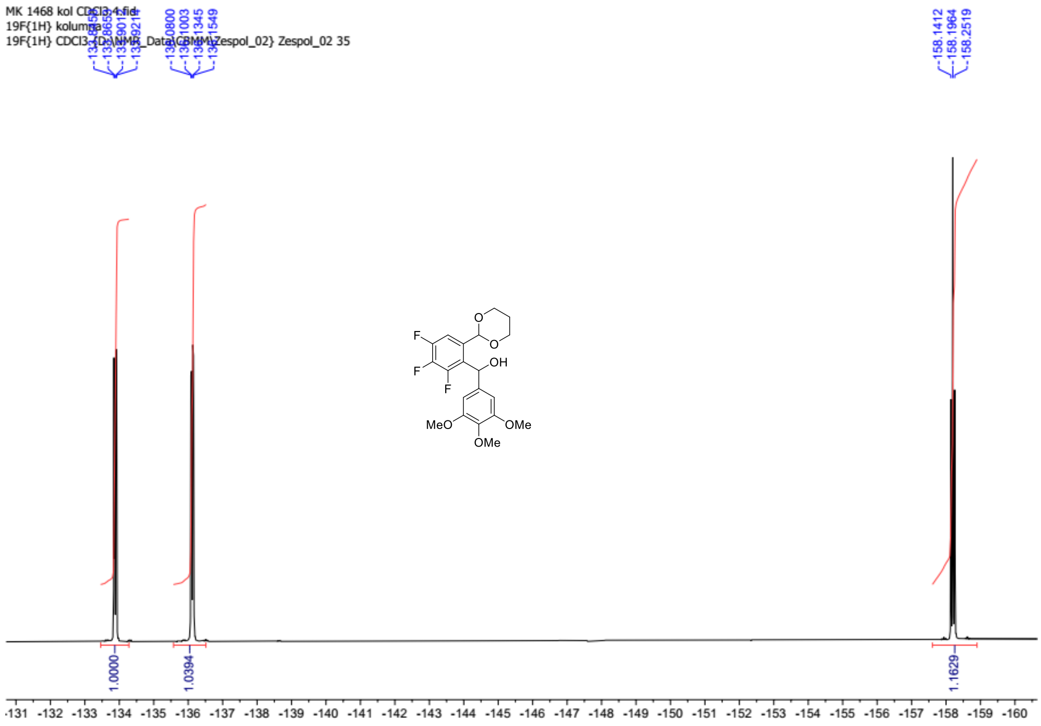

Figure S17.^{19}F{^{1}H} NMR of **1i**.

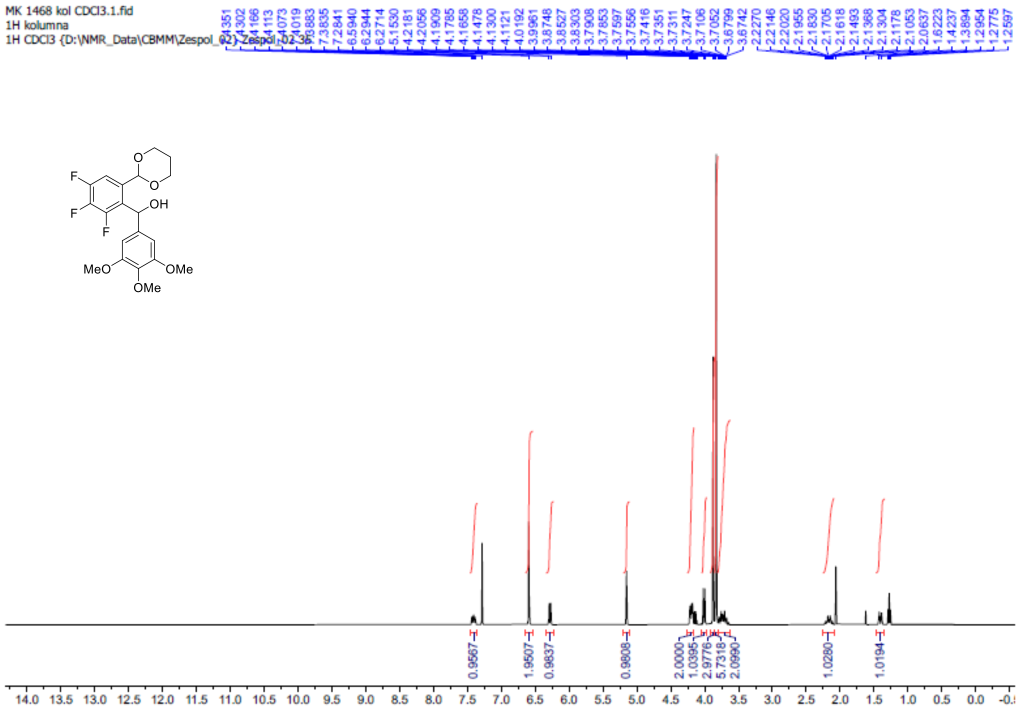

Figure S18.^{1}H NMR of **1i**.

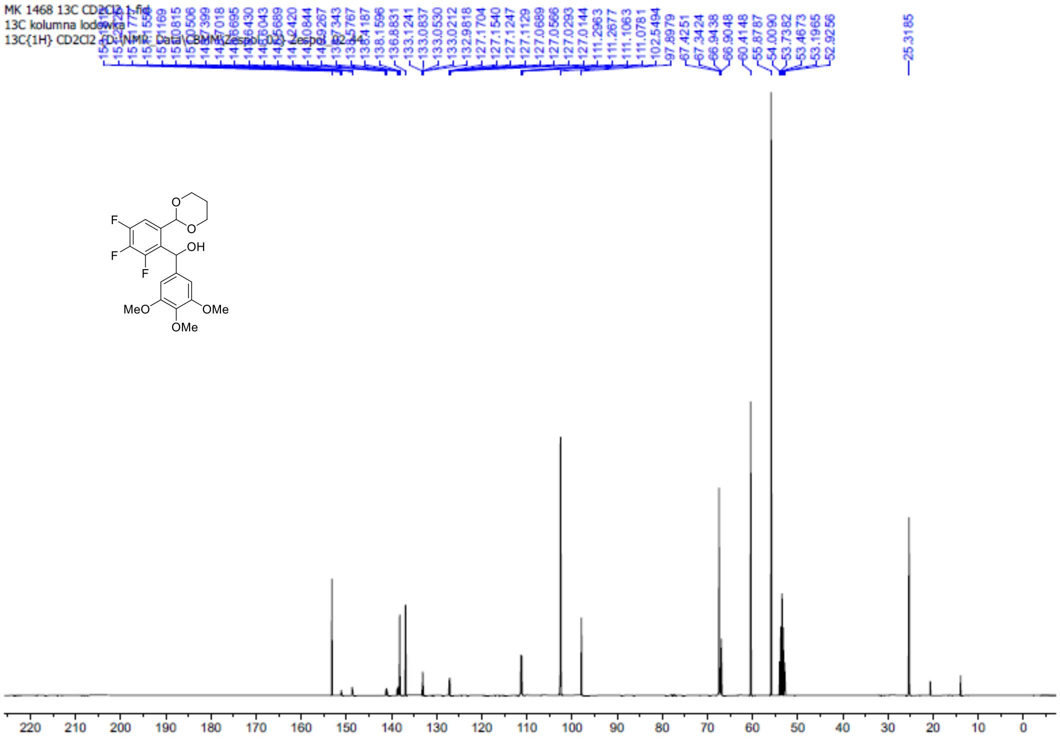

Figure S19.^{13}C{^{1}H} NMR of **1i**.

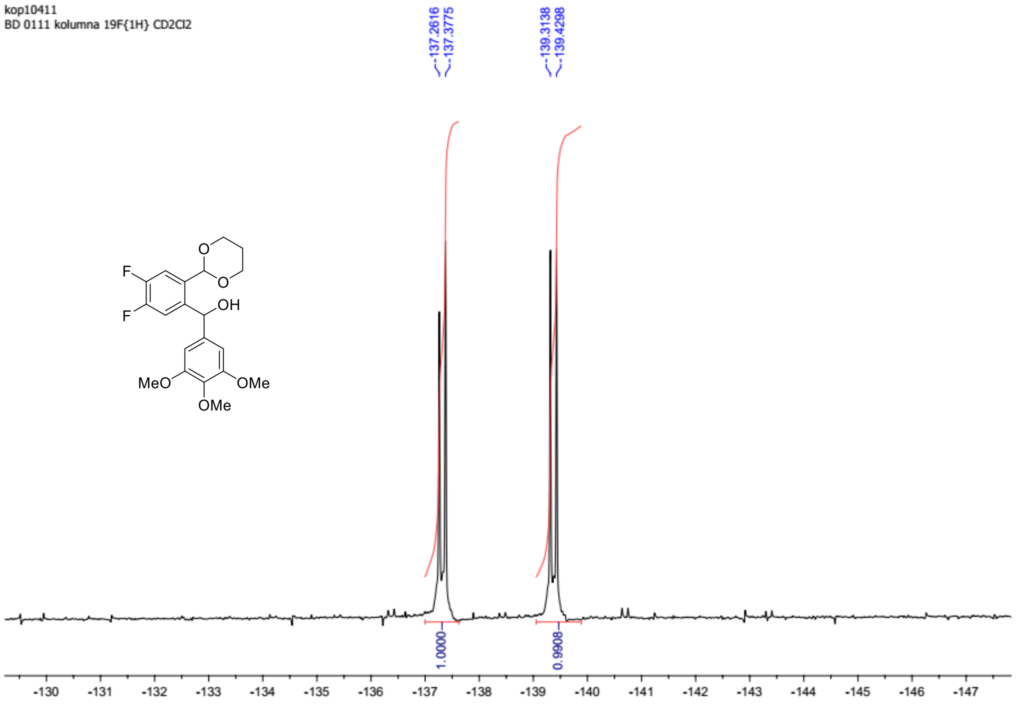

Figure S20.^{19}F{^{1}H} NMR of **1j**.

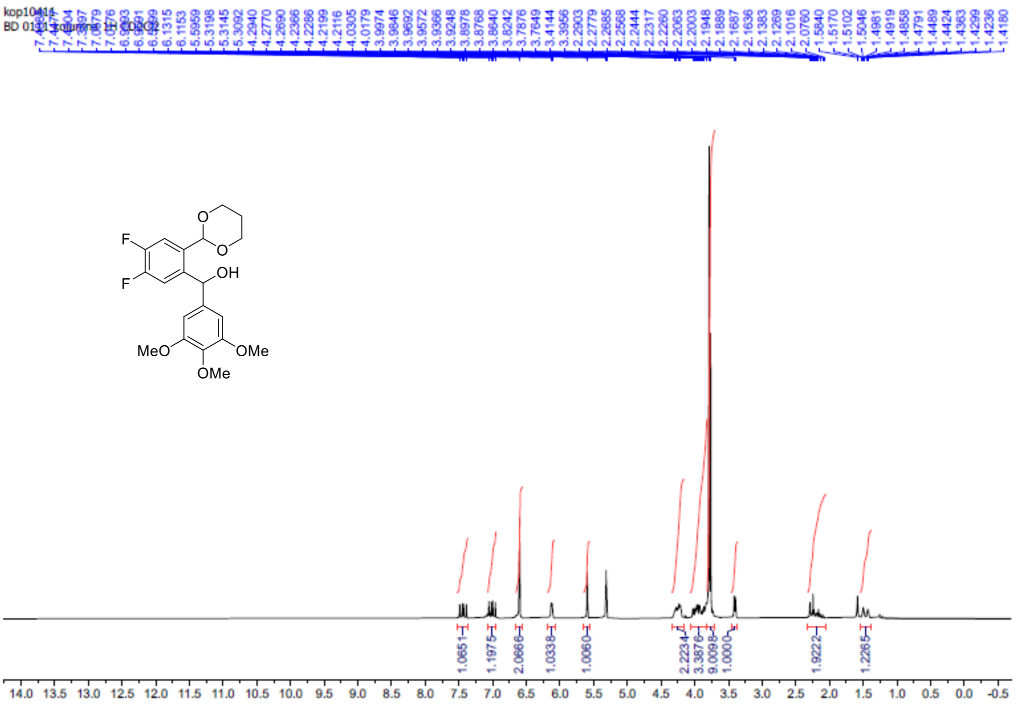

Figure S21. *¹H NMR of* ***1j***.

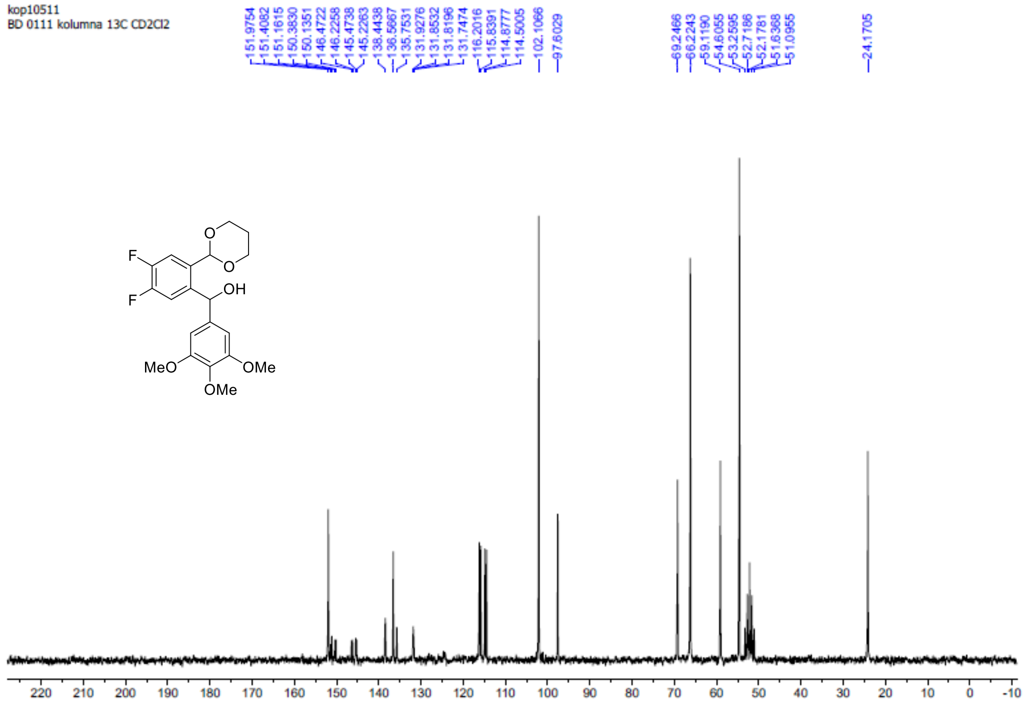

Figure S22. *¹³C{¹H} NMR of* ***1j***.

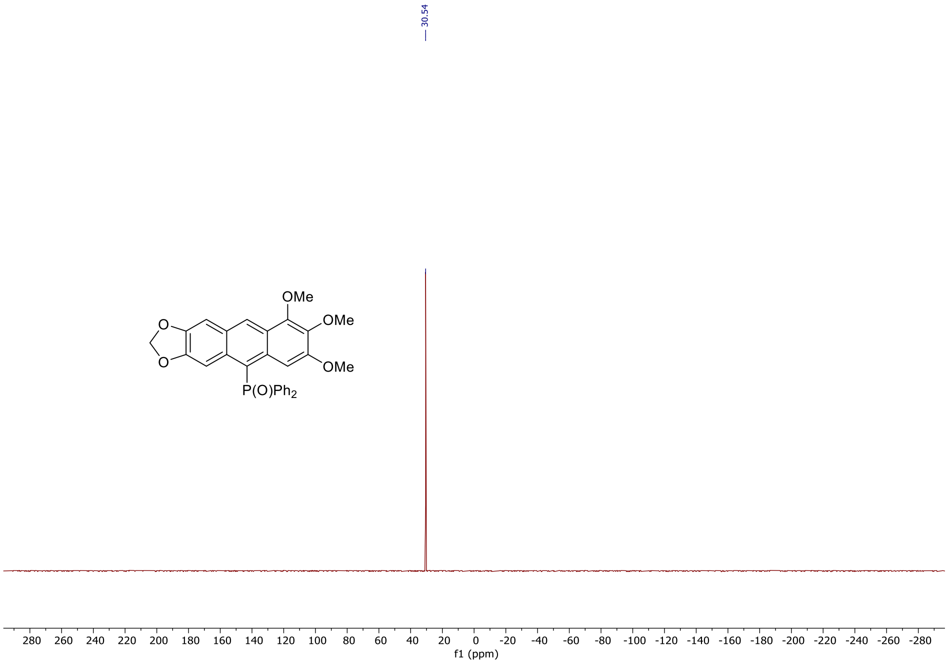

Figure S23. *^{31}P NMR of **4a**.*

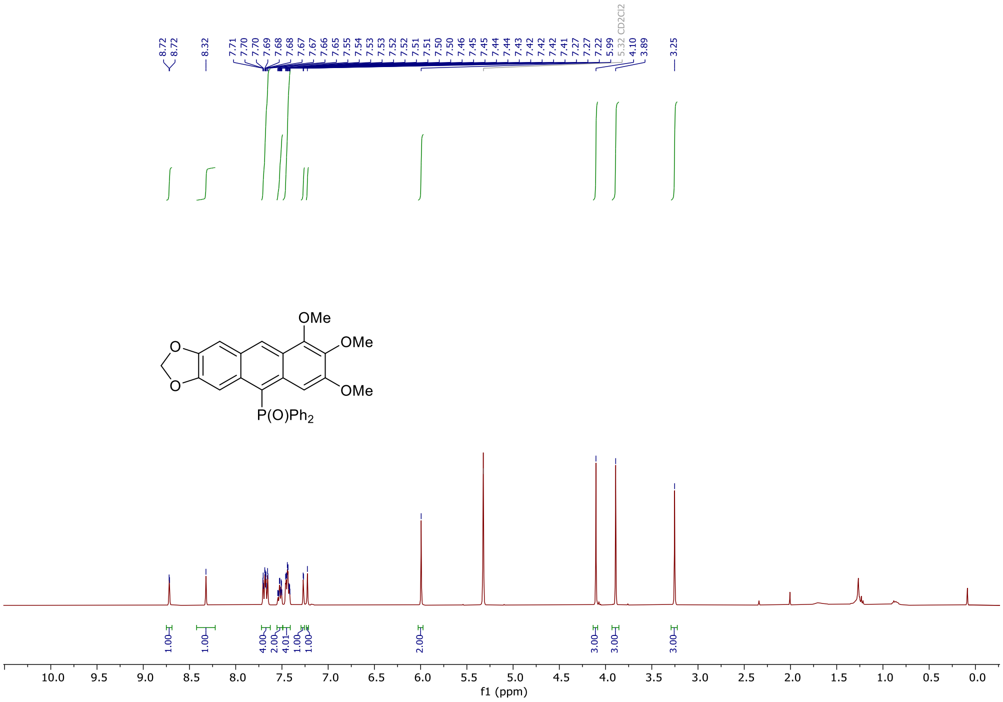

Figure S24. *^{1}H NMR of **4a**.*

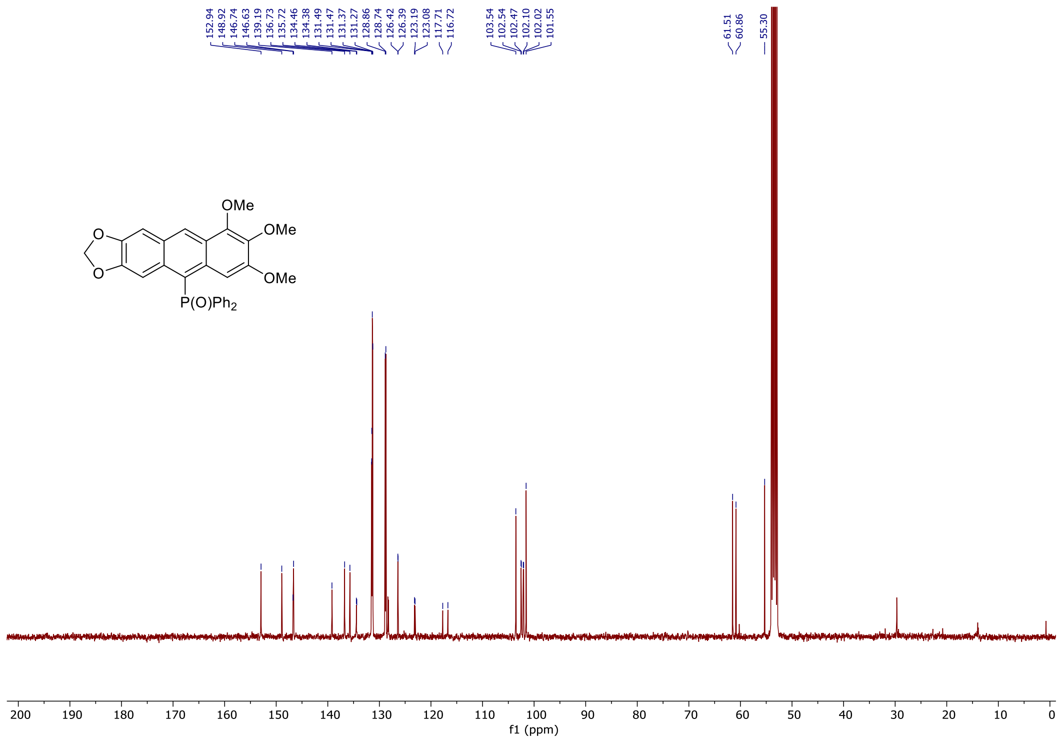

Figure S25. $^{13}C\{^{1}H\}$ NMR of ***4a***.

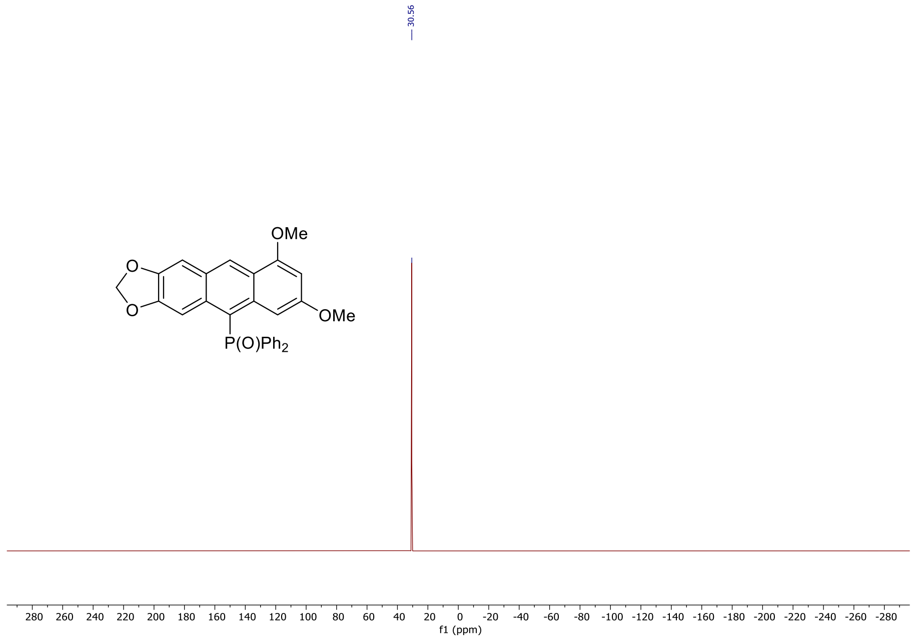

Figure S26. ^{31}P NMR of ***4b***.

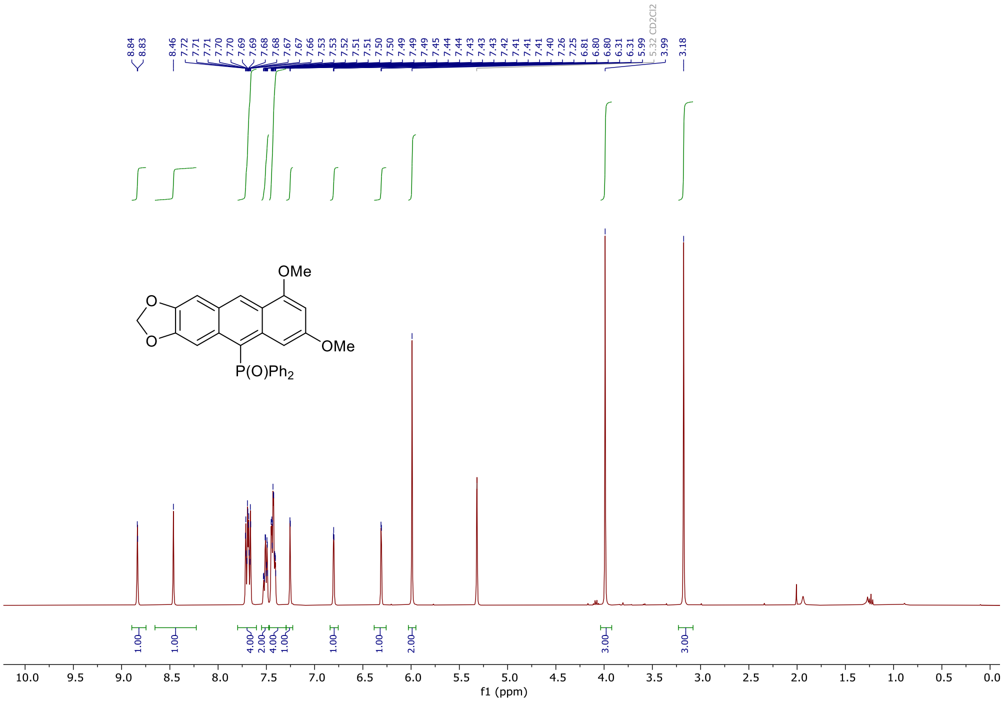

Figure S27. ^{1}H NMR of **4b**.

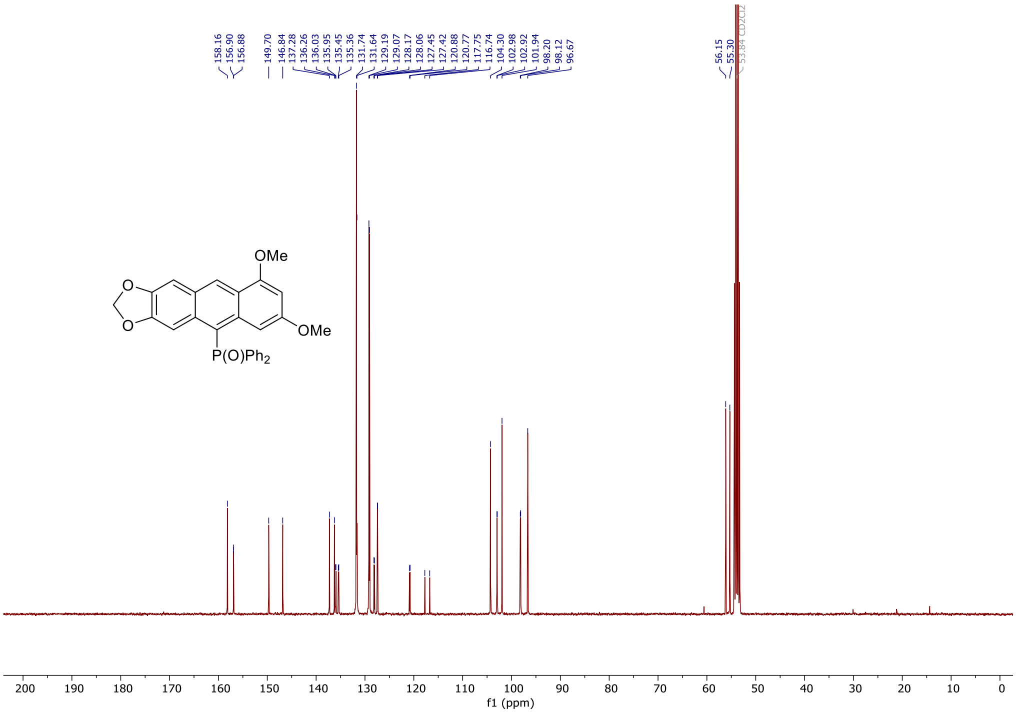

Figure S28. ^{13}C{^{1}H} NMR of **4b**.

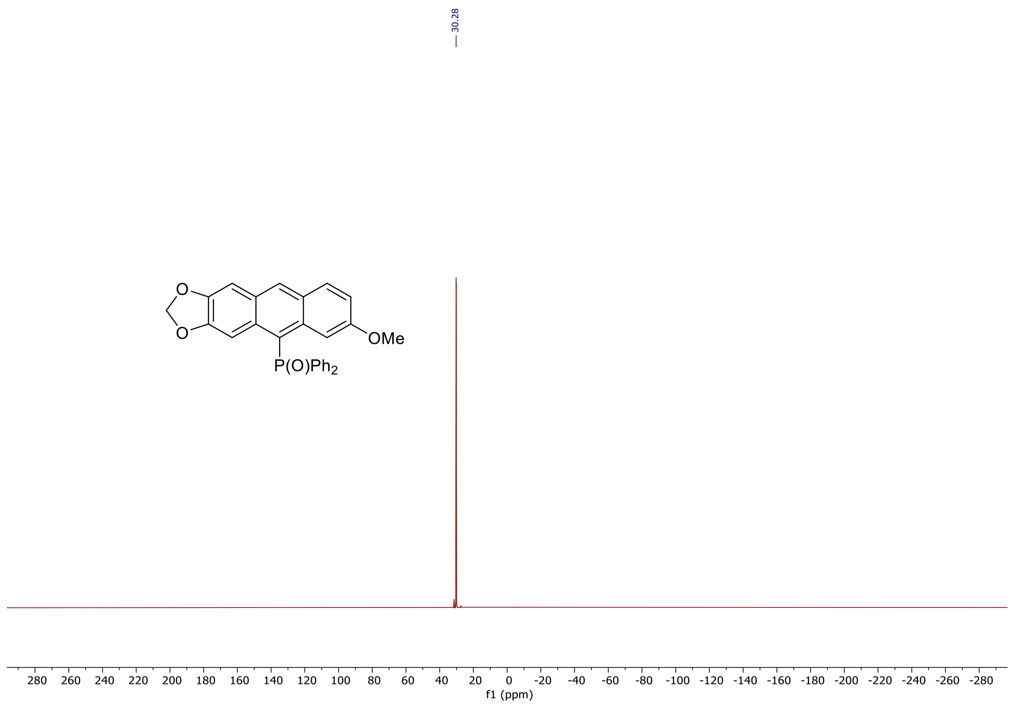

Figure S29. *³¹P NMR of* ***4c****.*

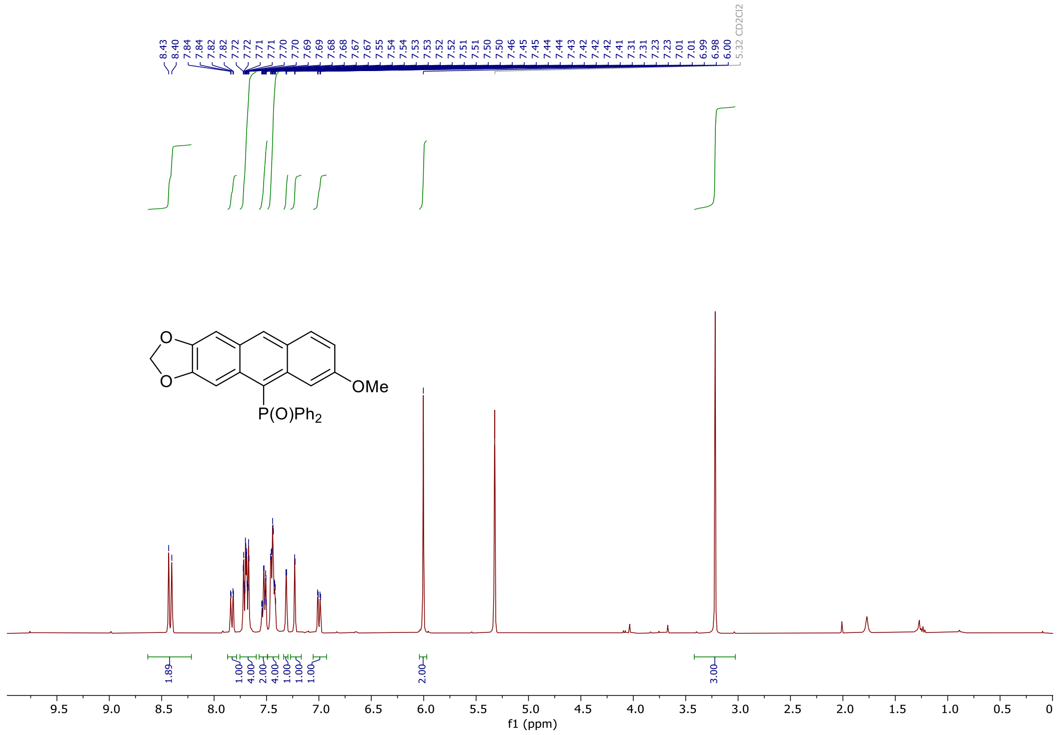

Figure S30. *¹H NMR of* ***4c****.*

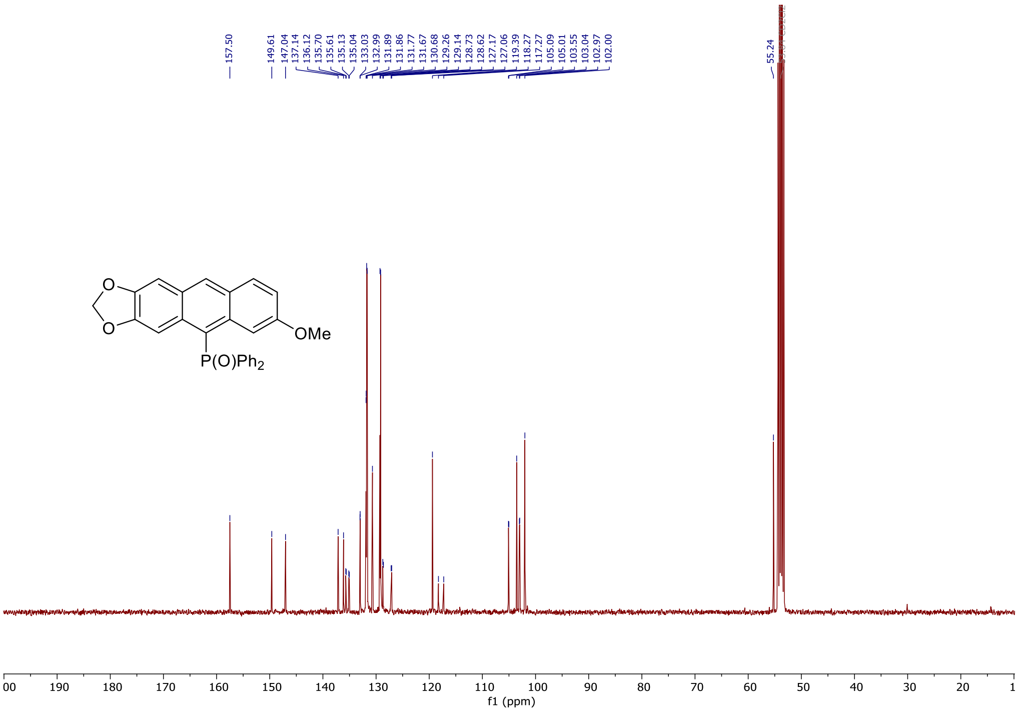

Figure S31. *$^{13}C\{^{1}H\}$ NMR of **4c**.*

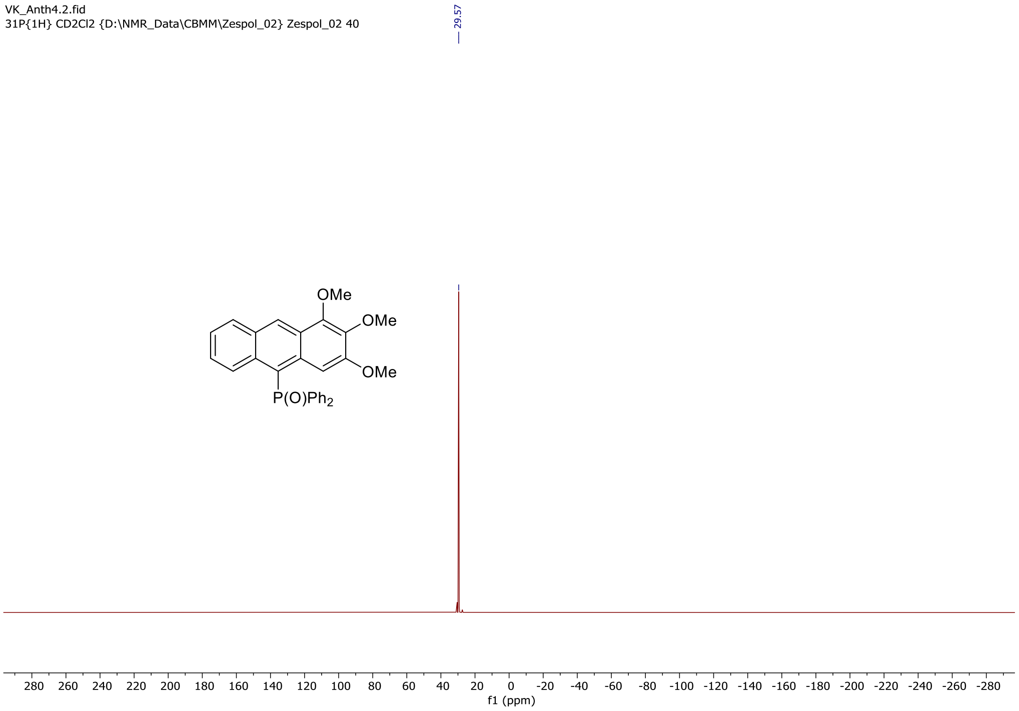

Figure S32. *^{31}P NMR of **4d**.*

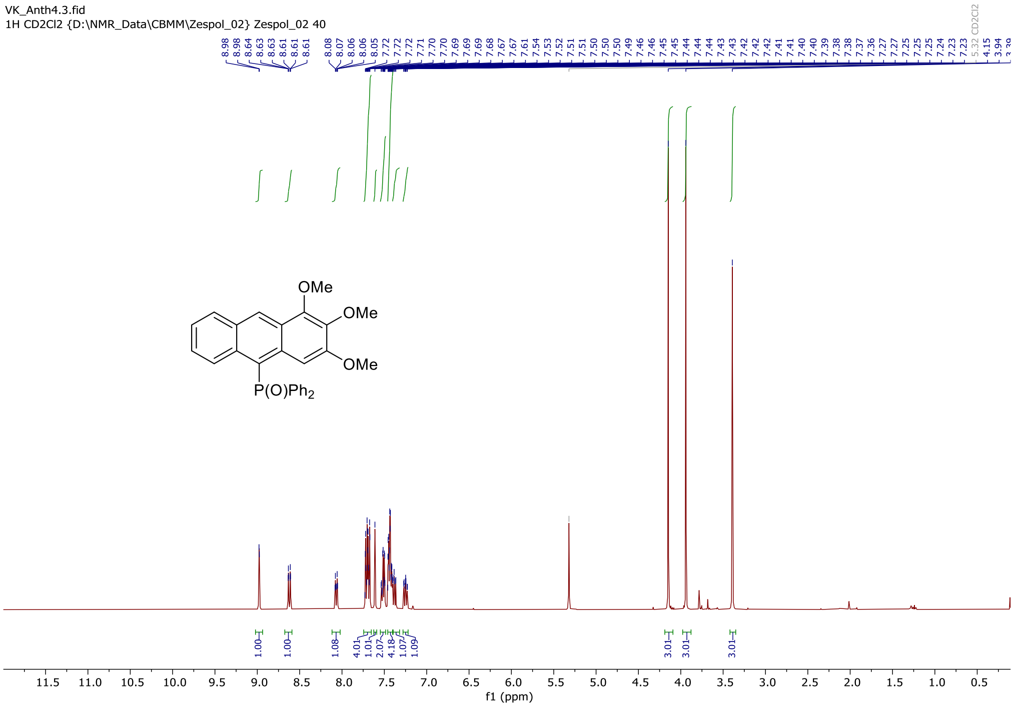

Figure S33. ^{1}H *NMR of* ***4d***.

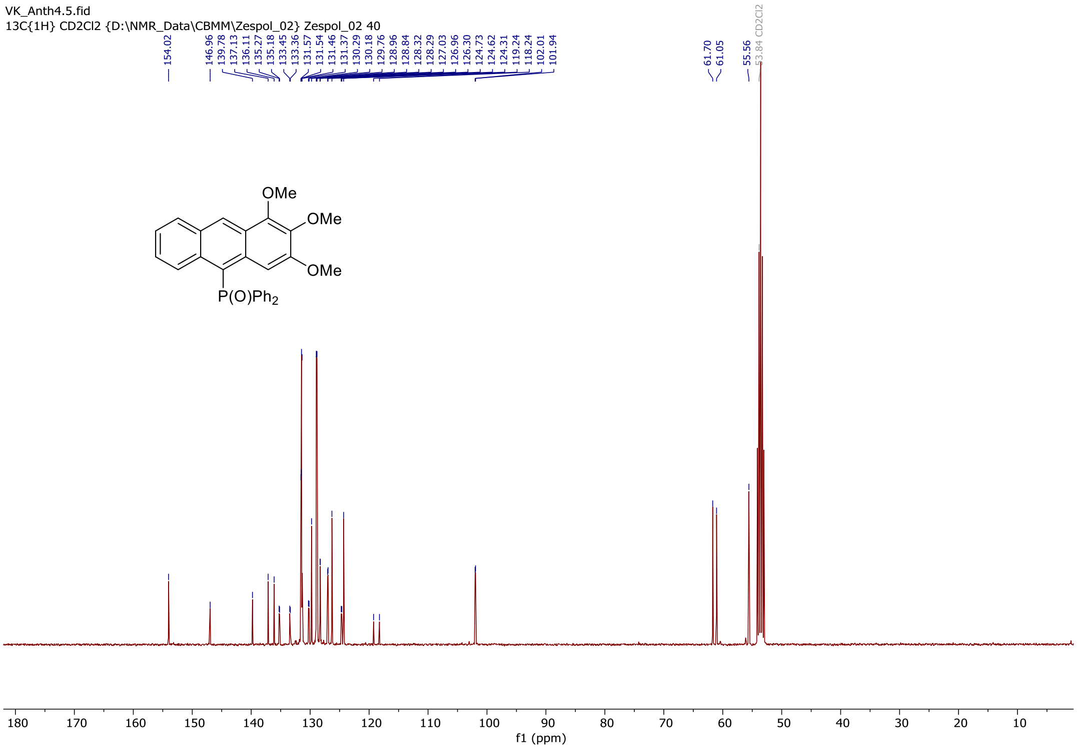

Figure S34. $^{13}C\{^{1}H\}$ *of* ***4d***.

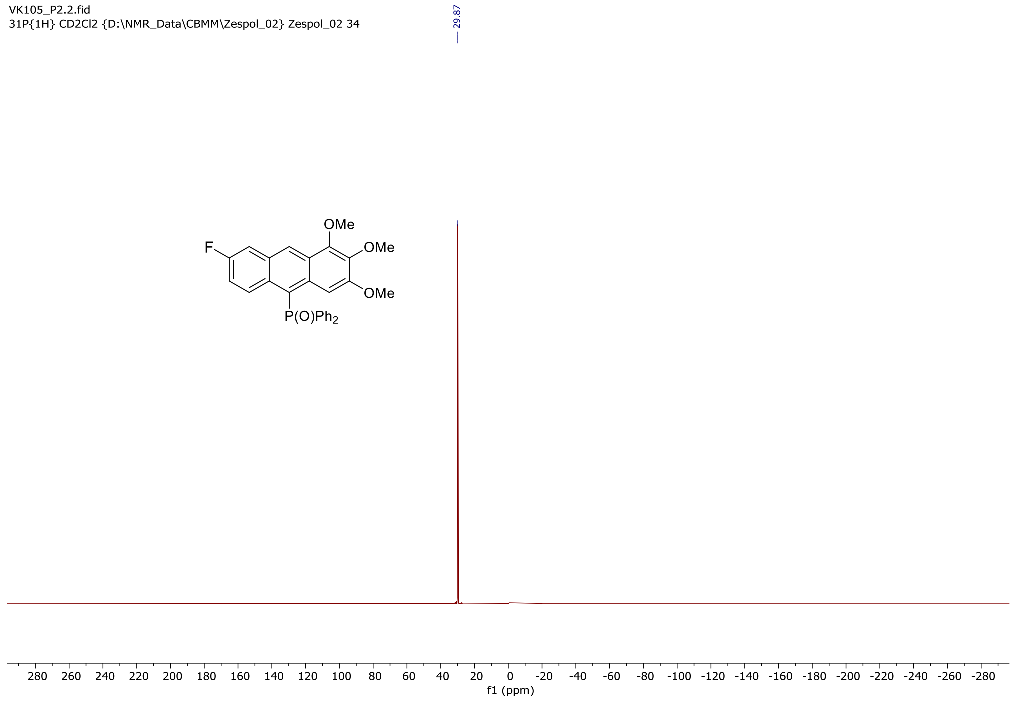

Figure S35. *^{31}P NMR of **4e**.*

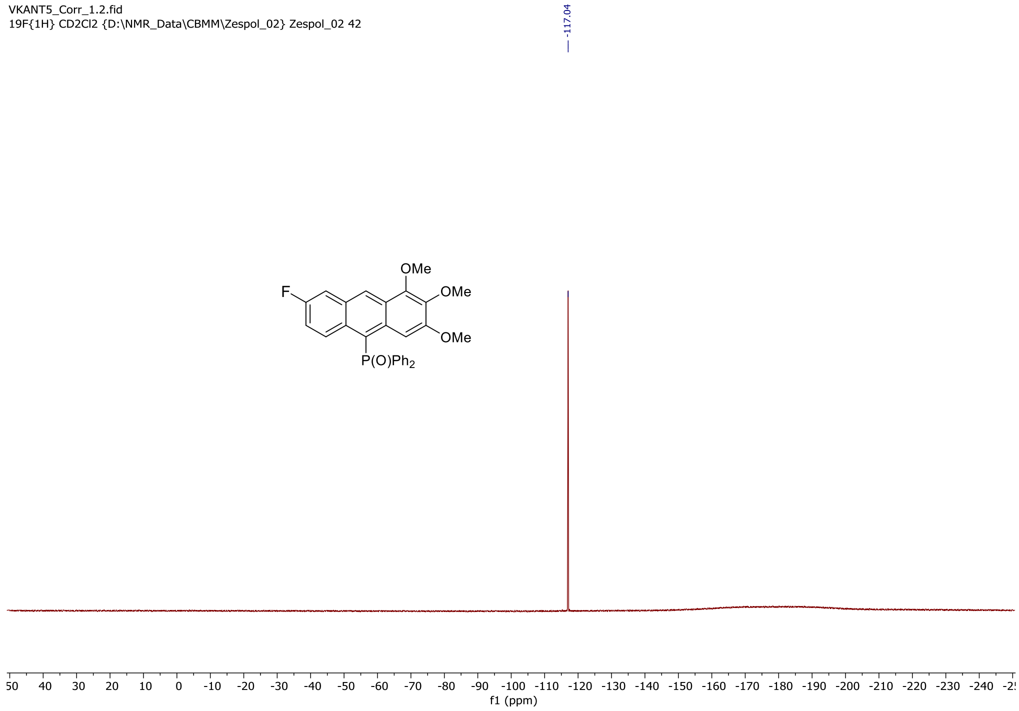

Figure S36. *^{19}F NMR of **4e**.*

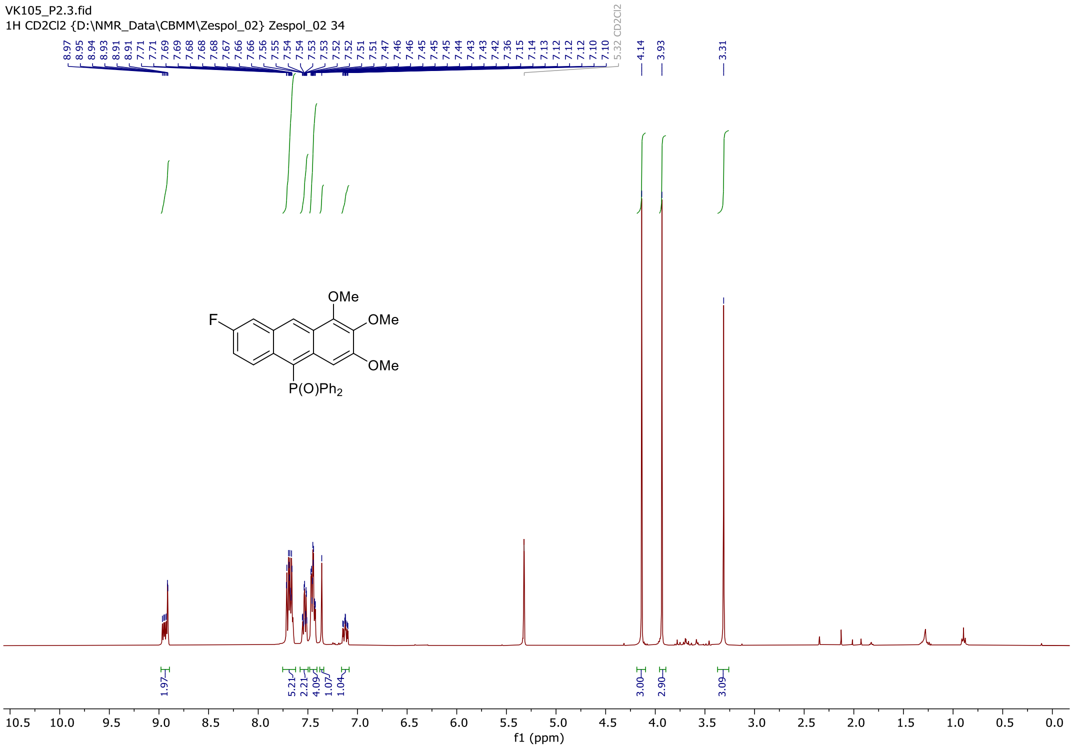

Figure S37. ^{1}H NMR of **4e**.

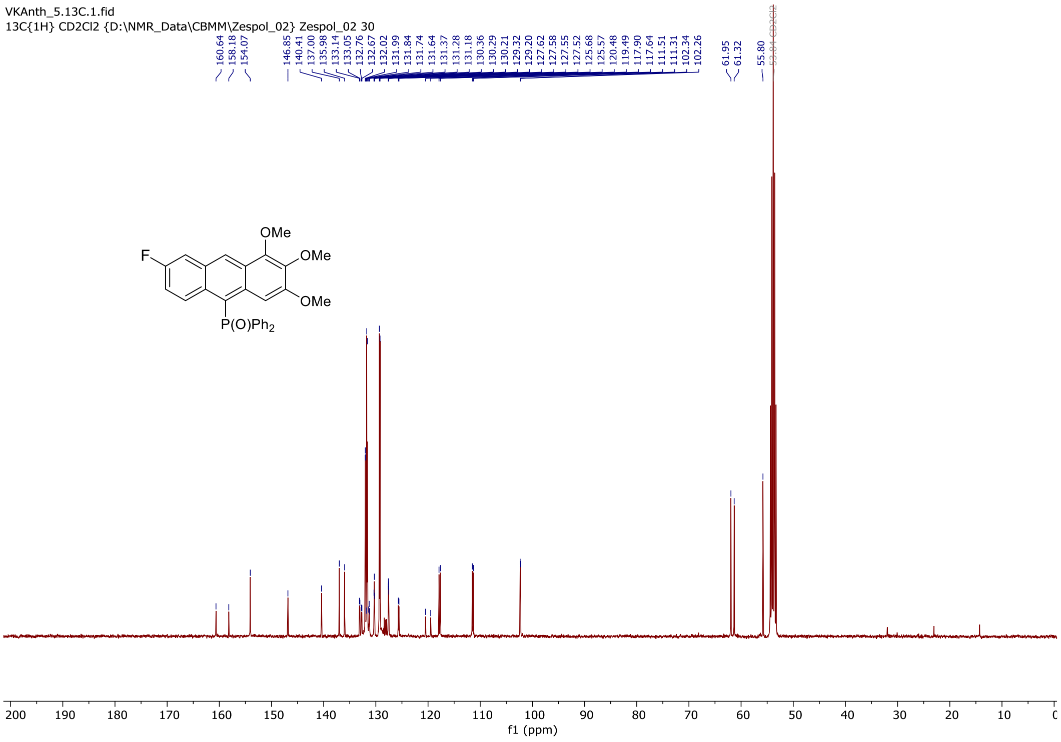

Figure S38. $^{13}C\{^{1}H\}$ NMR of **4e**.

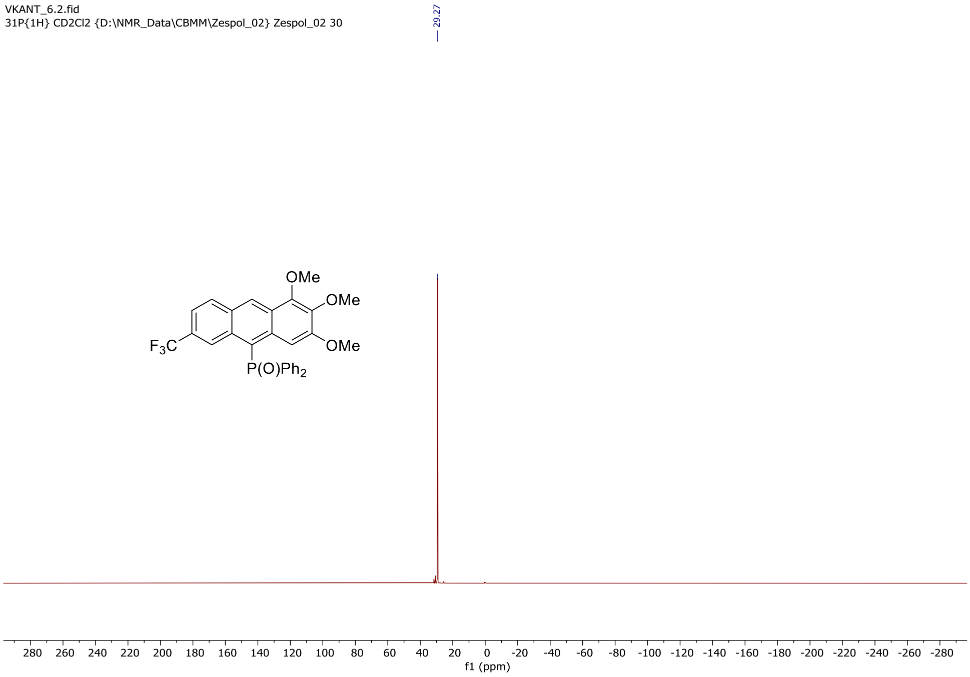

Figure S39. ^{31}P *NMR of* ***4f***.

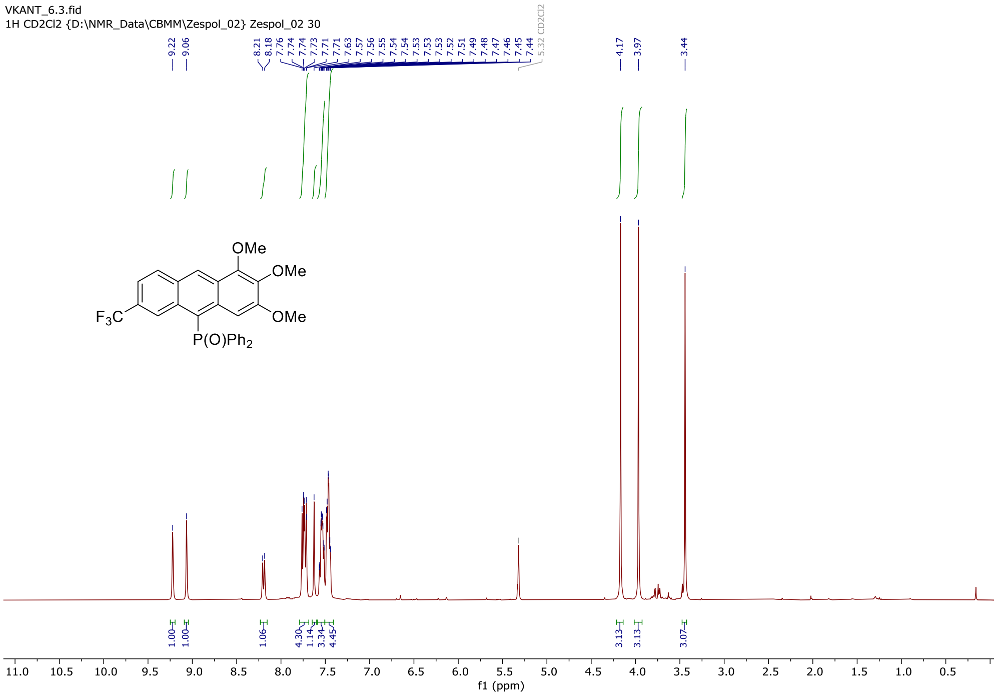

Figure S40. ^{1}H *NMR of* ***4f***.

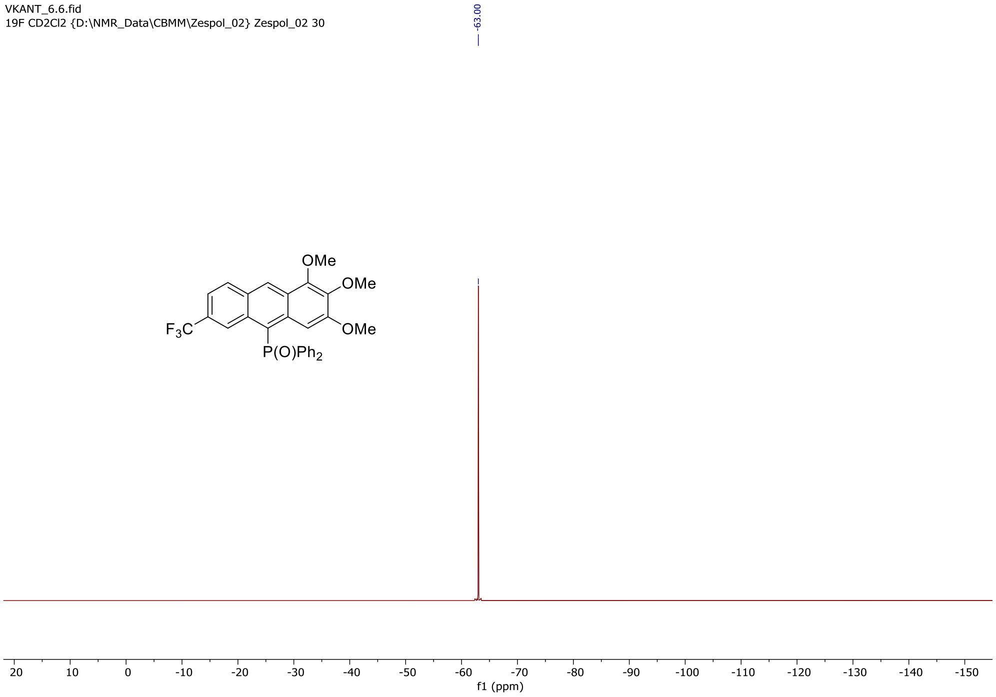

Figure S41. ^{19}F NMR of ***4f***.

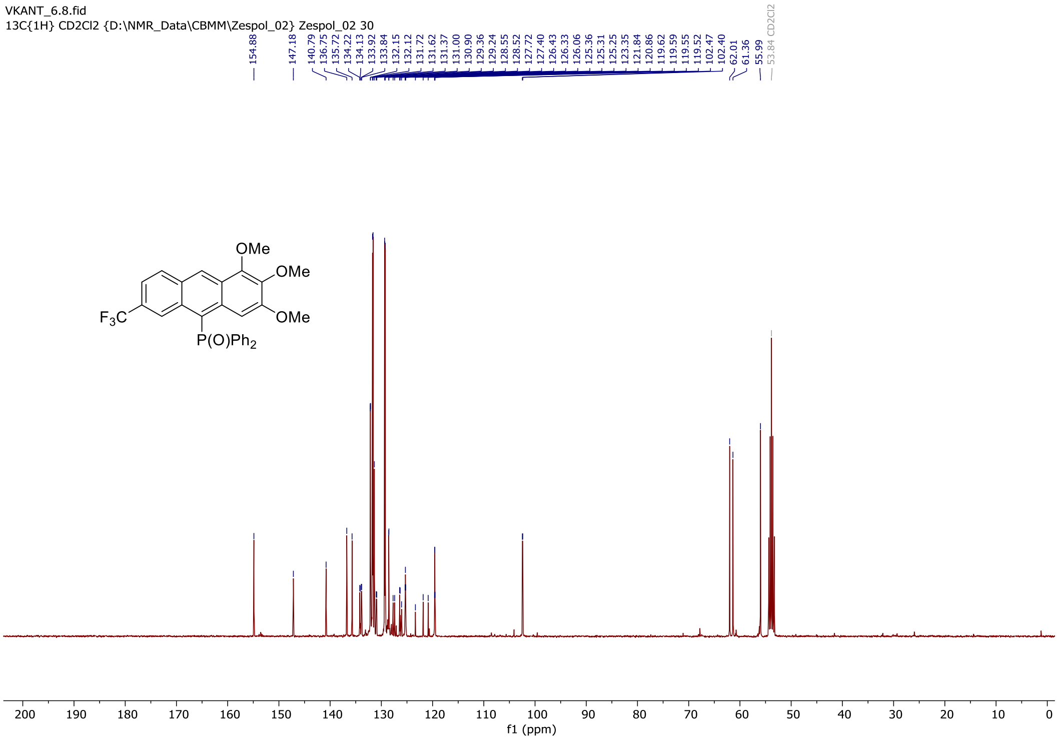

Figure S42. ^{13}C{^{1}H} NMR of ***4f***.

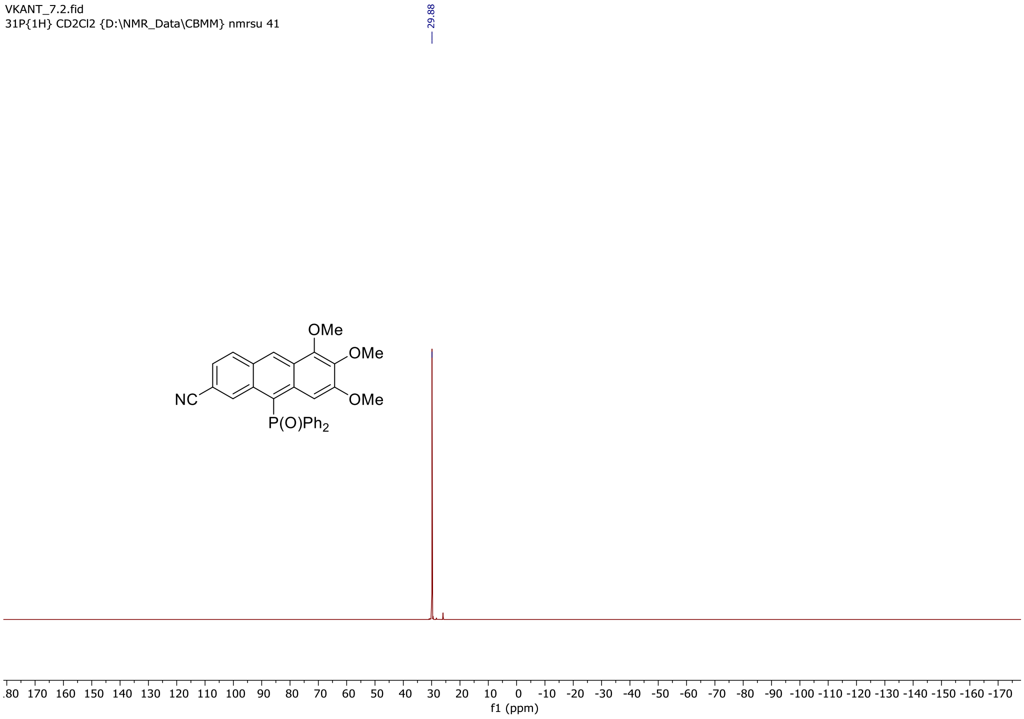

Figure S43. ^{31}P NMR of **4g**.

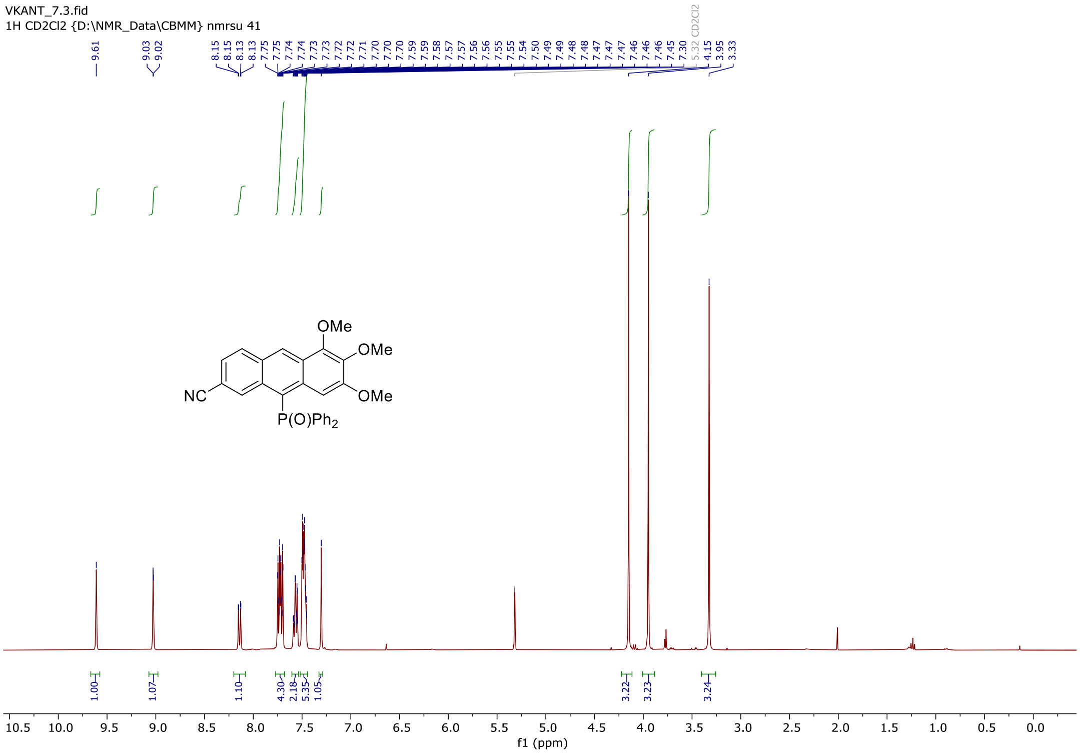

Figure S44. ^{1}H NMR of **4g**.

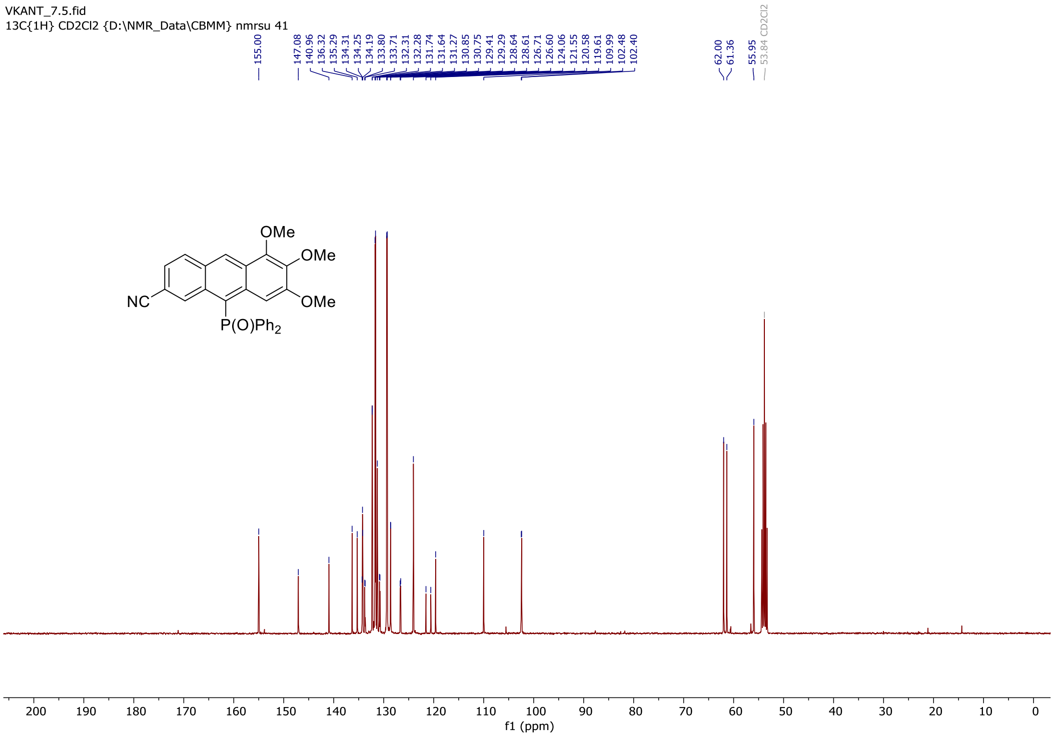

Figure S45. $^{13}C\{^1H\}$ *NMR of **4g**.*

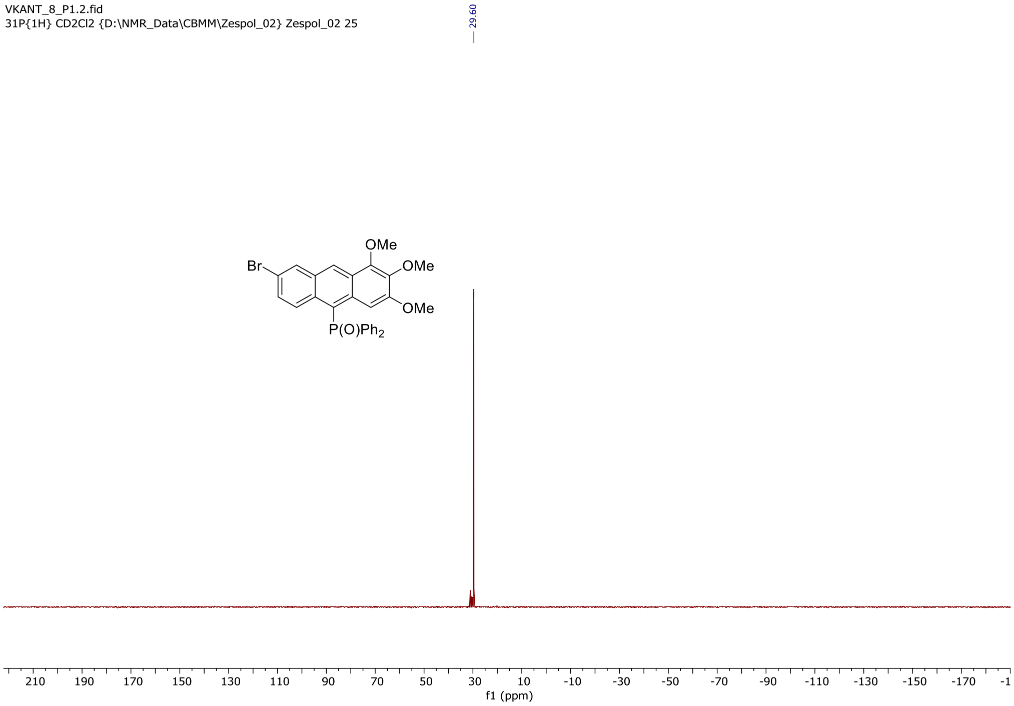

Figure S46. ^{31}P *NMR of **4h**.*

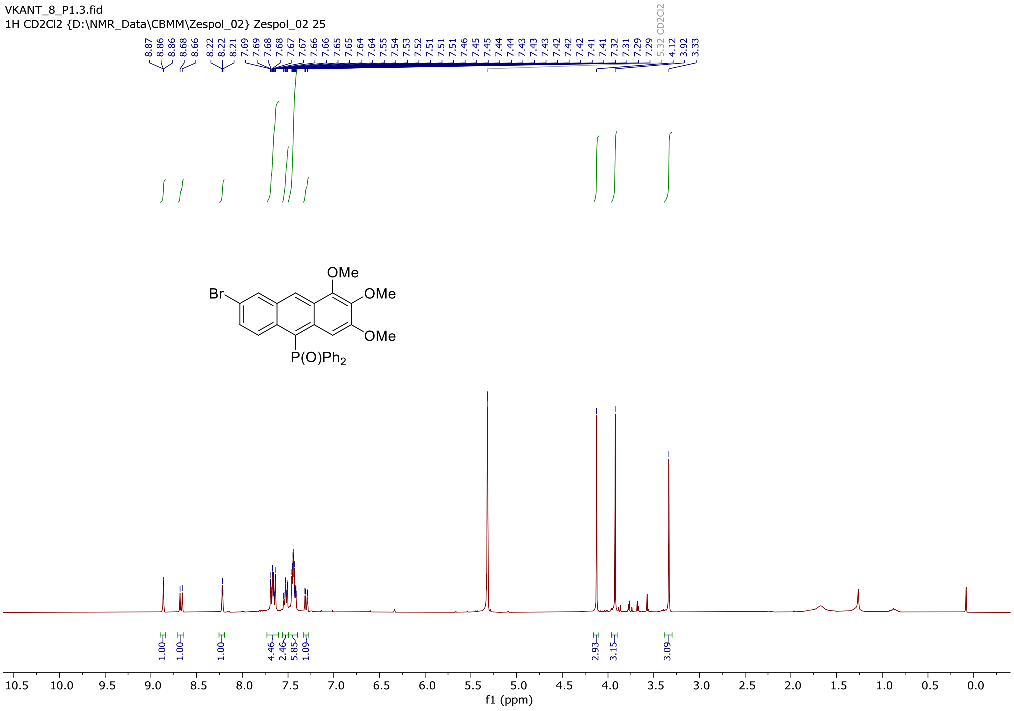

Figure S47. *^{1}H NMR of* ***4h***.

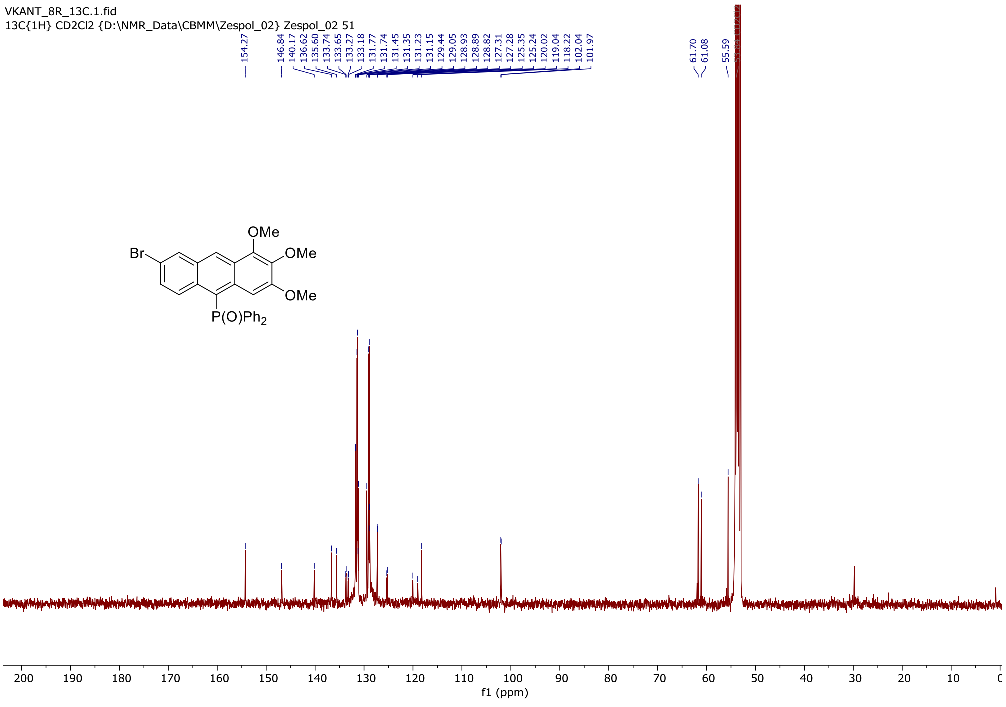

Figure S48. *^{13}C{^{1}H} NMR of* ***4h.***

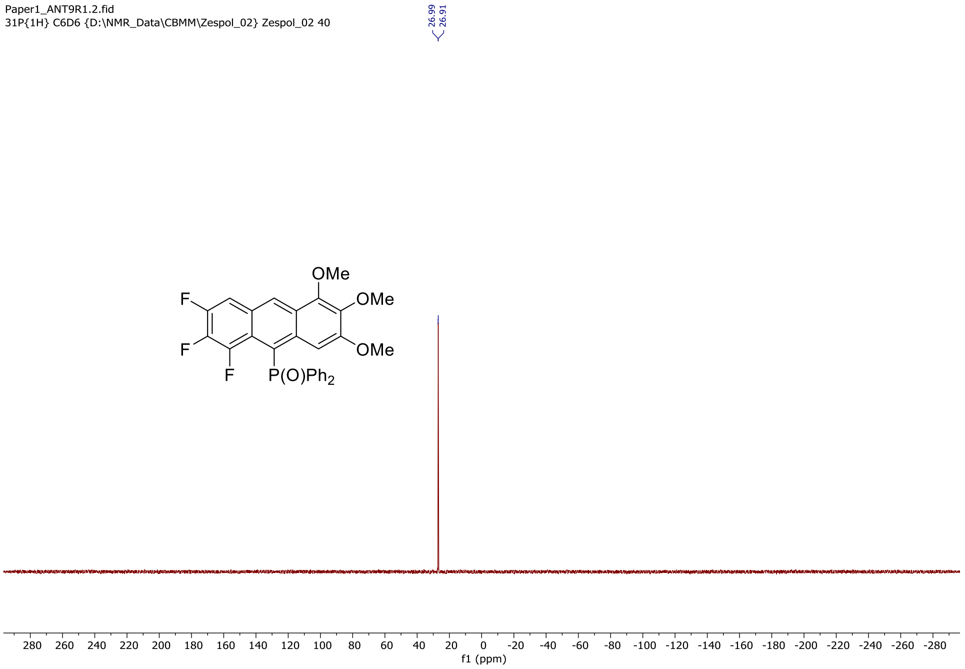

Figure S49. *31PNMR of* ***4i****.*

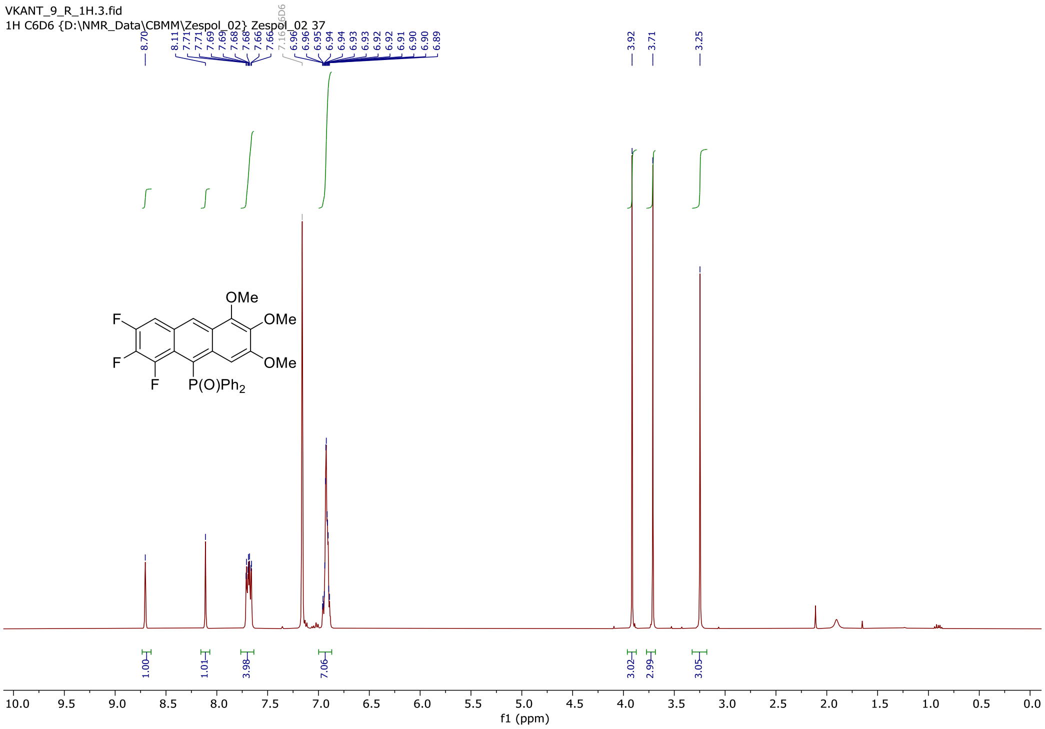

Figure S50. *^{1}H NMR of* ***4i****.*

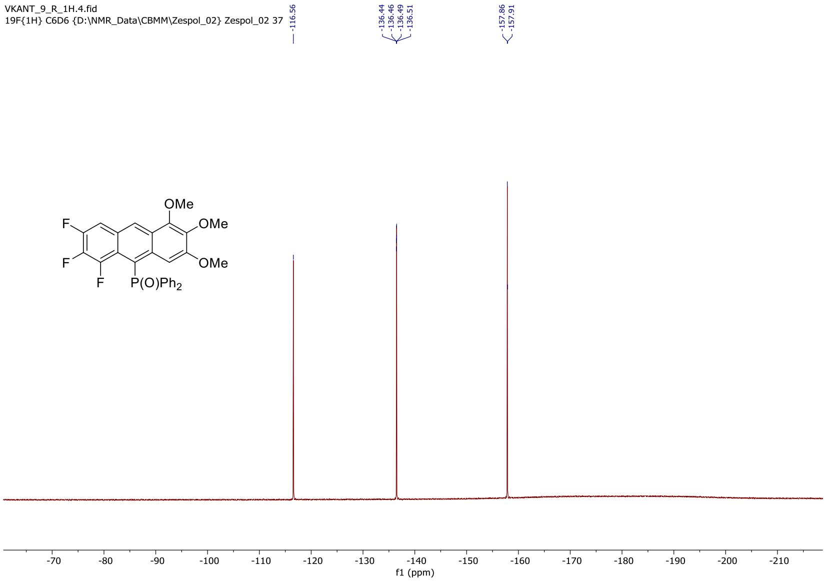

Figure S51. ^{19}F NMR of ***4i***.

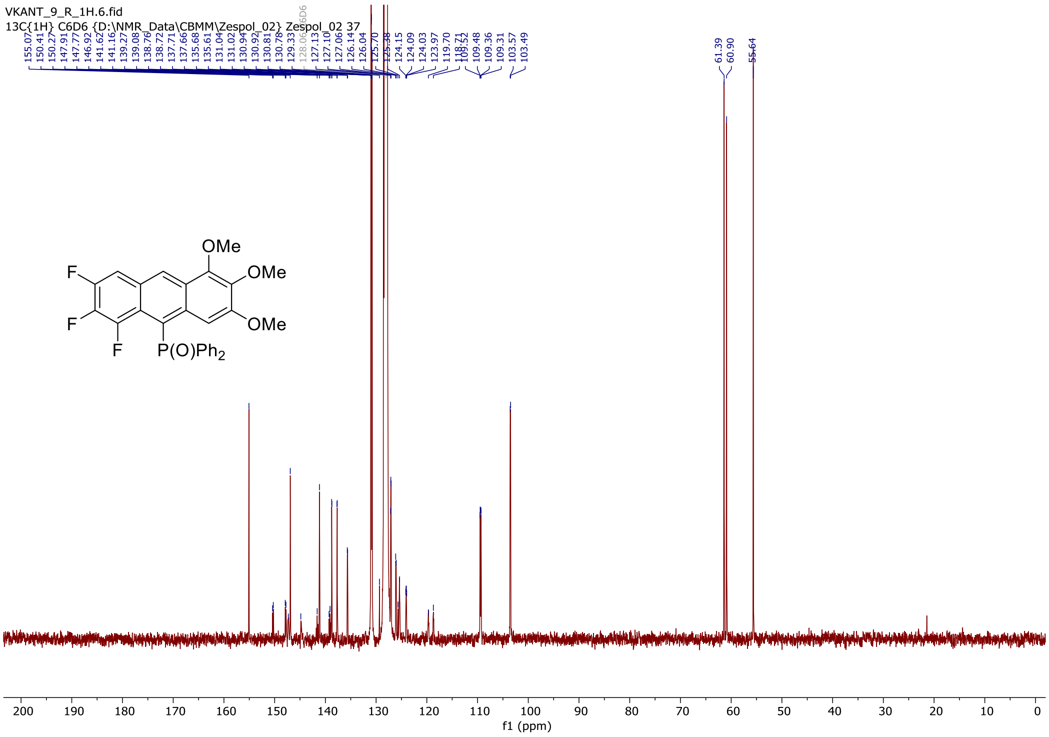

Figure S52. $^{13}C\{^{1}H\}$ NMR of ***4i***.

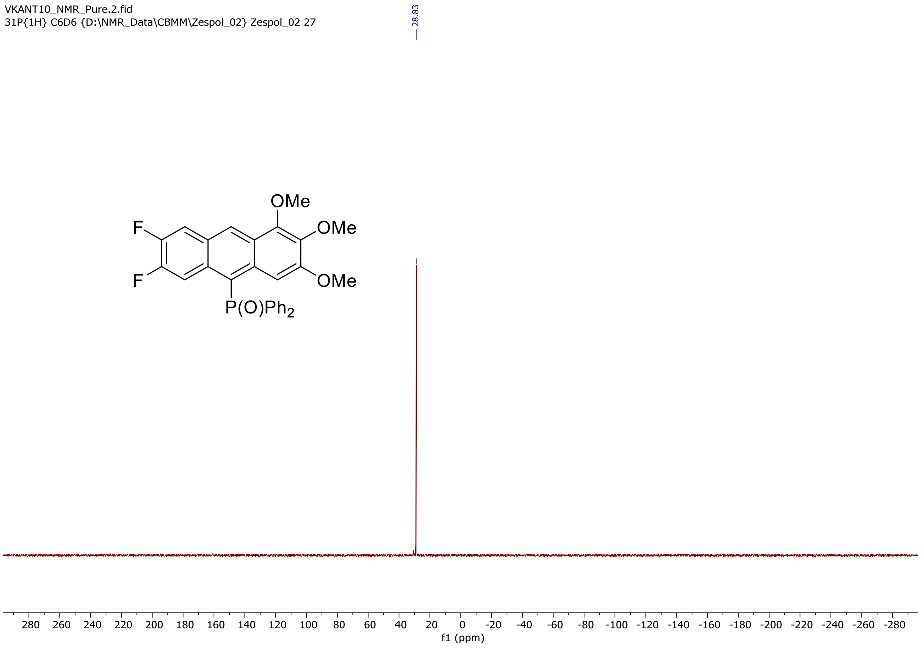

Figure S53. ^{31}*PNMR of* ***4j***.

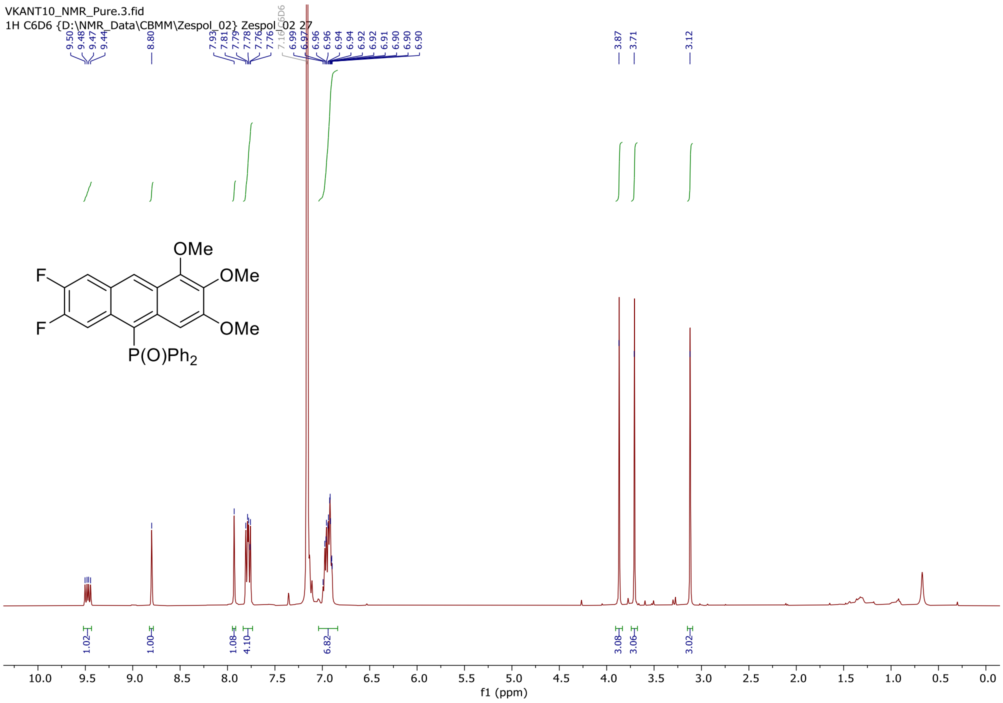

Figure S54. ^{1}*H NMR of* ***4j***.

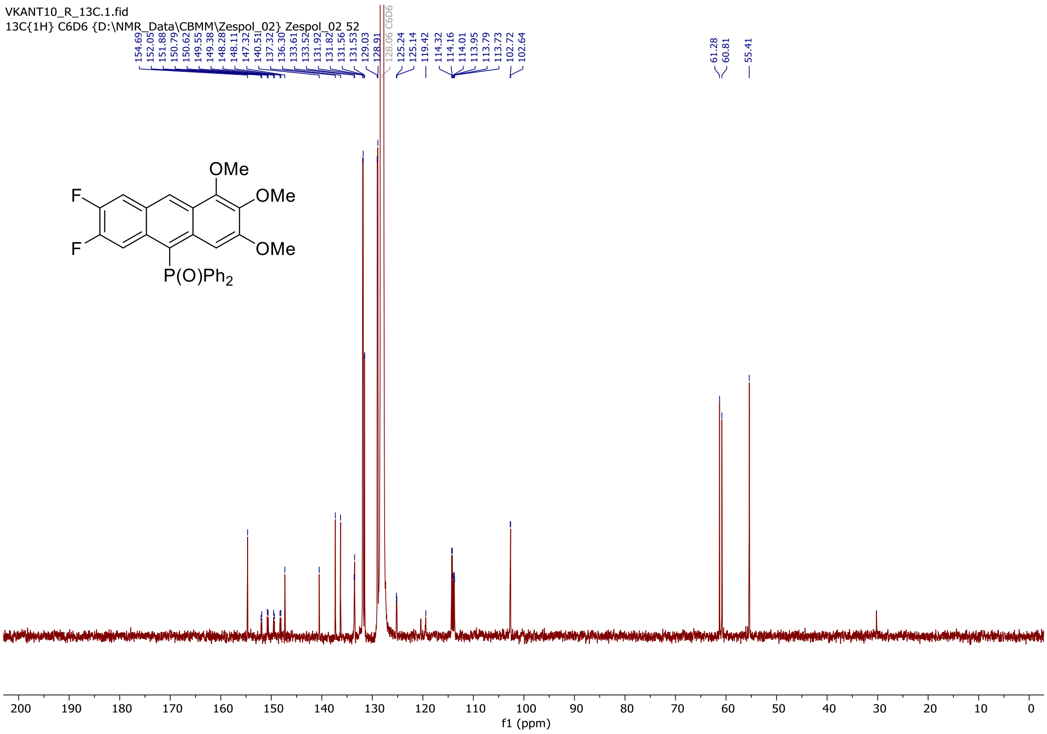

Figure S55. *$^{13}C\{^1H\}$ NMR of **4j**.*

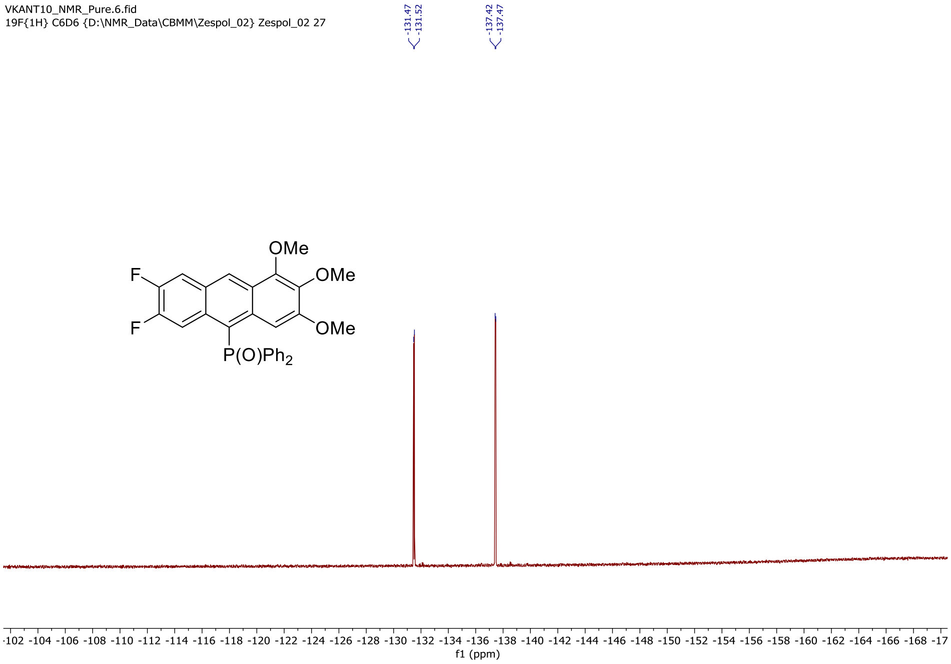

Figure S56. *^{19}F NMR of **4j**.*

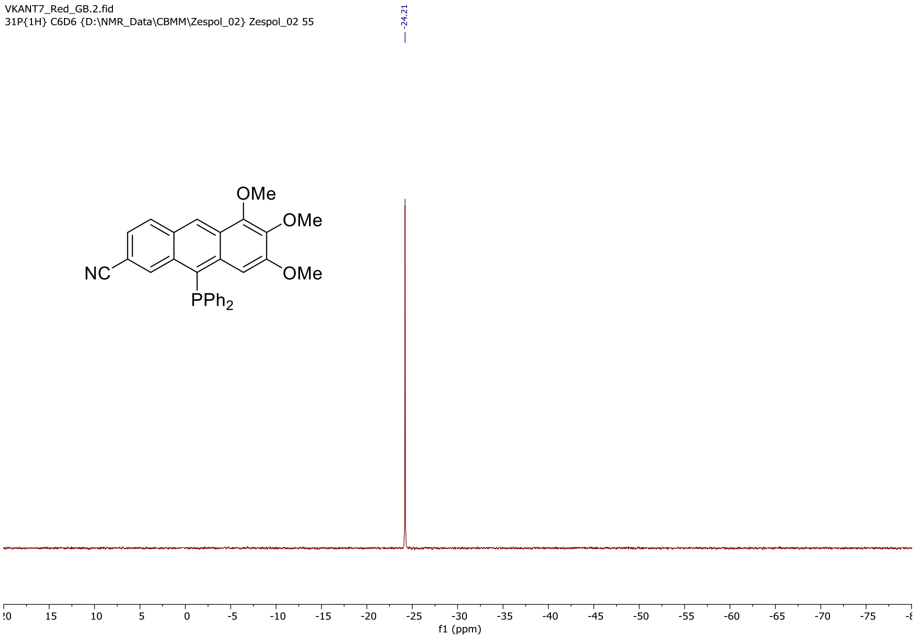

Figure S57. *^{31}P NMR of* ***5****.*

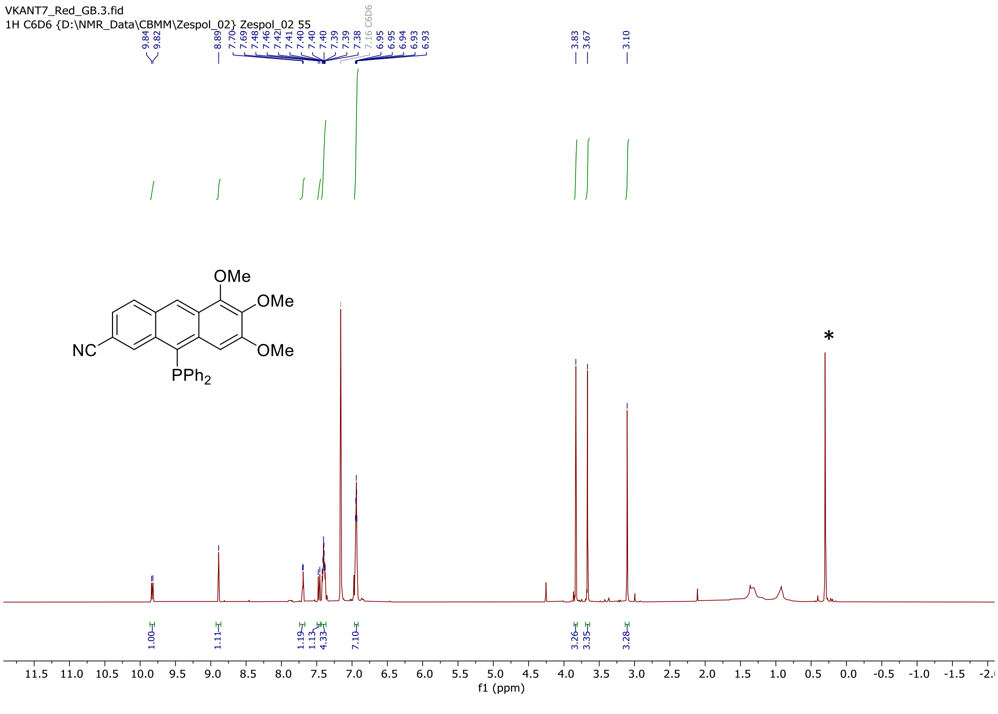

Figure S58. *^{1}H NMR of* ***5****. (* silane)*

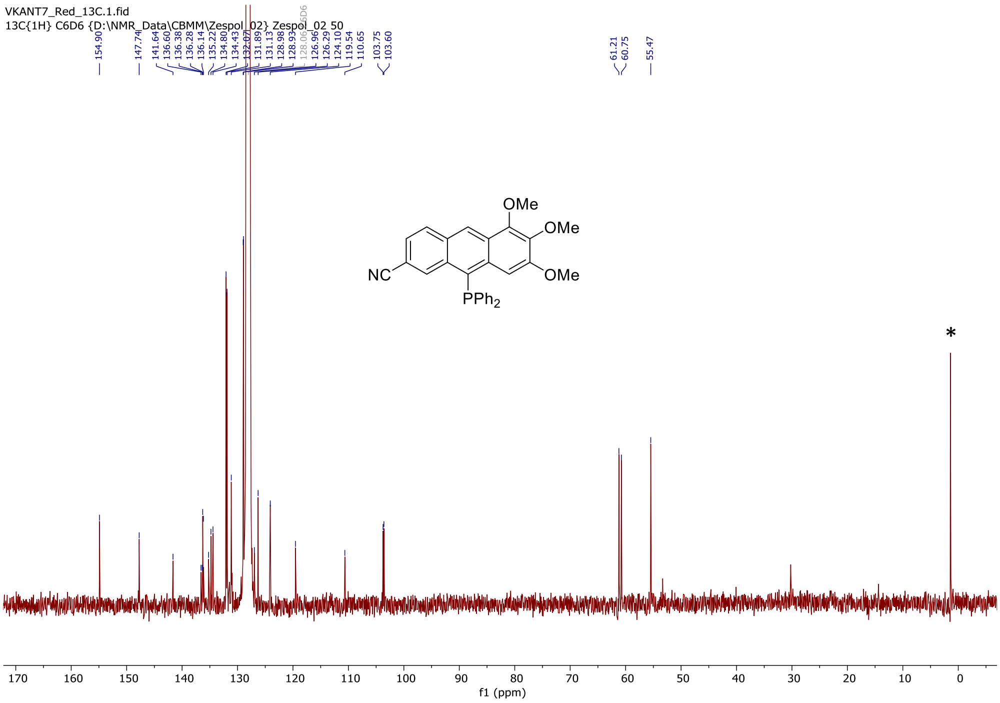

Figure S59. $^{13}C\{^1H\}$ *NMR of* ***5***. *(* silane)*

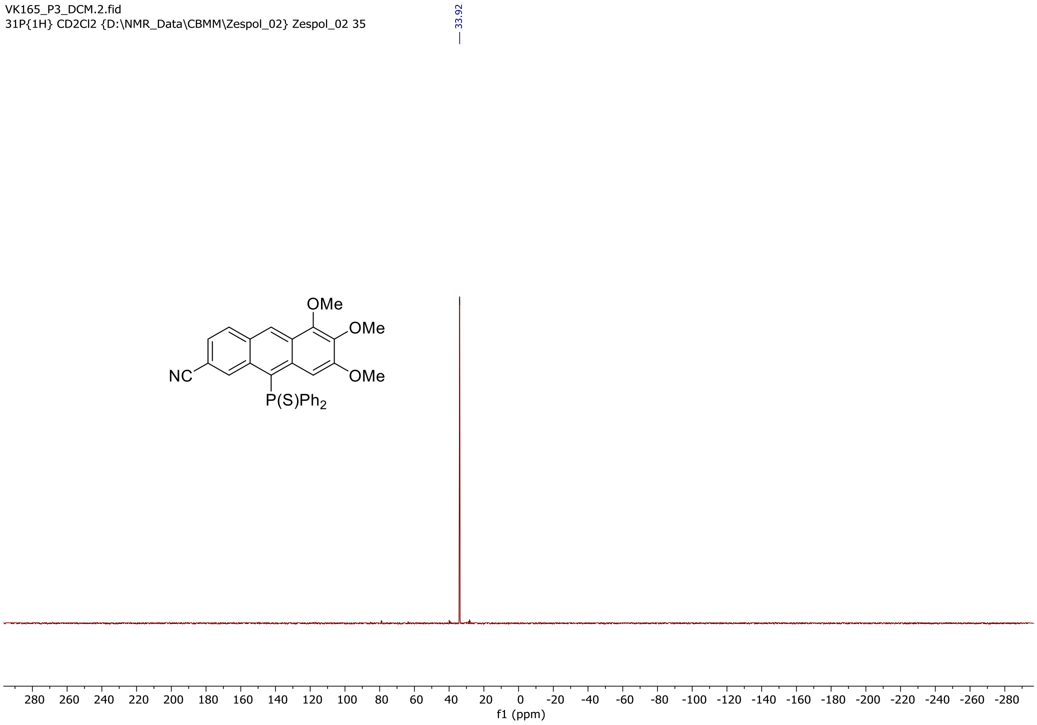

Figure S60. ^{31}P *NMR of* ***6***.

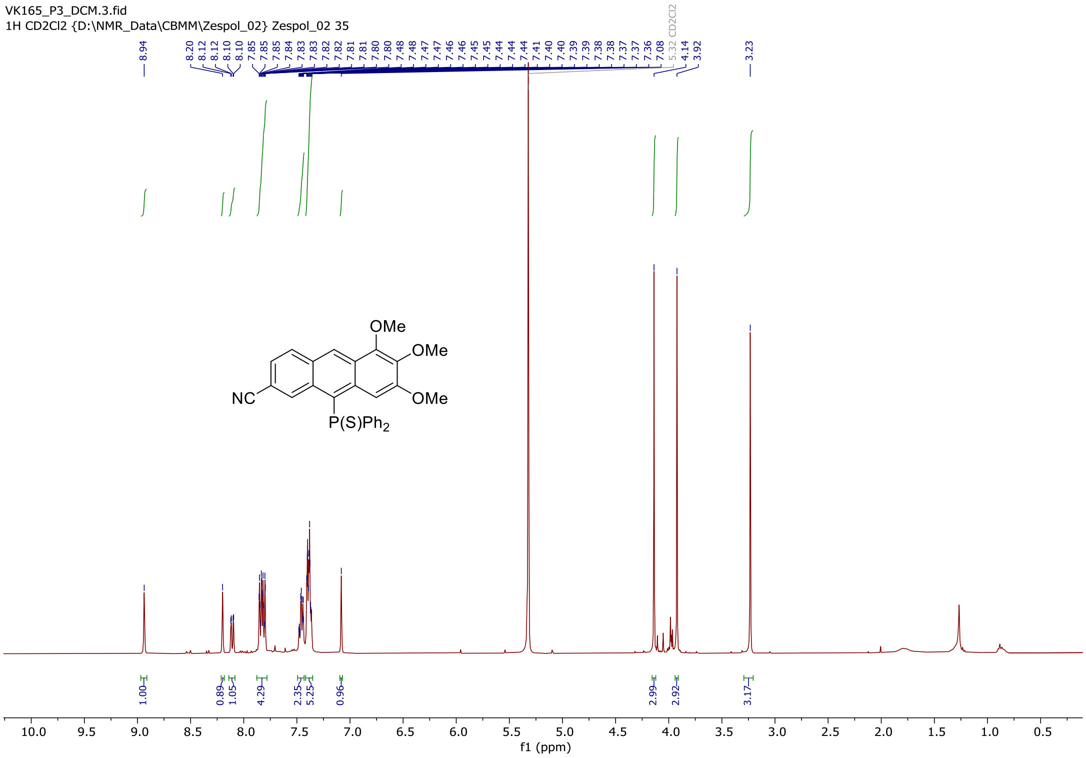

Figure S61. ^{1}H NMR of **6**.

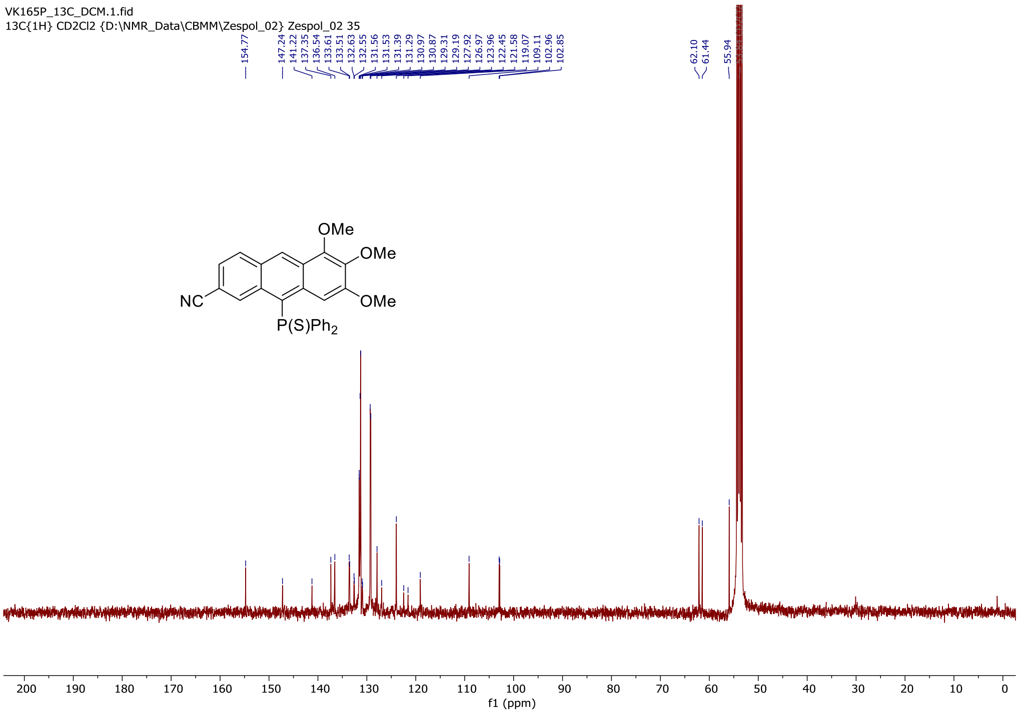

Figure S62. $^{13}C\{^{1}H\}$ NMR of **6**.

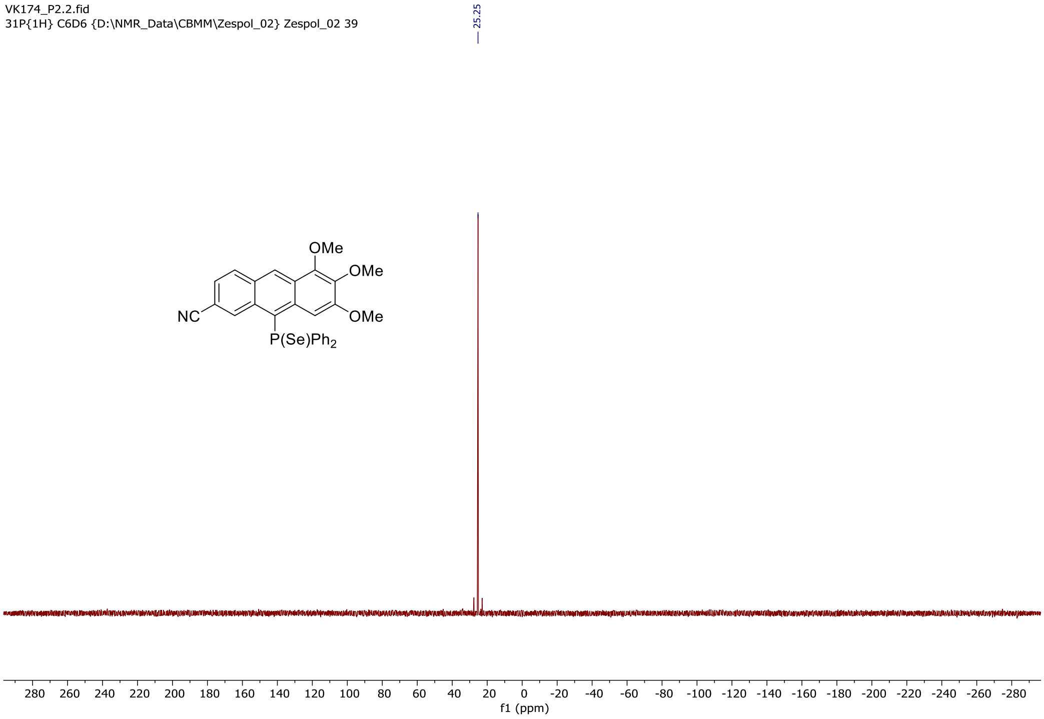

Figure S63. ^{31}P NMR of **7**.

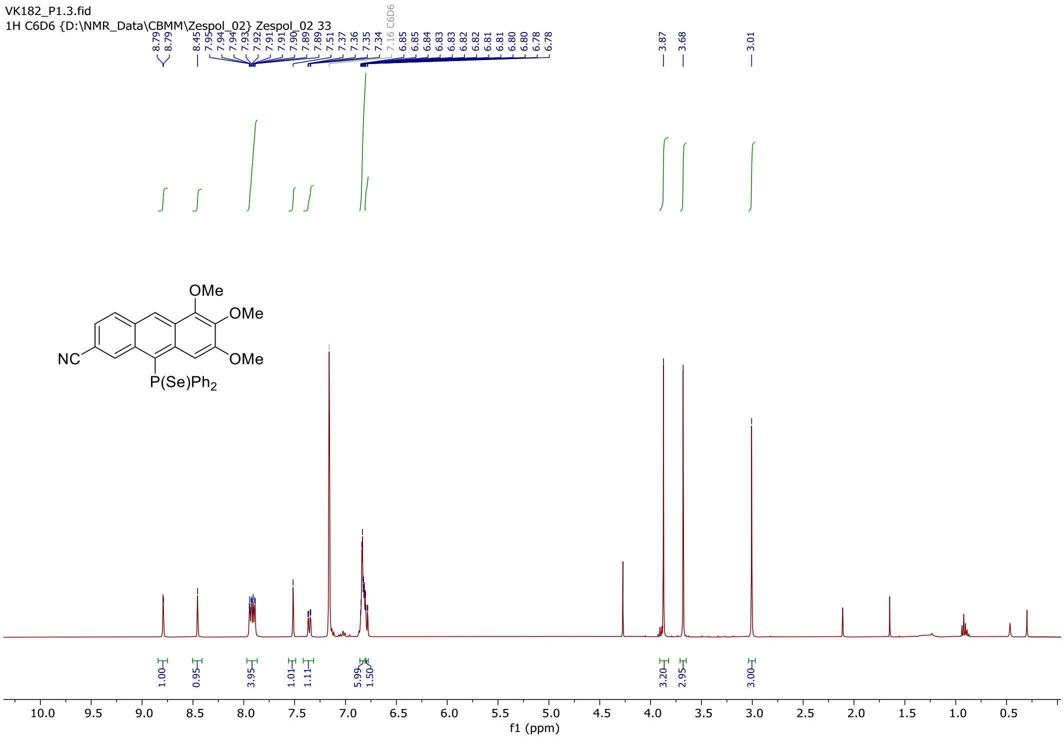

Figure S64. ^{1}H NMR of **7**.

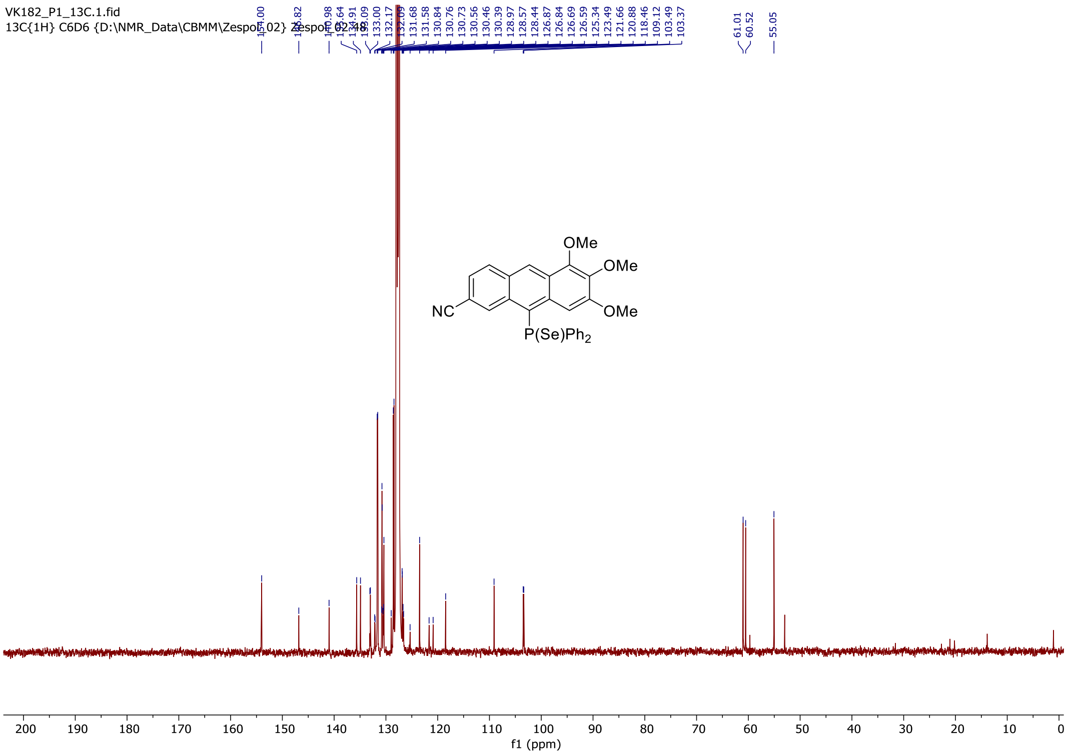

Figure S65. *$^{13}C\{^{1}H\}$ NMR of **7**.*

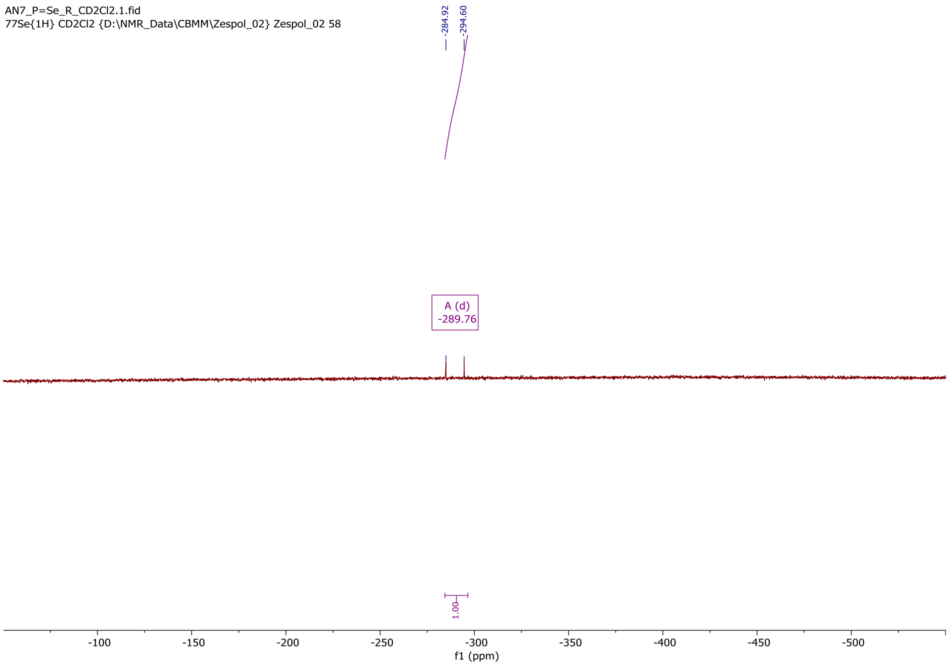

Figure S66. *$^{77}Se\{^{1}H\}$-CD_2Cl_2 NMR of **7**.*

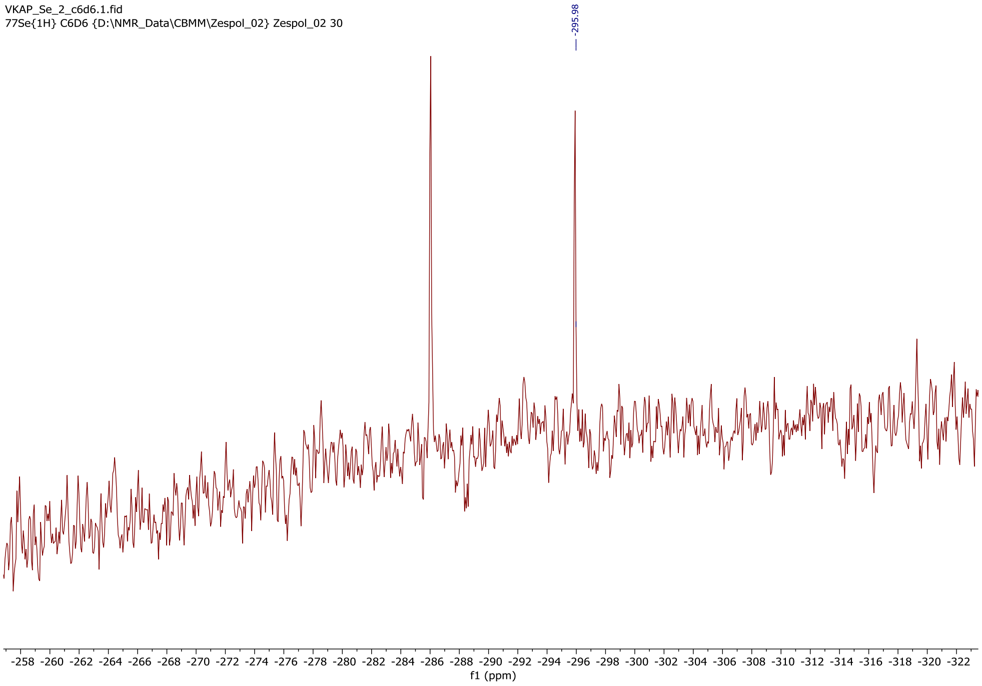

Figure S67. *^{77}Se{^{1}H}-C_6D_6 NMR of **7**.*

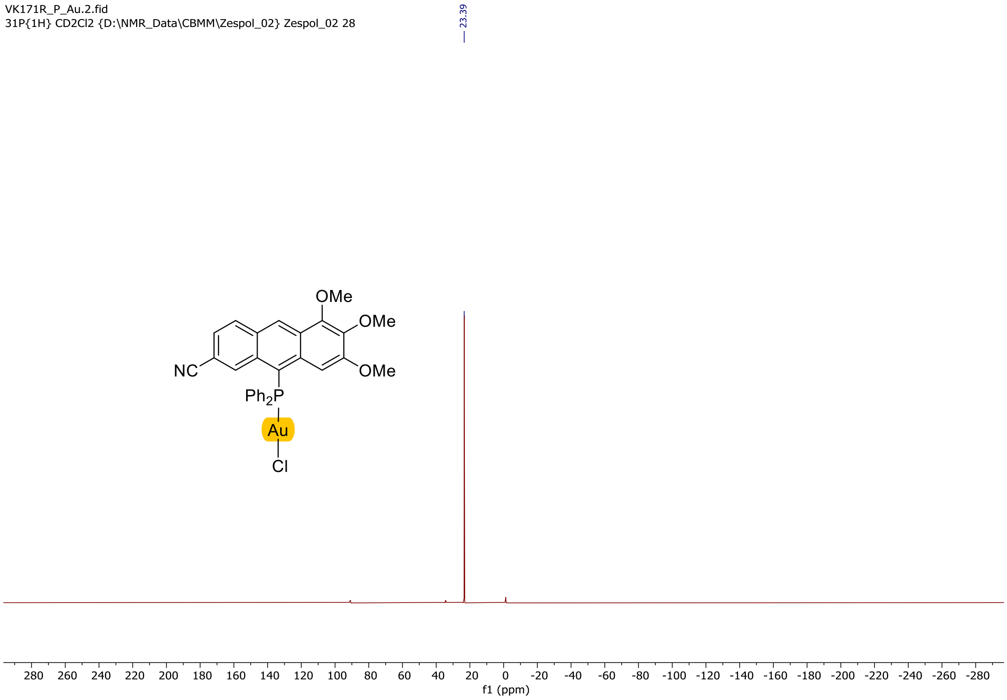

Figure S68. *^{31}P NMR of **8**.*

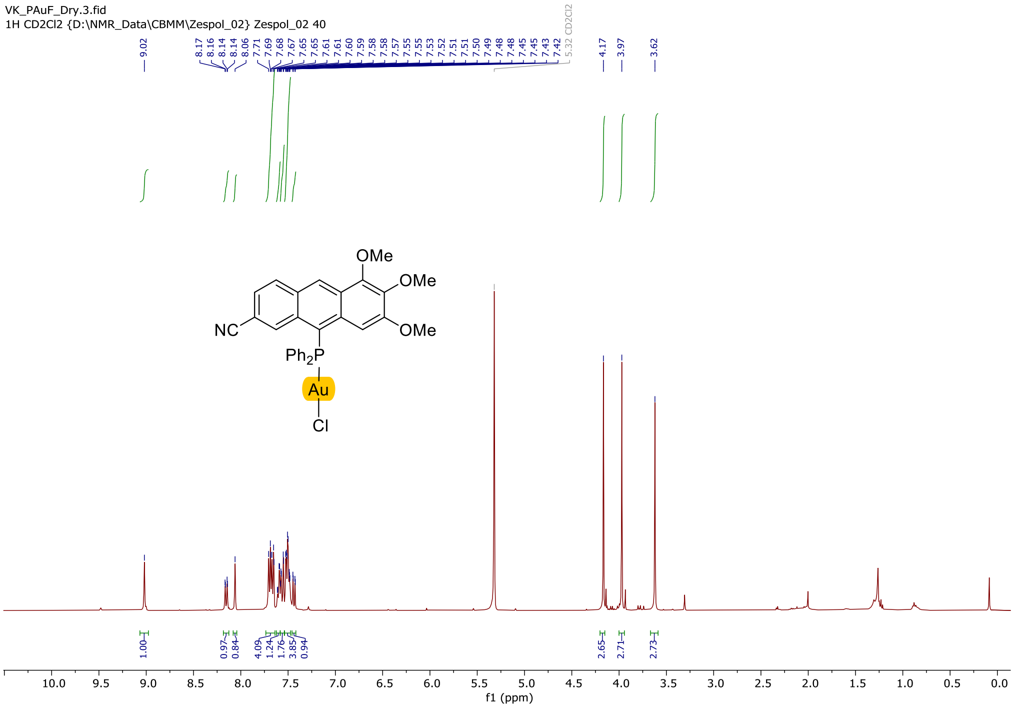

Figure S69. *^{1}H NMR of* ***8***.

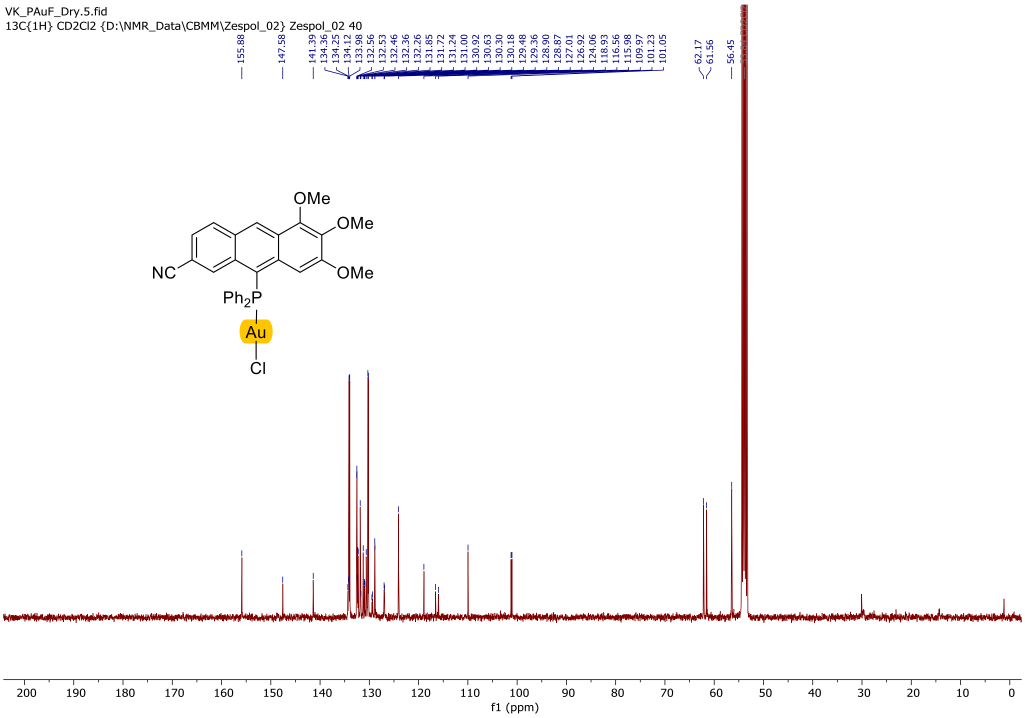

Figure S70. *^{13}C{^{1}H} NMR of* ***8***.

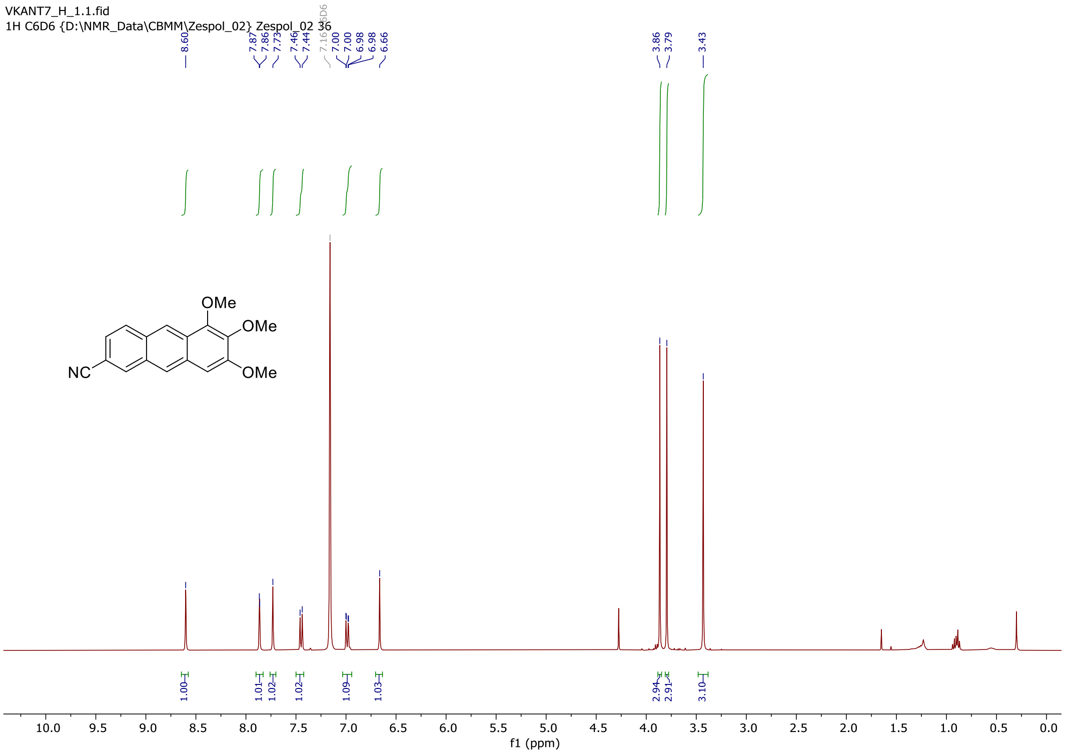

Figure S71. ^{1}H NMR of **9**.

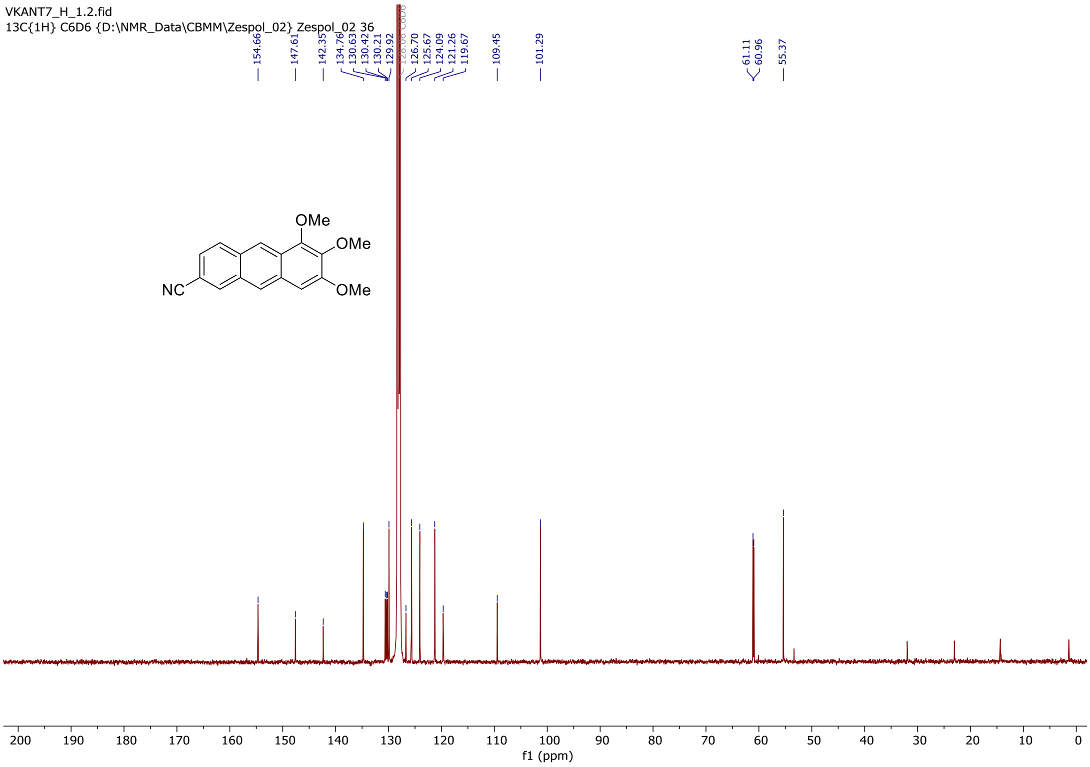

Figure S72. $^{13}C\{^{1}H\}$ NMR of **9**.

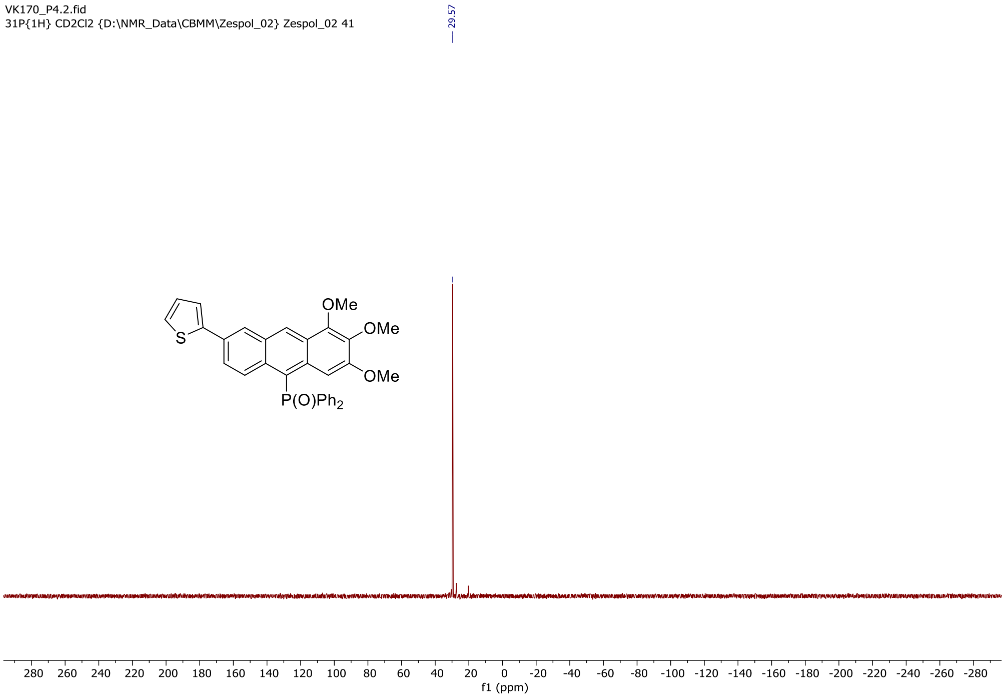

Figure S73. ^{31}P NMR of **10**.

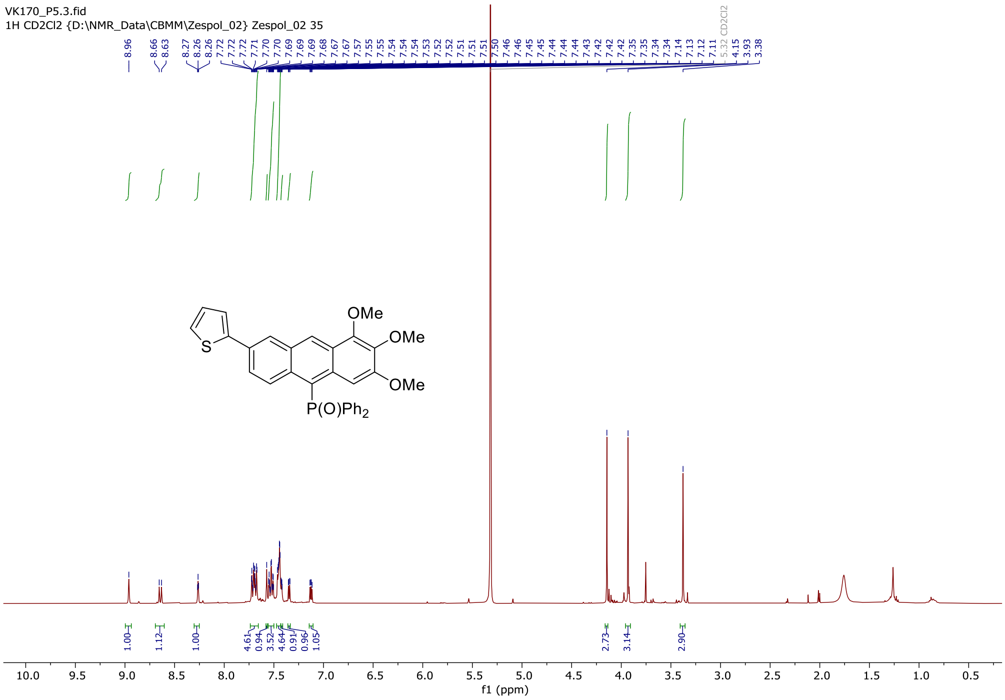

Figure S74. ^{1}H NMR of **10**.

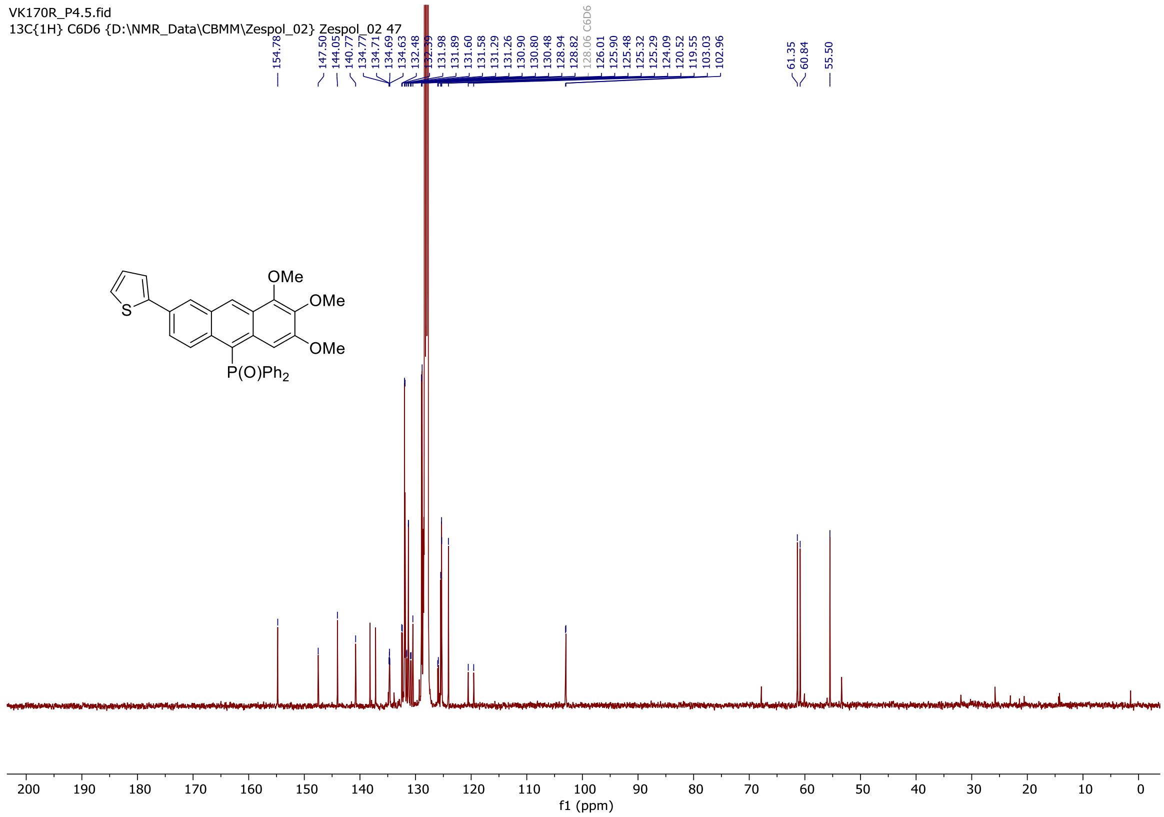

Figure S75. *$^{13}C\{^{1}H\}$ NMR of* ***10***.

4. Photophysical properties:

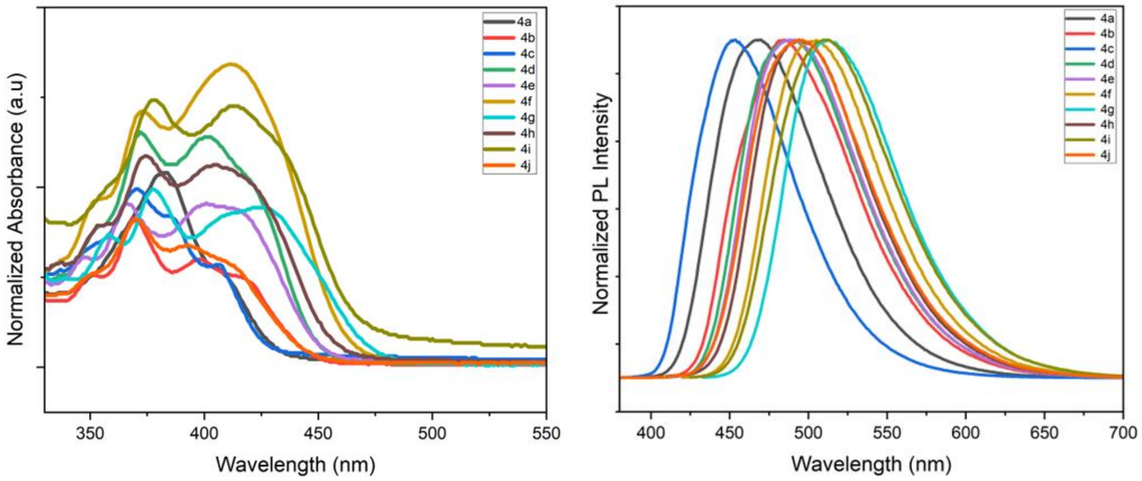

Figure S76. *Normalized absorbance and emission spectra of anthracene* ***4 a-j*** *in toluene solution.*

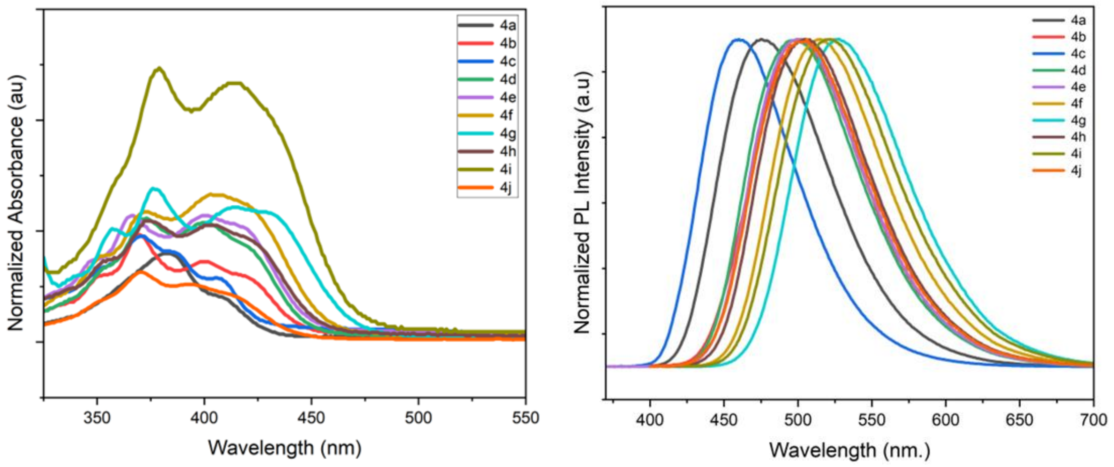

Figure S77. *Normalized absorbance and emission spectra of anthracene* ***4 a-j*** *in dichloromethane solution.*

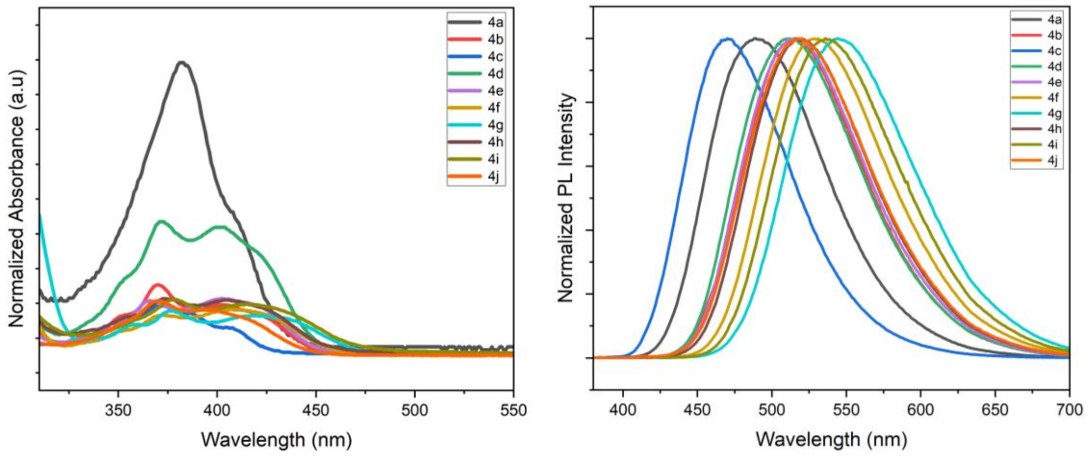

Figure S78. *Normalized absorbance and emission spectra of anthracene* ***4 a-j*** *in methanol solution.*

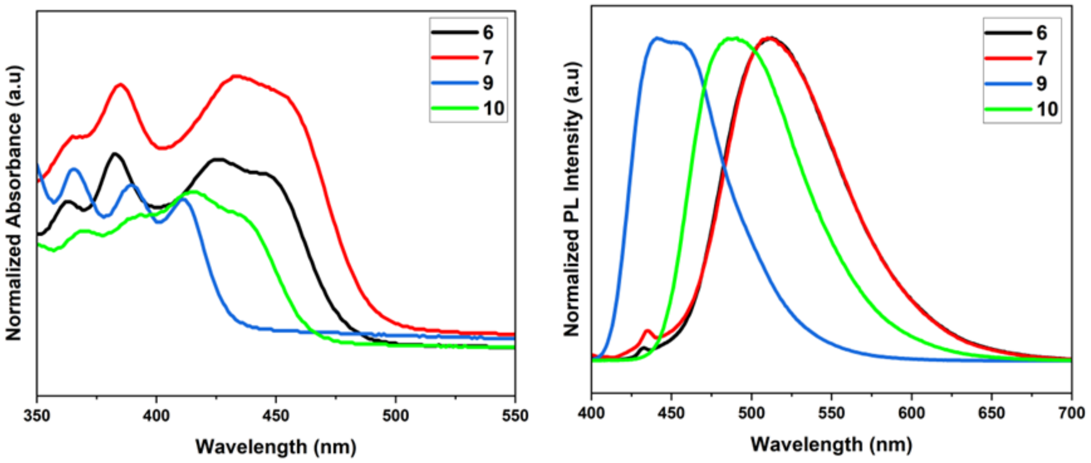

Figure S79. *Normalized absorbance and emission spectra of anthracene* ***6****,* ***7****,* ***9****, and* ***10*** *in toluene solution.*

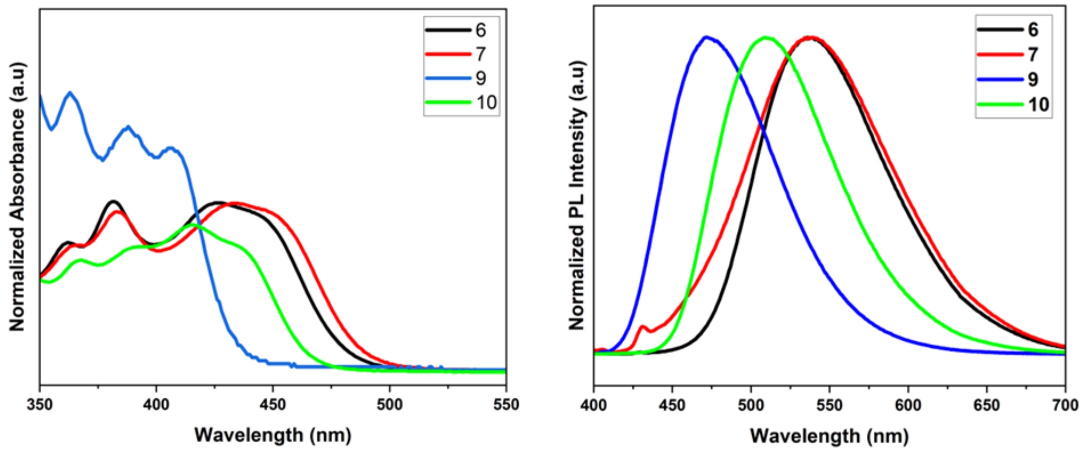

Figure S80. *Normalized absorbance and emission spectra of anthracene* ***6****,* ***7****,* ***9****, and* ***10*** *in methanol solution.*

Table S1. Solid-state emission, quantum yield, and lifetime in dichloromethane solution of selected anthracene.

**Nr**	**PL**[a] λ_{max} (nm)	**QY**[b] (%)	**τ**[c] (ns)
	Solid	Solid	DCM
**4b**	486	43.7	15.3
**4d**	485	40.5	17
**4e**	502	46.7	20.1
**4f**	511	>95	22.7
**4g**	532	79.2	23.3
**4i**	518	13.3	18.0
**4j**	494	36.6	36.6

[a] PL - emission maximum; [b] the absolute fluorescence quantum yield (QY); [c] lifetime (τ).

5. Crystal Structure Data

X-ray diffraction data for **9** and two dichloromethane solvates **4b** and **4j** were collected using an Oxford Diffraction Xcalibur Saphire 3 diffractometer with CuKα radiation. The structures were solved by direct methods and refined by full-matrix least-squares on F^2 with SHELXL-2019.[50] The non-hydrogen atoms were refined anisotropically. All aromatic H atoms and H atoms of methyl and methylene groups were positioned geometrically and constrained to ride on their parent atoms, with C–H distances of 0.93, 0.96 and 0.97 Å, respectively, and with $U_{iso}(H_{aromatic/methylene})$ and $U_{iso}(H_{methyl})$ values of 1.2 $U_{eq}(C_{aromatic/methylene})$ and 1.5 $U_{eq}(C_{methyl})$. Details of data collections and structure refinements have been deposited with the Cambridge Crystallographic Data Centre as supplementary publication number: CCDC 2378975 (**4b·DCM**), CCDC 2378976 (**4j·DCM**) and CCDC 2378974 (**9**).[51]

*Crystal structure data for **4b·DCM**:* $C_{29}H_{23}O_5P{\cdot}CH_2Cl_2$, M = 567.37, monoclinic, space group P2$_1$/n (No. 14), a = 11.4126(2) Å, b = 16.6184(2) Å, c = 14.7800(2) Å, β = 104.583(2)°, Z = 4, T = 293(2) K, D_{calc} = 1.389 g·cm^{-3}, CuKα radiation, $2\theta_{max}$ = 134.68°, 44224 reflections collected, 4868 reflections unique and 4562 reflections with $I > 2\sigma(I)$. Final GooF = 1.036, R1 = 0.0466 for 4562 reflections and 346 parameters. Further details on the crystal structure investigation have been deposited with the Cambridge Crystallographic Data Centre as supplementary publication number CCDC 2378975.

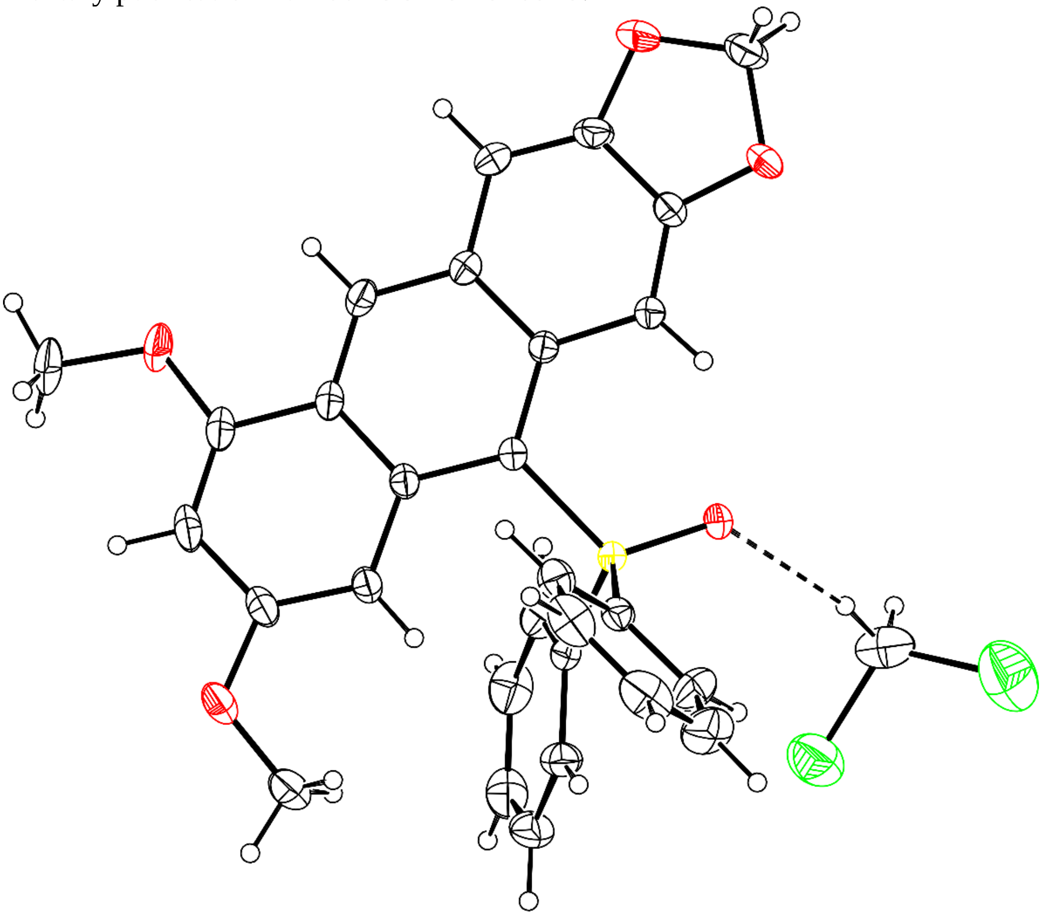

*Figure S81. The molecular structure of DCM solvate of **4b**; displacement ellipsoids are drawn at the 30% probability level. The C-H···O hydrogen bond between 4b and DCM are marked by dashed lines.*

Crystal structure data for ***4j·DCM****:* $C_{30}H_{25}O_4F_2P·CH_2Cl_2$, M = 589.37, monoclinic, space group $P\bar{1}$ (No. 2), a = 8.5583(3) Å, b = 11.4923(4) Å, c = 15.4268(5) Å, α = 107.454(3)°, β = 99.947(3)°, γ = 103.737°, Z = 2, T = 293(2) K, D_{calc} = 1.443 g·cm^{-3}, CuKα radiation, $2\theta_{max}$ = 136.00, 20158 reflections collected, 4887 reflections unique and 4397 reflections with $I > 2\sigma(I)$. Final GooF = 1.043, R1 = 0.0433 for 4397 reflections and 356 parameters. Further details on the crystal structure investigation have been deposited with the Cambridge Crystallographic Data Centre as supplementary publication number CCDC 2378976.

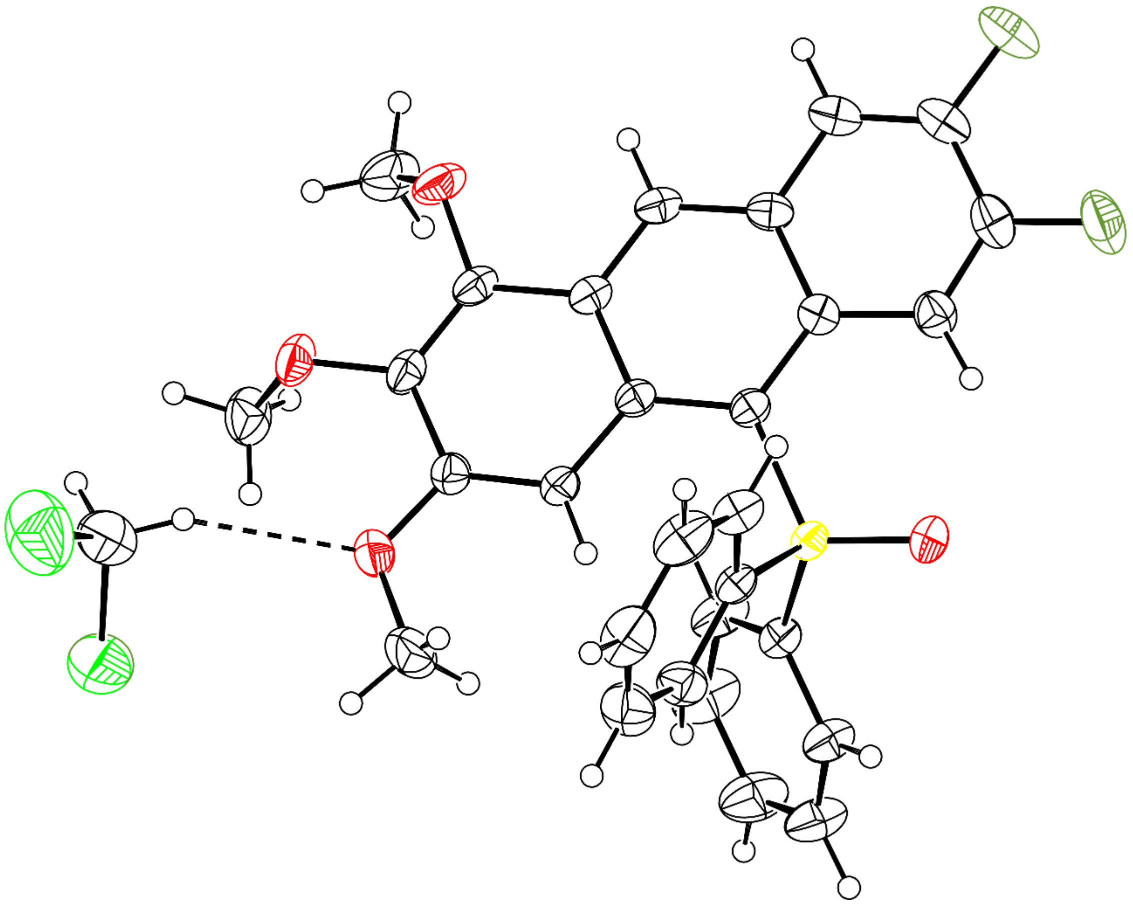

Figure S82. *The molecular structure of DCM solvate of* ***4j****; displacement ellipsoids are drawn at the 30% probability level. The C-H···O hydrogen bond between* ***4j*** *and DCM are marked by dashed lines.*

*Crystal structure data for **9**:* $C_{18}H_{15}O_3N$, M = 293.31, triclinic, space group P$\bar{1}$ (No. 2), a = 7.9397(4) Å, b = 9.5127(5) Å, c = 10.2548(5) Å, α = 102.595(4)°, β = 101.660(4)°, γ = 91.450(4)°, Z = 2, T = 293(2) K, D_{calc} = 1.319 g·cm^{-3}, CuKα radiation, θ_{max} = 67.936°, 9611 reflections collected, 2664 reflections unique and 2352 reflections with $I > 2\sigma(I)$. Final GooF = 1.050, R1 = 0.0401 for 2352 reflections and 203 parameters. Further details on the crystal structure investigation have been deposited with the Cambridge Crystallographic Data Centre as supplementary publication number CCDC 2378974.

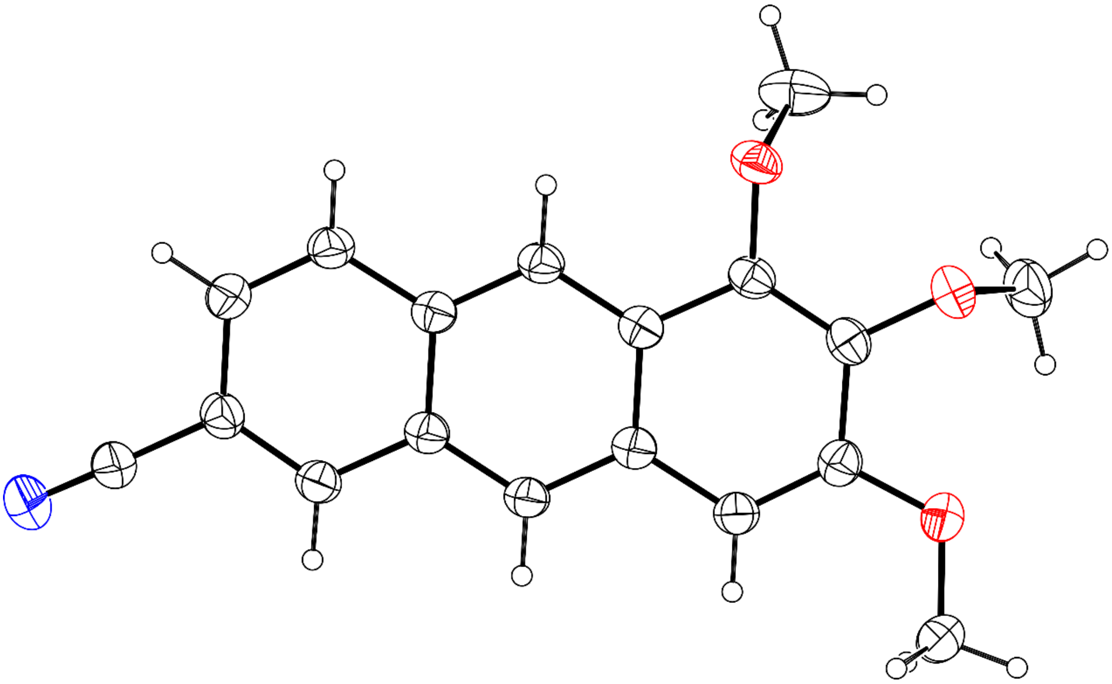

Figure S83. The molecular structures of 9; displacement ellipsoids are drawn at the 30% probability level.

Table S2. Geometrical parameters of intermolecular interactions in the crystal structures of **4b·DCM** and **4j·DCM**.

**4b·DCM**				
**WEAK HYDROGEN BONDS**				
D-H···A	D-H (Å)	H···A (Å)	D···A (Å)	D-H···A (deg)
C31-H31A···O6	0.97	2.56	3.372(4)	141.6(2)
C15-H15B···O6$^{(i)}$	0.93	2.54	3.443(2)	163.7(1)
C31-H31B···Cg$^{(ii)}$	0.97	2.58	3.299(4)	131.2(2)
Cg are centroids of benzene rings. Symmetry codes: (i)1-x, 1-y, -z. (ii) 1/2+x, 1/2-y,-1/2+z				
**4j·DCM**				
**WEAK HYDROGEN BONDS**				
D-H···A	D-H (Å)	H···A (Å)	D···A (Å)	D-H···A (deg)
C31-H31A···O3	0.97	2.56	3.372(4)	141.6(2)
C20-H20···O5$^{(i)}$	0.93	2.54	3.443(2)	163.7(1)
C31-H31A···F1$^{(i)}$	0.97	2.58	3.299(4)	131.2(2)
**π···π INTERACTIONS**				
π···π	Cg ···Cg (Å)	Dihedral angle α (deg)	Slip angle Θ (deg)	Slippage distance S (Å)
Cg1···Cg2$^{(i)}$	3.793(1)	3.8(1)	22.2	1.431
Cg2···Cg1$^{(i)}$	3.793(1)	3.8(1)	25.8	1.652
Cg2···Cg2$^{(ii)}$	3.761(1)	0.0(1)	27.0	1.707
Cg3···Cg3$_{(i)}$	3.860(1)	0.02(9)	26.0	1.693
**X-Y···π INTERACTION**				
X-Y···Cg	X-Y (Å)	Y···Cg (Å)		X-Y···Cg(deg)
C31-Cl2···Cg1$^{(iii)}$	1.723(4)	3.878(2)		145.7(1)
Cg are centroids of benzene rings. Symmetry codes: (i) 1-x, -y, 2-z. (ii) -x, -y,2-z.(iii) 1+x,y,z				

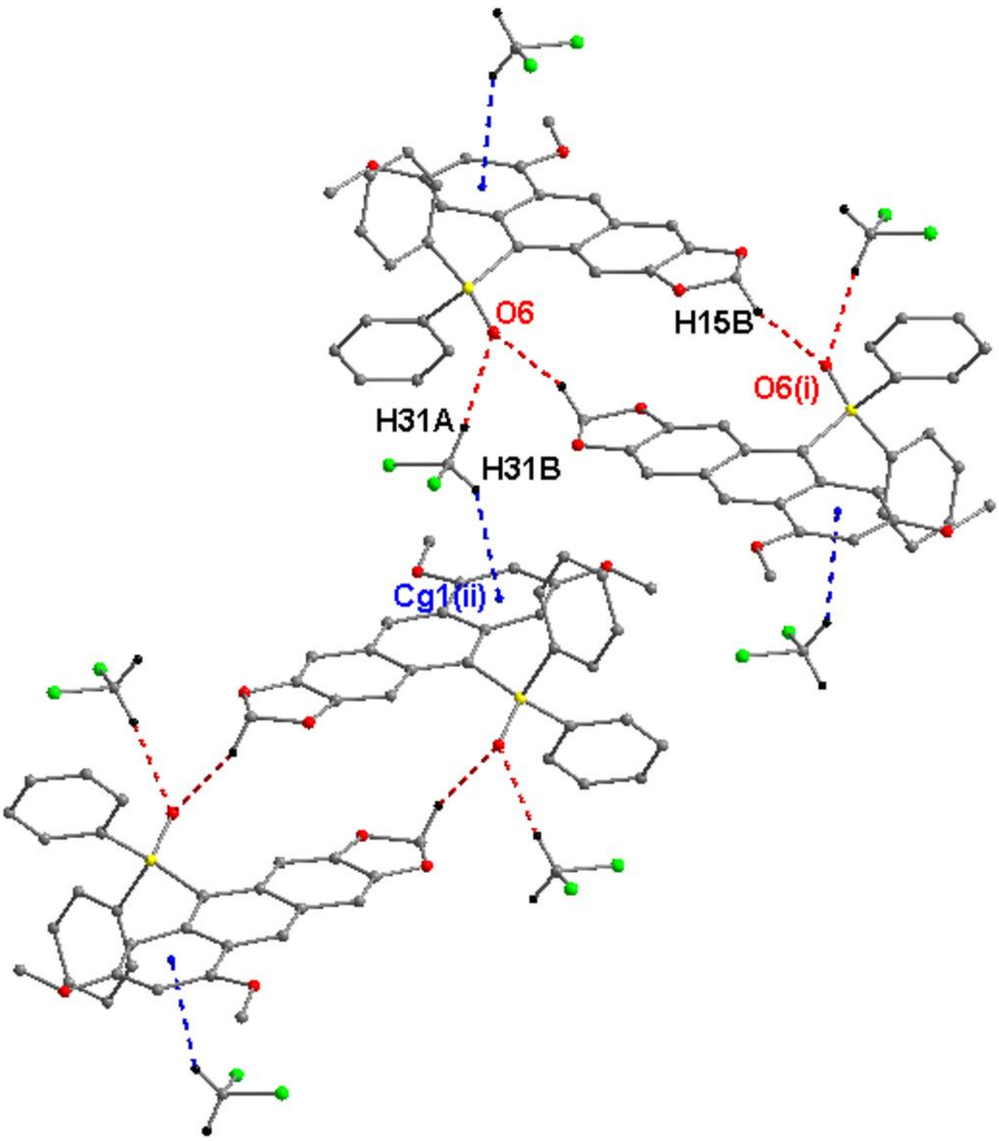

Figure S84 (enlarged Figure 6a). *Part of the crystal structure of* ***4b·DCM*** *the C-H···O and C-H···π hydrogen bonds (red and blue dashed lines, respectively). The centroids of benzene rings (Cg) are drawn with blue small spheres. Symmetry codes: (i)1-x, -y, 2-z. (ii) -x, -y,2-z.(iii) 1+x,y,z, (iv) x, -1+y, z.*

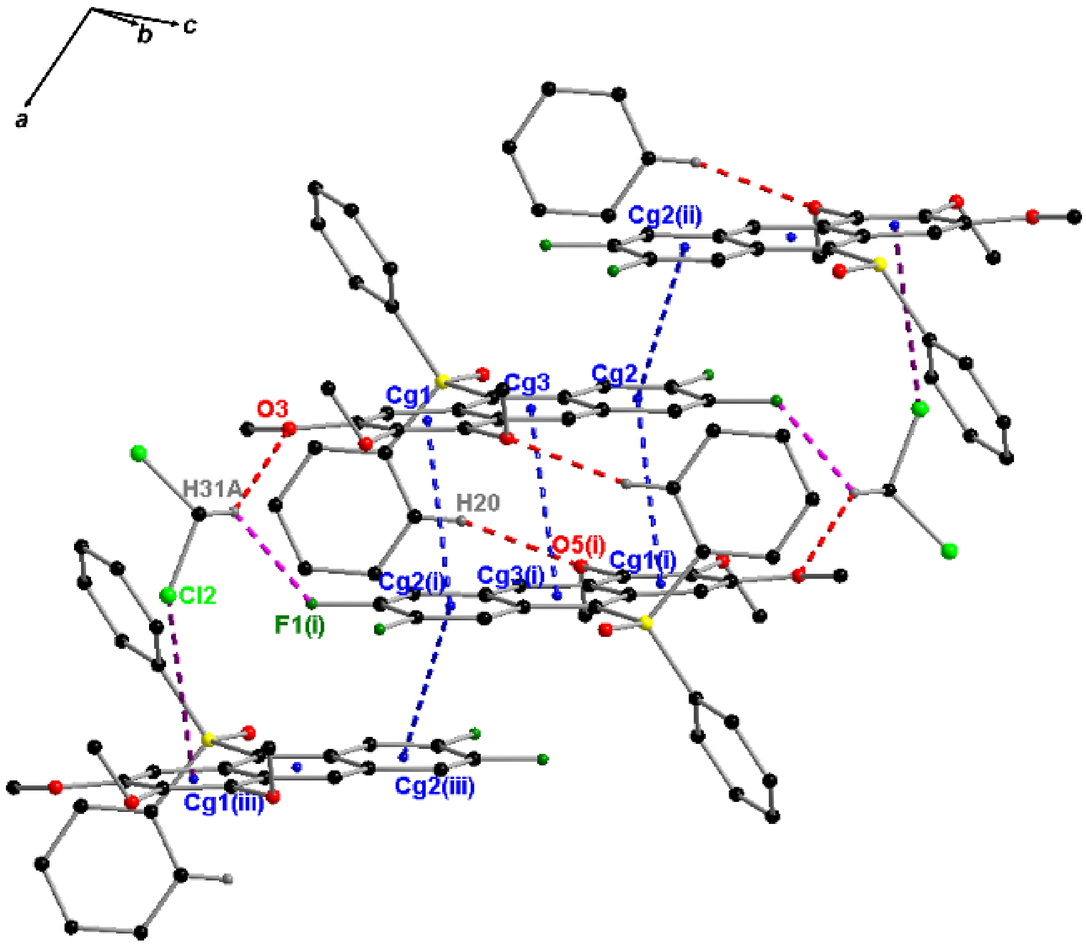

Figure S85 (enlarged Figure 5a). *Part of the crystal structure of* ***4j·DCM*** *showing the π···π interactions (blue dashed lines), C-H···O and C-H···F hydrogen bonds (red and pink dashed lines, respectively), and also C-Cl···π interaction (violet dashed line). The centroids of benzene rings (Cg) are drawn with blue small spheres. Symmetry codes: (i)1-x, -y, 2-z. (ii) -x, -y,2-z.(iii) 1+x,y,z, (iv) x, -1+y, z.*

6. Computational studies:

The molecular and electronic structures of **4a-4j**, **6**, **7**, **9** and **10** were calculated by DFT method using the gradient corrected three-parameter hybrid functional (B3LYP) with the 6-31++G(d,p) basis set. Full geometry optimizations of compounds in the gas phase were performed using GAUSSIAN09 quantum chemistry package.[52] In order to check the structural optimizations, the calculated vibrational frequencies of the compounds were used (no imaginary frequencies). To visualize the shapes of frontier molecular orbitals (HOMO and LUMO) Chemcraft program[53] was used.

Table S3. Atom coordinates (Å) for the geometry of **4a** optimized at the B3LYP/6-311++(d,p) level in the gas phase using Gaussian 09.

Atom	X	Y	Z
6	4.336404	0.135639	-0.300747
6	3.603578	1.292130	-0.171002
6	2.163314	1.253419	-0.119642
6	1.469039	-0.007293	-0.194610
6	2.266457	-1.183288	-0.325059
6	3.632987	-1.114626	-0.388992
6	1.443977	2.436825	0.006295
6	0.040121	-0.013930	-0.133498
6	-0.678721	1.204886	0.030510
6	0.053702	2.453601	0.088377
6	-0.620704	3.713330	0.243823
1	-0.049179	4.632378	0.271300
6	-1.968933	3.701568	0.353896
6	-2.695734	2.490192	0.320870
1	1.987355	3.371691	0.048485
1	1.768807	-2.133824	-0.420552
8	4.144699	2.545759	-0.150312
8	5.693038	0.200375	-0.484192
8	4.433783	-2.198162	-0.576234
15	-0.867599	-1.623565	-0.297957
8	-0.051214	-2.790551	-0.793330
6	5.335817	2.804670	0.598977
1	5.301258	2.297022	1.568236
1	6.225172	2.495059	0.051495
1	5.352623	3.883310	0.758603
6	6.526247	-0.452568	0.484517
1	6.335149	-0.053489	1.487075
1	6.368342	-1.530563	0.479089

1	7.552005	-0.226507	0.193872
6	3.813873	-3.470060	-0.772848
1	3.247874	-3.775397	0.111927
1	3.145009	-3.452243	-1.637207
1	4.630681	-4.168166	-0.948544
6	-2.110964	1.275068	0.169308
1	-2.734988	0.397433	0.154269
6	-2.249441	-1.343353	-1.478767
6	-3.555567	-1.775201	-1.227786
6	-1.935251	-0.807343	-2.734254
6	-4.535192	-1.663308	-2.214037
1	-3.817622	-2.190410	-0.261716
6	-2.914450	-0.696758	-3.716629
1	-0.924219	-0.475637	-2.943317
6	-4.217133	-1.123759	-3.457991
1	-5.545878	-1.997618	-2.007864
1	-2.661177	-0.277974	-4.684157
1	-4.979682	-1.036482	-4.223902
6	-1.588319	-2.037090	1.343257
6	-2.013963	-3.362905	1.515396
6	-1.645195	-1.157643	2.429505
6	-2.520689	-3.787500	2.740599
1	-1.920498	-4.062823	0.692939
6	-2.145641	-1.588227	3.657817
1	-1.288147	-0.141129	2.327598
6	-2.591322	-2.898836	3.813257
1	-2.848303	-4.814108	2.861253
1	-2.179738	-0.899285	4.494455
1	-2.979545	-3.231042	4.769754
8	-2.845774	4.749727	0.479055
8	-4.032260	2.771011	0.436546
6	-4.115154	4.159665	0.775628
1	-4.885870	4.634475	0.169394
1	-4.322475	4.259682	1.847570

Table S4. Atom coordinates (Å) for the geometry of **4b** optimized at the B3LYP/6-311++(d,p) level in the gas phase using Gaussian 09.

Atom	X	Y	Z
6	4.713824	0.039259	-0.135019
6	4.010344	1.201825	-0.010179
6	2.567050	1.199243	0.013076

6	1.858228	-0.051998	-0.110481
6	2.633791	-1.246709	-0.238619
6	4.003101	-1.189267	-0.248993
6	1.870139	2.391325	0.154310
6	0.428109	-0.028417	-0.089581
6	-0.271283	1.198309	0.105925
6	0.478492	2.432303	0.214319
6	-0.176072	3.697600	0.399568
1	0.410332	4.605253	0.465821
6	-1.525933	3.705089	0.491545
6	-2.270638	2.507233	0.413234
1	2.428274	3.315483	0.233524
1	2.120959	-2.186130	-0.356156
8	4.579450	2.434737	0.104795
8	4.814920	-2.274847	-0.371137
15	-0.509189	-1.605618	-0.368331
8	0.294018	-2.759218	-0.911880
6	5.998184	2.528424	0.086178
1	6.442983	1.977041	0.921101
1	6.407406	2.153703	-0.857796
1	6.227637	3.587960	0.186904
6	4.207276	-3.558743	-0.508775
1	3.612888	-3.809770	0.374895
1	3.567409	-3.601464	-1.394550
1	5.030781	-4.263547	-0.611185
6	-1.703232	1.287679	0.230871
1	-2.340570	0.420837	0.187186
6	-1.851087	-1.219518	-1.565862
6	-3.176345	-1.621484	-1.372288
6	-1.486699	-0.627588	-2.782003
6	-4.125199	-1.424362	-2.375128
1	-3.477316	-2.079909	-0.437588
6	-2.435203	-0.431558	-3.780999
1	-0.460225	-0.319423	-2.947238
6	-3.757200	-0.828835	-3.579135
1	-5.150966	-1.736332	-2.213063
1	-2.142876	0.030093	-4.717543
1	-4.495811	-0.675232	-4.357981
6	-1.284199	-2.094524	1.226402
6	-1.760847	-3.411604	1.308647
6	-1.331983	-1.281290	2.363521
6	-2.307574	-3.890591	2.495964
1	-1.675970	-4.063699	0.446856
6	-1.872827	-1.766566	3.553752
1	-0.935760	-0.274585	2.331209
6	-2.368467	-3.066688	3.619804
1	-2.673865	-4.909904	2.547546
1	-1.899071	-1.129079	4.430487

1	-2.787746	-3.442080	4.546767
8	-2.387843	4.762813	0.639771
8	-3.603389	2.804694	0.525345
6	-3.668738	4.183779	0.906304
1	-4.426347	4.688474	0.307783
1	-3.885061	4.253629	1.978816
1	5.793440	0.003560	-0.154121

Table S5. Atom coordinates (Å) for the geometry of **4c** optimized at the B3LYP/6-311++(d,p) level in the gas phase using Gaussian 09.

6	4.363643	-2.617704	0.073423
6	3.189026	-3.300323	-0.054392
6	1.922834	-2.607259	-0.062512
6	1.899777	-1.171093	0.079596
6	3.153085	-0.494778	0.210239
6	4.327999	-1.200450	0.205765
6	0.739781	-3.318727	-0.207050
6	0.632646	-0.507335	0.073460
6	-0.569431	-1.247338	-0.125628
6	-0.502086	-2.688148	-0.252849
6	-1.683207	-3.483596	-0.442494
1	-1.602948	-4.560246	-0.522961
6	-2.872516	-2.843193	-0.520397
6	-2.952850	-1.436154	-0.423631
1	0.787256	-4.396179	-0.300329
1	3.152882	0.573910	0.341499
8	5.560893	-0.637225	0.329065
15	0.565710	1.322352	0.376040
8	1.825669	1.944016	0.921400
6	5.642372	0.779027	0.484483
1	5.236217	1.295140	-0.390646
1	5.105336	1.111299	1.377253
1	6.703226	1.002579	0.584537
6	-1.870163	-0.639135	-0.236367
1	-2.014784	0.426339	-0.178295
6	-0.791415	1.609968	1.584068
6	-1.763695	2.599421	1.407966
6	-0.748701	0.900301	2.791009
6	-2.686233	2.867425	2.418890
1	-1.813219	3.157916	0.480587
6	-1.670428	1.169145	3.798095
1	0.005971	0.136517	2.942738

6	-2.642129	2.153027	3.613628
1	-3.438447	3.634171	2.270357
1	-1.630028	0.611930	4.727380
1	-3.360339	2.361531	4.398766
6	0.111276	2.142867	-1.206076
6	0.322572	3.528405	-1.271596
6	-0.325429	1.466263	-2.349828
6	0.065850	4.225769	-2.448812
1	0.713383	4.049308	-0.404969
6	-0.573991	2.166372	-3.529964
1	-0.459074	0.392363	-2.330631
6	-0.387405	3.545900	-3.579244
1	0.231715	5.296667	-2.487478
1	-0.906402	1.630436	-4.412017
1	-0.580506	4.087990	-4.498388
8	-4.136193	-3.357661	-0.669003
8	-4.265988	-1.058286	-0.524427
6	-4.985151	-2.233024	-0.916945
1	-5.888932	-2.321390	-0.315043
1	-5.213811	-2.177104	-1.987713
1	5.328797	-3.103112	0.081538
1	3.206826	-4.365887	-0.150084

Table S6. Atom coordinates (Å) for the geometry of **4d** optimized at the B3LYP/6-311++(d,p) level in the gas phase using Gaussian 09.

Atom	X	Y	Z
6	4.004827	-0.386008	-0.399699
6	3.564089	0.865836	-0.044455
6	2.152637	1.147243	0.076701
6	1.180396	0.104940	-0.166207
6	1.683089	-1.179275	-0.535709
6	3.026754	-1.410127	-0.658326
6	1.732706	2.420667	0.431463
6	-0.207074	0.406303	-0.026170
6	-0.618020	1.715887	0.366041
6	0.379921	2.733572	0.585237
6	-0.006840	4.051624	0.970440
1	0.775370	4.789035	1.117750
6	-1.320363	4.380587	1.155425
6	-2.312393	3.386127	0.964807
1	2.476463	3.188886	0.598504

1	0.976576	-1.960110	-0.763714
1	-3.356551	3.634854	1.119535
8	4.378062	1.942122	0.155622
8	5.335766	-0.604741	-0.640065
8	3.553188	-2.595267	-1.064452
15	-1.467365	-0.913103	-0.369529
8	-0.953966	-2.140283	-1.080418
6	5.623638	1.791615	0.844174
1	5.510712	1.126843	1.706244
1	6.400035	1.409745	0.182557
1	5.887153	2.790846	1.192199
6	6.004752	-1.582207	0.170333
1	5.945134	-1.308137	1.229695
1	5.583083	-2.575460	0.020150
1	7.046451	-1.569613	-0.149383
6	2.653820	-3.645559	-1.425626
1	2.046264	-3.956277	-0.571034
1	1.994589	-3.335131	-2.240251
1	3.285310	-4.470408	-1.751233
1	-1.605214	5.384271	1.449021
6	-1.976780	2.111940	0.587346
1	-2.776061	1.399614	0.460427
6	-2.782507	-0.151183	-1.403441
6	-4.143230	-0.327115	-1.132926
6	-2.397547	0.499715	-2.582383
6	-5.104628	0.151106	-2.022638
1	-4.458854	-0.829123	-0.225876
6	-3.358832	0.976533	-3.468355
1	-1.345521	0.635048	-2.807927
6	-4.714798	0.804095	-3.189699
1	-6.157256	0.012170	-1.802278
1	-3.049867	1.482513	-4.376149
1	-5.463104	1.176326	-3.880482
6	-2.217313	-1.410652	1.234926
6	-2.886398	-2.643832	1.243675
6	-2.093608	-0.694851	2.430535
6	-3.449848	-3.132127	2.419557
1	-2.939052	-3.225650	0.330625
6	-2.652177	-1.190654	3.608056
1	-1.553209	0.242247	2.454163
6	-3.336779	-2.403969	3.603683
1	-3.966251	-4.085605	2.413608

1	-2.545189	-0.629391	4.529606
1	-3.769895	-2.786993	4.521104

Table S7. Atom coordinates (Å) for the geometry of **4e** optimized at the B3LYP/6-311++(d,p) level in the gas phase using Gaussian 09.

Atom	X	Y	Z
6	4.086622	-0.357305	-0.369286
6	3.569446	0.894528	-0.138570
6	2.142546	1.102308	-0.055898
6	1.236421	-0.014635	-0.205514
6	1.817750	-1.296405	-0.447281
6	3.173600	-1.458103	-0.537494
6	1.646557	2.377488	0.171724
6	-0.167018	0.216131	-0.105060
6	-0.657220	1.530393	0.159940
6	0.276191	2.622687	0.287170
6	-0.186333	3.948035	0.543703
1	0.523802	4.761437	0.630350
6	-1.517704	4.176113	0.684591
6	-2.464418	3.137280	0.590785
1	2.343148	3.199682	0.268454
1	1.161634	-2.135901	-0.606456
1	-3.515441	3.363810	0.721628
8	4.316203	2.031191	-0.036845
8	5.430358	-0.518410	-0.577204
8	3.773436	-2.642334	-0.826771
15	-1.341975	-1.206233	-0.330423
8	-0.749747	-2.452035	-0.939152
6	5.559010	2.023387	0.673249
1	5.471904	1.445370	1.598619
1	6.364514	1.621151	0.060471
1	5.760993	3.066456	0.918247
6	6.144713	-1.375618	0.326153
1	6.061473	-1.003778	1.353596
1	5.778485	-2.400124	0.271281
1	7.186620	-1.336966	0.009528
6	2.941783	-3.770862	-1.105483
1	2.340365	-4.043261	-0.233605
1	2.278255	-3.569995	-1.950450
1	3.624852	-4.581700	-1.352535
6	-2.040427	1.859680	0.336916

1	-2.794161	1.091819	0.275181
6	-2.702845	-0.619670	-1.417355
6	-4.049369	-0.859771	-1.125626
6	-2.360203	-0.042345	-2.646647
6	-5.039784	-0.515173	-2.045033
1	-4.331739	-1.307641	-0.180072
6	-3.350472	0.301160	-3.561835
1	-1.318889	0.140251	-2.888114
6	-4.692654	0.066634	-3.262128
1	-6.081304	-0.702517	-1.808552
1	-3.075190	0.751403	-4.508926
1	-5.463435	0.335483	-3.975710
6	-2.057517	-1.610352	1.315005
6	-2.618783	-2.890074	1.439763
6	-2.011278	-0.774739	2.436203
6	-3.153579	-3.309810	2.654887
1	-2.609815	-3.558609	0.586606
6	-2.541027	-1.201207	3.653452
1	-1.552387	0.203103	2.372770
6	-3.119389	-2.463933	3.763081
1	-3.585789	-4.300703	2.739132
1	-2.494503	-0.546974	4.516847
1	-3.530353	-2.792804	4.711145
9	-1.973679	5.430252	0.931803

Table S8. Atom coordinates (Å) for the geometry of **4f** optimized at the B3LYP/6-311++(d,p) level in the gas phase using Gaussian 09.

Atom	X	Y	Z
6	4.573918	0.324260	-0.245868
6	3.798846	1.452165	-0.117148
6	2.358224	1.359885	-0.086960
6	1.710791	0.069626	-0.182350
6	2.553971	-1.073741	-0.311859
6	3.917041	-0.952521	-0.355666
6	1.597663	2.513329	0.037969
6	0.286585	0.004416	-0.141372
6	-0.473003	1.198709	0.027305
6	0.203529	2.467997	0.103894
6	-0.544742	3.672683	0.261371
1	-0.000871	4.610086	0.303326
6	-1.904174	3.659754	0.366711

6	-2.579979	2.409896	0.323727
1	2.101790	3.469050	0.094892
1	2.092686	-2.040772	-0.424727
8	4.287726	2.724061	-0.075732
8	5.927248	0.439755	-0.409796
8	4.759297	-2.000294	-0.542140
15	-0.564223	-1.638058	-0.347178
8	0.312144	-2.757585	-0.848213
6	5.477440	3.022912	0.663232
1	5.476022	2.501096	1.625267
1	6.371320	2.758974	0.100082
1	5.448824	4.098778	0.837296
6	6.777473	-0.199971	0.554889
1	6.549216	0.158796	1.564556
1	6.675081	-1.283609	0.514394
1	7.794131	0.087676	0.288672
6	4.192524	-3.295705	-0.757008
1	3.629413	-3.629672	0.118835
1	3.534279	-3.296016	-1.629349
1	5.039056	-3.958211	-0.928241
1	-2.464647	4.577954	0.490430
6	-1.898502	1.233485	0.159030
1	-2.470080	0.321200	0.140508
6	-1.931525	-1.377194	-1.545404
6	-3.240361	-1.807138	-1.305732
6	-1.602080	-0.849616	-2.801098
6	-4.208134	-1.702036	-2.304162
1	-3.514898	-2.212769	-0.339237
6	-2.570140	-0.745032	-3.794741
1	-0.588233	-0.521635	-3.002543
6	-3.875590	-1.170730	-3.547554
1	-5.221797	-2.031357	-2.105955
1	-2.306470	-0.331749	-4.761768
1	-4.629848	-1.086581	-4.321847
6	-1.295124	-2.099284	1.273943
6	-1.694579	-3.437516	1.410783
6	-1.392215	-1.243445	2.376115
6	-2.214796	-3.897330	2.617155
1	-1.573871	-4.118397	0.576014
6	-1.907046	-1.709315	3.585354
1	-1.056297	-0.217166	2.304370
6	-2.326194	-3.032095	3.705459

1	-2.523531	-4.932524	2.710466
1	-1.975334	-1.037395	4.433439
1	-2.727402	-3.391427	4.646561
6	-4.075219	2.411345	0.475130
9	-4.457852	2.983460	1.647242
9	-4.679571	3.127859	-0.506999
9	-4.615259	1.175601	0.449580

Table S9. Atom coordinates (Å) for the geometry of **4g** optimized at the B3LYP/6-311++(d,p) level in the gas phase using Gaussian 09.

Atom	X	Y	Z
6	4.255078	-0.067232	-0.309392
6	3.651981	1.146730	-0.082764
6	2.213482	1.255874	-0.017706
6	1.385554	0.080725	-0.187948
6	2.054453	-1.156955	-0.425124
6	3.420061	-1.226538	-0.493969
6	1.629698	2.492368	0.214020
6	-0.031923	0.213602	-0.109383
6	-0.609108	1.489210	0.167619
6	0.245413	2.640933	0.318071
6	-0.315327	3.924854	0.586755
1	0.361026	4.767281	0.681949
6	-1.659045	4.103470	0.731386
6	-2.516285	2.969808	0.613617
1	2.267050	3.359505	0.325902
1	1.458614	-2.037514	-0.599005
8	4.315092	2.331396	0.033189
8	5.607958	-0.137469	-0.498781
8	4.102165	-2.364769	-0.775600
15	-1.108388	-1.280318	-0.391242
8	-0.413420	-2.461038	-1.019478
6	5.561749	2.407538	0.734079
1	5.523887	1.817851	1.655205
1	6.388751	2.069342	0.111666
1	5.688736	3.460639	0.985709
6	6.368125	-0.946798	0.412416
1	6.255337	-0.576603	1.437532
1	6.065859	-1.991856	0.358840
1	7.407384	-0.844960	0.101755
6	3.355155	-3.548194	-1.071400

1	2.758235	-3.862803	-0.210969
1	2.696186	-3.391584	-1.928978
1	4.097605	-4.308828	-1.305807
1	-2.082416	5.077747	0.939163
6	-2.007947	1.717199	0.340663
1	-2.709769	0.903284	0.266101
6	-2.482277	-0.751478	-1.488114
6	-3.824472	-1.013582	-1.195279
6	-2.147174	-0.185309	-2.725065
6	-4.819030	-0.700517	-2.121213
1	-4.101526	-1.450632	-0.243168
6	-3.141981	0.126208	-3.646474
1	-1.108797	0.010787	-2.968754
6	-4.480050	-0.129622	-3.345333
1	-5.857445	-0.898752	-1.881451
1	-2.873679	0.568307	-4.599366
1	-5.254713	0.117065	-4.062536
6	-1.821717	-1.766280	1.230255
6	-2.344068	-3.066464	1.304807
6	-1.808728	-0.969999	2.380474
6	-2.872996	-3.545051	2.500190
1	-2.312422	-3.703865	0.428654
6	-2.333477	-1.455160	3.577706
1	-1.380563	0.023630	2.356277
6	-2.872385	-2.738154	3.637783
1	-3.276262	-4.550488	2.545878
1	-2.316243	-0.829849	4.463157
1	-3.280938	-3.112444	4.569818
6	-3.926585	3.136839	0.791276
7	-5.062783	3.288055	0.942097

Table S10. Atom coordinates (Å) for the geometry of **4h** optimized at the B3LYP/6-311++(d,p) level in the gas phase using Gaussian 09.

Atom	X	Y	Z
6	4.28044	1.02078	-0.33612
6	3.25745	1.93288	-0.23801
6	1.88104	1.49744	-0.18632
6	1.56197	0.08766	-0.23439
6	2.65568	-0.82336	-0.33525
6	3.94934	-0.37928	-0.39555
6	0.86708	2.43822	-0.08538

6	0.19435	-0.31484	-0.17698
6	-0.83135	0.66614	-0.03844
6	-0.47604	2.06228	-0.00319
6	-1.48122	3.06675	0.12810
1	-1.17769	4.10622	0.14265
6	-2.79314	2.71419	0.23912
6	-3.17629	1.35174	0.23168
1	1.12779	3.48805	-0.06191
1	2.44280	-1.87627	-0.41421
1	-4.22033	1.08452	0.33240
8	3.42311	3.28597	-0.24807
8	5.56812	1.44800	-0.52022
8	5.01987	-1.19968	-0.55377
15	-0.22621	-2.12170	-0.29713
8	0.87622	-3.00932	-0.81675
6	4.51756	3.89393	0.44637
1	4.66013	3.42854	1.42662
1	5.43888	3.83078	-0.13088
1	4.23154	4.93724	0.58179
6	6.53933	1.07988	0.47095
1	6.24830	1.46599	1.45423
1	6.66427	-0.00123	0.51899
1	7.47272	1.54761	0.15904
6	4.78173	-2.59950	-0.72011
1	4.31454	-3.02867	0.17074
1	4.14382	-2.78902	-1.58703
1	5.76297	-3.04476	-0.87472
6	-2.22699	0.37343	0.09729
1	-2.57109	-0.64832	0.09932
6	-1.67366	-2.27747	-1.41720
6	-2.77852	-3.07362	-1.09885
6	-1.58952	-1.69767	-2.68943
6	-3.79202	-3.27495	-2.03533
1	-2.85681	-3.53260	-0.12024
6	-2.60265	-1.89963	-3.62151
1	-0.73213	-1.08743	-2.95104
6	-3.70696	-2.68733	-3.29540
1	-4.64609	-3.89184	-1.77884
1	-2.53072	-1.44315	-4.60227
1	-4.49613	-2.84366	-4.02229
6	-0.73733	-2.68885	1.37552
6	-0.64993	-4.06958	1.60756

6	-1.13058	-1.84510	2.42014
6	-0.98270	-4.59692	2.85258
1	-0.29786	-4.71902	0.81434
6	-1.45633	-2.37608	3.66756
1	-1.17214	-0.77375	2.27362
6	-1.39035	-3.75096	3.88357
1	-0.91315	-5.66589	3.02065
1	-1.75464	-1.71254	4.47149
1	-1.64360	-4.16085	4.85504
35	-4.15038	4.05642	0.42034

Table S11. Atom coordinates (Å) for the geometry of **4i** optimized at the B3LYP/6-311++(d,p) level in the gas phase using Gaussian 09.

Atom	X	Y	Z
6	4.25959	-0.08094	-0.36577
6	3.63997	1.13064	-0.17627
6	2.20260	1.21376	-0.06068
6	1.39388	0.01852	-0.14525
6	2.07926	-1.21530	-0.34662
6	3.44257	-1.26323	-0.46430
6	1.59919	2.44873	0.11716
6	-0.02379	0.12032	-0.01646
6	-0.59739	1.38251	0.31732
6	0.22304	2.57125	0.32470
6	-0.33943	3.86050	0.55860
1	0.29146	4.73975	0.52595
6	-1.66159	3.98706	0.83844
6	-2.47901	2.84020	0.93967
1	2.21538	3.33782	0.11945
1	1.50183	-2.11400	-0.47508
8	4.28112	2.33260	-0.14077
8	5.60653	-0.13525	-0.59965
8	4.13640	-2.40118	-0.71783
15	-1.05322	-1.35139	-0.53993
8	-0.29923	-2.31377	-1.42209
6	5.54854	2.46660	0.51273
1	5.55457	1.91791	1.45949
1	6.36158	2.11806	-0.12233
1	5.66047	3.53235	0.71315
6	6.41240	-0.88614	0.32181
1	6.32517	-0.47089	1.33207

1	6.13021	-1.93823	0.32664
1	7.43881	-0.77918	-0.02755
6	3.40156	-3.60400	-0.96258
1	2.85454	-3.91864	-0.06906
1	2.69972	-3.47335	-1.78973
1	4.14854	-4.35283	-1.21962
6	-1.95351	1.59931	0.70319
6	-2.47403	-0.69765	-1.50009
6	-3.79941	-1.05361	-1.23434
6	-2.18247	0.05986	-2.64155
6	-4.81954	-0.65273	-2.09465
1	-4.04179	-1.62869	-0.34915
6	-3.20489	0.46696	-3.49447
1	-1.15598	0.32514	-2.86919
6	-4.52514	0.10992	-3.22305
1	-5.84477	-0.93136	-1.87828
1	-2.96972	1.05795	-4.37253
1	-5.32087	0.42499	-3.88882
6	-1.64580	-2.22786	0.95861
6	-2.19311	-3.50455	0.76666
6	-1.47172	-1.74880	2.26026
6	-2.59214	-4.27036	1.85901
1	-2.28562	-3.90310	-0.23722
6	-1.86098	-2.52187	3.35247
1	-1.02494	-0.77673	2.42718
6	-2.42900	-3.77871	3.15375
1	-3.01967	-5.25396	1.69963
1	-1.71894	-2.14189	4.35798
1	-2.73414	-4.37738	4.00483
9	-2.22907	5.18503	1.06744
9	-3.75574	2.97382	1.31915
9	-2.76675	0.55177	0.93830

Table S12. Atom coordinates (Å) for the geometry of **4j** optimized at the B3LYP/6-311++(d,p) level in the gas phase using Gaussian 09.

Atom	X	Y	Z
6	4.229241	-0.148874	-0.340455
6	3.615158	1.065051	-0.147059
6	2.175768	1.162621	-0.080549
6	1.358900	-0.022644	-0.211761
6	2.038382	-1.260694	-0.415038

6	3.404526	-1.319721	-0.487686
6	1.580997	2.400371	0.114625
6	-0.060894	0.100539	-0.131723
6	-0.651244	1.377510	0.101127
6	0.195553	2.540597	0.214091
6	-0.368238	3.831698	0.439532
1	0.277974	4.697894	0.514404
6	-1.711365	3.980940	0.568465
6	-2.553901	2.848962	0.481870
1	2.211371	3.275595	0.198898
1	1.450848	-2.151849	-0.560329
8	4.267740	2.259442	-0.068967
8	5.583225	-0.213173	-0.533681
8	4.096084	-2.460816	-0.738795
15	-1.121565	-1.410849	-0.346565
8	-0.427444	-2.611501	-0.937113
6	5.513377	2.367349	0.628780
1	5.480753	1.804869	1.566998
1	6.343462	2.017775	0.016689
1	5.631748	3.428532	0.849107
6	6.351469	-0.983746	0.402937
1	6.238278	-0.579470	1.415139
1	6.057208	-2.032507	0.386819
1	7.389525	-0.885415	0.086720
6	3.358190	-3.657916	-0.996566
1	2.765998	-3.952013	-0.125531
1	2.695191	-3.532706	-1.856339
1	4.105913	-4.419517	-1.210682
6	-2.056781	1.601226	0.259294
1	-2.774988	0.799148	0.207941
6	-2.515284	-0.939140	-1.446105
6	-3.846252	-1.243024	-1.142908
6	-2.205544	-0.384169	-2.694491
6	-4.854803	-0.983014	-2.070283
1	-4.102899	-1.673440	-0.182057
6	-3.213970	-0.125299	-3.617429
1	-1.175728	-0.154812	-2.945299
6	-4.541052	-0.423034	-3.306235
1	-5.884370	-1.217466	-1.824251
1	-2.964877	0.307873	-4.579599
1	-5.326253	-0.219305	-4.025561
6	-1.815660	-1.844280	1.299339

6	-2.298143	-3.154517	1.436171
6	-1.823706	-0.995386	2.411344
6	-2.809492	-3.593055	2.654603
1	-2.247372	-3.830382	0.590247
6	-2.330209	-1.440187	3.631888
1	-1.425066	0.007971	2.337970
6	-2.830269	-2.734698	3.753591
1	-3.181000	-4.607369	2.748674
1	-2.327770	-0.774892	4.487976
1	-3.223684	-3.077805	4.704019
9	-2.271391	5.186514	0.785743
9	-3.879290	3.035665	0.633008

Table S13. Atom coordinates (Å) for the geometry of **6** optimized at the B3LYP/6-311++(d,p) level in the gas phase using Gaussian 09.

Atom	X	Y	Z
6	3.916426	0.783241	-0.235855
6	3.146445	1.843175	0.183763
6	1.705874	1.788559	0.085222
6	1.062913	0.584049	-0.379537
6	1.895681	-0.477486	-0.835557
6	3.261069	-0.374897	-0.791100
6	0.938721	2.895427	0.424828
6	-0.356360	0.520561	-0.394081
6	-1.115367	1.712143	-0.216819
6	-0.446660	2.905215	0.238975
6	-1.196190	4.097430	0.468182
1	-0.669580	4.971147	0.836168
6	-2.532590	4.160637	0.205758
6	-3.187253	3.009431	-0.326221
1	1.433235	3.784588	0.793006
1	1.445243	-1.336366	-1.304456
8	3.629711	3.032887	0.632596
8	5.279531	0.894658	-0.286490
8	4.111069	-1.303667	-1.291841
15	-1.160347	-1.120445	-0.690771
6	4.839569	3.105044	1.395755
1	4.863054	2.320524	2.158089
1	5.718236	3.028024	0.757729
1	4.812768	4.079708	1.882826
6	6.047757	-0.022064	0.507634

1	5.834400	0.122883	1.572704
1	5.846999	-1.054996	0.224812
1	7.092121	0.217355	0.310604
6	3.563332	-2.442041	-1.960841
1	2.963234	-3.049907	-1.277839
1	2.952900	-2.136527	-2.814866
1	4.420656	-3.015653	-2.307914
1	-3.096897	5.070413	0.365883
6	-2.502379	1.829481	-0.524825
1	-3.032611	1.000334	-0.965042
6	-2.811929	-1.141035	0.144990
6	-2.959892	-0.769280	1.486967
6	-3.919983	-1.600679	-0.569081
6	-4.206317	-0.847402	2.099854
1	-2.111819	-0.410186	2.057081
6	-5.167220	-1.680206	0.050061
1	-3.798307	-1.886292	-1.607763
6	-5.313116	-1.301991	1.381833
1	-4.315207	-0.548866	3.136412
1	-6.023805	-2.031543	-0.513732
1	-6.284495	-1.356856	1.860010
6	-0.255987	-2.337787	0.378799
6	-0.191458	-3.664040	-0.055207
6	0.269457	-1.998359	1.630865
6	0.385815	-4.641043	0.755175
1	-0.588000	-3.922355	-1.030791
6	0.847664	-2.975736	2.436617
1	0.249849	-0.971101	1.974752
6	0.905467	-4.299813	2.001868
1	0.427619	-5.668129	0.409892
1	1.256881	-2.701895	3.402759
1	1.354101	-5.059565	2.632089
16	-1.246156	-1.653004	-2.598926
6	-4.573895	3.094313	-0.669885
7	-5.695274	3.184269	-0.935854

Table S14. Atom coordinates (Å) for the geometry of **7** optimized at the B3LYP/6-311++(d,p) level in the gas phase using Gaussian 09.

Atom	X	Y	Z
6	4.021801	0.739593	-0.231842
6	3.299578	1.853975	0.127058

6	1.855790	1.843079	0.071003
6	1.159377	0.630843	-0.283604
6	1.942927	-0.491574	-0.675772
6	3.311999	-0.433784	-0.676558
6	1.137553	2.999433	0.345646
6	-0.262031	0.619020	-0.256903
6	-0.974392	1.848048	-0.151194
6	-0.251210	3.046138	0.195422
6	-0.950762	4.279417	0.354009
1	-0.382449	5.157432	0.640705
6	-2.291212	4.373447	0.123800
6	-3.001359	3.211197	-0.301896
1	1.672988	3.894839	0.632310
1	1.451939	-1.367818	-1.066128
8	3.834720	3.055793	0.472875
8	5.385419	0.802015	-0.328249
8	4.114604	-1.428428	-1.125595
15	-1.128574	-1.011245	-0.399832
6	5.068790	3.141440	1.194766
1	5.095300	2.406344	2.004649
1	5.925158	2.999310	0.537871
1	5.083033	4.145405	1.618893
6	6.151184	-0.083543	0.502844
1	5.950309	0.115434	1.561557
1	5.935871	-1.126551	0.272885
1	7.195992	0.134409	0.284322
6	3.507704	-2.600161	-1.676251
1	2.914073	-3.126097	-0.923248
1	2.876176	-2.347742	-2.532113
1	4.333617	-3.230186	-2.000906
1	-2.817931	5.313139	0.230100
6	-2.364815	1.994734	-0.429374
1	-2.937654	1.156102	-0.790958
6	-2.767914	-0.906988	0.456869
6	-2.869852	-0.442131	1.774255
6	-3.909329	-1.366768	-0.202076
6	-4.103927	-0.428945	2.416172
1	-1.995383	-0.083369	2.302901
6	-5.143918	-1.353856	0.446446
1	-3.823206	-1.726874	-1.221256
6	-5.244136	-0.884125	1.753009
1	-4.176198	-0.060337	3.433186

1	-6.026203	-1.706447	-0.075335
1	-6.205445	-0.868634	2.253857
6	-0.243774	-2.163449	0.756708
6	-0.248646	-3.527593	0.456411
6	0.336219	-1.724359	1.952468
6	0.314642	-4.443307	1.344292
1	-0.686942	-3.863910	-0.476849
6	0.901051	-2.641504	2.835059
1	0.369385	-0.668313	2.191817
6	0.890149	-4.003237	2.534287
1	0.302899	-5.500355	1.102752
1	1.354270	-2.290769	3.755614
1	1.329370	-4.715714	3.223579
6	-4.394026	3.322135	-0.612261
7	-5.519235	3.433952	-0.852497
34	-1.268189	-1.746659	-2.411890

Table S15. Atom coordinates (Å) for the geometry of **9** optimized at the B3LYP/6-311++(d,p) level in the gas phase using Gaussian 09.

Atom	X	Y	Z
6	4.213122	-0.226674	-0.060669
6	3.650695	1.002224	-0.332684
6	2.217342	1.164044	-0.347545
6	1.386577	0.025072	-0.060885
6	1.995363	-1.234856	0.215408
6	3.357865	-1.360661	0.204387
6	1.626289	2.397531	-0.625639
6	-0.001262	0.185121	-0.066059
6	-0.600482	1.420068	-0.344935
6	0.235588	2.556524	-0.632500
6	-0.385814	3.810462	-0.915417
1	0.245142	4.665473	-1.132890
6	-1.744216	3.946019	-0.916724
6	-2.574918	2.814931	-0.630043
1	2.262335	3.246173	-0.841056
1	1.356504	-2.084870	0.410919
8	4.343963	2.123198	-0.667417
8	5.566308	-0.404207	-0.181970
8	4.029236	-2.527095	0.399992
6	5.621778	2.412471	-0.090120
1	5.612743	2.218848	0.986695

1	6.413518	1.834838	-0.564486
1	5.779667	3.477113	-0.262382
6	6.297102	-0.772609	0.997626
1	6.175587	-0.012975	1.777746
1	5.977626	-1.744309	1.373157
1	7.343106	-0.819006	0.696127
6	3.274228	-3.720523	0.580678
1	2.658811	-3.668012	1.484630
1	2.636760	-3.916690	-0.287048
1	4.005253	-4.520357	0.684059
1	-2.209511	4.899516	-1.132511
6	-2.012960	1.588340	-0.353109
1	-2.649496	0.737685	-0.138329
6	-3.996834	2.968733	-0.633637
7	-5.145220	3.103434	-0.638613
1	-0.632622	-0.671274	0.148841

Table S16. Atom coordinates (Å) for the geometry of **10** optimized at the B3LYP/6-311++(d,p) level in the gas phase using Gaussian 09.

Atom	X	Y	Z
6	-4.806636	0.709785	-0.229951
6	-3.915976	1.755378	-0.187107
6	-2.492683	1.515080	-0.181538
6	-1.613818	2.587991	-0.132825
1	-2.014975	3.592951	-0.115314
6	-0.232048	2.402462	-0.093127
6	0.638760	3.528139	-0.010130
6	1.991888	3.381220	0.059850
6	2.567723	2.074253	0.060487
6	1.739452	0.971357	-0.019491
1	2.206084	0.000413	0.020345
6	0.317722	1.070063	-0.118769
6	-0.569567	-0.046773	-0.202704
6	-1.981755	0.163359	-0.218545
6	-2.942659	-0.891066	-0.265103
6	-4.286954	-0.631273	-0.280803
1	0.194722	4.518017	-0.007867
15	0.087514	-1.779039	-0.305633
8	-0.900085	-2.827093	-0.754640
1	-2.588077	-1.905744	-0.336432
8	-4.270582	3.072360	-0.213073

6	-5.397487	3.536204	0.537499
1	-5.256256	4.612530	0.639716
1	-6.332936	3.329880	0.019245
1	-5.414725	3.078497	1.531619
1	2.635733	4.251789	0.101057
6	4.022630	1.902709	0.140972
6	4.801395	0.896012	-0.379352
16	5.029155	3.057366	0.996296
6	6.188130	1.043678	-0.098942
1	4.394124	0.087401	-0.971518
6	6.466062	2.163196	0.633481
1	6.948264	0.351991	-0.437847
1	7.423219	2.519558	0.982112
8	-6.147708	0.952502	-0.368218
8	-5.238849	-1.595459	-0.382056
6	-7.018534	0.477073	0.669074
1	-6.987410	-0.609356	0.743874
1	-6.748682	0.925107	1.632101
1	-8.020098	0.800412	0.386623
6	-4.814780	-2.952577	-0.526721
1	-4.259808	-3.288375	0.353848
1	-5.729455	-3.533500	-0.631893
1	-4.189058	-3.076499	-1.414148
6	1.489414	-1.767581	-1.494806
6	2.663162	-2.488745	-1.253034
6	1.301797	-1.164975	-2.745243
6	3.638105	-2.598584	-2.244618
1	2.824626	-2.962061	-0.291694
6	2.277682	-1.272819	-3.731596
1	0.392901	-0.609471	-2.947901
6	3.448148	-1.990338	-3.483567
1	4.544070	-3.160933	-2.047085
1	2.124045	-0.798605	-4.694429
1	4.206760	-2.075266	-4.253604
6	0.745541	-2.236639	1.350335
6	0.842234	-3.611760	1.610879
6	1.078248	-1.326850	2.360164
6	1.296473	-4.064907	2.846755
1	0.534874	-4.317618	0.847881
6	1.526386	-1.784375	3.598573
1	0.979462	-0.262211	2.194200
6	1.643894	-3.151300	3.841288

1	1.368270	-5.130166	3.036387
1	1.778307	-1.070154	4.374441
1	1.992900	-3.503721	4.805640

Table S17. HOMO and LUMO orbitals and E_{HOMO}, E_{LUMO} and Eg values calculated for anthracenes **4a-j** in the gas phase at B3LYP/6-311++(d,p) level using Gaussian 09.

Compound	HOMO	LUMO	E_g (eV)
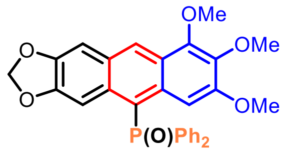 **4a**	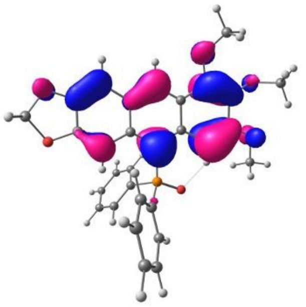 E_{HOMO}=-5.277 eV	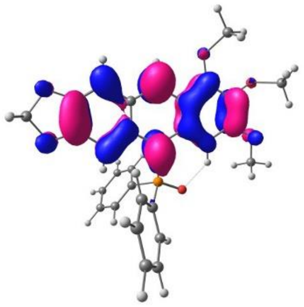 E_{LUMO}=-1.870 eV	3.407
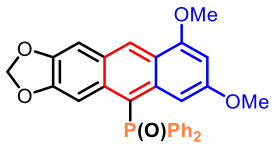 **4b**	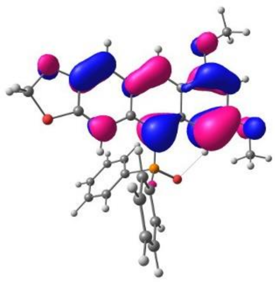 E_{HOMO}=-5.273 eV	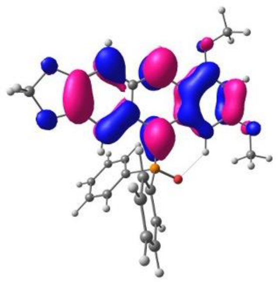 E_{LUMO}=-1.872 eV	3.400
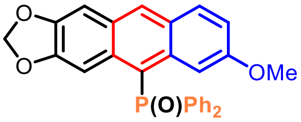 **4c**	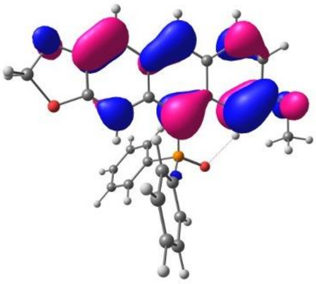 E_{HOMO}=-5.488 eV	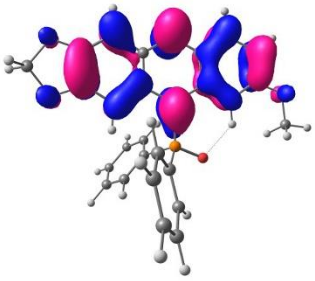 E_{LUMO}=-2.000 eV	3.488
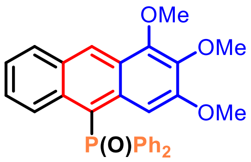 **4d**	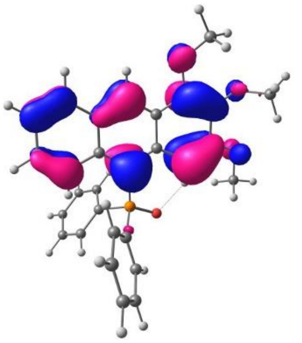 E_{HOMO}=-5.342 eV	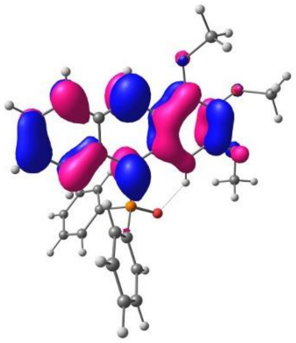 E_{LUMO}=-2.022 eV	3.321

<table>
<tr><td>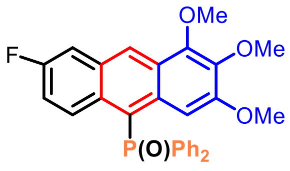

4e</td><td>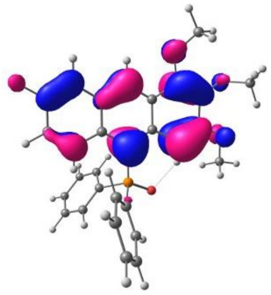
EHOMO=-5.436 eV</td><td>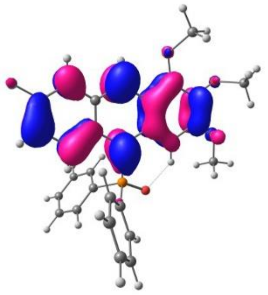
ELUMO=-2.348 eV</td><td>3.088</td></tr>
<tr><td>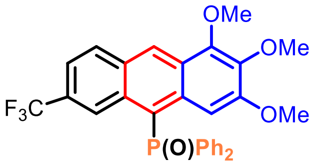

4f</td><td>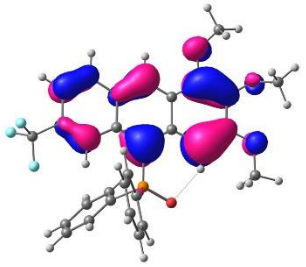
EHOMO=-5.607 eV</td><td>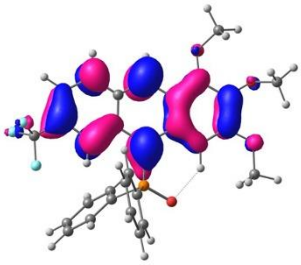
ELUMO=-2.167 eV</td><td>3.440</td></tr>
<tr><td>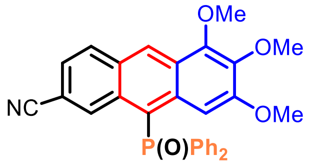

4g</td><td>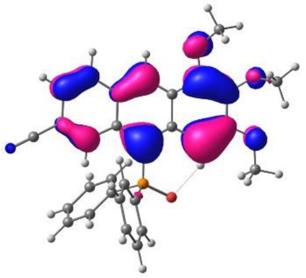
EHOMO=-5.678 eV</td><td>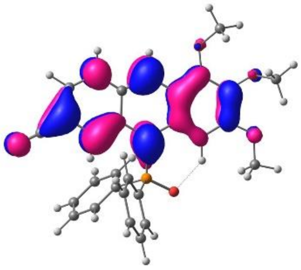
ELUMO=-2.547 eV</td><td>3.131</td></tr>
<tr><td>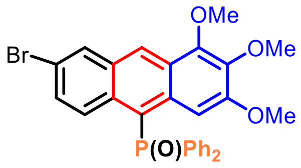

4h</td><td>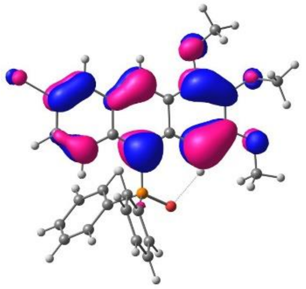
EHOMO=-5.473 eV</td><td>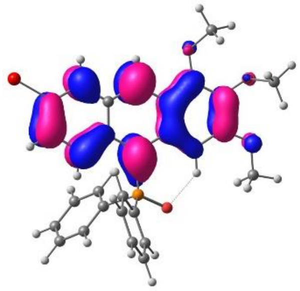
ELUMO=-2.210 eV</td><td>3.264</td></tr>
<tr><td>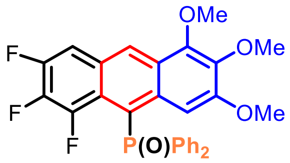

4i</td><td>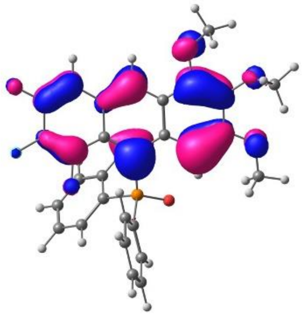
EHOMO=-5.616 eV</td><td>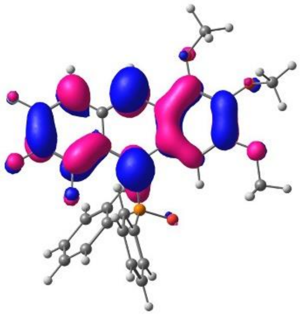
ELUMO=-2.371 eV</td><td>3.245</td></tr>
</table>

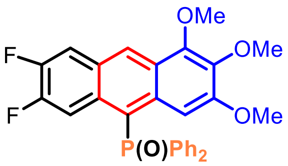 4j	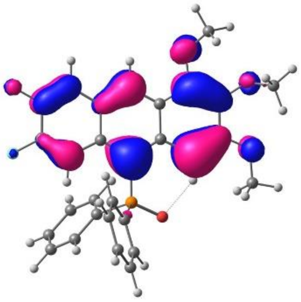 E_{HOMO}=-5.444 eV	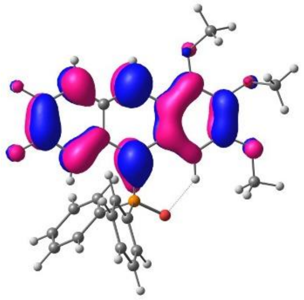 E_{LUMO}=-2.232 eV	3.212

Table S18. HOMO and LUMO orbitals and E_{HOMO}, E_{LUMO} and Eg values calculated for anthracenes **6**, **7**, **9** and **10** in the gas phase at B3LYP/6-311++(d,p) level using Gaussian 09.

Compound	HOMO	LUMO	Eg (eV)
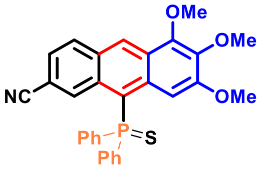 6	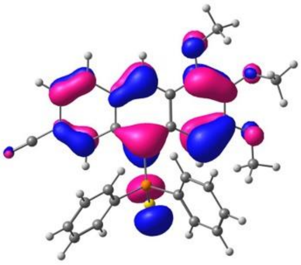 E_{HOMO}=-5.714 eV	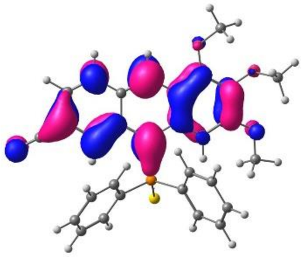 E_{LUMO}=-2.627 eV	3.087
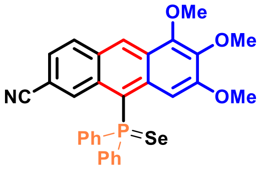 7	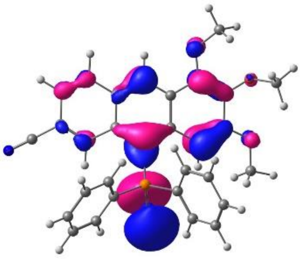 E_{HOMO}=-5.606 eV	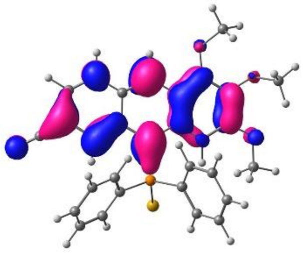 E_{LUMO}=-2.641 eV	2.965
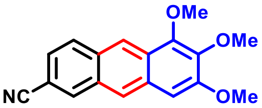 9	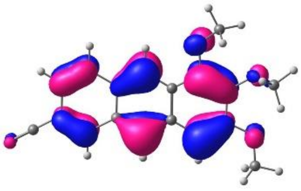 E_{HOMO}=-5.649 eV	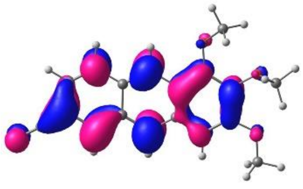 E_{LUMO}=-2.313 eV	3.336
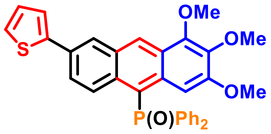 10	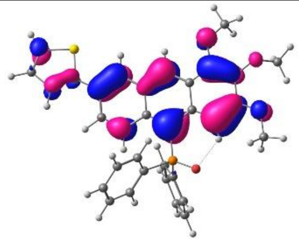 E_{HOMO}=-5.290 eV	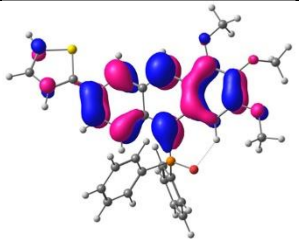 E_{LUMO}=-2.118 eV	3.173

7. CIE 1931 color space coordinates:

Table S19. CIE 1931 color space chromaticity coordinates of **4a-j** and **6-10** in three different solvents.

Nr	**Toluene**		**DCM**		**MeOH**	
	x	y	x	y	x	y
**4a**	0.173	0.248	0.164	0.273	0.224	0.412
**4b**	0.204	0.375	0.211	0.444	0.315	0.537
**4c**	0.154	0.154	0.149	0.162	0.189	0.273
**4d**	0.209	0.405	0.204	0.429	0.309	0.527
**4e**	0.216	0.426	0.212	0.453	0.331	0.525
**4f**	0.274	0.517	0.265	0.545	0.383	0.542
**4g**	0.318	0.567	0.322	0.583	0.443	0.505
**4h**	0.241	0.463	0.227	0.487	0.348	0.530
**4i**	0.299	0.540	0.293	0.562	0.413	0.534
**4j**	0.235	0.438	0.220	0.461	0.338	0.537
**6**	0.383	0.508	0.388	0.553	0.427	0.537
**7**	0.220	0.678	0.289	0.651	0.468	0.510
**9**	0.149	0.121	0.155	0.228	0.185	0.304
**10**	0.222	0.450	0.260	0.595	0.319	0.514

8. Reaction mechanisms

8.1. Reaction mechanism of the TMSOTf catalyzed C-O-P to C-P(=O) rearrangement of 2a-j to 3a-j

The mechanism underlying the Lewis acid rearrangement of phosphinites **2a-j** to phosphine oxides **3a-j** has been performed in literature (ref. 33: *Angew. Chem. Int. Ed.* 2003, *42*, 2389-2392).

Intermolecular S_N2 mechanism involving two phosphinite molecules, in which the P^{III} atom of the neutral phosphinite **II** attacks primary CH_2OP carbon atom of the silylated phosphinite **I** has been proposed by the authors, both for primary $R^3CH_2OPR^1R^2$ and secondary R^3R^4 $CHOPR^1R^2$ phosphinites.

In secondary phosphinites **2a-j**, a steric hindrance of two bulky aryl groups, especially *ortho*-substituted by acetal moiety, significantly slow down the S_N2 attack. Therefore, a simple, unimolecular S_N1 mechanism has been offered below, in which stabilized dibenzylic carbocation **III** is formed followed by reaction of the latter with silylated diphenyl phosphinite, initiated by the TfO^- anion (Scheme 1S).

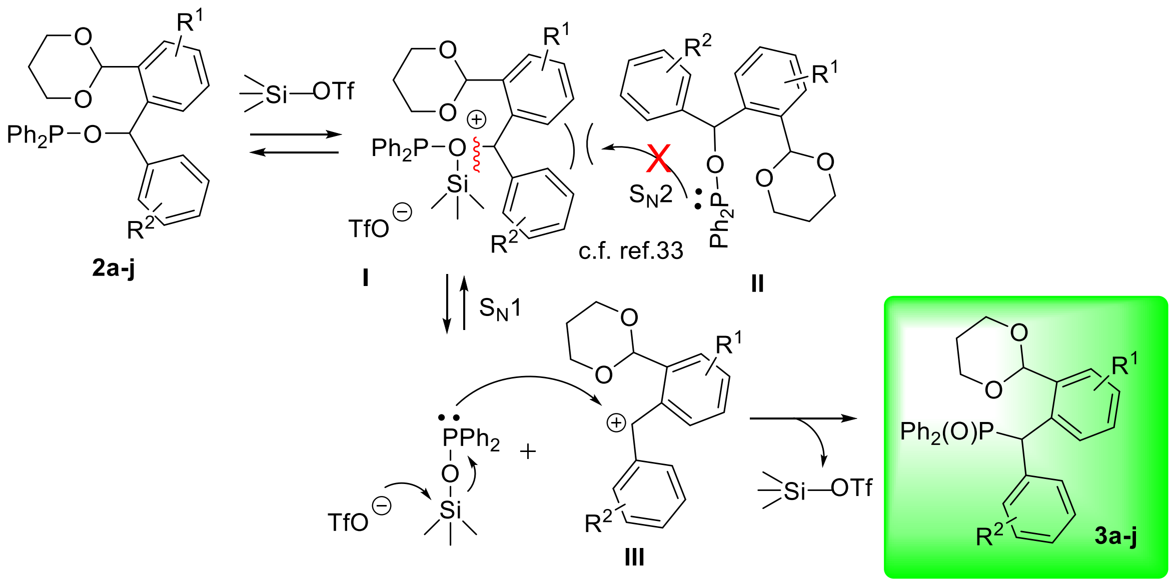

Scheme 1S

8.2. Reaction mechanism of the *phospho*-Friedel-Crafts-Bradsher cyclization of 3a-j to 4a-j

The synthesis of **4a-j** assumes the initial formation of the carbocation **I** from the C-O bond cleavage of the protonated acetal **3a-j** and further proceeds *via* intermediates **II-IV** (**A**, solid lines).

Alternative pathway may include a full deacetalization of **3a-j** to the aldehyde **V** followed by protonation of the latter and cyclization of the resulting carbocation **VI** to **4a-j** *via* the intermediate **VII** (**B**, dotted lines).

However, the mechanism **A** of the cyclization reaction strongly dominates over the mechanism **B**, as the carbocation **I**, which is formed first, preferentially cyclizes compared to the carbocation **VI** (Scheme 2S).

.

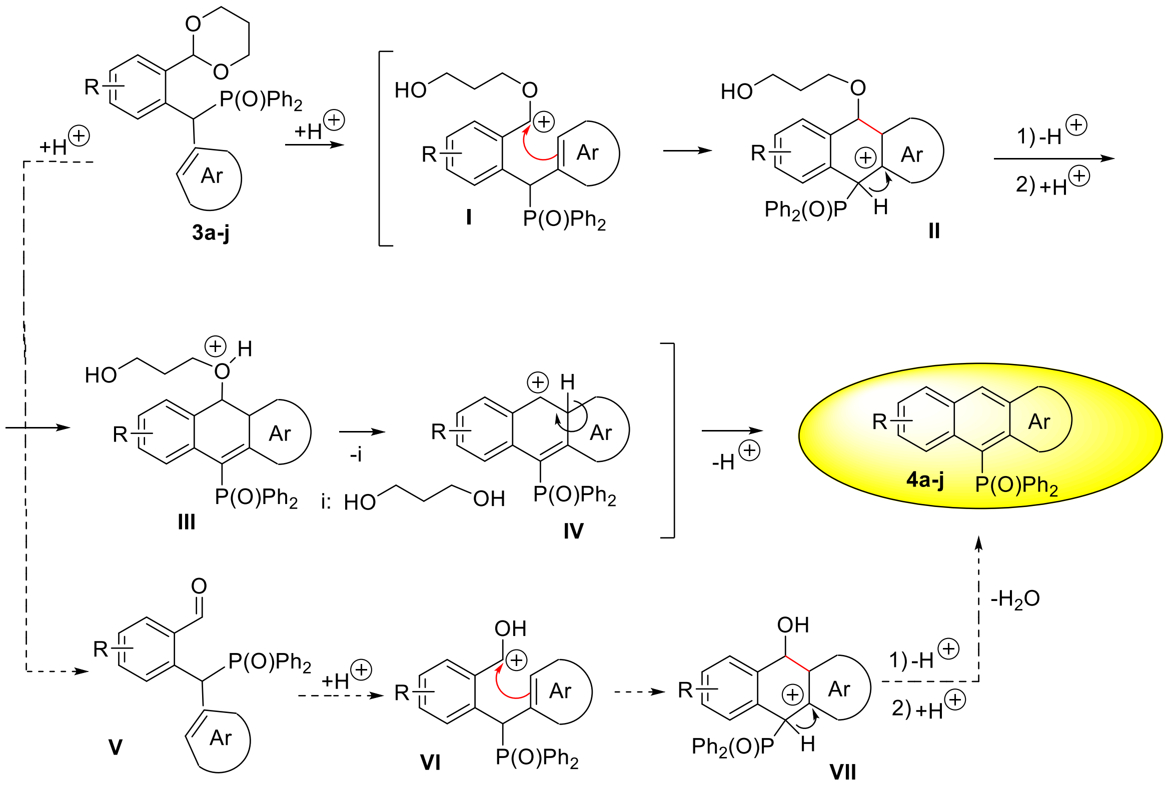

Scheme 2S

8.3. Reaction mechanism of the dephosphorylation of 4g to 9

Transformation of **4g** to **9** was realized as a one-pot synthesis involving deoxygenation of the phosphine oxide **4g** to the phosphine **5** with excess of silane Cl_3SiH[54] followed by quaternization of the resulting phosphine **5** to the corresponding phosphonium iodide with excess of methyl iodide. The latter underwent, *in situ*, the *ipso* attack of the Si-H bond, facilitated by the presence of the 6-CN group to get **9** (Scheme 3S).

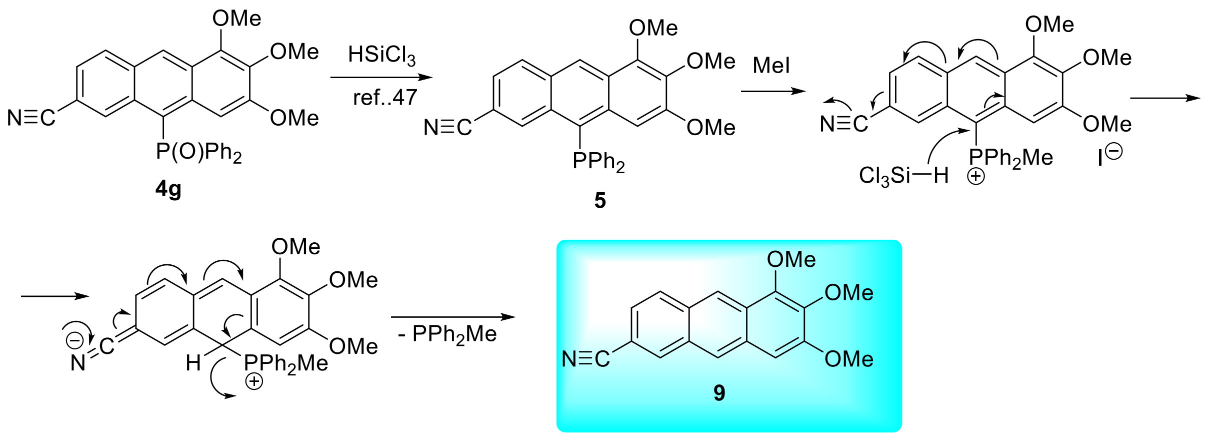

Scheme 3S

9. References

50. Sheldrick G. M. *Acta Crystallogr. C* **2015**, *71*, 3-8.

51. Deposition numbers CCDC 2378975 (for **4b**), CCDC 2378976 (for **4j**), and CCDC 2378974 (for **9**) contain the supplementary crystallographic data for this paper. These data are provided free of charge by the joint Cambridge Crystallographic Data Centre.

52. Frisch M. J.; Trucks G. W.; Schlegel H. B.; Scuseria G. E.; Robb M. A.; Cheeseman J. R.; Scalmani G.; Barone V.; Petersson G. A.; Nakatsuji H.; Li X.; Caricato M.; Marenich A.; Bloino J.; Janesko B. G.; Gomperts R.; Mennucci B.; Hratchian H. P.; Ortiz J. V.; Izmaylov A. F.; Sonnenberg J. L.; Williams-Young D.; Ding F.; Lipparini F.; Egidi F.; Goings J.; Peng B.; Petrone A.; Henderson T.; Ranasinghe D.; Zakrzewski V. G.; Gao J.; Rega N.; Zheng G.; Liang W.; Hada M.; Ehara M.; Toyota K.; Fukuda R.; Hasegawa J.; Ishida M.; Nakajima T.; Honda Y.; Kitao O.; Nakai H.; Vreven T.; Throssell K.; Montgomery J. A.; Peralta J. E.; Ogliaro F.; Bearpark M.; Heyd J. J.; Brothers E.; Kudin K. N.; Staroverov V. N.; Keith T.; Kobayashi R.; Normand J.; Raghavachari K.; Rendell A.; Burant J. C.; Iyengar S. S.; Tomasi J.; Cossi M.; Millam J. M.; Klene M.; Adamo C.; Cammi R.; Ochterski J. W.; Martin R. L.; Morokuma K.; Farkas O.; Foresman J. B.; Fox D. J. Gaussian 09, Revision A.02, Gaussian, Inc., Wallingford CT, **2016**.

53. Chemcraft - graphical software for visualization of quantum chemistry computations. https://www.chemcraftprog.com

54. Hérault D.; Nguyen D.H.; Nuel D.; Buono G. *Chem. Soc. Rev.* **2015**, *44*, 2508-2528.
